# Subtype-Dependent Expression Patterns of Core Hippo Pathway Components in Thymic Epithelial Tumors (TETs): An RT-qPCR Study

**DOI:** 10.3390/biomedicines14020305

**Published:** 2026-01-29

**Authors:** Lisa Elm, Nadja Gerlitz, Jens Neumann, Georgia Levidou

**Affiliations:** Department of Pathology, Nuremberg Clinic, Paracelsus Medical University, 90419 Nuremberg, Germany; lisa.elm@klinikum-nuernberg.de (L.E.); nadja.gerlitz@klinikum-nuernberg.de (N.G.); jens.neumann@klinikum-nuernberg.de (J.N.)

**Keywords:** hippo signaling pathway, thymic epithelial tumors, RT-qPCR, relative gene expression, TETs, YAP1, TEAD4

## Abstract

**Background/Objectives:** Thymic epithelial tumors (TETs) are rare, histologically heterogeneous neoplasms lacking robust molecular biomarkers. Hippo pathway dysregulation—driving *YAP*/*TEAD*-dependent transcription—has been implicated across cancers, but transcript-level data in TETs are limited. **Methods:** We profiled 26 (23 TETs and three normal thymus) formalin-fixed and paraffin-embedded (FFPE) specimens by SYBR real-time quantitative polymerase chain reaction (RT-qPCR) across World Health Organization (WHO) subtypes, focusing on core Hippo components *YAP1*, *TEAD4*, *MST1*, *SAV1*, *LATS1*, and *MOB1A*. Expression was normalized to the geometric mean of *HPRT1* and *TBP* and reported as log_2_ fold change (log_2_FC) using the 2^−ΔΔCq^ method relative to the pooled normal. Group differences were compared using non-parametric tests. **Results:** Median log_2_FC values showed subtype-dependent upregulation of *YAP1*/*TEAD4*, notably in type A (*YAP1* ≈ +3.43) and B3 (*YAP1* ≈ +2.78) thymomas, with *TEAD4* strongly increased in thymic carcinoma (TC; ≈ +3.49) and elevated in type A/B3. Upstream kinases tended to be subtype-specifically reduced, particularly in TC (*MST1* ≈ −1.38; *LATS1* ≈ −1.34), and modestly in B1. *SAV1* was elevated in type A (≈+2.25) and B3 (≈+2.01), while *MOB1A* remained near baseline. Differential expression among WHO subtypes (Kruskal–Wallis) was significant for *YAP1* (*p* = 0.003), *TEAD4* (*p* = 0.015), *SAV1* (*p* = 0.004), *MST1* (*p* = 0.012), and *LATS1* (*p* = 0.036), but not for *MOB1A* (*p* = 0.09). **Conclusions:** TETs seem to exhibit subtype-dependent expression patterns of core Hippo pathway components, characterized by enhanced *YAP1*–*TEAD4* transcriptional output in selected subtypes and marked reduction of the *MST1*/*LATS1* kinase module, most pronounced in TC. These exploratory patterns nominate candidate markers for subtype stratification and clinical validation.

## 1. Introduction

Thymic epithelial tumors (TETs) are uncommon neoplasms arising from thymic epithelial cells, with heterogeneous clinical courses [1]. Although they comprise only 0.2–1.5% of all malignancies, they are the most frequent tumors of the anterior mediastinum [2]. Histology alone does not reliably predict biological behavior—particularly given that clinical aggressiveness spans from thymoma type A/AB through B1–B3 to thymic carcinoma (TC)—making risk stratification and the separation of indolent from aggressive disease challenging and underscoring the need for robust molecular biomarkers to improve diagnosis and prognosis [2,3].

A detailed understanding of the molecular basis of cancer is fundamental to designing effective, individualized therapies [4]. Among signaling circuits that drive tumorigenesis when deregulated, the Hippo pathway centrally regulates tissue and organ growth during development. Its disruption promotes malignancy by coupling unchecked proliferation with impaired apoptosis—two canonical cancer hallmarks [4]. In normal tissues, Hippo signaling acts as a growth-suppressive kinase cascade that constrains transcriptional programs driving proliferation. When the pathway is active (“Hippo on”), upstream kinases mammalian STE20-like kinase 1/2 (*MST1*/*STK4*; *MST2*/*STK3*) and the scaffold Salvador family WW domain-containing protein 1 (*SAV1*) promote activation of large tumor suppressor kinase 1/2 (*LATS1*/*2*) (with MOB kinase activator 1A/B (*MOB1A*/*B*) as cofactors). This leads to phosphorylation of Yes-associated protein 1 (*YAP1*) and transcriptional co-activator with PDZ-binding motif (*TAZ*/*WWTR1*) and their cytoplasmic retention and/or proteasomal degradation [2]. When Hippo signaling is inactive (“Hippo off”), dephosphorylated *YAP1*/*TAZ* translocate to the nucleus and bind TEA domain transcription factors 1–4 (*TEAD1–4*) to induce target genes involved in cell-cycle progression, survival, and tissue remodeling [2].

Hippo dysregulation arises from both genetic and non-genetic mechanisms and contributes to tumor development across entities—including glioma, breast, liver, lung, prostate, colorectal, and gastric cancers [5,6,7]. Importantly, diverse upstream disturbances appear to converge on a shared downstream output, activation of the transcriptional co-effectors *YAP1* and *TAZ*, which, through *TEAD1–4*, reprogram gene expression linked to tumor progression, metastasis, immune modulation, metabolic adaptation, and therapy resistance [4,6]. Humans encode four TEAD paralogs (*TEAD1–4*) that partner with *YAP1*/*TAZ*. Therapeutic efforts have therefore sought to interfere with YAP/TAZ–TEAD complex formation [6].

Prior work on Hippo signaling in TETs remains limited. Although key studies implicate this pathway, uncertainties persist regarding subtype-resolved expression patterns and their clinical relevance [2,8]. Moreover, Hippo activity appears context dependent, with reports across cancers noting differences related to subcellular localization (nuclear vs. cytoplasmic) and tumor type, highlighting the need for careful, subtype-aware analyses [2,6,8].

Building on our prior immunohistochemical (IHC) survey of TETs—which demonstrated widespread expression of core and upstream Hippo components and, notably, associated cytoplasmic TEAD4 with poorer overall survival [2]—we focused on a transcript-level characterization of the Hippo pathway in a subset of the original cohort. Specifically, we profiled quantitative real-time polymerase chain reaction (RT-qPCR) expression of the pathway’s output and control tiers—*YAP1*, *TEAD4*, and the upstream regulators *MST1*, *SAV1*, *LATS1*, and *MOB1A*—across histological subtypes, using pooled normal thymus as reference. Our primary aim was to describe the messenger RNA (mRNA) expression patterns of these core Hippo components across TET subtypes relative to normal thymus and to assess whether transcript-level differences qualitatively mirror and extend the protein-level alterations observed previously. We sought to define subtype-associated mRNA expression patterns of core Hippo components that may strengthen the molecular framework to improve our understanding of the molecular background of TETs that could support future molecular stratification of indolent vs. aggressive TETs.

## 2. Materials and Methods

### 2.1. Study Design and Ethical Considerations

This retrospective, anonymized study was conducted in accordance with the Declaration of Helsinki (institutional review board (IRB) statement IRB-2024-07 dated 5 April 2024).

### 2.2. Case Selection and Tissue Processing

This study includes 26 archival FFPE samples, comprising 23 thymic epithelial tumors (TETs) and three samples of non-neoplastic thymus (N1–N3), diagnosed between 2013 and 2023 in the Department of Pathology, Nuremberg Clinic (Nuremberg, Germany). The median age at diagnosis was 59 years (range 36–77 years), with a slight male predominance. Clinical and pathological characteristics refer to the 23 TET patients and are summarized in Table 1.

This collection of cases derives from a larger group of IHC-characterized cohort of 77 cases from our previous investigation [2]. The selection of cases to be included in the present investigation was based on block quality and available tissue, comprising normal thymus (N) (*n* = 3), thymoma type A (*n* = 3), thymoma B1–B3 (*n* = 5 each), and TC (*n* = 5). For each selected paraffin-embedded block, 5–10 sections of 5 µm thickness were prepared.

### 2.3. RNA Extraction and Nucleic Acid Quantification

Total RNA was isolated using the Maxwell^®^ RSC FFPE RNA Kit (Promega, Madison, WI, USA) following the manufacturer’s instructions, including a deparaffinization step and DNase I treatment. Because thymic tissue can harbor a physiologically high genomic DNA (gDNA) burden, RNA and residual DNA concentrations were quantified fluorometrically to guide downstream primer strategies (Qubit™, Thermo Fisher Scientific, Waltham, MA, USA). Spectrophotometric purity (A260/280) was assessed with a NanoDrop™ Lite (Thermo Fisher Scientific, Waltham, MA, USA; Table S1), with an expected ratio of 1.8–2.0. As RNA integrity number (RIN) and DV200 metrics are of limited utility for highly fragmented FFPE-derived RNA, functional integrity was assessed indirectly by consistent amplification of short amplicons of reference genes and by passing of the kit-provided internal control (IC) in all analyzed samples (Section 2.4).

### 2.4. One-Step RT-qPCR Workflow

Reverse transcription (RT) and qPCR were performed in a single-tube, two-phase protocol using the QuantiNova^®^ SYBR^®^ Green RT-PCR Kit (QIAGEN, Hilden, Germany, Cat. No. 208154) on a CFX96™ Real-Time PCR Detection System (Bio-Rad, Hercules, CA, USA), following the manufacturer’s instructions. Total nucleic acid input was titrated in pilot reactions (10–100 ng per 20 µL). A total of 50 ng per 20 µL provided consistent amplification, linear response, and acceptable replicate dispersion and was used for all analytical runs.

Reactions (20 µL) contained 10 µL 2× SYBR^®^ Green RT-PCR Master Mix, 0.2 µL QN SYBR^®^ Green RT-Mix, 2 µL 10× primer mix (0.5 µM each primer), 2 µL RNA template (50 ng) and 5.8 µL nuclease-free water, or an optional additional 1 µL QN IC RNA and 4.8 µL nuclease-free water. Cycling consisted of RT at 50 °C for 10 min, polymerase activation at 95 °C for 2 min, followed by 40 cycles of 95 °C for 5 s and 60 °C for 10 s. The melt curve analysis (65–95 °C; 0.2 °C increments for 5 s) was used to confirm single-product amplification. Cq values were calculated with CFX Manager™ Dx version 3.1 (Bio-Rad, Hercules, CA, USA), using a constant baseline and threshold across plates.

To ensure inter-run comparability in the absence of a pooled inter-run calibrator, two representative FFPE samples were re-analyzed in triplicate for selected targets (*YAP1*, *MOB1A*, TATA-box binding protein (*TBP*), and Hypoxanthine-guanine phosphoribosyl transferase 1 [*HPRT1*, RealTimePrimers.com (RTP)]) after scheduled replacement of the CFX96™ instrument (after sample 21) and across reagent lot changes, accepting an inter-run/instrument variation of ≤0.5–1 Cq (Tables S2–S4). In addition, potential plate effects were assessed using reference-gene Cq distributions, with *HPRT1* (RTP) and *TBP* showing strong cross-run correlation (Spearman’s rho = 0.88 and 0.89 for blocks 1 and 2; both *p* < 0.001) and no relevant inter-run deviations (Kruskal–Wallis, all *p* > 0.10; Table S3).

*SAV1* and *LATS1* were quantified in a subsequent run (block 2) due to reagent resupply, using the same reference genes (*HPRT1* (RTP) and *TBP*) and the same ΔΔCq normalization to the median of normal thymus samples (N1 and N3) as for all other targets.

**Figure A1:**
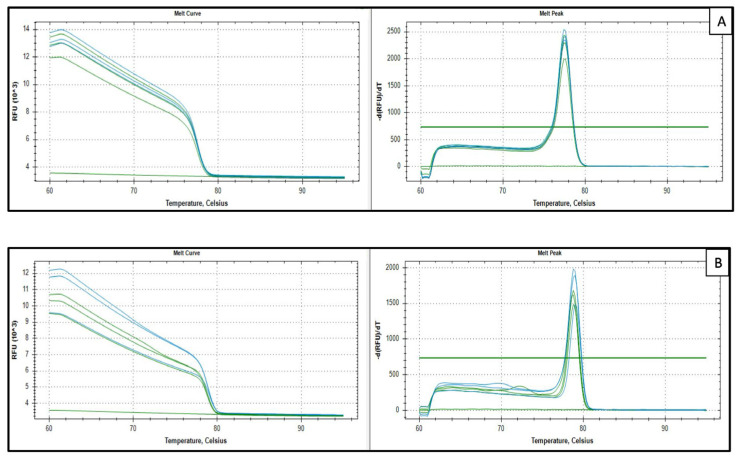
Representative melt curve analysis examples with their no-template control (NTC). (**A**) *HPRT1* ([RTP], Sample 20) and (**B**) *TEAD4* (Samples 1 and 2). Melt curve analysis (65–95 °C; 0.2 °C increments for 5 s) on a CFX96™ real-time PCR detection system (Bio-Rad, Hercules, CA, USA) verified single-product amplification. Cq values were calculated with CFX Manager™ Dx version 3.1 (Bio-Rad, Hercules, CA, USA) using a constant baseline and threshold across plates.

**Figure A2:**
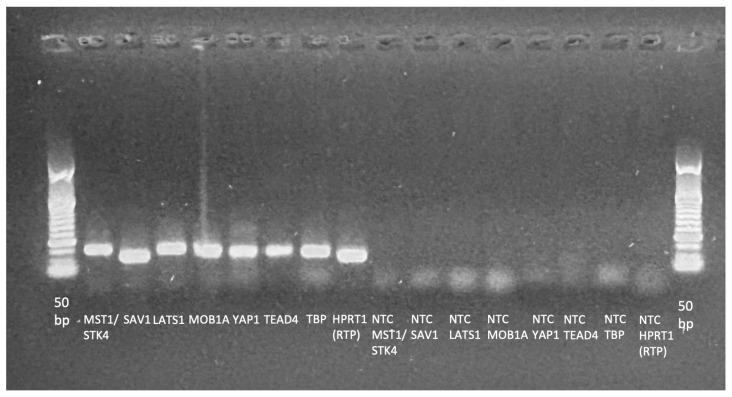
Agarose gel electrophoresis of the included assays with their NTCs. A 50 bp DNA ladder was run as a size marker to assign the approximate amplicon sizees (expected range: 94–141 bp). The image was acquired using an EZEE ClearView UV transilluminator (Cleaver Scientific, Rugby, UK). Electrophoresis was performed on a 1.5% agarose gel at 120 V for 20 min.

Each plate included no-template controls (NTC) for every assay and no-reverse-transcriptase controls (NRT) for every sample. NTCs were required to show no amplification or only late signals (Cq ≥ 37.5 within 40 cycles), and NRTs were required to be negative to exclude gDNA contribution. Assay specificity was confirmed by single-peak melt curves and single bands at expected amplicon sizes on agarose gels. Minor primer–dimer peaks at ~72 °C were occasionally observed for *TEAD4* (3/17 plates) and *YAP1* (1/17 plates) in NTCs only at very late Cq values (Cq ≥ 37.5) and were considered negligible for quantification. Representative melt curves and gel images are shown in Figure A1 and Figure A2. Full melt-curve sets are provided in Supplementary Figure S1.

An internal amplification control was included once per sample (QuantiNova Internal Control RNA (QN IC RNA); QuantiNova^®^ LNA^®^ PCR Reference Assay, QIAGEN, Hilden, Germany; Ref. No. 249920) to monitor RT/amplification performance and potential inhibition [9]. Runs were considered acceptable for ΔCq (sample − IC) < 2 (Table S5). No separate pooled positive calibrator was included; inter-run performance was monitored via the kit-provided IC and predefined quality criteria (QC) (Section 2.8), and expression values were normalized to validated reference genes (Section 2.6).

For *MOB1A*, the initial measurement of the normal thymus sample N1 failed quality control (all three technical replicates were invalid) and was therefore re-analyzed on an additional plate. The repeat Cq values were normalized using the same reference genes (*HPRT1* (RTP) and *TBP*), and N1 was retained as calibrator for *MOB1A* in combination with N3.

### 2.5. Primer Design, Procurement, and Assay Validation

Primers targeted core Hippo-pathway genes. PrimeTime™ qPCR Primers for *MST1*/*STK4* (Hs.PT.58.20785666), *SAV1* (Hs.PT.58.45488696), *LATS1* (Hs.PT.58.40644872), *MOB1A* (Hs.PT.58.40138473), *YAP1* (Hs.PT.58.22607088), *TEAD4* (Hs.PT.58.23238289), *TBP* (Hs.PT.58.20792004), peptidyl-prolyl isomerase A (*PPIA*; Hs.PT.39a.22214851), and *HPRT1* (Hs.PT.58.20881146) were obtained from Integrated DNA Technologies (IDT™, Coralville, IA, USA). *HPRT1* was also sourced from RealTimePrimers.com (Elkin Parks, PA, USA; VHPS-4263).

Amplicon lengths were 94–141 bp (FFPE-compatible). In-silico specificity (NCBI BLAST, web version) ensured transcript coverage and excluded pseudogene/gDNA matches. Despite DNase I treatment, low-level gDNA carryover was detectable in some NRT controls during early validation; therefore, assays were designed or replaced to span exon–exon junctions. Notably, *SAV1* (IDT™; Hs.PT.58.45727297) and *PPIA* showed sporadic NRT signals in a subset of specimens. *SAV1* was replaced by an alternative exon–exon assay (IDT™; Hs.PT.58.45488696), and *PPIA* was excluded from normalization (Section 3.3). In-run validation required a single melt curve peak and the absence of NRT signals.

Two different *TAZ*/*WWTR1* primers from IDT™ (Hs.PT.58.1944253 and Hs.PT.58.19363927) were initially included in the qPCR panel, but their SYBR Green assay showed low expression levels, recurrent primer–dimer amplification and/or non-specific amplification in NTCs and NRT controls. Because robust quantification could not be ensured, *TAZ* qPCR data were excluded from further analyses. *LATS1* (IDT™; Hs.PT.58.39498320) was also replaced because of non-specific amplification (Table S6 and Figure S1).

Details of the primer design, including forward and reverse primer sequence, amplicon size, exon location, and RefSeq number, are listed in the Supplementary Table S6.

**Figure A3:**
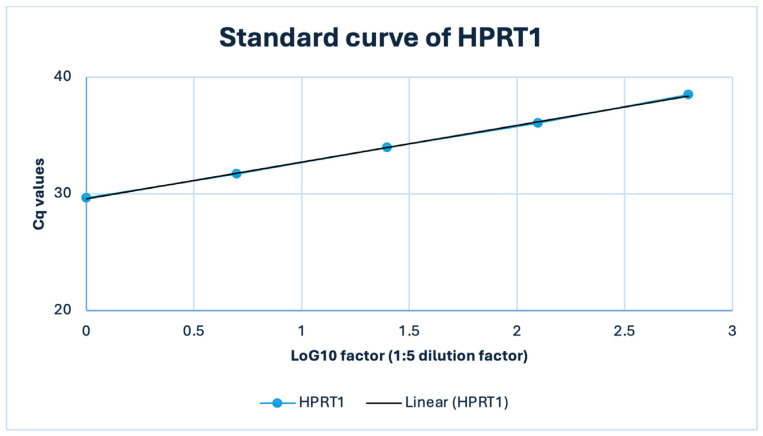
Standard curve of the *HPRT1* (RTP) qPCR assay in a representative FFPE thymic sample (TET B3). A 1:5 serial dilution of FFPE cDNA (50–0.08 ng input) yielded a highly linear relationship between Cq and log_10_ template amount (R^2^ = 1.0) with an amplification efficiency of approximately 107%.

PCR efficiency was assessed by 1:5 serial dilution of a representative FFPE-derived complementary DNA (cDNA) sample (50–0.4 ng input; Table S7), demonstrating log-linear amplification within the working range, and a representative *HPRT1* (RTP) dilution series showed a linear relationship between Cq and log_10_ template amount (R^2^ = 1.00) with an apparent efficiency of ~107% (E = 2.07; Figure A3). Because FFPE-based dilution series can yield apparent efficiencies > 100% due to matrix/template-related artefacts rather than true reaction kinetics, assay-specific efficiency correction was not applied, and relative expression was calculated using the comparative 2^−ΔΔCq^ method assuming approximately similar amplification behavior across assays (E ≈ 2) [10,11,12,13,14].

### 2.6. Reference Gene Strategy and Stability Assessment

**Figure A4:**
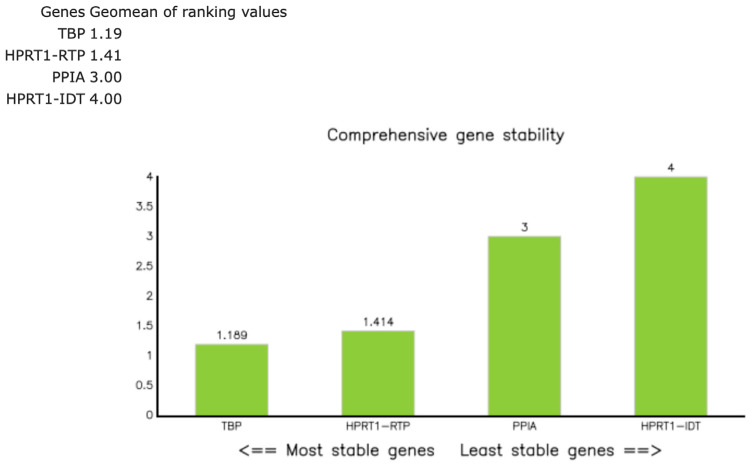
RefFinder composite stability ranking of the candidate reference genes *TBP*, *HPRT1* (RTP), *PPIA*, and *HPRT1* (IDT™) in 26 thymic samples. The green bars represent the RefFinder composite rank values, calculated as the geometric mean of the stability ranks obtained from geNorm, NormFinder, BestKeeper, and the comparative ΔCq method. The lower values indicate more stable expression. *TBP* (1.19) and *HPRT1* ([RTP]; 1.41) showed the highest stability (composite value ≤ 1.5), whereas *PPIA* (3.00) and *HPRT1* ([IDT™]; 4.00) were less stable. Consequently, the geometric mean of *HPRT1* (RTP) and *TBP* was used for normalization in subsequent analyses [18].

Candidate reference genes included *HPRT1* (RTP), *HPRT1* (IDT™), *TBP*, and *PPIA*, selected based on prior use in thymic tissues/FFPE qPCR and reported stability in relative expression analyses [15,16,17]. These candidates were assayed across all 26 samples (Tables S8 and S9). Expression stability was evaluated with RefFinder (composite of geNorm, NormFinder, BestKeeper, and ΔCq approaches) [18]. The a priori criterion was the lowest composite rank across groups, with composite values ≤ 1.5 considered indicative of good stability and values > 2–3 suggesting suboptimal reference genes. RefFinder identified *HPRT1* ([RTP]; composite rank 1.41) and *TBP* (1.19) as the most stable reference genes (Figure A4; Table S10), and their geometric mean was used for normalization. *HPRT1* (IDT™) ranked lower (4.00), and *PPIA* (3.00) was excluded due to sporadic gDNA-related amplification (Section 2.5).

To mitigate run-specific variation in downstream ΔΔCq/log_2_FC estimates, the pooled median of the two normal thymus samples (N1/N3) served as the common calibrator across runs (Section 2.7).

### 2.7. Quantification and Data Processing

Primary data were Cq values from technical triplicates. Outlier handling followed Section 2.8. Relative expression was computed as 2^−ΔΔCq^ (reported as FC), normalizing to the geometric mean of *HPRT1* (RTP) and *TBP*. For visualization and statistical analyses, values were additionally expressed as log_2_FC (Tables S11 and S12). Calculations were performed in Excel (Microsoft, Redmond, WA, USA; version 16.100.3). The calibrator was defined as the median of the two histologically normal thymus samples (N1 and N3), which showed low variability in Hippo pathway gene expression (log_2_FC near 0), whereas the third non-neoplastic sample (N2) was excluded due to benign cystic histology with sparse thymic parenchyma/reduced epithelial content and atypically high ΔCq values indicating a divergent expression profile.

### 2.8. Replicates and Quality Criteria

All reactions were run in technical triplicate and assessed for intra-assay variability (Tables S13 and S14). Triplicates with all three Cq values within ≤ 0.5 Cq were considered technically consistent (“gold standard”). For FFPE-derived RNA, a total spread of up to 0.8 Cq was accepted (“FFPE accepted”). Any replicate deviating by > 0.5 Cq from the mean of the two most consistent replicates was defined as a technical outlier and excluded from mean and standard deviation (SD) calculations. Reactions without two consistent replicates (ΔCq > 0.8) were classified as invalid and repeated. Biological replication was provided by independent tissue specimens per diagnostic group (N, A, B1, B2, B3, and TC).

### 2.9. Statistics

Log_2_FC expression values were tested for normality. Group comparisons were tested using non-parametric tests (Mann–Whitney U test or Kruskal–Wallis ANOVA as appropriate) with correction for multiple comparisons (Benjamini–Hochberg false discovery rate). Correlations were assessed by Spearman’s correlation coefficient. The significance level was α = 0.05 (two-sided). Analyses were performed using STATA software 11.0 (STATA/SE, StataCorp LP, College Station, TX, USA).

### 2.10. Immunohistochemical (IHC) Data

A subset of previously generated IHC data from the same cohort was used only for qualitative contextualization of RT-qPCR findings. IHC staining and scoring were performed as described in our prior study [2]. Key conditions are summarized in Table S15.

### 2.11. Reporting Standards

Methods and reporting adhere to the core recommendations of the original Minimum Information for Publication of Quantitative Real-Time PCR Experiments (MIQE) guidelines (2009) [10] and were further designed in alignment with key MIQE 2.0 updates [19], with detailed assay information and raw data provided in the Supplementary Materials.

## 3. Results

### 3.1. Relative Expression of Hippo Pathway Genes

Relative expression levels of six Hippo pathway genes (*MST1*, *SAV1*, *LATS1*, *MOB1A*, *YAP1*, and *TEAD4*) are presented for descriptive and statistical reporting as log_2_FC relative to the median of normal thymus samples (N1 and N3) as the calibrator (2^−ΔΔCq^). An FC of 1.0 (log_2_FC = 0) corresponds to the expression level in normal thymic tissue, with expression levels normalized to the housekeeping genes *HPRT1* (RTP) and *TBP*. For readability, the corresponding approximate FCs (~x-fold) derived from the log_2_FC values are shown in parentheses where appropriate. For each gene, median log_2_FC values were determined for TET subtypes (A, B1–B3, and TC) and are reported descriptively, highlighting inter-subtype differences and, where evident, intra-group heterogeneity. Individual sample values are visualized as boxplots to illustrate the distribution within each subtype.

The analyzed targets are presented in Hippo pathway order, from upstream kinases and scaffolding components (*MST1*, *SAV1*, *LATS1*, and *MOB1A*) to the transcriptional coactivator (*YAP1*) and its nuclear effector (*TEAD4*).

**Figure 1:**
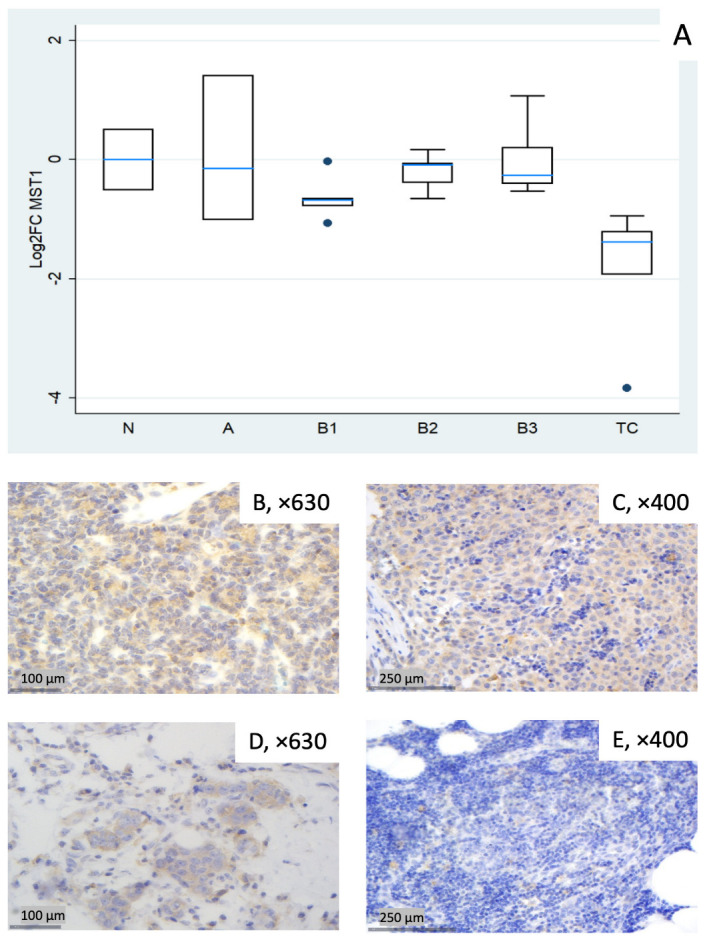
*MST1* expression in thymic epithelial tumors (TETs). (**A**) Boxplots depicting *MST1* mRNA expression among WHO histological subtypes of TETs. (**B**–**E**) Representative IHC images of cytoplasmic MST1 expression in (**B**) type A thymoma (×630), (**C**) type B3 thymoma (×400), (**D**) TC (×630), and (**E**) normal thymic tissue (×400).

**Figure 2:**
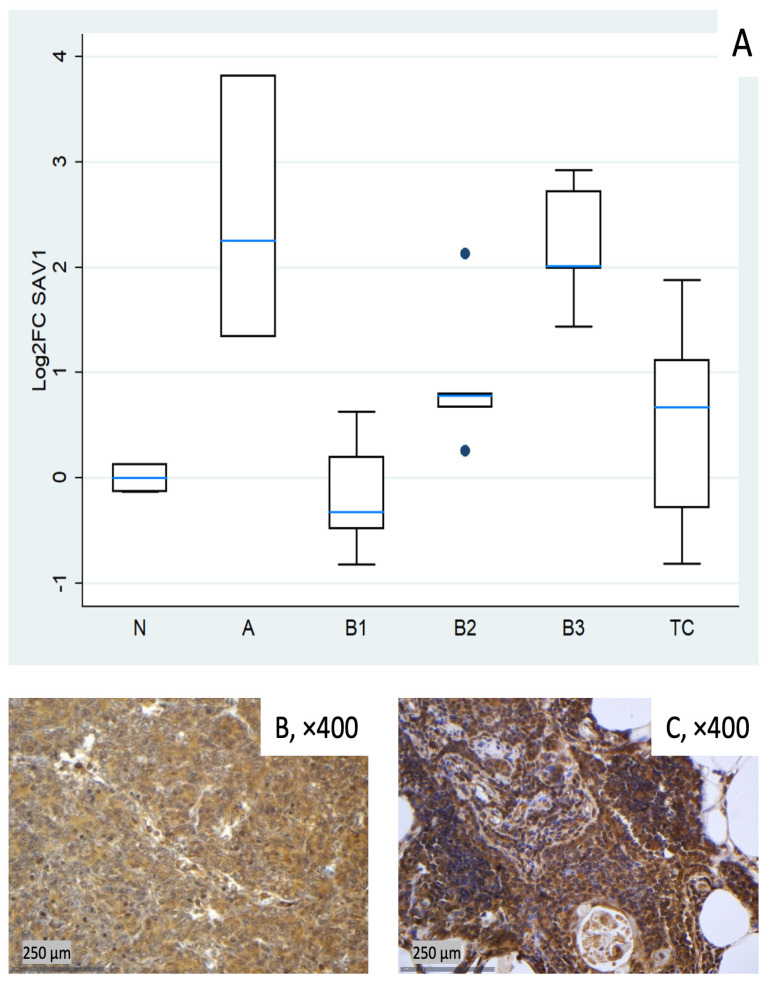
*SAV1* expression in TETs. (**A**) Boxplots depicting *SAV1* mRNA expression across WHO histological subtypes of TETs. (**B**,**C**) Representative IHC images of cytoplasmic SAV1 expression in (**B**) type B3 thymoma (×400) and (**C**) normal thymic tissue (×400).

**Figure 3:**
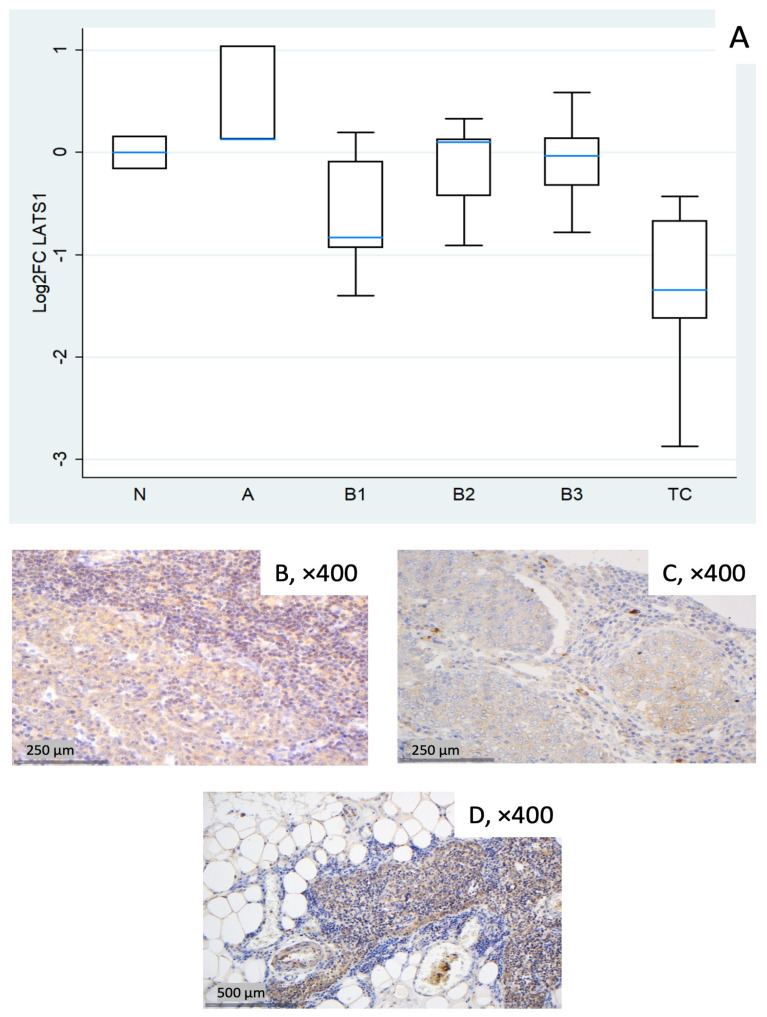
*LATS1* expression in TETs. (**A**) Boxplots depicting *LATS1* mRNA expression across WHO histological subtypes of TETs. (**B**–**D**) Representative IHC images of cytoplasmic LATS1 in (**B**) type A thymoma showing strong expression (×400), (**C**) TC with moderate staining (×400), and (**D**) normal thymic tissue (×400).

**Figure 4:**
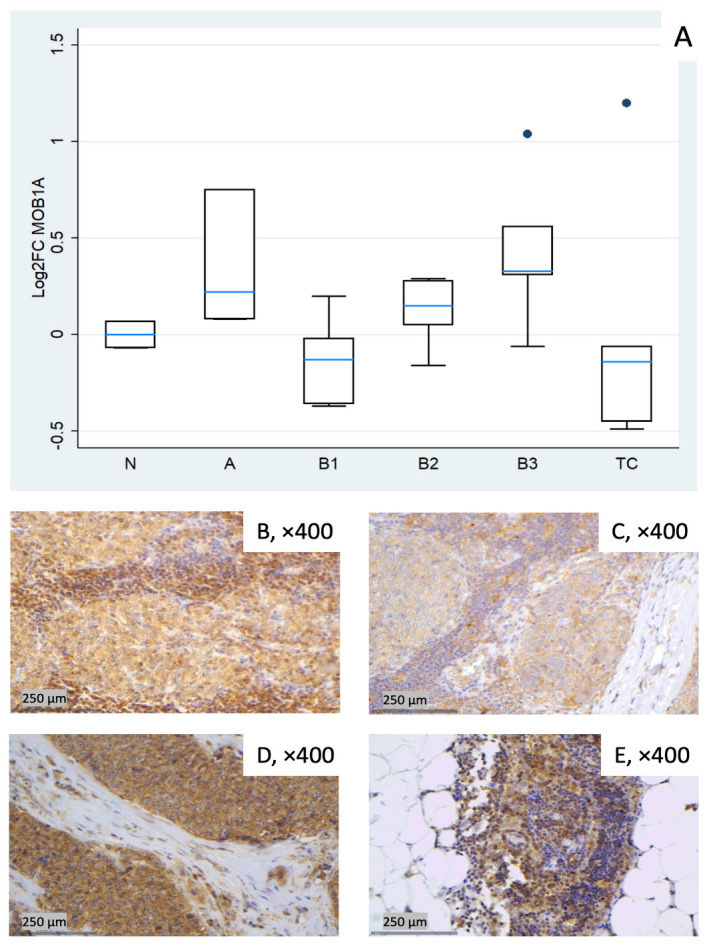
*MOB1A* expression in TETs. (**A**) Boxplots depicting *MOB1A* mRNA expression across WHO histological subtypes of TETs. (**B**–**E**) Representative IHC images of cytoplasmic MOB1A expression in (**B**) type A thymoma (×400), (**C**) type B3 thymoma (×400), (**D**) TC (×400), and (**E**) normal thymic tissue (×400).

**Figure 5:**
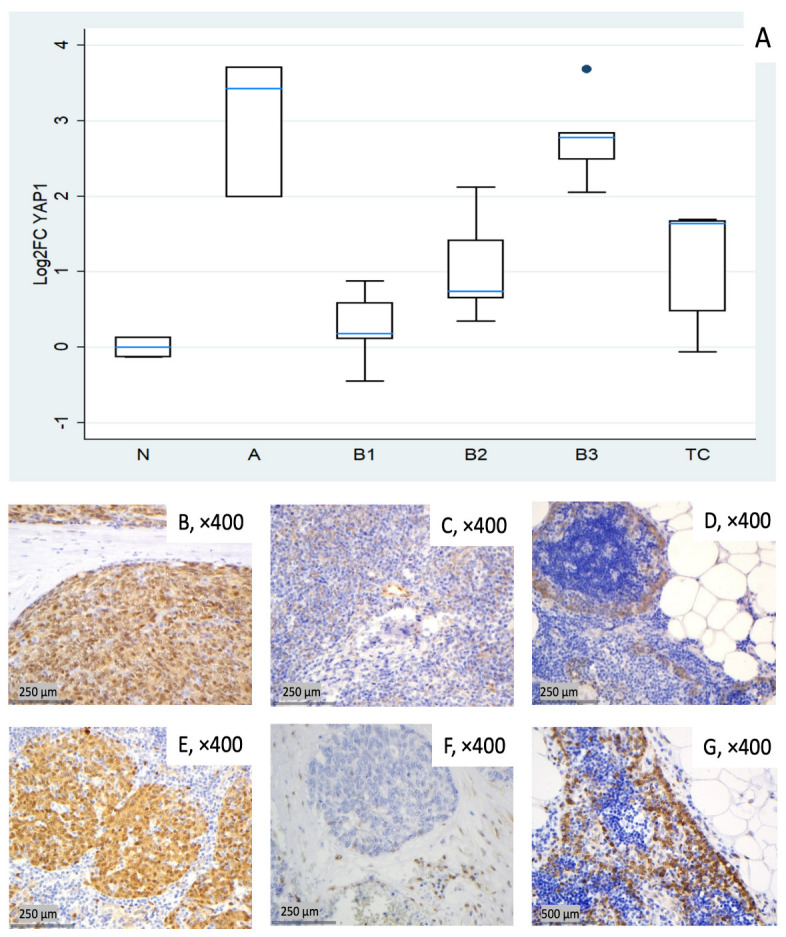
*YAP1* expression in TETs. (**A**) Boxplots depicting *YAP1* mRNA expression across WHO histological subtypes of TETs. (**B**–**D**) Representative IHC images showing nuclear and cytoplasmic YAP1 staining in (**B**) type A thymoma (×400), (**C**) type B2 thymoma with moderate staining (×400), and (**D**) normal thymic tissue (×400). (**E**–**G**) Representative IHC images of nuclear and cytoplasmic expression of active YAP1 (AYAP) in (**E**) type A thymoma with strong staining (×400), (**F**) TC with low nuclear but moderate cytoplasmic staining (×400), and (**G**) normal thymic tissue (×400).

**Figure 6:**
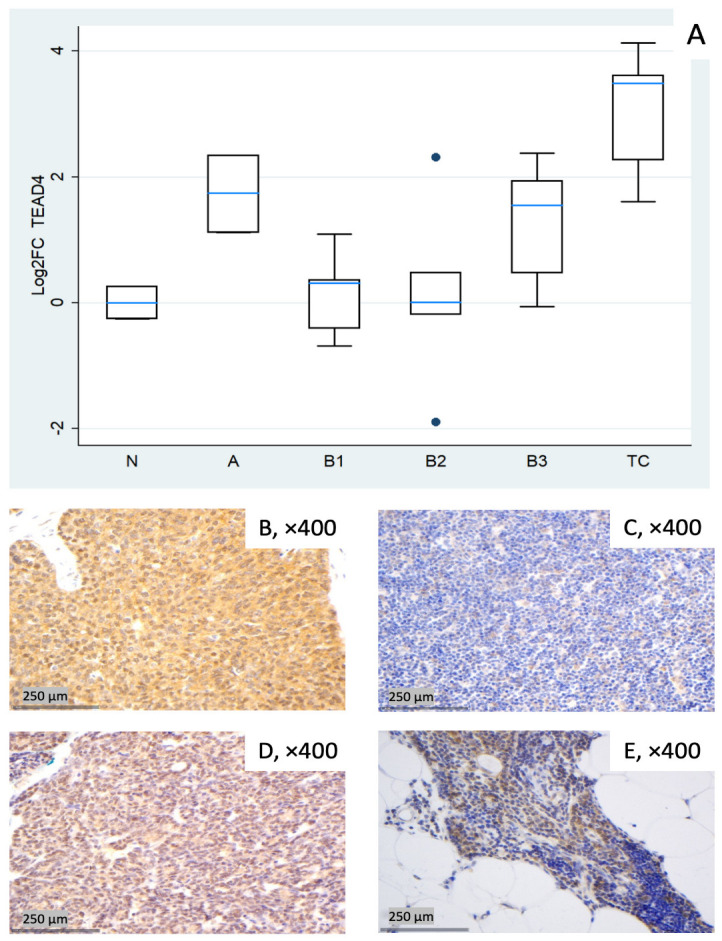
*TEAD4* expression in TETs. (**A**) Boxplots depicting *TEAD4* mRNA expression across WHO histological subtypes of TETs. (**B**–**E**) Representative IHC of nuclear and cytoplasmic TEAD4 expression in (**B**) TC with moderate nuclear and strong cytoplasmic staining (×400), (**C**) type B1 thymoma with low staining (×400), (**D**) type A thymoma with strong staining (×400), and (**E**) normal thymic tissue staining (cytoplasmic, nuclear, ×400).

For overview and visual context, representative (illustrative) IHC micrographs are shown for each target (Figure 1, Figure 2, Figure 3, Figure 4, Figure 5 and Figure 6). To aid interpretation of transcript–protein relationships, we provide a cohort-level summary of IHC subcellular localization in Figure S2. As expected, MST1, SAV1, LATS1, and MOB1A showed exclusively cytoplasmic staining, whereas YAP1, active YAP1 (AYAP) and TEAD4 displayed variable nuclear positivity in addition to cytoplasmic staining.

#### 3.1.1. Relative Expression of MST1

*MST1* showed broadly lower transcript levels across subtypes, most pronounced in TC (median log_2_FC = −1.38, corresponding to ~0.4-fold relative to normal thymus, Mann–Whitney U test, TC vs. all other groups (N, A, B1–B3), *p* = 0.002) and in B1 (median log_2_FC = −0.68, ~0.6-fold). Type A displayed notable dispersion (roughly log_2_FC −1.0 to +1.4 across cases), yielding a slightly negative median overall. B2 and B3 were modestly below normal (Figure 1A). A Kruskal–Wallis test restricted to tumor subtypes confirmed a statistically significant differential expression of *MST1* among different World Health Organization (WHO) histological subtypes (*p* = 0.012). Representative IHC micrographs (Figure 1B–E) depict the observed range of cytoplasmic MST1 staining and show partial overlap with the mRNA distribution in Figure 1A.

#### 3.1.2. Relative Expression of SAV1

*SAV1* tended to be clearly elevated in type A thymomas (median log_2_FC = +2.25, ~5-fold) and in B3 (log_2_FC = +2.01, ~4-fold), with modest increases in B2 and TC (around median log_2_FC +0.7). B1 centered around baseline with low dispersion (Figure 2A). These differences among different histological TET subtypes were proven to be statistically significant (Kruskal–Wallis, *p* = 0.004). Representative IHC images (Figure 2B,C) demonstrate the spectrum of cytoplasmic SAV1 protein expression across the cohort and qualitatively correspond to the mRNA distribution shown in Figure 2A.

#### 3.1.3. Relative Expression of LATS1

*LATS1* showed a marked reduction in TC (median log_2_FC = −1.34, ~0.4-fold; Mann–Whitney U test: TC vs. all other groups (N, A, B1–B3), *p* = 0.008). B1 showed a mild decrease (median log_2_FC = −0.83, ~0.6-fold) with wide dispersion, whereas types A, B2, and B3 were near baseline on median (Figure 3A). The Kruskal–Wallis test confirmed a statistically significant differential expression of *LATS1* among different WHO histological subtypes (*p* = 0.036). Representative IHC panels (Figure 3B–D) highlight inter-case variability in cytoplasmic LATS1 staining and partly reflect the RT-qPCR distribution in Figure 3A.

#### 3.1.4. Relative Expression of MOB1A

*MOB1A* remained largely stable across TET subtypes (Kruskal–Wallis, *p* = 0.09). Modest increases were observed in type A (median log_2_FC = +0.22) and in B3 (median log_2_FC = +0.33), while slight decreases were observed in B1 (median log_2_FC = −0.13) and TC (median log_2_FC = −0.14). B2 clustered near baseline with low-to-moderate dispersion (Figure 4A). Representative IHC images (Figure 4B–E) illustrate the range of cytoplasmic MOB1A protein expression observed in the cohort and show variable correspondence with the mRNA distribution in Figure 4A.

#### 3.1.5. Relative Expression of YAP1

In this cohort, *YAP1* expression tended to be elevated across all tumor subtypes. Type A thymomas (median log_2_FC = +3.43, ~11-fold) and B3 thymomas (median log_2_FC = +2.78, ~7-fold) showed the highest median values, followed by TC (median log_2_FC = +1.64, ~3-fold). B1 and B2 showed lower medians (median log_2_FC 0.18 to 0.74) but marked inter-sample variability, ranging from near baseline to moderately elevated expression (Figure 5A). Kruskal–Wallis confirmed the statistically significant differential expression among TET subtypes (*p* = 0.003). Representative IHC images (Figure 5B–G) demonstrate the spectrum of cytoplasmic YAP1 protein expression across the cohort, consistent with the mRNA pattern shown in Figure 5A.

#### 3.1.6. Relative Expression of TEAD4

*TEAD4* showed pronounced upregulation in TC (median log_2_FC = +3.49, ~11-fold; Mann–Whitney U test: TC vs. all other groups (N, A, B1–B3), *p* = 0.004) and clear increases in type A (median log_2_FC = +1.74, ~3- to 4-fold) and B3 (median log_2_FC = +1.55, ~3-fold). B1 remained close to baseline yet was dispersed (log_2_FC about −0.7 to +1.1), and B2 exhibited a widespread distribution with a near-zero median, indicating heterogeneity rather than a uniform shift (roughly log_2_FC −1.9 to +2.3 across cases, Figure 6A). These differences among different histological TET subtypes were proven to be statistically significant (Kruskal–Wallis, *p* = 0.015). Representative IHC panels (Figure 6B–E) highlight inter-case variability in TEAD4 staining and show qualitative agreement with the RT-qPCR distribution in Figure 6A.

### 3.2. Consolidated Overview of the Relative Expression Results of Hippo Pathway Genes

Table 2 summarizes the sample-wise fold changes (FCs) and corresponding log_2_FC values for the core Hippo pathway components, while Table 3 presents the subtype-specific median log_2_FC values.

**Figure 7:**
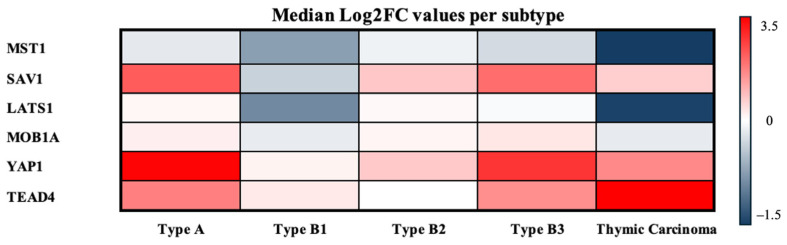
Median log_2_FCs per TET subtype. Heatmap of median log_2_FCs for Hippo pathway components across TET subtypes. Warmer colors indicate higher expression relative to normal, and cooler colors indicate lower expression.

To provide an integrated overview of these expression patterns across TET subtypes, the median log_2_FC values of all Hippo components were subsequently visualized in a heatmap (Figure 7).

### 3.3. Contextualization of Subtype-Associated Hippo Pathway Expression Using the Cancer Genome Atlas Thymoma (TCGA-THYM) RNA-Sequencing Data

To contextualize our exploratory RT-qPCR findings within an independent dataset, subtype-resolved mRNA expression patterns of selected Hippo pathway components were examined in The Cancer Genome Atlas Thymoma (TCGA-THYM) RNA-sequencing cohort via cBioPortal (study: Thymoma (TCGA, Firehose Legacy), accessed 20 January 2026, Figure S3) [20]. Due to platform- and normalization-related differences between RNA-sequencing and RT-qPCR, comparisons were limited to the directionality of subtype-associated trends.

Across the TCGA-THYM cohort, *MST1* expression varied across histological subtypes but showed a comparatively heterogeneous distribution, with less consistent directional overlap with our FFPE-based RT-qPCR measurements. *SAV1* displayed higher expression in type A thymomas and lower levels in B1 tumors, broadly matching the direction of change observed in our RT-qPCR series. *LATS1* likewise exhibited subtype-dependent variation in TCGA-THYM, but concordance with our cohort was less pronounced than for downstream targets. *MOB1A* showed comparatively limited variation across subtypes, consistent with the largely stable expression pattern observed in our RT-qPCR dataset.

**Figure 8:**
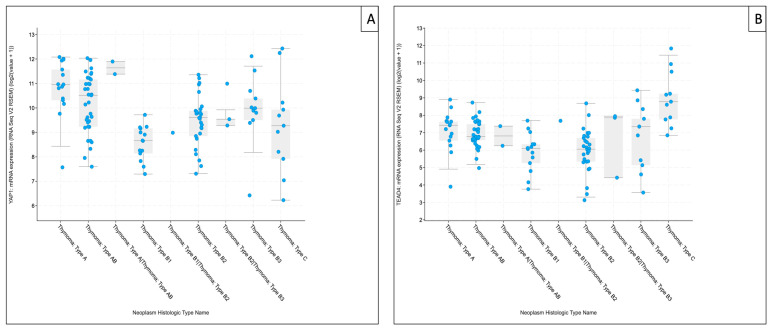
The Cancer Genome Atlas Thymoma (TCGA-THYM) RNA-sequencing–based mRNA expression of downstream Hippo pathway components. Boxplots show *YAP1* (**A**) and *TEAD4* (**B**) expression across TCGA-THYM histological annotations. Values represent cohort-normalized RNA-sequencing expression (log2[RSEM + 1]; platform-specific units). Points denote individual tumor samples (*n* = 124 samples/patients). Data were retrieved via cBioPortal for Cancer Genomics (study: Thymoma (TCGA, Firehose Legacy), accessed on 20 January 2026) [20].

For downstream components, *YAP1* tended to be higher in type A and B3 thymomas and remained higher than B1/B2 subtypes, mirroring the directionality observed in our RT-qPCR analysis. *TEAD4* showed the clearest subtype-associated gradient in TCGA-THYM, with the highest median expression in TC and comparatively higher medians also in type A and type B3 thymomas, whereas B1/B2 subtypes exhibited lower median levels—aligning directionally with our cohort, despite marked within-subtype variability. For clarity and focus on these downstream effectors, TCGA-THYM expression plots for *YAP1* and *TEAD4* are shown in the main manuscript (Figure 8A,B), whereas corresponding analyses for upstream Hippo components (*MST1*, *SAV1*, *LATS1*, and *MOB1A*) are provided in Supplementary Figure S3.

## 4. Discussion

TETs are rare neoplasms of the anterior mediastinum with pronounced histological heterogeneity and a wide range of clinical behavior, making diagnostic and prognostic assessment based on histology alone challenging [1,2,3,21,22]. The Hippo signaling pathway, a central regulator of tissue growth and tumor progression, has emerged as relevant in TETs [2,8,23,24,25,26,27]. However, prior work has focused predominantly on IHC analyses [2,8], while systematic transcript-level data remain scarce and the functional implications of observed alterations are largely unclear [28]. Beyond IHC, integrative genomic and transcriptomic studies have defined molecular classes aligned with histology and outcome and highlighted substantial microenvironmental heterogeneity in TETs [29,30,31]. In this exploratory study, we profiled the mRNA expression of the core Hippo pathway components *YAP1*, *TEAD4*, *MST1*, *SAV1*, *LATS1*, and *MOB1A* in TETs using RT-qPCR across WHO subtypes and related these patterns to existing IHC data (Table S16) from the same cohort.

Taken together, based on the median log_2_FC values, our RT-qPCR data indicate subtype-associated differences in core Hippo gene expression, with upregulated *YAP1* and *TEAD4* transcripts in type A and B3 thymomas and in TCs, alongside reduced *MST1* and *LATS1* levels, which are most pronounced in carcinomas. In contrast, adaptor proteins display a divergent pattern, with *SAV1* tending to be increased in type A and B3 thymomas, whereas *MOB1A* remains largely stable across subtypes. Collectively, these findings support a subtype-linked variation in Hippo-related transcriptional output rather than a uniform shift across TETs [31,32]. In other epithelial malignancies, pathway-focused analyses suggest that Hippo status is often better captured by coordinated YAP/TAZ–TEAD programs than by single-gene measurements, consistent with interpreting our RT-qPCR results primarily as subtype-associated transcriptional signatures [24,32,33]. The observed *YAP1*/*TEAD4* pattern aligns with reports in other cancers (e.g., ovarian cancer, lung adenocarcinoma, urothelial carcinoma) linking YAP/TEAD activity to proliferation, epithelial–mesenchymal transition (EMT), stemness features, immune modulation, and adverse outcomes [33,34,35,36,37,38,39,40,41]. While TETs are genetically less complex than many other solid cancers [21,30,31], the convergence of our mRNA data with this broader literature supports the biological plausibility that enhanced *YAP1*–*TEAD4* transcriptional output may also play a role in more aggressive behavior in a subset of TETs [36,42,43,44]. Lower *MST1* and *LATS1* transcript levels are compatible with their tumor-suppressive roles in the canonical Hippo cascade [45,46,47], while increased *SAV1* may reflect compensatory regulation or context-dependent pathway rewiring rather than simple loss-of-function [23,47,48,49,50]. Overall, the combination of these kinase-module reductions with preserved or elevated *SAV1* and largely stable *MOB1A* expression suggests a component-specific modulation of the Hippo kinase cassette at the transcript level in TETs, rather than a uniform downshift across all measured components [27,47,51,52].

This transcript signature partially mirrors our IHC findings and complements previous IHC studies of Hippo signaling in TETs [1,2,3,8]. Palamaris et al. reported widespread expression of *YAP1*, *TAZ*, *LATS1*, and *TEAD4* in thymomas and TCs with complex nuclear–cytoplasmic localization patterns [8]. In a separate IHC cohort, we similarly observed strong expression of core and upstream Hippo components and linked high cytoplasmic TEAD4 to poorer overall survival [2]. In the present study, higher *YAP1* transcript levels—most evident in type A and B3 thymomas—were broadly consistent with stronger YAP1/AYAP staining, whereas *TEAD4* showed subtype-associated transcript elevation (particularly in TC) alongside predominantly strong cytoplasmic staining but highly variable nuclear positivity. Notably, individual tumors exhibited discordant *TEAD4* transcript and staining patterns, supporting the concept that TEAD4 localization and nuclear engagement may be influenced by regulatory mechanisms beyond transcript abundance, including context-dependent YAP/TAZ–TEAD dynamics [32,35,51,52,53,54]. In contrast, *MST1*, *SAV1*, *LATS1*, and *MOB1A* showed only modest or heterogeneous transcript changes despite consistently intense cytoplasmic protein expression. Such transcript–protein discrepancies are expected for Hippo signaling, where functional pathway state is largely determined by post-transcriptional and post-translational control rather than simple on/off shifts in transcript abundance [22,45,51,52,53]. Several methodological aspects likely contribute to these transcript–protein discrepancies. Bulk RT-qPCR from FFPE tissue captures mixed cell populations, whereas IHC scoring was restricted to tumor cells. Differences in thymocyte/stromal admixture may therefore contribute to transcript–protein discrepancies and attenuate apparent subtype differences [53]. Single-cell and immune-profiling studies of TETs further underscore subtype-associated variability in epithelial and immune/stromal composition, reinforcing that bulk transcript measurements can be influenced by microenvironmental admixture [54,55,56]. Moreover, IHC is inherently semi-quantitative and, in our cohort, showed clear ceiling effects for several Hippo core components (e.g., MOB1A, SAV1, and LATS1). This narrow dynamic range is particularly relevant for protein-stable molecules whose levels are regulated predominantly beyond the transcript level [57,58,59]. In addition, IHC captures compartmental localization (nuclear vs. cytoplasmic), which may change independently of transcript abundance and further weaken sample-level mRNA–protein concordance. Biologically, discordance between transcript abundance and protein staining is expected for Hippo signaling because pathway state is primarily determined by phosphorylation, protein turnover, and subcellular localization rather than mRNA levels [47,60]. Upstream kinases and scaffold proteins may remain protein-stable and highly expressed, while their activity (or phosphorylation state) changes [47,60], whereas YAP/TAZ–TEAD output can be modulated by mechanotransduction [47], cell–cell contact [60,61], and feedback loops that do not necessarily scale with transcript abundance [62]. In addition, transcript isoform usage and differential mRNA/protein half-lives can decouple mRNA from IHC intensity [63,64]. Technically, FFPE-related RNA fragmentation, variable tumor cellularity, regional sampling differences between sections used for RT-qPCR vs. IHC, and antibody epitope performance can further contribute. Together, these considerations underscore that mRNA and protein provide complementary views and that transcript abundance alone is an incomplete proxy for pathway state, particularly for upstream regulators [23,47,57,58,59,65].

When viewed against the broader molecular landscape of TETs defined by large-scale sequencing efforts, our findings add a pathway-focused perspective rather than uncovering new driver events [28,30,31,66,67]. Integrated genomic analyses, such as the TCGA study by Radovich et al. and the more recent multiomics work by Möhrmann et al., indicate that TETs are characterized by relatively low mutational burdens. The TCGA analysis defined four molecular subtypes closely aligned with WHO histology and survival and highlighted the thymoma-enriched General Transcription Factor II-I (*GTF2I*) L424H mutation (particularly in type A/AB), alongside enrichment of *HRAS*/*NRAS*/*TP53* alterations and increasing aneuploidy/genomic complexity in more aggressive tumors [30,31]. Within this framework, higher *YAP1*/*TEAD4* transcript levels in type A, B3, and TC may reflect subtype-specific transcriptional programs superimposed on distinct genomic backgrounds rather than a shared “Hippo-on/Hippo-off” state. Recent multi-omic precision oncology cohorts further subdivide TCs into immune-infiltrated (“hot”) and immune-poor (“cold”) groups with different outcomes, suggesting that pathway signals, such as *YAP*/*TEAD*, should ultimately be interpreted together with immune context and broader molecular classes [30]. Additional independent genomic studies have similarly reported recurrent driver patterns and subtype-associated molecular profiles in TETs, supporting the view that pathway-associated signals should be interpreted within established molecular classes [29,68]. Accordingly, Hippo pathway-related alterations appear to represent one of several converging signaling alterations rather than a single dominant driver [30,31]. Our findings therefore fit with a model in which Hippo pathway changes modulate, rather than fully determine, the biological behavior of TETs and act in concert with other genomic and microenvironmental factors [50,69,70,71]. Consistent with this interpretation, subtype-level expression trends for *YAP1* and *TEAD4* observed in our RT-qPCR cohort showed similar directional patterns in the independent TCGA-THYM RNA-sequencing dataset, supporting cross-cohort contextualization of our exploratory findings.

The present work provides, to our knowledge, one of the first dedicated transcript-level analyses of core Hippo pathway components in TETs and integrates these data with previously established IHC patterns [2], but it has methodological limitations. First, analyses were performed on FFPE material, including archival blocks of up to ten years of age, which is associated with RNA fragmentation and pre-analytical variability [72,73]. Nonetheless, stable reference gene performance and concordant trends across related genes and subtypes argue against random artefacts and support the robustness of the main expression patterns. Second, the number of cases per histological subtype was limited, reducing statistical power, and underscoring the exploratory, hypothesis-generating nature of subtype-resolved comparisons. Although multiple-testing correction was applied, smaller effect sizes may have gone undetected and subtype-level estimates should be interpreted cautiously. In addition, bulk RT-qPCR does not resolve cellular heterogeneity. In TETs, variable admixture of thymocytes and stromal components can dilute tumor-epithelial transcripts and confound subtype comparisons, particularly when epithelial tumor cellularity differs across WHO subtypes. Third, despite careful assay design and run-to-run quality control, residual technical variability cannot be fully excluded, as is typical for FFPE-based transcript studies. Moreover, thymic tissue and TETs harbor high amounts of gDNA, which could not be completely removed even by repeated DNase treatment. We therefore used exon–exon spanning assays with partially larger amplicon length, which are suboptimal for FFPE but still showed reproducible, linear amplification [10,74]. Within these constraints, the recurrent upregulation of *YAP1*/*TEAD4* and the reduction of *MST1*/*LATS1* in more aggressive subtypes are likely to be robust and biologically meaningful, whereas subtle FCs should be interpreted cautiously.

Although this study offers an integrated transcript-level overview of Hippo pathway components in TETs, several questions remain open. A key next step will be to validate these expression patterns in larger, independently collected cohorts with comprehensive clinicopathological and outcome data. In parallel, functional studies in appropriate thymic epithelial model systems will be required to define how Hippo pathway alterations translate into cellular phenotypes and treatment responses [22,23,55]. Integrating RT-qPCR and IHC with higher-dimensional approaches, including whole-transcriptome profiling, genomic and epigenomic analyses, as well as single-cell or spatial methods to capture tumor–immune interactions, may further help place Hippo dysregulation within the broader molecular context of TETs and to identify co-operating pathways. Proteomic and phosphoproteomic approaches could help map Hippo pathway activity and phosphorylation patterns, thereby capturing post-translational regulation that may not be reflected at the mRNA level. Finally, as pharmacologic inhibitors targeting the YAP/TAZ–TEAD interface are entering early clinical development, our findings nominate Hippo-related candidates for further evaluation in TETs and provide a rationale for incorporating validated Hippo-related biomarkers into future biomarker-driven trials [2,75,76,77,78].

## 5. Conclusions

In conclusion, our study indicates subtype-dependent expression patterns of core Hippo pathway components in TETs rather than a uniform pattern across entities. Based on median log_2_FC values, *YAP1* and *TEAD4* transcript levels were higher in selected subtypes, notably type A and B3 thymomas and TCs. These transcript-level patterns co-occurred with lower *MST1* and *LATS1* transcript levels, particularly in TCs, whereas *SAV1* and *MOB1A* showed more nuanced, component-specific variation. Integration with IHC highlights that Hippo pathway state is influenced by post-transcriptional/post-translational regulation, and that mRNA abundance only partially mirrors protein expression and localization. Given the exploratory nature of subtype-resolved analyses in this rare tumor entity, these findings nominate *YAP1*/*TEAD4-* and kinase-related readouts as candidates for independent validation in larger, ideally multi-center cohorts, with complementary spatial and functional approaches.

## Figures and Tables

**Table 1:** Clinicopathological characteristics of the 23 patients diagnosed with thymic epithelial tumors (TETs) included in the RT-qPCR cohort. Three additional samples of non-neoplastic thymus (N1–N3) served as normal reference tissue and are not listed.

Parameter	Median	Mix–Max
Age (years)	59	36–77
Tumor Size (cm)	7.5	2.4–13
	**Number**	**%**
**Gender**		
Male	13	56.5
Female	10	43.5
**WHO Subtypes**		
Type A	3	13
Type B1	5	21.7
Type B2	5	21.7
Type B3	5	21.7
Thymic carcinoma (TC)	5	21.7
**Masaoka-Koga Stage** *		
I	5	21.7
II	9	39.1
III	2	8.7
IVa	3	13
IVb	2	8.7
Presence of Myasthenia Gravis	4	17.4
**Event**		
Alive, censored	23	100
Dead	0	0

* For two samples, the Masaoka-Koga stage was not available.

**Table 2:** Results of the gene expression analysis of core Hippo pathway components in thymic epithelial tumors (TETs). Shown are sample-wise fold changes (FC, median N1/N3) and corresponding log_2_ fold change (log_2_FC) values for *MST1*, *SAV1*, *LATS1*, *MOB1A*, *YAP1*, and *TEAD4* relative to normal thymus (N), with expression levels normalized to the housekeeping genes *HPRT1* (RTP) and *TBP*. Fold-change values < 1 were interpreted as downregulation and values > 1 as upregulation compared with the normal thymus. Accordingly, log_2_FC values < 0 indicate downregulation and values > 0 indicate upregulation. N2 was excluded due to a benign cyst (labeled in red).

	Fold Change (FC) (Median N1/N3)						Log_2_FC					
Sample (TET Subtype)	MST1	SAV1	LATS1	MOB1A	YAP1	TEAD4	MST1	SAV1	LATS1	MOB1A	YAP1	TEAD4
1 (N)	1.42	0.92	1.12	0.95	0.91	0.84	0.51	−0.13	0.16	−0.07	−0.13	−0.26
2 (N)	1.34	1.53	1.76	1.13	2.25	0.94	0.42	0.62	0.82	0.18	1.17	−0.08
3 (N)	0.70	1.09	0.89	1.05	1.10	1.20	−0.51	0.13	−0.16	0.07	0.13	0.26
4 (A)	2.66	2.52	1.09	1.68	3.98	2.17	1.41	1.34	0.13	0.75	1.99	1.12
5 (A)	0.90	14.07	2.05	1.06	10.76	5.07	−0.15	3.82	1.04	0.08	3.43	2.34
6 (A)	0.50	4.76	1.09	1.17	13.11	3.33	−1.01	2.25	0.13	0.22	3.71	1.74
7 (B1)	0.58	0.72	0.94	0.91	1.08	1.24	−0.78	−0.48	−0.09	−0.13	0.11	0.31
8 (B1)	0.64	1.54	0.56	0.78	1.84	1.28	−0.65	0.63	−0.83	−0.37	0.88	0.36
9 (B1)	0.98	1.15	1.15	0.78	1.50	0.75	−0.03	0.20	0.20	−0.36	0.59	−0.41
10 (B1)	0.48	0.80	0.52	1.15	1.13	2.13	−1.07	−0.32	−0.93	0.20	0.18	1.09
11 (B1)	0.62	0.56	0.38	0.98	0.73	0.62	−0.68	−0.82	−1.40	−0.02	−0.45	−0.69
12 (B2)	1.13	1.20	1.08	0.89	1.57	0.88	0.17	0.26	0.10	−0.16	0.65	−0.19
13 (B2)	0.64	1.60	0.75	1.04	1.67	0.27	−0.65	0.67	−0.42	0.05	0.74	−1.90
14 (B2)	0.76	1.74	0.53	1.11	2.68	1.01	−0.39	0.80	−0.91	0.15	1.42	0.01
15 (B2)	0.94	4.38	1.26	1.21	4.34	4.97	−0.09	2.13	0.33	0.28	2.12	2.31
16 (B2)	0.96	1.72	1.09	1.22	1.28	1.39	−0.06	0.78	0.13	0.29	0.35	0.48
17 (B3)	0.69	2.70	0.98	1.24	4.15	1.38	−0.53	1.44	−0.03	0.31	2.05	0.47
18 (B3)	2.10	3.96	1.50	1.47	6.86	0.96	1.07	1.99	0.59	0.56	2.78	−0.06
19 (B3)	0.76	4.03	0.58	0.96	5.61	3.84	−0.40	2.01	−0.78	−0.06	2.49	1.94
20 (B3)	1.15	7.54	1.10	2.05	12.79	5.21	0.20	2.92	0.14	1.04	3.68	2.38
21 (B3)	0.83	6.57	0.80	1.25	7.15	2.93	−0.26	2.72	−0.32	0.33	2.84	1.55
22 (TC)	0.43	3.67	0.74	0.73	3.11	4.83	−1.21	1.88	−0.43	−0.45	1.64	2.27
23 (TC)	0.07	0.57	0.14	2.30	3.23	17.48	−3.83	−0.81	−2.87	1.20	1.69	4.13
24 (TC)	0.38	0.82	0.63	0.91	1.40	3.05	−1.38	−0.28	−0.67	−0.14	0.48	1.61
25 (TC)	0.26	1.59	0.33	0.71	0.96	11.22	−1.93	0.67	−1.62	−0.49	−0.06	3.49
26 (TC)	0.52	2.17	0.39	0.96	3.19	12.19	−0.94	1.12	−1.34	−0.06	1.67	3.61

**Table 3:** Summary of subtype-specific median log_2_FC values for the core Hippo pathway components *MST1*, *SAV1*, *LATS1*, *MOB1A*, *YAP1*, and *TEAD4* across TET subtypes. Median log_2_FC values < 0 were interpreted as downregulation and values > 0 as upregulation compared with the normal thymus.

	MST1	SAV1	LATS1	MOB1A	YAP1	TEAD4
**Thymoma Type A**	−0.15	2.25	0.13	0.22	3.43	1.74
**Thymoma Type B1**	−0.68	−0.32	−0.83	−0.13	0.18	0.31
**Thymoma Type B2**	−0.09	0.78	0.10	0.15	0.74	0.01
**Thymoma Type B3**	−0.26	2.01	−0.03	0.33	2.78	1.55
**Thymus Carcinoma (TC)**	−1.38	0.67	−1.34	−0.14	1.64	3.49

## Data Availability

The data in this work can be obtained upon request from the corresponding author.

## Supplementary Materials

The following supporting information can be downloaded at: https://www.mdpi.com/article/10.3390/biomedicines14020305/s1, Table S1: Sample input concentrations and spectrophotometric purity (A260/280 ratios); Table S2: Reagent lot comparison; Table S3: Inter-run variability; Table S4: Cross-instrument comparison; Table S5: Calculation of internal controls (ICs); Table S6: Primer assay oligonucleotide sequences; Figure S1: Melt curves of the primer assays; Table S7: Primer efficiency assessment; Table S8: Replicate-level QC and exclusion criteria of excluded housekeeping gene (HKG) samples excluded in analysis; Table S9: Housekeeping gene (HKG) Cq datasets used for RefFinder stability analysis; Table S10: Details of the HKG stability statistics from RefFinder; Table S11: Calculation sheets for relative gene expression block 1; Table S12: Calculation sheets for relative gene expression block 2; Table S13: Raw Cq data matrices; Table S14: Replicate-level quality control (QC) metrics and exclusion criteria of samples included in analysis; Table S15: IHC antibodies, retrieval, controls, and scoring; Table S16: Relative gene expression in relation to IHC results on a per-sample basis; Figure S2: IHC subcellular localization; Figure S3: The Cancer Genome Atlas Thymoma (TCGA-THYM) RNA-sequencing–based mRNA expression of upstream Hippo pathway components.

## Abbreviations

The following abbreviations are used in this manuscript:

PMU	Paracelsus Medical University
TET(s)	Thymic Epithelial Tumor(s)
WHO	World Health Organization
FC(s)	Fold change(s)
TC	Thymic Carcinoma
YAP1	Yes-associated protein 1
TAZ	Transcriptional co-activator with PDZ-binding motif
WWTR1	WW domain-containing transcription regulator 1
TEAD1–4	TEA domain transcription factor 1–4
IHC	Immunohistochemistry/immunohistochemical
MST1/2	Mammalian STE20-like kinases 1/2
SAV1	Salvador homolog 1
LATS1/2	Large tumor suppressor kinases 1/2
MOB1(A/B)	Mps one binder 1(A/B)
mRNA	Messenger RNA
IRB	Institutional review board
gDNA	Genomic DNA
RIN	RNA integrity number
IC	Internal control
RT	Reverse transcription
TBP	TATA-box binding protein
HPRT1	Hypoxanthine-guanine phosphoribosyltransferase 1
RTP	RealTimePrimers.com
Cq	Quantification Cycle
Log_2_FC	Log_2_-transformed fold changes
N	Normal (thymus) sample
NTC	No-template controls
NRT	No-reverse transcription control
QC	Quality control
PPIA	Peptidyl-prolyl isomerase A
IDTTM	Integrated DNA Technologies
cDNA	Complementary DNA
MIQE	Minimum Information for Publication of Quantitative Real-Time PCR Experiments
HKG	Housekeeping gene
EMT	Epithelial–mesenchymal transition
AYAP	Active YAP1
TCGA (-THYM)	The Cancer Genome Atlas Program (Thymoma)
GTF2I	General Transcription Factor II-I

## References

[1] Imbimbo M., Salfi G., Borgeaud M., Ottaviano M., Froesch P., Bouchaab H., Cafarotti S., Addeo A. (2025). Thymic epithelial tumors: What’s new and what’s next?. ESMO Rare Cancers.

[2] Elm L., Gerlitz N., Hochholzer A., Papadopoulos T., Levidou G. (2025). Hippo Pathway Dysregulation in Thymic Epithelial Tumors (TETs): Associations with Clinicopathological Features and Patients’ Prognosis. Int. J. Mol. Sci..

[3] von der Thüsen J. (2024). Thymic epithelial tumours: Histopathological classification and differential diagnosis. Histopathology.

[4] Barron D.A., Kagey J.D. (2014). The role of the Hippo pathway in human disease and tumorigenesis. Clin. Transl. Med..

[5] Lo Sardo F., Strano S., Blandino G. (2018). YAP and TAZ in Lung Cancer: Oncogenic Role and Clinical Targeting. Cancers.

[6] Pham T.H., Pahuja K.B., Hagenbeek T.J., Zbieg J., Noland C.L., Pham V.C., Yao X., Rose C.M., Browder K.C., Lee H.-J. (2024). Targeting the Hippo Pathway in Cancers via Ubiquitination Dependent TEAD Degradation.

[7] Sanchez-Vega F., Mina M., Armenia J., Chatila W.K., Luna A., La K.C., Dimitriadoy S., Liu D.L., Kantheti H.S., Saghafinia S. (2018). Oncogenic Signaling Pathways in The Cancer Genome Atlas. Cell.

[8] Palamaris K., Levidou G., Kordali K., Masaoutis C., Rontogianni D., Theocharis S. (2023). Searching for Novel Biomarkers in Thymic Epithelial Tumors: Immunohistochemical Evaluation of Hippo Pathway Components in a Cohort of Thymic Epithelial Tumors. Biomedicines.

[9] QIAGEN (2024). QuantiNova SYBR Green RT-PCR Handbook.

[10] Bustin S.A., Benes V., Garson J.A., Hellemans J., Huggett J., Kubista M., Mueller R., Nolan T., Pfaffl M.W., Shipley G.L. (2009). The MIQE Guidelines: Minimum Information for Publication of Quantitative Real-Time PCR Experiments. Clin. Chem..

[11] Dietrich D., Uhl B., Sailer V., Holmes E.E., Jung M., Meller S., Kristiansen G. (2013). Improved PCR Performance Using Template DNA from Formalin-Fixed and Paraffin-Embedded Tissues by Overcoming PCR Inhibition. PLoS ONE.

[12] Ruijter J.M., Pfaffl M.W., Zhao S., Spiess A.N., Boggy G., Blom J., Rutledge R.G., Sisti D., Lievens A., De Preter K. (2013). Evaluation of qPCR curve analysis methods for reliable biomarker discovery: Bias, resolution, precision, and implications. Methods.

[13] Suslov O., Steindler D.A. (2005). PCR inhibition by reverse transcriptase leads to an overestimation of amplification efficiency. Nucleic Acids Res..

[14] Svec D., Tichopad A., Novosadova V., Pfaffl M.W., Kubista M. (2015). How good is a PCR efficiency estimate: Recommendations for precise and robust qPCR efficiency assessments. Biomol. Detect. Quantif..

[15] Borowska D., Rothwell L., Bailey R.A., Watson K., Kaiser P. (2016). Identification of stable reference genes for quantitative PCR in cells derived from chicken lymphoid organs. Vet. Immunol. Immunopathol..

[16] Rácz G.A., Nagy N., Gál Z., Pintér T., Hiripi L., Vértessy B.G. (2019). Evaluation of critical design parameters for RT-qPCR-based analysis of multiple dUTPase isoform genes in mice. FEBS Open Bio.

[17] Medrano G., Guan P., Barlow-Anacker A.J., Gosain A. (2017). Comprehensive selection of reference genes for quantitative RT-PCR analysis of murine extramedullary hematopoiesis during development. PLoS ONE.

[18] Xie F., Xiao P., Chen D., Xu L., Zhang B. (2012). miRDeepFinder: A miRNA analysis tool for deep sequencing of plant small RNAs. Plant Mol. Biol..

[19] Bustin S.A., Ruijter J.M., van den Hoff M.J.B., Kubista M., Pfaffl M.W., Shipley G.L., Tran N., Rödiger S., Untergasser A., Mueller R. (2025). MIQE 2.0: Revision of the Minimum Information for Publication of Quantitative Real-Time PCR Experiments Guidelines. Clin. Chem..

[20] The cBioPortal for Cancer Genomics. Thymoma (TCGA, Firehose Legacy) (study ID: thym_tcga). https://www.cbioportal.org/study?id=thym_tcga.

[21] Kuhn E., Pescia C., Mendogni P., Nosotti M., Ferrero S. (2023). Thymic Epithelial Tumors: An Evolving Field. Life.

[22] Müller D., Loskutov J., Küffer S., Marx A., Regenbrecht C.R.A., Ströbel P., Regenbrecht M.J. (2024). Cell Culture Models for Translational Research on Thymomas and Thymic Carcinomas: Current Status and Future Perspectives. Cancers.

[23] Fu M., Hu Y., Lan T., Guan K.-L., Luo T., Luo M. (2022). The Hippo signalling pathway and its implications in human health and diseases. Signal Transduct. Target. Ther..

[24] Wang Y., Xu X., Maglic D., Dill M.T., Mojumdar K., Ng P.K.-S., Jeong K.J., Tsang Y.H., Moreno D., Bhavana V.H. (2018). Comprehensive Molecular Characterization of the Hippo Signaling Pathway in Cancer. Cell Rep..

[25] Yu F.X., Zhao B., Guan K.L. (2015). Hippo Pathway in Organ Size Control, Tissue Homeostasis, and Cancer. Cell.

[26] Zhao B., Li L., Lei Q., Guan K.L. (2010). The Hippo-YAP pathway in organ size control and tumorigenesis: An updated version. Genes Dev..

[27] Zhong Z., Jiao Z., Yu F.-X. (2024). The Hippo signaling pathway in development and regeneration. Cell Rep..

[28] Elm L., Levidou G. (2024). The Molecular Landscape of Thymic Epithelial Tumors: A Comprehensive Review. Int. J. Mol. Sci..

[29] Lee H.-S., Jang H.-J., Shah R., Yoon D., Hamaji M., Wald O., Lee J.-S., Sugarbaker D.J., Burt B.M. (2017). Genomic Analysis of Thymic Epithelial Tumors Identifies Novel Subtypes Associated with Distinct Clinical Features. Clin. Cancer Res..

[30] Möhrmann L., Rostock L., Werner M., Oleś M., Arnold J.S., Paramasivam N., Jöhrens K., Rupp L., Schmitz M., Richter D. (2025). Genomic landscape and molecularly informed therapy in thymic carcinoma and other advanced thymic epithelial tumors. Med.

[31] Radovich M., Pickering C.R., Felau I., Ha G., Zhang H., Jo H., Hoadley K.A., Anur P., Zhang J., McLellan M. (2018). The Integrated Genomic Landscape of Thymic Epithelial Tumors. Cancer Cell.

[32] Schmauch B., Cabeli V., Domingues O.D., Le Douget J.E., Hardy A., Belbahri R., Maussion C., Romagnoni A., Eckstein M., Fuchs F. (2025). Deep learning uncovers histological patterns of YAP1/TEAD activity related to disease aggressiveness in cancer patients. iScience.

[33] Liu M., Song Y., Kang Y., Xue N., Zhao J., Jin Y., Liu C., Wang B. (2025). Integrative analysis identifies TEAD4 as a universal prognostic biomarker in human cancers. Front. Immunol..

[34] Chen M., Huang B., Zhu L., Chen K., Liu M., Zhong C. (2020). Structural and Functional Overview of TEAD4 in Cancer Biology. Onco. Targets Ther..

[35] Gong X., Li N., Sun C., Li Z., Xie H. (2022). A Four-Gene Prognostic Signature Based on the TEAD4 Differential Expression Predicts Overall Survival and Immune Microenvironment Estimation in Lung Adenocarcinoma. Front. Pharmacol..

[36] Hsu S.-C., Lin C.-Y., Lin Y.-Y., Collins C.C., Chen C.-L., Kung H.-J. (2022). TEAD4 as an Oncogene and a Mitochondrial Modulator. Front. Cell Dev. Biol..

[37] Liu J.-Y., Li Y.-H., Lin H.-X., Liao Y.-J., Mai S.-J., Liu Z.-W., Zhang Z.-L., Jiang L.-J., Zhang J.-X., Kung H.-F. (2013). Overexpression of YAP 1 contributes to progressive features and poor prognosis of human urothelial carcinoma of the bladder. BMC Cancer.

[38] Park J.H., Shin J.E., Park H.W. (2018). The Role of Hippo Pathway in Cancer Stem Cell Biology. Mol. Cells.

[39] Sun Z., Xu R., Li X., Ren W., Ou C., Wang Q., Zhang H., Zhang X., Ma J., Wang H. (2015). Prognostic Value of Yes-Associated Protein 1 (YAP1) in Various Cancers: A Meta-Analysis. PLoS ONE.

[40] Xia Y., Chang T., Wang Y., Liu Y., Li W., Li M., Fan H.-Y. (2014). YAP Promotes Ovarian Cancer Cell Tumorigenesis and Is Indicative of a Poor Prognosis for Ovarian Cancer Patients. PLoS ONE.

[41] Zhang W., Li J., Wu Y., Ge H., Song Y., Wang D., Yuan H., Jiang H., Wang Y., Cheng J. (2018). TEAD4 overexpression promotes epithelial-mesenchymal transition and associates with aggressiveness and adverse prognosis in head neck squamous cell carcinoma. Cancer Cell Int..

[42] Hu X., Zhang Y., Yu H., Zhao Y., Sun X., Li Q., Wang Y. (2022). The role of YAP1 in survival prediction, immune modulation, and drug response: A pan-cancer perspective. Front. Immunol..

[43] Luo J., Zou H., Guo Y., Tong T., Chen Y., Xiao Y., Pan Y., Li P. (2023). The oncogenic roles and clinical implications of YAP/TAZ in breast cancer. Br. J. Cancer.

[44] Zanconato F., Cordenonsi M., Piccolo S. (2016). YAP/TAZ at the Roots of Cancer. Cancer Cell.

[45] Bae J.S., Kim S.M., Lee H. (2017). The Hippo signaling pathway provides novel anti-cancer drug targets. Oncotarget.

[46] Furth N., Aylon Y. (2017). The LATS1 and LATS2 tumor suppressors: Beyond the Hippo pathway. Cell Death Differ..

[47] Meng Z., Moroishi T., Guan K.L. (2016). Mechanisms of Hippo pathway regulation. Genes Dev..

[48] Bae S.J., Ni L., Osinski A., Tomchick D.R., Brautigam C.A., Luo X. (2017). SAV1 promotes Hippo kinase activation through antagonizing the PP2A phosphatase STRIPAK. Elife.

[49] Lin Z., Xie R., Guan K., Zhang M. (2020). A WW Tandem-Mediated Dimerization Mode of SAV1 Essential for Hippo Signaling. Cell Rep..

[50] Zhang H., Yang Z., Nakamura F. (2023). Importance of the filamin A-Sav1 interaction in organ size control: Evidence from transgenic mice. Int. J. Dev. Biol..

[51] Ni L., Zheng Y., Hara M., Pan D., Luo X. (2015). Structural basis for Mob1-dependent activation of the core Mst-Lats kinase cascade in Hippo signaling. Genes Dev..

[52] Han H., Wang W. (2023). A tale of two Hippo pathway modules. EMBO J..

[53] Hartmann K., Schlombs K., Laible M., Gürtler C., Schmidt M., Sahin U., Lehr H.-A. (2018). Robustness of biomarker determination in breast cancer by RT-qPCR: Impact of tumor cell content, DCIS and non-neoplastic breast tissue. Diagn. Pathol..

[54] Xin Z., Lin M., Hao Z., Chen D., Chen Y., Chen X., Xu X., Li J., Wu D., Chai Y. (2022). The immune landscape of human thymic epithelial tumors. Nat. Commun..

[55] Liu X., Wang C., Huang Y., Lv Q., Yu C., Ying J., Duan L., Guo Y., Huang G., Shen W. (2024). Abnormal Cellular Populations Shape Thymic Epithelial Tumor Heterogeneity and Anti-Tumor by Blocking Metabolic Interactions in Organoids. Adv. Sci..

[56] Nabel C.S., Ackman J.B., Hung Y.P., Louissaint A., Riely G.J. (2024). Single-Cell Sequencing Illuminates Thymic Development: An Updated Framework for Understanding Thymic Epithelial Tumors. Oncologist.

[57] Liu Y., Deng J. (2019). Ubiquitination-deubiquitination in the Hippo signaling pathway (Review). Oncol. Rep..

[58] Nguyen T.H., Kugler J.M. (2018). Ubiquitin-Dependent Regulation of the Mammalian Hippo Pathway: Therapeutic Implications for Cancer. Cancers.

[59] Peng S., Li C., He Y., Xue L., Guo X. (2025). Regulatory roles of RNA binding proteins in the Hippo pathway. Cell Death Discov..

[60] Zhao B., Li L., Tumaneng K., Wang C.Y., Guan K.L. (2010). A coordinated phosphorylation by Lats and CK1 regulates YAP stability through SCF(beta-TRCP). Genes Dev..

[61] Gumbiner B.M., Kim N.G. (2014). The Hippo-YAP signaling pathway and contact inhibition of growth. J. Cell. Sci..

[62] Moroishi T., Park H.W., Qin B., Chen Q., Meng Z., Plouffe S.W., Taniguchi K., Yu F.X., Karin M., Pan D. (2015). A YAP/TAZ-induced feedback mechanism regulates Hippo pathway homeostasis. Genes Dev..

[63] Vogel C., Marcotte E.M. (2012). Insights into the regulation of protein abundance from proteomic and transcriptomic analyses. Nat. Rev. Genet..

[64] Liu Y., Beyer A., Aebersold R. (2016). On the Dependency of Cellular Protein Levels on mRNA Abundance. Cell.

[65] Cunningham R., Hansen C.G. (2022). The Hippo pathway in cancer: YAP/TAZ and TEAD as therapeutic targets in cancer. Clin. Sci..

[66] Yang J., Zhang B., Guan W., Fan Z., Pu X., Zhao L., Jiang W., Cai W., Quan X., Miao S. (2023). Molecular genetic characteristics of thymic epithelial tumors with distinct histological subtypes. Cancer Med..

[67] Takata S. (2024). Genomic insights into molecular profiling of thymic carcinoma: A narrative review. Mediastinum.

[68] Zhang X., Zhang P., Cong A., Feng Y., Chi H., Xia Z., Tang H. (2023). Unraveling molecular networks in thymic epithelial tumors: Deciphering the unique signatures. Front. Immunol..

[69] Yang D., Zhang N., Li M., Hong T., Meng W., Ouyang T. (2021). The Hippo Signaling Pathway: The Trader of Tumor Microenvironment. Front. Oncol..

[70] Mokhtari R.B., Ashayeri N., Baghaie L., Sambi M., Satari K., Baluch N., Bosykh D.A., Szewczuk M.R., Chakraborty S. (2023). The Hippo Pathway Effectors YAP/TAZ-TEAD Oncoproteins as Emerging Therapeutic Targets in the Tumor Microenvironment. Cancers.

[71] Ghaboura N. (2025). Unraveling the Hippo pathway: YAP/TAZ as central players in cancer metastasis and drug resistance. EXCLI J..

[72] von Ahlfen S., Missel A., Bendrat K., Schlumpberger M. (2007). Determinants of RNA quality from FFPE samples. PLoS ONE.

[73] Cronin M., Pho M., Dutta D., Stephans J.C., Shak S., Kiefer M.C., Esteban J.M., Baker J.B. (2004). Measurement of gene expression in archival paraffin-embedded tissues: Development and performance of a 92-gene reverse transcriptase-polymerase chain reaction assay. Am. J. Pathol..

[74] Aggerholm-Pedersen N., Safwat A., Bærentzen S., Nordsmark M., Nielsen O.S., Alsner J., Sørensen B.S. (2014). The importance of reference gene analysis of formalin-fixed, paraffin-embedded samples from sarcoma patients—An often underestimated problem. Transl. Oncol..

[75] Hagenbeek T.J., Zbieg J.R., Hafner M., Mroue R., Lacap J.A., Sodir N.M., Noland C.L., Afghani S., Kishore A., Bhat K.P. (2023). An allosteric pan-TEAD inhibitor blocks oncogenic YAP/TAZ signaling and overcomes KRAS G12C inhibitor resistance. Nat. Cancer.

[76] Chapeau E.A., Sansregret L., Galli G.G., Chène P., Wartmann M., Mourikis T.P., Jaaks P., Baltschukat S., Barbosa I.A.M., Bauer D. (2024). Direct and selective pharmacological disruption of the YAP-TEAD interface by IAG933 inhibits Hippo-dependent and RAS-MAPK-altered cancers. Nat. Cancer.

[77] Pobbati A.V., Kumar R., Rubin B.P., Hong W. (2023). Therapeutic targeting of TEAD transcription factors in cancer. Trends Biochem. Sci..

[78] Lao Z., Chen X., Pan B., Fang B., Yang W., Qian Y. (2025). Pharmacological regulators of Hippo pathway: Advances and challenges of drug development. FASEB J..

