# Supplementary material for: Subtype-Dependent Expression Patterns of Core Hippo Pathway Components in Thymic Epithelial Tumors (TETs): An RT-qPCR Study
Source: Biomedicines. 2026 Jan 29;14(2):305. doi: 10.3390/biomedicines14020305 (PMC12937678; doi:10.3390/biomedicines14020305)
Supplement: Supplementary file 1 [file biomedicines-14-00305-s001.zip › Figure S1 Melt curves of the primer assays.pdf]

Figure S1. Melt curves of the primer assays. (A-G) Melt curves of the included primer assays in the final analysis with their No-template control (NTC) and No-Reverse-Transcription control (NRT): A. *MST1*/*STK4* (sample 20); B. *SAV1* (sample 20); C. *LATS1* (sample 20); D. *MOB1A* (sample 20); E. *YAP1* (sample 20); F. *TEAD4* (sample 1 and 2); G. *TBP* (sample 1 and 2); H. *HPRT1* (RealTimePrimers.com (RTP); sample 1 and 2); (I-N) Melt curves of the excluded assays: I. *HPRT1* (Integrated DNA Technologies (IDT); sample 3 and 23); J. *PPIA* (sample 3 and 23); K. *LATS1* (sample 3 and 23); L. *SAV1* (sample 3 and 23); M. *TAZ*/*WWTR1* (sample 3 and 23); N. *TAZ*/*WWTR1* (sample 3 and 23).

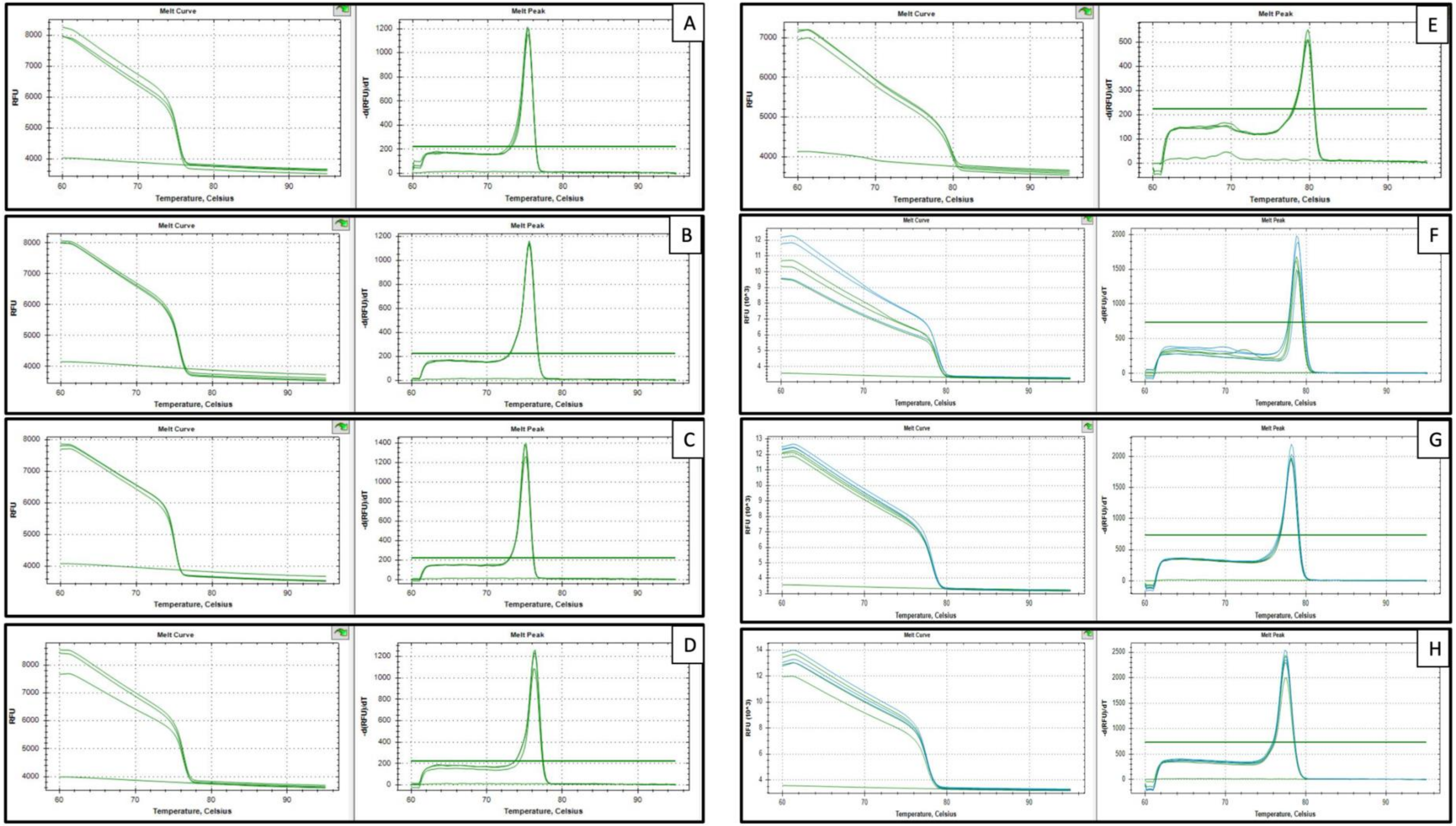

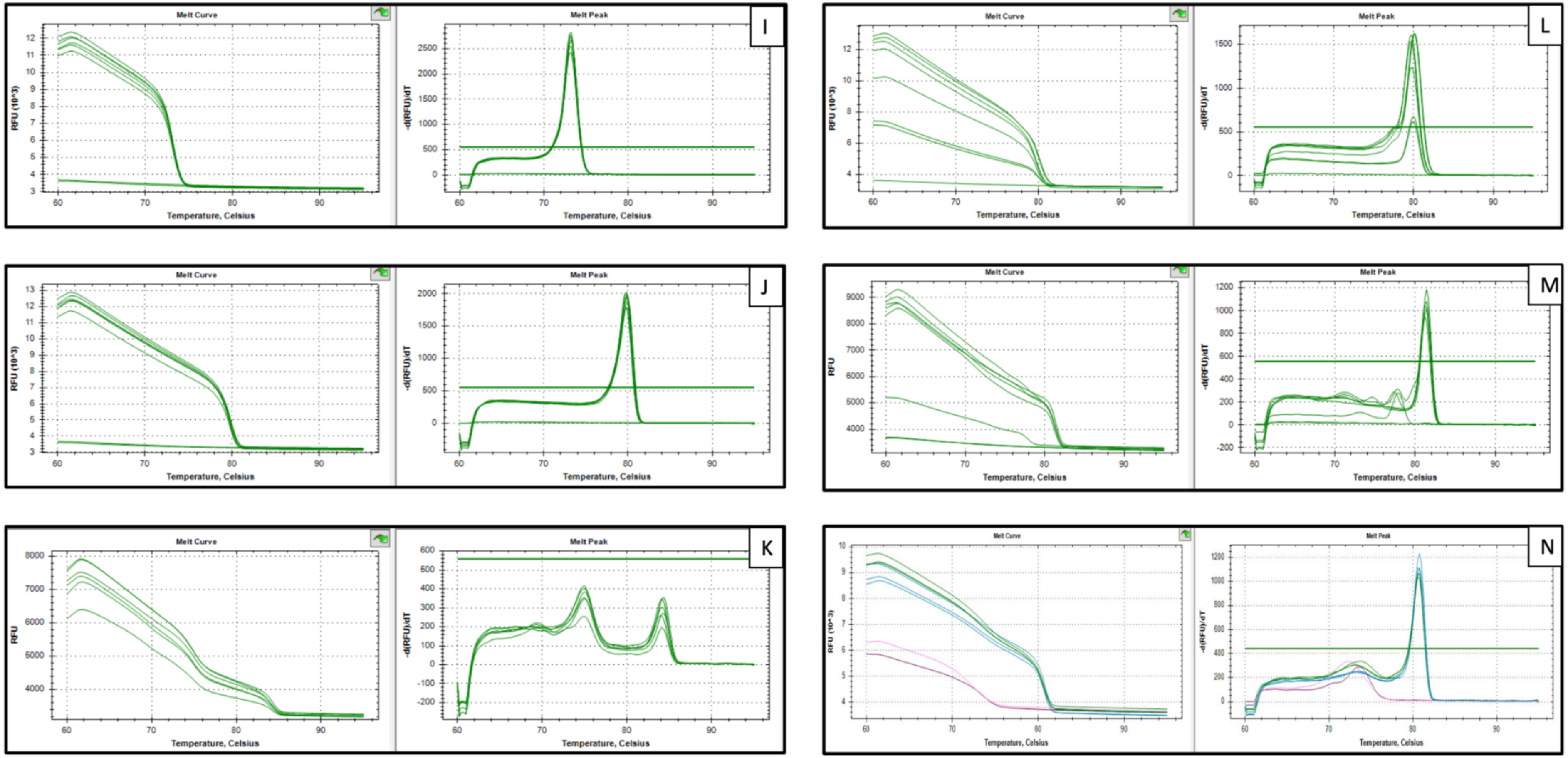
I
J
K
L
M
N
Melt Curve
Melt Peak
RFU (10^3)
RFU
-d(RFU)/dT
Temperature, Celsius
