# Supplementary material for: Subtype-Dependent Expression Patterns of Core Hippo Pathway Components in Thymic Epithelial Tumors (TETs): An RT-qPCR Study
Source: Biomedicines. 2026 Jan 29;14(2):305. doi: 10.3390/biomedicines14020305 (PMC12937678; doi:10.3390/biomedicines14020305)

**Figure S2.** Summary of subcellular localization patterns based on IHC scoring. Tumor samples (n = 23) were categorized as cytoplasmic only (cytoplasmic positivity > 0% and nuclear positivity = 0%), nuclear only (nuclear > 0% and cytoplasmic = 0%), or mixed (both compartments > 0%). YAP1 and AYAP showed nuclear-only versus mixed localization patterns, whereas TEAD4 showed cytoplasmic-only versus mixed patterns. MST1, SAV1, LATS1 and MOB1A were exclusively cytoplasmic across tumor samples (Table S16).

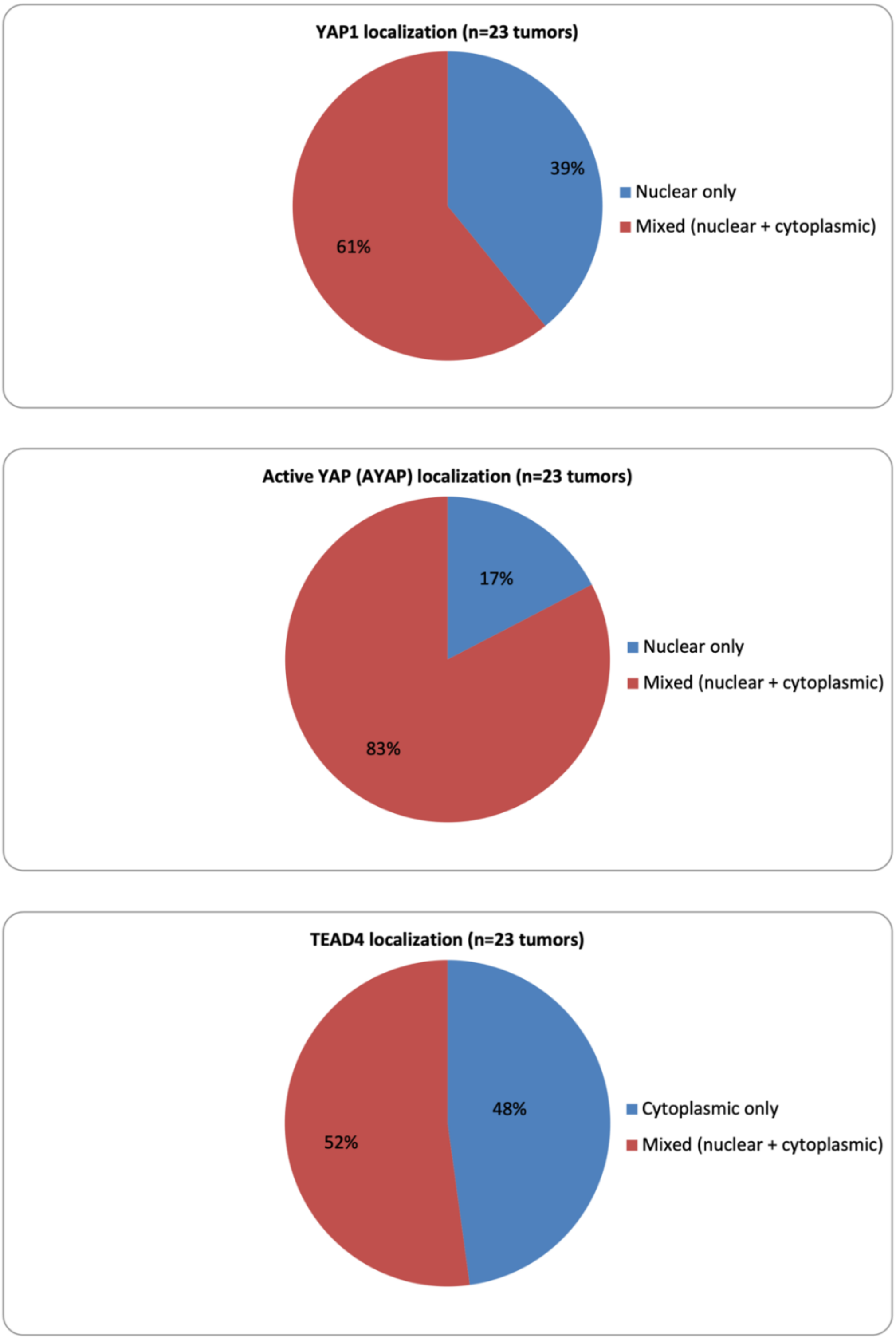

Supplement: Supplementary file 1 [file biomedicines-14-00305-s001.zip › Figure S2 Summary of subcellular localization patterns based on IHC scoring.pdf]
