# Supplementary material for: Subtype-Dependent Expression Patterns of Core Hippo Pathway Components in Thymic Epithelial Tumors (TETs): An RT-qPCR Study
Source: Biomedicines. 2026 Jan 29;14(2):305. doi: 10.3390/biomedicines14020305 (PMC12937678; doi:10.3390/biomedicines14020305)
Supplement: Supplementary file 1 [file biomedicines-14-00305-s001.zip › Figure S3 The Cancer Genome Atlas Thymoma (TCGA-THYM) RNA-sequencing expression of upstream Hippo pathway components.pdf]

Figure S3. The Cancer Genome Atlas Thymoma (TCGA-THYM) RNA-sequencing expression of upstream Hippo pathway components. Boxplots show *MST1* (A), *SAV1* (B), *LATS1* (C), and *MOB1A* (D) expression across TCGA-THYM histological subtypes. Data are derived from the TCGA-THYM cohort and represent RNA-sequencing–based, cohort-normalized expression values (RNA Seq V2 RSEM (log2(value+1)). Points denote individual tumor samples (n= 124 samples/patients). The displayed genes correspond to those assessed by RT-qPCR in this study. Data were retrieved via cBioPortal for Cancer Genomics (study: Thymoma (TCGA, Firehose Legacy), accessed 20 January 2026).

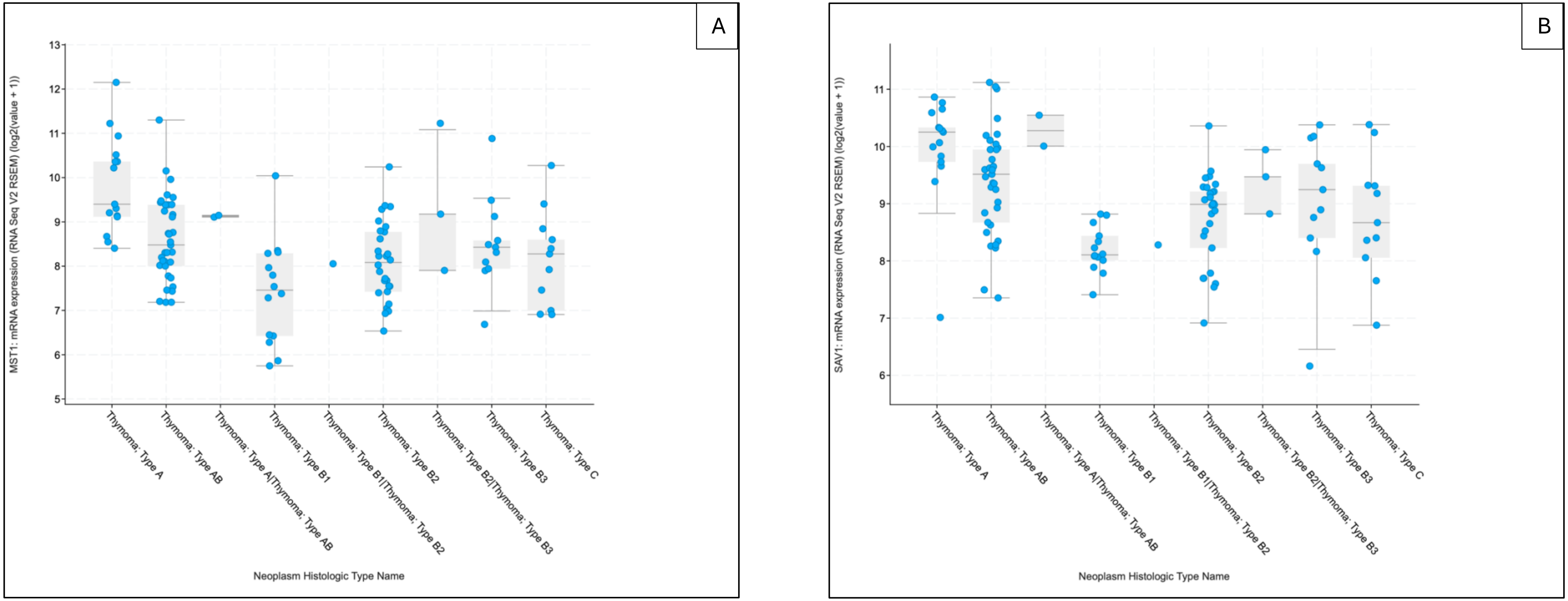

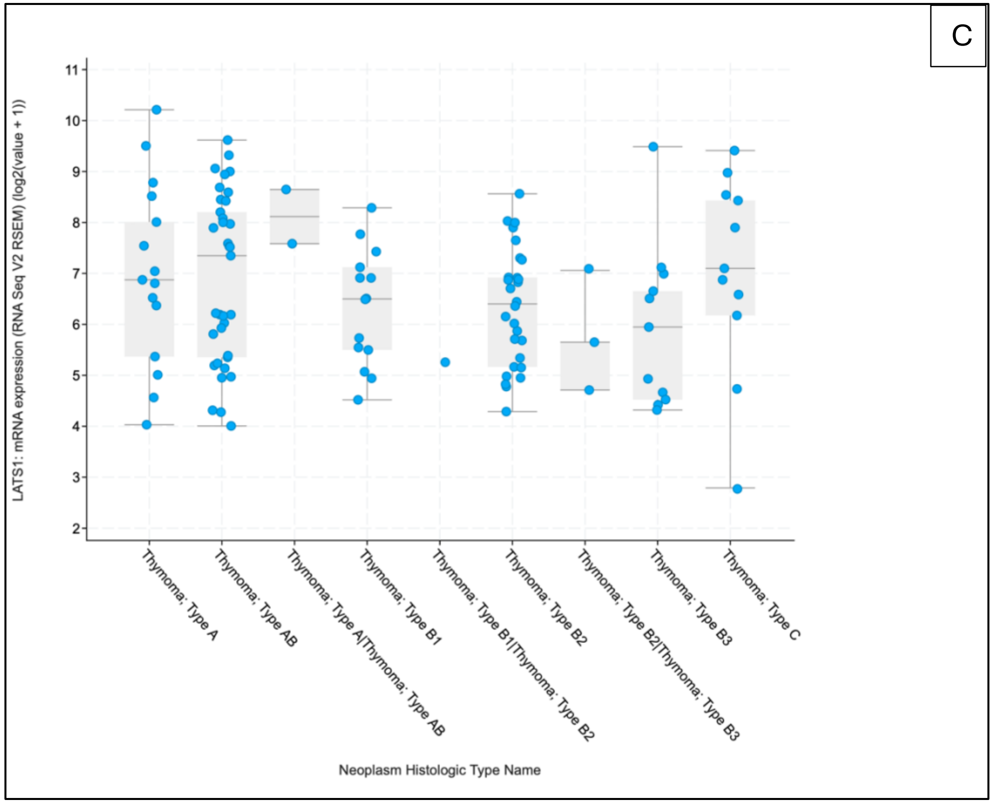
C
LATS1: mRNA expression (RNA Seq V2 RSEM) (log2(value + 1))
Thymoma; Type A
Thymoma; Type AB
Thymoma; Type A|Thymoma; Type AB
Thymoma; Type B1
Thymoma; Type B1|Thymoma; Type B2
Thymoma; Type B2
Thymoma; Type B2|Thymoma; Type B3
Thymoma; Type B3
Thymoma; Type C
Neoplasm Histologic Type Name

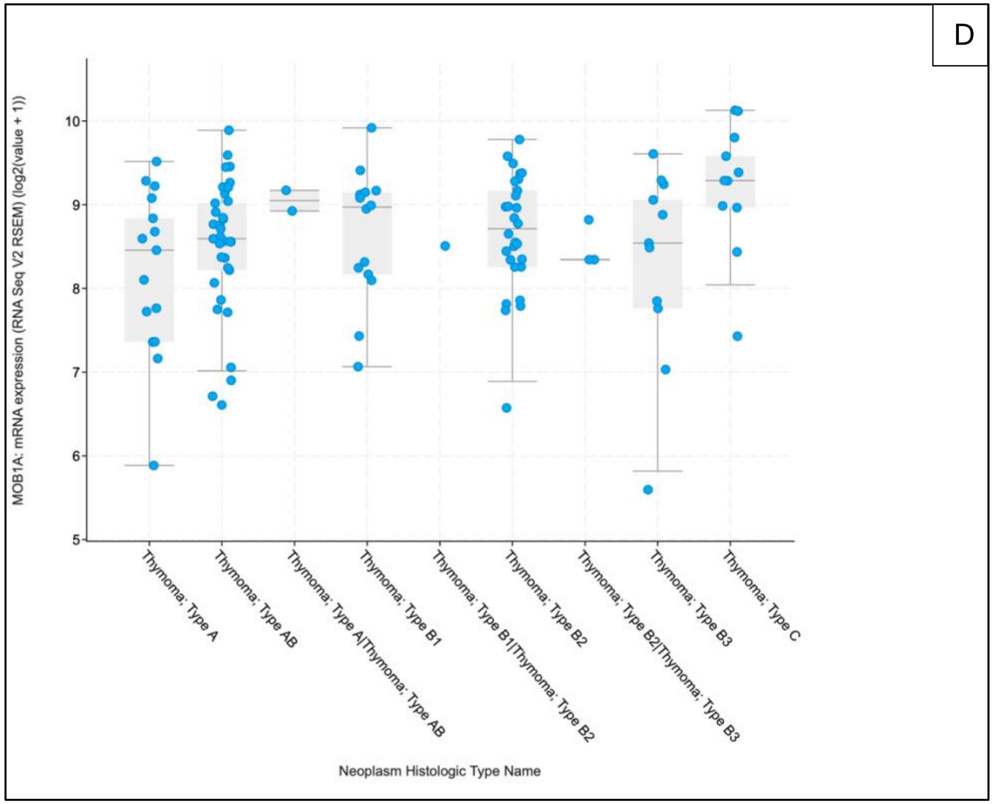
D
MOB1A: mRNA expression (RNA Seq V2 RSEM) (log2(value + 1))
Thymoma; Type A
Thymoma; Type AB
Thymoma; Type A|Thymoma; Type AB
Thymoma; Type B1
Thymoma; Type B1|Thymoma; Type B2
Thymoma; Type B2
Thymoma; Type B2|Thymoma; Type B3
Thymoma; Type B3
Thymoma; Type C
Neoplasm Histologic Type Name
