# Supplementary material for: Subtype-Dependent Expression Patterns of Core Hippo Pathway Components in Thymic Epithelial Tumors (TETs): An RT-qPCR Study
Source: Biomedicines. 2026 Jan 29;14(2):305. doi: 10.3390/biomedicines14020305 (PMC12937678; doi:10.3390/biomedicines14020305)
Supplement: Supplementary file 1 [file biomedicines-14-00305-s001.zip › Table S1 Sample concentrations and A260_280 ratios.pdf]

Table S1. Sample input concentrations and spectrophotometric purity (A260/280 ratios). RNA purity (A260/280) was high across all extracts (1.81–2.02).

Thymoma type	Sample	Concentration RNA Qubit	Concentration DNA Qubit after DNAse	Q-Wert A260/280
NG	1	108	17,3	2,02
NG	2	22,2	1,14	1,83
NG	3	60	8,8	1,95
A	4	102	21	2
A	5	190	44,6	1,9
A	6	84,4	9,66	1,88
B1	7	21	2,46	1,9
B1	8	40,6	36	1,9
B1	9	74,2	49,8	1,99
B1	10	256	70	1,93
B1	11	*54*	17	1,94
B2	12	34	48	2,02
B2	13	33	13,5	1,84
B2	14	34	2,03	1,81
B2	15	266	43	1,91
B2	16	140	36,8	1,92
B3	17	23,6	1,92	1,94
B3	18	37	2,98	2
B3	19	46	3,88	1,93
B3	20	362	54,6	1,93
B3	21	90,8	11,4	1,92
TC	22	33,4	2,92	1,91
TC	23	*150*	32,2	1,94
TC	24	138	23,4	1,93
TC	25	188	34,4	1,96

TC	26	*21*	1,05	1,92
