# Supplementary material for: Subtype-Dependent Expression Patterns of Core Hippo Pathway Components in Thymic Epithelial Tumors (TETs): An RT-qPCR Study
Source: Biomedicines. 2026 Jan 29;14(2):305. doi: 10.3390/biomedicines14020305 (PMC12937678; doi:10.3390/biomedicines14020305)
Supplement: Supplementary file 1 [file biomedicines-14-00305-s001.zip › Table S10 Details of the HKG stability statistics from RefFinder.pdf]

Table S10. Details of the HKG stability statistics from RefFinder. This table summarizes the RefFinder-based evaluation of four candidate housekeeping genes (*TBP*, *HPRT1* (RealTimePrimers.com [RTP], *PPIA*, *HPRT1* (Integrated DNA Technologies [IDT]) using the ΔCt method, BestKeeper, NormFinder and geNorm, with lower geometric mean ranking values indicating higher stability. Across all four algorithms, *TBP* and *HPRT1* (RTP) consistently ranked as the most stable genes (geomean rankings 1.19 and 1.41), followed by *PPIA* and *HPRT1* (IDT) (3.00 and 4.00). BestKeeper analysis of Cq data (n = 26) showed SD ≤1.3 Cq and CV ≈4% for *TBP* and *HPRT1* (RTP), which lies within commonly accepted thresholds for stable reference genes (approximately SD ≤1.5 Cq and CV <5%), whereas *PPIA* and particularly *HPRT1* (IDT) displayed higher variability (SD ≥1.5 Cq, CV ≥5.5%). High Pearson correlation coefficients between *TBP*, *HPRT1* (RTP) and *PPIA* and the BestKeeper index (r ≥0.88, p = 0.001) further support their suitability, and the comprehensive RefFinder ranking therefore identifies *TBP* and *HPRT1* (RTP) as the preferred reference genes.

Ranking Order (Better--Good--Average)

<table>
<tr><td>Method</td><td>1</td><td>2</td><td>3</td><td>4</td></tr>
<tr><td>Delta CT</td><td>TBP</td><td>HPRT1RTP</td><td>PPIA</td><td>HPRT1IDT</td></tr>
<tr><td>BestKeeper</td><td>HPRT1RTP</td><td>TBP</td><td>PPIA</td><td>HPRT1IDT</td></tr>
<tr><td>Normfinder</td><td>TBP</td><td>HPRT1RTP</td><td>PPIA</td><td>HPRT1IDT</td></tr>
<tr><td>Genorm</td><td>TBP | HPRT1RTP</td><td></td><td>PPIA</td><td>HPRT1IDT</td></tr>
<tr><td>Recommended comprehensive ranking</td><td>TBP</td><td>HPRT1RTP</td><td>PPIA</td><td>HPRT1IDT</td></tr>
</table>

Genes	Geomean of ranking values
TBP	1.19
HPRT1RTP	1.41
PPIA	3.00
HPRT1IDT	4.00

Genes	Average of STDEV
TBP	1.32
HPRT1RTP	1.34
PPIA	1.54
HPRT1IDT	1.87

CP data of housekeeping Genes by BEST KEEPER

	TBP	HPRT1RTP	HPRT1IDT	PPIA
n	26	26	26	26
geo Mean [CP]	32.05	29.42	30.97	26.95
AR Mean [CP]	32.09	29.45	31.05	27.03
min [CP]	28.45	26.58	27.01	23.02
max [CP]	35.10	31.88	37.74	33.53
std dev [+/- CP]	1.27	1.17	1.74	1.50
CV [% CP]	3.94	3.97	5.61	5.57
min [x-fold]	-12.09	-7.15	-15.58	-15.26
max [x-fold]	8.31	5.51	108.99	95.54
std dev [+/- x-fold]	2.40	2.25	3.34	2.84

Pearson correlation coefficient (r) by BEST KEEPER

	TBP	HPRT1RTP	HPRT1IDT	PPIA
HPRT1RTP	0.769	-	-	-
p-value	0.001	-	-	-
HPRT1IDT	0.672	0.661	-	-
p-value	0.001	0.001	-	-
PPIA	0.826	0.823	0.499	-
p-value	0.001	0.001	0.009	-

Pearson correlation coefficient (r)

BestKeeper vs.	TBP	HPRT1RTP	HPRT1IDT	PPIA
coeff. of corr. [r]	0.920	0.912	0.814	0.888
p-value	0.001	0.001	0.001	0.001

Gene name	Stability value
TBP	0.514
HPRT1RTP	0.592
PPIA	1.193
HPRT1IDT	1.693

Gene name	Stability value
TBP丨HPRT1RTP	1.073
PPIA	1.163
HPRT1IDT	1.516
