# Supplementary material for: Subtype-Dependent Expression Patterns of Core Hippo Pathway Components in Thymic Epithelial Tumors (TETs): An RT-qPCR Study
Source: Biomedicines. 2026 Jan 29;14(2):305. doi: 10.3390/biomedicines14020305 (PMC12937678; doi:10.3390/biomedicines14020305)
Supplement: Supplementary file 1 [file biomedicines-14-00305-s001.zip › Table S11 Calculation sheets for relative gene expression block 1.pdf]

Table S11. Calculation sheets for relative gene expression block 1. This table summarizes the stepwise calculations used to derive relative gene expression levels for block 1. For each sample, the mean Cq values of the target genes (*YAP1*, *MST1*, *MOB1A*, *TEAD4*) and the housekeeping genes (*TBP*, *HPRT1* [RTP]) are shown. Expression values for each HKG were calculated and used to obtain the geometric mean of the reference genes, which was converted back to Cq values. Values highlighted in red indicate measurements that were repeated. Subsequently, ΔCt values were determined as the difference between each target gene and the geometric mean of the housekeeping genes. These ΔCt values were compared with the median ΔCt of normal thymic tissue (N) to obtain ΔΔCt values. Fold changes (FC) were calculated using the formula $2^{-\Delta\Delta Cq}$, and final expression values are presented as log_2 fold change (log_2FC) for *YAP1*, *MST1*, *MOB1A*, and *TEAD4*. Sample N2 was excluded from the analysis because histology revealed a benign cyst.

	Target (Mean)				HKG (Mean)					HKG (geometric mean)	Delta Ct (Target- geometric mean HKG)			
Sample	YAP1	MST1	MOB1A	TEAD4	TBP	HPRT1 RTP	Expression value TBP	Expression value HPRT1 RTP	Gemoetric Mean	Back to Cq	YAP1	MST1	MOB1A	TEAD4
1 (NG)	33,88	29,12		34,57	31,81	29,57	2,65605E-10	1,25471E-09	5,77284E-10	30,69	3,19	-1,57	-2,22	3,88
2 (NG)*	33,67	30,3	29,31	35,49	32,78	30,79	1,35593E-10	5,38625E-10	2,70247E-10	31,785	1,885	-1,485	-2,475	3,705
3 (NG)	33,92	30,44	28,63	34,36	32,2	29,79	2,02691E-10	1,07725E-09	4,67278E-10	30,995	2,925	-0,555	-2,365	3,365
4 (A)	32,49	28,95	28,38	33,93	32,58	30,27	1,55755E-10	7,72364E-10	3,46843E-10	31,425	1,065	-2,475	-3,045	2,505
5 (A)	32,24	31,7	30,24	33,89	33,34	31,88	9,19729E-11	2,53025E-10	1,5255E-10	32,61	-0,37	-0,91	-2,37	1,28
6 (A)	30,31	30,91	28,45	32,85	31,97	29,96	2,37723E-10	9,57506E-10	4,77096E-10	30,965	-0,655	-0,055	-2,515	1,885
7 (B1)	33,15		28,04		31,25	29,15	3,91573E-10	1,67871E-09	8,10763E-10	30,2	2,95		-2,16	
8 (B1)	32	29,41	27,9	33,09	31,1	28,55	4,34477E-10	2,54445E-09	1,05143E-09	29,825	2,175	-0,415	-1,925	3,265
9 (B1)	30,6	27,1	26,2	32,16	29,15	27,11	1,67871E-09	6,90362E-09	3,40429E-09	28,13	2,47	-1,03	-1,93	4,03
10 (B1)	35,88	33,01	30,51	35,53	35,1	30,9	2,71548E-11	4,99083E-10	1,16415E-10	33	2,88	0,01	-2,49	2,53
11 (B1)	33,3	29,41	27,52	34,1	31,16	28,42	4,16779E-10	2,78437E-09	1,07725E-09	29,79	3,51	-0,38	-2,27	4,31
12 (B2)		26,28			28,45	26,58	2,72707E-09	9,96834E-09	5,21386E-09	27,515		-1,235		
13 (B2)	33,01	30,28	28,35	36,22	31,68	29,71	2,9065E-10	1,13867E-09	5,75287E-10	30,695	2,315	-0,415	-2,345	5,525
14 (B2)		29,36	27,59	33,65	32,03	28,04	2,28039E-10	3,62342E-09	9,09E-10	30,035		-0,675	-2,445	3,615
15 (B2)	33,75	31,84	30,24	34,12	34,21	31,41	5,03227E-11	3,50468E-10	1,32802E-10	32,81	0,94	-0,97	-2,57	1,31
16 (B2)	31,74	28,03	26,45	32,18	30,32	27,75	7,46054E-10	4,43014E-09	1,818E-09	29,035	2,705	-1,005	-2,585	3,145
17 (B3)	31,12	29,58	27,51	33,27	31,78	28,45	2,71186E-10	2,72707E-09	8,59967E-10	30,115	1,005	-0,535	-2,605	3,155
18 (B3)	28,53	26,12	25,4	31,93	29,17	27,33	1,6556E-09	5,92721E-09	3,13258E-09	28,25	0,28	-2,13	-2,85	3,68
19 (B3)	30,73	29,5	27,93	31,84	31,79	28,53	2,69312E-10	2,57997E-09	8,33557E-10	30,16	0,57	-0,66	-2,23	1,68
20 (B3)	30,71	30,07	28	32,57	31,12	31,54	4,28496E-10	3,20268E-10	3,70451E-10	31,33	-0,62	-1,26	-3,33	1,24
21 (B3)	32,35	31,33	29,51	34,2	33,77	30,49	6,8268E-11	6,63125E-10	2,12768E-10	32,13	0,22	-0,8	-2,62	2,07
22 (TC)	32,38	31,11	29,12	32,31	32,39	29,53	1,7768E-10	1,28998E-09	4,78753E-10	30,96	1,42	0,15	-1,84	1,35
23 (TC)	33,69		28,83	31,82	33,94	30,71	6,06795E-11	5,69336E-10	1,85868E-10	32,325	1,365		-3,495	-0,505
24 (TC)	33,86	31,6	29,13	33,3	32,76	29,81	1,37486E-10	1,06242E-09	3,82188E-10	31,285	2,575	0,315	-2,155	2,015
25 (TC)	34,65	32,4	29,73	31,67	34,23	28,84	4,96299E-11	2,08111E-09	3,2138E-10	31,535	3,115	0,865	-1,805	0,135
26 (TC)	33,79	32,28	30,17	32.42	34,21	30,6	5,03227E-11	6,14444E-10	1,75842E-10	32,405	1,385	-0,125	-2,235	0,015

	Median Deltact Normal thymic tissue (=N)				Deltadelta Ct (deltaCt target- Median deltaCt N)				Fold change (FC) : 2^(-deltadeltaCt) (Median NG 1 and 3)				Log2FC			
Sample	YAP1	MST1	MOB1A	TEAD4	YAP1	MST1	MOB1A	TEAD4	YAP1	MST1	MOB1A	TEAD4	YAP1	MST1	MOB1A	TEAD4
1 (NG)	3,0575	-1,0625	-2,2925	3,6225	0,1325	-0,5075	0,0725	0,2575	0,91	1,42	0,95	0,84	-0,13	0,51	-0,07	-0,26
2 (NG)*	3,0575	-1,0625	-2,2925	3,6225	-1,1725	-0,4225	-0,1825	0,0825	2,25	1,34	1,13	0,94	1,17	0,42	0,18	-0,08
3 (NG)	3,0575	-1,0625	-2,2925	3,6225	-0,1325	0,5075	-0,0725	-0,2575	1,10	0,70	1,05	1,20	0,13	-0,51	0,07	0,26
4 (A)	3,0575	-1,0625	-2,2925	3,6225	-1,9925	-1,4125	-0,7525	-1,1175	3,98	2,66	1,68	2,17	1,99	1,41	0,75	1,12
5 (A)	3,0575	-1,0625	-2,2925	3,6225	-3,4275	0,1525	-0,0775	-2,3425	10,76	0,90	1,06	5,07	3,43	-0,15	0,08	2,34
6 (A)	3,0575	-1,0625	-2,2925	3,6225	-3,7125	1,0075	-0,2225	-1,7375	13,11	0,50	1,17	3,33	3,71	-1,01	0,22	1,74
7 (B1)	3,0575		-2,2925	3,6225	-0,1075		0,1325		1,08		0,91		0,11		-0,13	
8 (B1)	3,0575	-1,0625	-2,2925	3,6225	-0,8825	0,6475	0,3675	-0,3575	1,84	0,64	0,78	1,28	0,88	-0,65	-0,37	0,36
9 (B1)	3,0575	-1,0625	-2,2925	3,6225	-0,5875	0,0325	0,3625	0,4075	1,50	0,98	0,78	0,75	0,59	-0,03	-0,36	-0,41
10 (B1)	3,0575	-1,0625	-2,2925	3,6225	-0,1775	1,0725	-0,1975	-1,0925	1,13	0,48	1,15	2,13	0,18	-1,07	0,20	1,09
11 (B1)	3,0575	-1,0625	-2,2925	3,6225	0,4525	0,6825	0,0225	0,6875	0,73	0,62	0,98	0,62	-0,45	-0,68	-0,02	-0,69
12 (B2)		-1,0625				-0,1725				1,13				0,17		
13 (B2)	3,0575	-1,0625	-2,2925	3,6225	-0,7425	0,6475	-0,0525	1,9025	1,67	0,64	1,04	0,27	0,74	-0,65	0,05	-1,90
14 (B2)		-1,0625	-2,2925	3,6225		0,3875	-0,1525	-0,0075		0,76	1,11	1,01		-0,39	0,15	0,01
15 (B2)	3,0575	-1,0625	-2,2925	3,6225	-2,1175	0,0925	-0,2775	-2,3125	4,34	0,94	1,21	4,97	2,12	-0,09	0,28	2,31
16 (B2)	3,0575	-1,0625	-2,2925	3,6225	-0,3525	0,0575	-0,2925	-0,4775	1,28	0,96	1,22	1,39	0,35	-0,06	0,29	0,48
17 (B3)	3,0575	-1,0625	-2,2925	3,6225	-2,0525	0,5275	-0,3125	-0,4675	4,15	0,69	1,24	1,38	2,05	-0,53	0,31	0,47
18 (B3)	3,0575	-1,0625	-2,2925	3,6225	-2,7775	-1,0675	-0,5575	0,0575	6,86	2,10	1,47	0,96	2,78	1,07	0,56	-0,06
19 (B3)	3,0575	-1,0625	-2,2925	3,6225	-2,4875	0,4025	0,0625	-1,9425	5,61	0,76	0,96	3,84	2,49	-0,40	-0,06	1,94
20 (B3)	3,0575	-1,0625	-2,2925	3,6225	-3,6775	-0,1975	-1,0375	-2,3825	12,79	1,15	2,05	5,21	3,68	0,20	1,04	2,38
21 (B3)	3,0575	-1,0625	-2,2925	3,6225	-2,8375	0,2625	-0,3275	-1,5525	7,15	0,83	1,25	2,93	2,84	-0,26	0,33	1,55
22 (TC)	3,0575	-1,0625	-2,2925	3,6225	-1,6375	1,2125	0,4525	-2,2725	3,11	0,43	0,73	4,83	1,64	-1,21	-0,45	2,27
23 (TC)	3,0575	-1,0625	-2,2925	3,6225	-1,6925		-1,2025	-4,1275	3,23		2,30	17,48	1,69		1,20	4,13
24 (TC)	3,0575	-1,0625	-2,2925	3,6225	-0,4825	1,3775	0,1375	-1,6075	1,40	0,38	0,91	3,05	0,48	-1,38	-0,14	1,61
25 (TC)	3,0575	-1,0625	-2,2925	3,6225	0,0575	1,9275	0,4875	-3,4875	0,96	0,26	0,71	11,22	-0,06	-1,93	-0,49	3,49
26 (TC)	3,0575	-1,0625	-2,2925	3,6225	-1,6725	0,9375	0,0575	-3,6075	3,19	0,52	0,96	12,19	1,67	-0,94	-0,06	3,61

* excluded from analysis

	Target (Mean)				HKG (Mean)					HKG (geometric mean)	Delta Ct (Target- geometric mean HKG)			
Repetitions	YAP1	MST1	MOB1A	TEAD4	TBP	HPRT1 RTP	Expression value TBP	Expression value HPRT1 RTP	Gemoetric Mean	Back to Cq	YAP1	MST1	MOB1A	TEAD4
Sample														
1 (NG)			25,99		29,26	27,16	1,55547E-09	6,66846E-09	3,22065E-09	28,21			-2,22	
7 (B1)		29,6		33,2	31,49	28,28	3,31563E-10	3,06812E-09	1,0086E-09	29,885		-0,285		3,315
12 (B2)	29,65		25,11	31,05	28,13	26,35	3,40429E-09	1,16912E-08	6,30874E-09	27,24	2,41		-2,13	3,81
14 (B2)	30,76				30,96	27,29	4,78753E-10	6,09385E-09	1,70805E-09	29,125	1,635			
23 (TC)		34,67			33,67	30,14	7,31678E-11	8,45193E-10	2,48678E-10	31,905		2,765		

	Median Deltact Normal thymic tissue (=N)				Deltadelta Ct (deltaCt target- Median deltaCt N)				Fold change (FC) : 2^(-deltadeltaCt) (Median NG 1 and 3)				Log2FC			
Repetitions	YAP1	MST1	MOB1A	TEAD4	YAP1	MST1	MOB1A	TEAD4	YAP1	MST1	MOB1A	TEAD4	YAP1	MST1	MOB1A	TEAD4
Sample																
1 (NG)			-2,2925				0,0725				0,95				-0,07	
7 (B1)		-1,0625		3,6225		0,7775		-0,3075		0,58		1,24		-0,78		0,31
12 (B2)	3,0575		-2,2925	3,6225	-0,6475		0,1625	0,1875	1,57		0,89	0,88	0,65		-0,16	-0,19
14 (B2)	3,0575				-1,4225				2,68				1,42			
23 (TC)		-1,0625				3,8275				0,07				-3,83		
