# Supplementary material for: Subtype-Dependent Expression Patterns of Core Hippo Pathway Components in Thymic Epithelial Tumors (TETs): An RT-qPCR Study
Source: Biomedicines. 2026 Jan 29;14(2):305. doi: 10.3390/biomedicines14020305 (PMC12937678; doi:10.3390/biomedicines14020305)
Supplement: Supplementary file 1 [file biomedicines-14-00305-s001.zip › Table S12 Calculation sheets for relative gene expression block 2.pdf]

Table S12. Calculation sheets for relative gene expression block 2. This table summarizes the stepwise calculations used to derive relative gene expression levels for block 2. For each sample, the mean Cq values of the target genes (*LATS1*, *SAV1*) and the housekeeping genes (*TBP*, *HPRT1* [RTP]) are shown. Expression values for each HKG were calculated and used to obtain the geometric mean of the reference genes, which was converted back to Cq values. Subsequently, ΔCt values were determined as the difference between each target gene and the geometric mean of the housekeeping genes. These ΔCt values were compared with the median ΔCt of normal thymic tissue (N) to obtain ΔΔCt values. Fold changes (FC) were calculated using the formula $2^{-\Delta\Delta Cq}$, and final expression values are presented as $\log_2$ fold change ($\log_2$FC) *LATS1* and *SAV1*. Sample N2 was excluded from the analysis because histology revealed a benign cyst.

			HKG (Mean)					HKG (geometr	Delta Ct (Target- geometric mean HKG)		Median Deltact Normal thymic tissue (=N)		Deltadelta Ct (deltaCt target- Median deltaCt N)		Fold change (FC) : 2^(-deltadeltaCt) (Median NG 1 and 3)		Log2FC	
Probe	LATS1	SAV1	TBP	HPRT1 RTP	Expression value TBP	Expression value HPRT1 RTP	Gemoetric Mean	Back to Cq	LATS1	SAV1	LATS1	SAV1	LATS1	SAV1	LATS1	SAV1	LATS1	SAV1
1 (NG)	28,96	29,19	30,1	30,36	8,68955E-10	7,25654E-10	7,94078E-10	30,23	-1,27	-1,04	-1,105	-1,165	-0,165	0,125	1,12	0,92	0,16	-0,13
2 (NG)	29,29	29,43	30,64	31,78	5,97642E-10	2,71186E-10	4,02582E-10	31,21	-1,92	-1,78	-1,105	-1,165	-0,815	-0,615	1,76	1,53	0,82	0,62
3 (NG)	30,56	30,21	31,41	31,59	3,50468E-10	3,09359E-10	3,29272E-10	31,5	-0,94	-1,29	-1,105	-1,165	0,165	-0,125	0,89	1,09	-0,16	0,13
4 (A)	28,23	26,96	29,11	29,81	1,7259E-09	1,06242E-09	1,35412E-09	29,46	-1,23	-2,5	-1,105	-1,165	-0,125	-1,335	1,09	2,52	0,13	1,34
5 (A)	30,19	27,35	31,46	33,2	3,38529E-10	1,01345E-10	1,85225E-10	32,33	-2,14	-4,98	-1,105	-1,165	-1,035	-3,815	2,05	14,07	1,04	3,82
6 (A)	28,39	26,21	29,2	30,05	1,62153E-09	8,99598E-10	1,20778E-09	29,625	-1,235	-3,415	-1,105	-1,165	-0,13	-2,25	1,09	4,76	0,13	2,25
7 (B1)	27,65	27,98	28,46	28,87	2,70823E-09	2,03828E-09	2,3495E-09	28,665	-1,015	-0,685	-1,105	-1,165	0,09	0,48	0,94	0,72	-0,09	-0,48
8 (B1)	31,1	29,59	31,22	31,54	3,99801E-10	3,20268E-10	3,57832E-10	31,38	-0,28	-1,79	-1,105	-1,165	0,825	-0,625	0,56	1,54	-0,83	0,63
9 (B1)	27,97	27,91	28,88	29,67	2,0242E-09	1,17068E-09	1,53938E-09	29,275	-1,305	-1,365	-1,105	-1,165	-0,2	-0,2	1,15	1,15	0,20	0,20
10 (B1)	31,95	31,28	32,32	31,93	1,86514E-10	2,44406E-10	2,13507E-10	32,125	-0,175	-0,845	-1,105	-1,165	0,93	0,32	0,52	0,80	-0,93	-0,32
11 (B1)	30,44	29,81	30,56	29,74	6,31718E-10	1,11524E-09	8,39355E-10	30,15	0,29	-0,34	-1,105	-1,165	1,395	0,825	0,38	0,56	-1,40	-0,82
12 (B2)	27,36	27,14	28,4	28,74	2,82324E-09	2,23048E-09	2,50942E-09	28,57	-1,21	-1,43	-1,105	-1,165	-0,105	-0,265	1,08	1,20	0,10	0,26
13 (B2)	31,5	30,35	32,08	32,3	2,20271E-10	1,89117E-10	2,04101E-10	32,19	-0,69	-1,84	-1,105	-1,165	0,415	-0,675	0,75	1,60	-0,42	0,67
14 (B2)	30,17	28,4	30,95	29,78	4,82083E-10	1,08474E-09	7,23143E-10	30,365	-0,195	-1,965	-1,105	-1,165	0,91	-0,8	0,53	1,74	-0,91	0,80
15 (B2)	31,16	29,3	32,26	32,93	1,94434E-10	1,22203E-10	1,54144E-10	32,595	-1,435	-3,295	-1,105	-1,165	-0,33	-2,13	1,26	4,38	0,33	2,13
16 (B2)	31	30,29	32,19	32,28	2,04101E-10	1,91757E-10	1,97833E-10	32,235	-1,235	-1,945	-1,105	-1,165	-0,13	-0,78	1,09	1,72	0,13	0,78
17 (B3)	29,35	27,83	30,7	30,16	5,73296E-10	8,33557E-10	6,91285E-10	30,43	-1,08	-2,6	-1,105	-1,165	0,025	-1,435	0,98	2,70	-0,03	1,44
18 (B3)	27,04	25,58	28,28	29,18	3,06812E-09	1,64416E-09	2,24599E-09	28,73	-1,69	-3,15	-1,105	-1,165	-0,585	-1,985	1,50	3,96	0,59	1,99
19 (B3)	30,02	27,17	30,75	29,94	5,53768E-10	9,70872E-10	7,33238E-10	30,345	-0,325	-3,175	-1,105	-1,165	0,78	-2,01	0,58	4,03	-0,78	2,01
20 (B3)	30,09	27,25	31,12	31,54	4,28496E-10	3,20268E-10	3,70451E-10	31,33	-1,24	-4,08	-1,105	-1,165	-0,135	-2,915	1,10	7,54	0,14	2,92
21 (B3)	31,11	28,01	32,07	31,71	2,21803E-10	2,84668E-10	2,51277E-10	31,89	-0,78	-3,88	-1,105	-1,165	0,325	-2,715	0,80	6,57	-0,32	2,72
22 (TC)	30,19	27,83	30,97	30,77	4,75446E-10	5,46144E-10	5,0957E-10	30,87	-0,68	-3,04	-1,105	-1,165	0,425	-1,875	0,74	3,67	-0,43	1,88
23 (TC)	34,76	32,64	33,25	32,74	9,78932E-11	1,39405E-10	1,16819E-10	32,995	1,765	-0,355	-1,105	-1,165	2,87	0,81	0,14	0,57	-2,87	-0,81
24 (TC)	29,5	29,05	30,24	29,63	7,88593E-10	1,2036E-09	9,74243E-10	29,935	-0,435	-0,885	-1,105	-1,165	0,67	0,28	0,63	0,82	-0,67	-0,28
25 (TC)	32,43	30,08	32,37	31,46	1,8016E-10	3,38529E-10	2,46961E-10	31,915	0,515	-1,835	-1,105	-1,165	1,62	-0,67	0,33	1,59	-1,62	0,67
26 (TC)	35,51	32,99	35,33	35,21	2,31532E-11	2,51613E-11	2,41364E-11	35,27	0,24	-2,28	-1,105	-1,165	1,345	-1,115	0,39	2,17	-1,34	1,12

* excluded from analysis
