# Supplementary material for: Subtype-Dependent Expression Patterns of Core Hippo Pathway Components in Thymic Epithelial Tumors (TETs): An RT-qPCR Study
Source: Biomedicines. 2026 Jan 29;14(2):305. doi: 10.3390/biomedicines14020305 (PMC12937678; doi:10.3390/biomedicines14020305)
Supplement: Supplementary file 1 [file biomedicines-14-00305-s001.zip › Table S13 Raw Cq data matrices.pdf]

Table S13. Raw Cq data matrices.

	A	B	C	D	E	F	G	H	I
1	File Name	Sample 1 und 2 NG.pcrd							
2	Created By	admin							
3	Notes								
4	ID								
5	Run Started	07/07/2025 10:09:08 UTC							
6	Run Ended	07/07/2025 12:05:24 UTC							
7	Sample Vol	20							
8	Lid Temp	105							
9	Protocol Fil	Originalprotokoll mit Schmelzkurve ab 60 Grad.prcl							
10	Plate Setup	Plattenvorlage PhD Projekt Ansatz.pltd							
11	Base Serial	BR207086							
12	Optical Hea	787BR15299							
13	CFX Manag	3.1.3086.0516.							
14									
15	Well group	All Wells							
16	Amplificatio	4							
17	Melt step	6							
18									
19									
20	Well	Fluor	Target	Content	Sample	Cq	Melt Temperature	Peak Height	End RFU
21	A01	SYBR	YAP1	Unkn	1 50ng	34,74	80,00	1342,84	2720,22
22	A03	SYBR	MST1	Unkn	1 50ng	29,22	75,60	3066,64	7275,44
23	A06	SYBR	TEAD4	Unkn	1 50ng	36,44	79,00	1486,82	2234,85
24	A09	SYBR	TBP	Unkn	1 50ng	31,78	78,20	2033,73	5331,82
25	A10	SYBR	HPRT1 RT	Unkn	1 50ng	29,42	77,60	2348,79	6533,50
26	A11	SYBR	YAP1	NTC	H20		None	None	0,74
27	A12	SYBR	TBP	NTC	H20		None	None	-0,60
28	B01	SYBR	YAP1	Unkn	1 50ng	33,99	80,00	1331,16	3448,57
29	B03	SYBR	MST1	Unkn	1 50ng	29,10	75,60	3133,92	7681,21
30	B06	SYBR	TEAD4	Unkn	1 50ng	34,58	79,00	1891,84	3839,29
31	B09	SYBR	TBP	Unkn	1 50ng	31,55	78,20	2195,61	5592,35
32	B10	SYBR	HPRT1 RT	Unkn	1 50ng	29,62	77,60	2408,09	6643,92
33	B12	SYBR	HPRT1 RT	NTC	H20		None	None	-2,05
34	C01	SYBR	YAP1	Unkn	1 50ng	33,68	80,20	1458,84	3434,44
35	C03	SYBR	MST1	Unkn	1 50ng	28,90	75,60	3286,85	8095,53
36	C06	SYBR	TEAD4	Unkn	1 50ng	34,47	78,80	1982,40	4049,72
37	C09	SYBR	TBP	Unkn	1 50ng	31,94	78,20	1949,37	5306,14
38	C10	SYBR	HPRT1 RT	Unkn	1 50ng	29,00	77,40	2540,69	7368,00
39	C11	SYBR	MST1	NTC	H20		None	None	-0,83
40	C12	SYBR	IC	Unkn	H20	25,96	80,80	4417,14	10103,55
41	D11	SYBR	MOB1A	NTC	H20		None	None	-0,60
42	D12	SYBR	IC	Unkn	H20	27,11	80,80	4436,03	10019,21
43	E12	SYBR	IC	Unkn	Probe 1	26,94	80,80	4145,80	9539,66
44	F01	SYBR	YAP1	Unkn	2 50ng	33,60	80,20	1445,72	3851,09
45	F03	SYBR	MST1	Unkn	2 50ng	30,18	75,60	2868,75	7017,38
46	F04	SYBR	MOB1A	Unkn	2 50ng	29,37	76,40	3103,35	7402,68
47	F06	SYBR	TEAD4	Unkn	2 50ng	35,34	78,80	1621,07	3213,88
48	F09	SYBR	TBP	Unkn	2 50ng	32,57	78,20	1976,79	5123,91
49	F10	SYBR	HPRT1 RT	Unkn	2 50ng	30,67	77,40	2434,13	6691,74
50	F11	SYBR	TEAD4	NTC	H20		None	None	-1,41
51	F12	SYBR	IC	Unkn	Probe 2	28,16	81,00	3994,43	9056,95
52	G01	SYBR	YAP1	Unkn	2 50ng	33,56	80,20	1443,43	3952,27
53	G03	SYBR	MST1	Unkn	2 50ng	30,42	75,60	2962,10	7017,35
54	G04	SYBR	MOB1A	Unkn	2 50ng	29,31	76,60	3115,82	7654,09

	A	B	C	D	E	F	G	H	I
55	G06	SYBR	TEAD4	Unkn	2 50ng	35,53	78,80	1683,24	3094,83
56	G09	SYBR	TBP	Unkn	2 50ng	32,60	78,20	1976,11	5146,75
57	G10	SYBR	HPRT1 RT	Unkn	2 50ng	30,80	77,60	2291,74	6365,90
58	G12	SYBR	YAP1	NRT	Probe 1	39,90	None	None	460,36
59	H01	SYBR	YAP1	Unkn	2 50ng	33,69	80,20	1352,94	3856,59
60	H03	SYBR	MST1	Unkn	2 50ng	30,16	75,60	2755,17	6969,03
61	H04	SYBR	MOB1A	Unkn	2 50ng	29,12	76,60	2910,44	7305,89
62	H06	SYBR	TEAD4	Unkn	2 50ng	36,08	78,80	1487,42	2681,90
63	H09	SYBR	TBP	Unkn	2 50ng	33,03	78,20	1948,58	4907,61
64	H10	SYBR	HPRT1 RT	Unkn	2 50ng	31,64	77,60	1999,87	5547,30
65	H12	SYBR	MST1	NRT	Probe 2		None	None	0,07
66	A07	SYBR	HPRT1 IDT	Unkn	1 50ng	30,74	73,20	3139,98	5760,72
67	A08	SYBR	PPIA	Unkn	1 50ng	26,88	80,00	2389,79	7150,99
68	B07	SYBR	HPRT1 IDT	Unkn	1 50ng	30,51	73,20	3155,52	5890,49
69	B08	SYBR	PPIA	Unkn	1 50ng	26,61	80,00	2481,42	7472,51
70	C07	SYBR	HPRT1 IDT	Unkn	1 50ng	31,20	73,20	3127,68	5517,97
71	C08	SYBR	PPIA	Unkn	1 50ng	26,59	79,80	2322,81	7169,70
72	F07	SYBR	HPRT1 IDT	Unkn	2 50ng	32,37	73,00	3115,28	5163,74
73	F08	SYBR	PPIA	Unkn	2 50ng	29,33	79,60	1880,85	6022,11
74	G07	SYBR	HPRT1 IDT	Unkn	2 50ng	32,61	73,00	2996,39	5040,41
75	G08	SYBR	PPIA	Unkn	2 50ng	28,73	79,60	1932,00	6451,27
76	G11	SYBR	HPRT1 IDT	NTC	H20	0,00	None	None	-1,45
77	H07	SYBR	HPRT1 IDT	Unkn	2 50ng	32,28	73,20	2832,18	5088,09
78	H08	SYBR	PPIA	Unkn	2 50ng	28,80	79,60	1756,29	6058,51
79	H11	SYBR	PPIA	NTC	H20	0,00	None	None	-1,09

	A	B	C	D	E	F	G	H	I
1	File Name	Sample 3 NG and Sample 4 Thymoma A.pcrd							
2	Created By	admin							
3	Notes								
4	ID								
5	Run Started	07/08/2025 13:40:15 UTC							
6	Run Ended	07/08/2025 15:37:11 UTC							
7	Sample Vol	20							
8	Lid Temp	105							
9	Protocol Fil	Originalprotokoll mit Schmelzkurve ab 60 Grad.prcl							
10	Plate Setup	Plattenvorlage PhD Projekt Ansatz.pltd							
11	Base Serial	BR207086							
12	Optical Hea	787BR15299							
13	CFX Manag	3.1.3086.0516.							
14									
15	Well group	All Wells							
16	Amplificatio	4							
17	Melt step	6							
18									
19									
20	Well	Fluor	Target	Content	Sample	Cq	Melt Temperature	Peak Height	End RFU
21	A11	SYBR	YAP1	NTC	H20		None	None	-1,26
22	A12	SYBR	TBP	NTC	H20		None	None	-1,84
23	B01	SYBR	YAP1	Unkn	3 50ng	33,91	80,00	1156,77	2940,75
24	B03	SYBR	MST1	Unkn	3 50ng	30,43	75,60	2332,81	5737,36
25	B04	SYBR	MOB1A	Unkn	3 50ng	28,63	76,60	2647,68	6478,45
26	B06	SYBR	TEAD4	Unkn	3 50ng	34,62	78,80	1494,15	3315,38
27	B09	SYBR	TBP	Unkn	3 50ng	32,05	78,20	1812,57	4644,17
28	B10	SYBR	HPRT1 RT	Unkn	3 50ng	29,87	77,60	2046,97	5783,89
29	B12	SYBR	HPRT1 RT	NTC	H20		None	None	-2,71
30	C01	SYBR	YAP1	Unkn	3 50ng	33,93	80,00	1088,65	3020,66
31	C03	SYBR	MST1	Unkn	3 50ng	36,20	75,60	1841,03	2247,16
32	C04	SYBR	MOB1A	Unkn	3 50ng	28,66	76,60	2605,70	6476,73
33	C06	SYBR	TEAD4	Unkn	3 50ng	35,43	78,80	1299,84	2603,65
34	C09	SYBR	TBP	Unkn	3 50ng	32,26	78,00	1613,75	4764,39
35	C10	SYBR	HPRT1 RT	Unkn	3 50ng	29,82	77,40	1981,76	5888,23
36	C11	SYBR	MST1	NTC	H20		None	None	-0,32
37	C12	SYBR	IC	Unkn	H20	25,57	80,80	3880,86	9064,56
38	D01	SYBR	YAP1	Unkn	3 50ng	34,54	80,20	1169,30	2891,30
39	D03	SYBR	MST1	Unkn	3 50ng	30,45	75,60	2319,84	5729,99
40	D04	SYBR	MOB1A	Unkn	3 50ng	28,60	76,40	2599,01	6594,17
41	D06	SYBR	TEAD4	Unkn	3 50ng	34,09	78,80	1517,24	3632,40
42	D09	SYBR	TBP	Unkn	3 50ng	32,30	78,00	1651,45	4742,76
43	D10	SYBR	HPRT1 RT	Unkn	3 50ng	29,67	77,40	2029,31	5955,99
44	D11	SYBR	MOB1A	NTC	H20		None	None	-2,05
45	D12	SYBR	IC	Unkn	H20	25,43	81,00	4013,18	9539,08
46	E01	SYBR	YAP1	Unkn	4 50ng	32,85	80,20	1164,20	3541,59
47	E03	SYBR	MST1	Unkn	4 50ng	28,81	75,60	2761,55	7024,31
48	E04	SYBR	MOB1A	Unkn	4 50ng	28,26	76,40	2796,83	7036,83
49	E06	SYBR	TEAD4	Unkn	4 50ng	33,62	33,62	1634,24	4093,92
50	E09	SYBR	TBP	Unkn	4 50ng	32,44	78,00	1660,77	4627,50
51	E10	SYBR	HPRT1 RT	Unkn	4 50ng	30,29	77,40	2062,88	5942,56
52	E12	SYBR	IC	Unkn	Probe 3	26,97	81,00	3749,69	8649,50
53	F01	SYBR	YAP1	Unkn	4 50ng	32,26	80,20	1322,49	4284,18
54	F03	SYBR	MST1	Unkn	4 50ng	29,32	75,60	2522,88	6398,48
55	F04	SYBR	MOB1A	Unkn	4 50ng	28,54	76,40	2605,08	6699,54

	A	B	C	D	E	F	G	H	I
56	F06	SYBR	TEAD4	Unkn	4 50ng	35,35	35,35	1366,10	2788,46
57	F09	SYBR	TBP	Unkn	4 50ng	32,99	78,00	1644,40	4327,99
58	F10	SYBR	HPRT1 RT	Unkn	4 50ng	30,22	77,40	2066,43	6036,59
59	F11	SYBR	TEAD4	NTC	H20		None	None	203,60
60	F12	SYBR	IC	Unkn	Probe 4	27,26	81,00	3859,57	8990,11
61	G01	SYBR	YAP1	Unkn	4 50ng	32,35	80,20	1336,34	4025,64
62	G03	SYBR	MST1	Unkn	4 50ng	28,73	75,60	2611,72	6810,96
63	G04	SYBR	MOB1A	Unkn	4 50ng	28,34	76,40	2689,33	6981,19
64	G06	SYBR	TEAD4	Unkn	4 50ng	34,23	34,23	1528,83	3695,11
65	G09	SYBR	TBP	Unkn	4 50ng	32,32	78,00	1528,14	4521,45
66	G10	SYBR	HPRT1 RT	Unkn	4 50ng	30,30	77,40	1989,44	5991,90
67	G12	SYBR	MST1	NRT	Probe 3		None	None	0,55
68	H12	SYBR	MOB1A	NRT	Probe 4		None	None	-2,18
69	B07	SYBR	HPRT1 IDT	Unkn	3 50ng	30,86	73,20	2801,35	5208,95
70	B08	SYBR	PPIA	Unkn	3 50ng	27,51	80,00	1890,56	6106,41
71	C07	SYBR	HPRT1 IDT	Unkn	3 50ng	31,38	73,20	2622,10	4753,86
72	C08	SYBR	PPIA	Unkn	3 50ng	27,29	79,80	1862,64	6063,42
73	D07	SYBR	HPRT1 IDT	Unkn	3 50ng	31,08	73,20	2818,86	5208,12
74	D08	SYBR	PPIA	Unkn	3 50ng	27,49	79,80	1799,81	5793,05
75	E07	SYBR	HPRT1 IDT	Unkn	4 50ng	31,74	73,20	2573,94	4562,33
76	E08	SYBR	PPIA	Unkn	4 50ng	27,70	79,80	1760,98	5679,03
77	F07	SYBR	HPRT1 IDT	Unkn	4 50ng	32,10	73,00	2662,73	4600,89
78	F08	SYBR	PPIA	Unkn	4 50ng	27,79	79,80	1919,00	6115,47
79	G07	SYBR	HPRT1 IDT	Unkn	4 50ng	32,10	73,00	2591,21	4661,16
80	G08	SYBR	PPIA	Unkn	4 50ng	27,70	79,80	1757,91	5733,56
81	G11	SYBR	HPRT1 IDT	NTC	H20	0,00	None	None	-1,78
82	H11	SYBR	PPIA	NTC	H20	0,00	None	None	-1,04

	A	B	C	D	E	F	G	H	I
1	File Name	Sample 5 and 6 Thymoma A.pcrd							
2	Created By	admin							
3	Notes								
4	ID								
5	Run Started	07/09/2025 07:48:47 UTC							
6	Run Ended	07/09/2025 09:45:36 UTC							
7	Sample Vol	20							
8	Lid Temp	105							
9	Protocol Fil	Originalprotokoll mit Schmelzkurve ab 60 Grad.prcl							
10	Plate Setup	Plattenvorlage PhD Projekt Ansatz.pltd							
11	Base Serial	BR207086							
12	Optical Hea	787BR15299							
13	CFX Manag	3.1.3086.0516.							
14									
15	Well group	All Wells							
16	Amplificatio	4							
17	Melt step	6							
18									
19									
20	Well	Fluor	Target	Content	Sample	Cq	Melt Temperature	Peak Height	End RFU
21	A11	SYBR	YAP1	NTC	H20		None	None	98,21
22	A12	SYBR	TBP	NTC	H20		None	None	-2,13
23	B01	SYBR	YAP1	Unkn	5 50ng	32,85	80,00	1239,46	3436,57
24	B03	SYBR	MST1	Unkn	5 50ng	31,83	75,60	2419,57	5017,13
25	B04	SYBR	MOB1A	Unkn	5 50ng	30,14	76,60	2613,23	5685,00
26	B06	SYBR	TEAD4	Unkn	5 50ng	33,44	79,00	1669,80	4183,85
27	B09	SYBR	TBP	Unkn	5 50ng	33,18	78,20	1659,87	3948,94
28	B10	SYBR	HPRT1 RT	Unkn	5 50ng	31,83	77,40	2012,60	4801,14
29	B12	SYBR	HPRT1 RT	NTC	H20		None	None	-1,06
30	C01	SYBR	YAP1	Unkn	5 50ng	32,35	80,00	1292,11	3529,57
31	C03	SYBR	MST1	Unkn	5 50ng	31,52	75,60	2388,20	5260,43
32	C04	SYBR	MOB1A	Unkn	5 50ng	30,29	76,60	2506,88	5561,55
33	C06	SYBR	TEAD4	Unkn	5 50ng	34,33	78,80	1514,46	3427,48
34	C09	SYBR	TBP	Unkn	5 50ng	33,48	78,20	1803,71	3904,31
35	C10	SYBR	HPRT1 RT	Unkn	5 50ng	31,92	77,40	1876,87	4617,39
36	C11	SYBR	MST1	NTC	H20		None	None	-0,53
37	C12	SYBR	IC	Unkn	H20	25,06	80,80	3849,95	8649,73
38	D01	SYBR	YAP1	Unkn	5 50ng	32,13	80,00	1307,23	4004,23
39	D03	SYBR	MST1	Unkn	5 50ng	31,76	75,60	2369,59	4939,79
40	D04	SYBR	MOB1A	Unkn	5 50ng	30,29	76,40	2474,68	5462,80
41	D06	SYBR	TEAD4	Unkn	5 50ng	34,45	78,80	1700,17	3359,71
42	D09	SYBR	TBP	Unkn	5 50ng	33,35	78,20	1788,76	4070,97
43	D10	SYBR	HPRT1 RT	Unkn	5 50ng	37,48	77,40	988,41	1340,02
44	D11	SYBR	MOB1A	NTC	H20		None	None	-1,81
45	D12	SYBR	IC	Unkn	H20	25,16	80,80	3681,38	8434,72
46	E01	SYBR	YAP1	Unkn	6 50ng	30,56	30,56	1438,21	4663,70
47	E03	SYBR	MST1	Unkn	6 50ng	30,61	75,60	2601,47	5971,19
48	E04	SYBR	MOB1A	Unkn	6 50ng	28,37	76,60	2829,44	6832,93
49	E06	SYBR	TEAD4	Unkn	6 50ng	32,53	32,53	1755,65	4806,81
50	E09	SYBR	TBP	Unkn	6 50ng	31,82	78,20	1833,08	4932,39
51	E10	SYBR	HPRT1 RT	Unkn	6 50ng	30,04	77,60	2179,39	6126,38
52	E12	SYBR	IC	Unkn	Probe 5	27,22	80,80	3508,12	7746,37
53	F01	SYBR	YAP1	Unkn	6 50ng	37,90	37,90	662,27	1095,67
54	F03	SYBR	MST1	Unkn	6 50ng	31,23	75,60	2484,31	5520,45
55	F04	SYBR	MOB1A	Unkn	6 50ng	28,54	76,40	2759,07	6710,46

	A	B	C	D	E	F	G	H	I
56	F06	SYBR	TEAD4	Unkn	6 50ng	37,91	37,91	698,37	1087,77
57	F09	SYBR	TBP	Unkn	6 50ng	32,17	78,20	1818,72	4747,16
58	F10	SYBR	HPRT1 RT	Unkn	6 50ng		None	None	-1,72
59	F11	SYBR	TEAD4	NTC	H20		None	None	-3,86
60	F12	SYBR	IC	Unkn	Probe 6	26,53	80,80	3743,20	8618,13
61	G01	SYBR	YAP1	Unkn	6 50ng	30,05	30,05	1512,43	5142,03
62	G03	SYBR	MST1	Unkn	6 50ng	30,89	75,60	2515,29	5758,95
63	G04	SYBR	MOB1A	Unkn	6 50ng	28,43	76,40	2803,05	6857,79
64	G06	SYBR	TEAD4	Unkn	6 50ng	33,17	33,17	1733,89	4429,11
65	G09	SYBR	TBP	Unkn	6 50ng	31,93	78,20	1838,02	4831,81
66	G10	SYBR	HPRT1 RT	Unkn	6 50ng	29,88	77,60	2279,66	6530,03
67	H12	SYBR	TEAD4	NRT	Probe 6		None	None	-1,36
68	B07	SYBR	HPRT1 IDT	Unkn	5 50ng	33,16	73,20	2823,61	3969,91
69	B08	SYBR	PPIA	Unkn	5 50ng	28,48	80,00	2168,53	5897,88
70	C07	SYBR	HPRT1 IDT	Unkn	5 50ng	33,27	73,20	2603,93	3726,85
71	C08	SYBR	PPIA	Unkn	5 50ng	28,33	79,80	1928,10	5235,06
72	D07	SYBR	HPRT1 IDT	Unkn	5 50ng	33,84	73,20	2533,13	3526,73
73	D08	SYBR	PPIA	Unkn	5 50ng	28,90	79,80	1986,16	5654,98
74	E07	SYBR	HPRT1 IDT	Unkn	6 50ng	31,50	73,20	2693,01	4566,45
75	E08	SYBR	PPIA	Unkn	6 50ng	26,51	79,80	2135,69	6251,36
76	F07	SYBR	HPRT1 IDT	Unkn	6 50ng	31,49	73,00	2951,55	5070,55
77	F08	SYBR	PPIA	Unkn	6 50ng	26,38	79,80	2297,30	6725,91
78	G07	SYBR	HPRT1 IDT	Unkn	6 50ng	32,01	73,00	2766,32	4690,62
79	G08	SYBR	PPIA	Unkn	6 50ng	30,66	79,80	1943,36	4837,57
80	G11	SYBR	HPRT1 IDT	NTC	H20		None	None	-1,40
81	H11	SYBR	PPIA	NTC	H20		None	None	-1,96

	A	B	C	D	E	F	G	H	I
1	File Name	Sample 7 B1 and NRT Sample 5.pcrd							
2	Created By	admin							
3	Notes								
4	ID								
5	Run Started	07/10/2025 11:08:20 UTC							
6	Run Ended	07/10/2025 13:05:07 UTC							
7	Sample Vol	20							
8	Lid Temp	105							
9	Protocol Fil	Originalprotokoll mit Schmelzkurve ab 60 Grad.prcl							
10	Plate Setup	Plattenvorlage PhD Projekt Ansatz.pltd							
11	Base Serial	BR207086							
12	Optical Hea	787BR15299							
13	CFX Manag	3.1.3086.0516.							
14									
15	Well group	All Wells							
16	Amplificatio	4							
17	Melt step	6							
18									
19									
20	Well	Fluor	Target	Content	Sample	Cq	Melt Temperature	Peak Height	End RFU
21	A02	SYBR	TEAD4	NRT	5 50ng		None	None	2,37
22	A11	SYBR	YAP1	NTC	H20		None	None	213,07
23	A12	SYBR	TBP	NTC	H20		None	None	-2,74
24	B01	SYBR	YAP1	Unkn	7 50ng	32,79	80,00	1106,64	3094,67
25	B04	SYBR	MOB1A	Unkn	7 50ng	28,02	76,60	2741,04	6657,53
26	B09	SYBR	TBP	Unkn	7 50ng	30,93	78,20	1757,43	4858,43
27	B10	SYBR	HPRT1 RT	Unkn	7 50ng	29,17	77,60	2081,71	5827,16
28	B12	SYBR	HPRT1 RT	NTC	H20		None	None	-1,51
29	C01	SYBR	YAP1	Unkn	7 50ng	33,50	80,00	1126,07	2788,54
30	C04	SYBR	MOB1A	Unkn	7 50ng	28,13	76,60	2540,41	6124,04
31	C09	SYBR	TBP	Unkn	7 50ng	31,48	78,20	1752,93	4647,43
32	C10	SYBR	HPRT1 RT	Unkn	7 50ng	29,21	77,40	2034,80	5812,12
33	C12	SYBR	IC	Unkn	H20	24,43	80,80	4152,68	9943,90
34	D01	SYBR	YAP1	Unkn	7 50ng	34,18	80,00	878,84	2524,41
35	D04	SYBR	MOB1A	Unkn	7 50ng	27,97	76,40	2763,62	6799,07
36	D09	SYBR	TBP	Unkn	7 50ng	31,33	78,00	1873,70	4995,76
37	D10	SYBR	HPRT1 RT	Unkn	7 50ng	29,06	77,40	2199,34	6353,03
38	D11	SYBR	MOB1A	NTC	H20		None	None	-1,64
39	D12	SYBR	IC	Unkn	H20	24,91	81,00	4017,52	9885,11
40	E12	SYBR	IC	Unkn	Probe 7	26,30	80,80	3543,06	7874,49
41	F11	SYBR	TEAD4	NTC	H20	38,25	None	None	744,99
42	B07	SYBR	HPRT1 IDT	Unkn	7 50ng	29,42	73,40	3044,65	5736,58
43	B08	SYBR	PPIA	Unkn	7 50ng	26,55	80,00	2141,11	6376,28
44	C07	SYBR	HPRT1 IDT	Unkn	7 50ng	31,82	73,20	2471,97	3834,93
45	C08	SYBR	PPIA	Unkn	7 50ng	27,02	79,80	1904,23	5650,25
46	D07	SYBR	HPRT1	Unkn	7 50ng	30,01	73,20	2947,96	5375,02
47	D08	SYBR	PPIA	Unkn	7 50ng	26,48	79,80	2046,73	6005,87
48	G11	SYBR	HPRT1 IDT	NTC	H20		None	None	-1,66
49	H08	SYBR	PPIA	NRT	7 50ng	31,12	79,80	1784,32	1797,70
50	H11	SYBR	PPIA	NTC	H20		None	None	-1,61

	A	B	C	D	E	F	G	H	I
1	File Name	Sample 8 B1, Repitition Sample 7 B1 and 23 TC.pcrd							
2	Created By	admin							
3	Notes								
4	ID								
5	Run Started	07/29/2025 13:58:09 UTC							
6	Run Ended	07/29/2025 15:53:57 UTC							
7	Sample Vol	20							
8	Lid Temp	105							
9	Protocol Fil	Originalprotokoll mit Schmelzkurve ab 60 Grad.prcl							
10	Plate Setup	Probe 8 B1, WDH Probe 7 B1 und 23 TC.pltd							
11	Base Serial	BR203528							
12	Optical Hea	787BR12390							
13	CFX Manag	3.1.3086.0516.							
14									
15	Well group	All Wells							
16	Amplificatio	4							
17	Melt step	6							
18									
19									
20	Well	Fluor	Target	Content	Sample	Cq	Melt Temperature	Peak Height	End RFU
21	A04	SYBR	TEAD4	Unkn	Probe 7 50ng	33,14	78,60	838,22	1903,41
22	A11	SYBR	YAP1	NTC	H20		None	None	2,07
23	A12	SYBR	TBP	NTC	H20		None	None	0,69
24	B02	SYBR	MST1	Unkn	Probe 7 50ng	29,62	75,40	1315,43	2879,22
25	B05	SYBR	TBP	Unkn	Probe 7 50ng	31,33	78,00	918,79	2326,22
26	B06	SYBR	HPRT1 RT	Unkn	Probe 7 50ng	28,37	77,40	1159,44	3125,35
27	B07	SYBR	MST1	Unkn	Probe 23 50ng		None	None	-1,27
28	B09	SYBR	TBP	Unkn	Probe 23 50ng	33,76	78,00	831,43	1656,59
29	B10	SYBR	HPRT1 RT	Unkn	Probe 23 50ng	30,17	77,20	1130,87	2859,93
30	B12	SYBR	HPRT1 RT	NTC	H20		None	None	-1,09
31	C02	SYBR	MST1	Unkn	Probe 7 50ng	29,57	75,40	1399,30	2934,32
32	C04	SYBR	TEAD4	Unkn	Probe 7 50ng	33,27	78,60	871,32	2016,91
33	C05	SYBR	TBP	Unkn	Probe 7 50ng	31,37	78,00	987,73	2360,41
34	C06	SYBR	HPRT1 RT	Unkn	Probe 7 50ng	28,29	77,20	1186,11	3244,42
35	C07	SYBR	MST1	Unkn	Probe 23 50ng	34,54	75,20	1008,90	1486,74
36	C09	SYBR	TBP	Unkn	Probe 23 50ng	33,37	77,80	844,01	1874,48
37	C10	SYBR	HPRT1 RT	Unkn	Probe 23 50ng	30,14	77,20	1207,91	3045,15
38	C11	SYBR	MST1	NTC	H20		None	None	-0,19
39	C12	SYBR	IC	Unkn	H20	24,38	80,80	2412,17	5576,45
40	D02	SYBR	MST1	Unkn	Probe 7 50ng	28,86	75,40	1420,00	3179,13
41	D04	SYBR	TEAD4	Unkn	Probe 7 50ng	33,19	78,60	797,38	2026,35
42	D05	SYBR	TBP	Unkn	Probe 7 50ng	31,77	78,00	923,79	2286,28
43	D06	SYBR	HPRT1 RT	Unkn	Probe 7 50ng	28,18	77,20	1182,37	3316,85
44	D07	SYBR	MST1	Unkn	Probe 23 50ng	34,80	75,00	711,82	1331,66
45	D09	SYBR	TBP	Unkn	Probe 23 50ng	33,89	77,80	804,85	1657,51
46	D10	SYBR	HPRT1 RT	Unkn	Probe 23 50ng	30,10	77,20	1107,43	3042,13
47	D11	SYBR	MOB1A	NTC	H20		None	None	-0,76
48	D12	SYBR	IC	Unkn	H20	24,55	81,00	2318,05	5302,13
49	E01	SYBR	YAP1	Unkn	Probe 8 50ng	32,01	80,00	612,49	1712,95
50	E03	SYBR	MST1	Unkn	Probe 8 50ng	29,38	75,40	1480,10	3190,16
51	E04	SYBR	MOB1A	Unkn	Probe 8 50ng	27,97	76,40	1527,05	3463,90
52	E06	SYBR	TEAD4	Unkn	Probe 8 50ng	32,94	78,60	903,02	2231,33
53	E09	SYBR	TBP	Unkn	Probe 8 50ng	31,03	78,00	1037,88	2687,05
54	E10	SYBR	HPRT1 RT	Unkn	Probe 8 50ng	30,06	77,40	1186,09	3077,63
55	E12	SYBR	IC	Unkn	Probe 8 50ng	25,21	81,00	2315,70	5383,55

	A	B	C	D	E	F	G	H	I
56	F01	SYBR	YAP1	Unkn	Probe 8 50ng	31,97	79,80	615,60	1807,48
57	F03	SYBR	MST1	Unkn	Probe 8 50ng	29,40	75,40	1358,18	3074,08
58	F04	SYBR	MOB1A	Unkn	Probe 8 50ng	27,98	76,40	1448,57	3372,24
59	F06	SYBR	TEAD4	Unkn	Probe 8 50ng	33,17	78,60	881,11	2172,62
60	F09	SYBR	TBP	Unkn	Probe 8 50ng	31,09	78,00	971,50	2601,53
61	F10	SYBR	HPRT1 RT	Unkn	Probe 8 50ng	28,52	77,40	1213,95	3387,07
62	F11	SYBR	TEAD4	NTC	H20	37,57	None	None	568,73
63	G01	SYBR	YAP1	Unkn	Probe 8 50ng	32,03	79,80	604,51	1727,93
64	G03	SYBR	MST1	Unkn	Probe 8 50ng	29,46	75,40	1378,99	3030,61
65	G04	SYBR	MOB1A	Unkn	Probe 8 50ng	27,75	76,40	1458,15	3397,23
66	G06	SYBR	TEAD4	Unkn	Probe 8 50ng	33,17	78,80	901,64	2179,07
67	G09	SYBR	TBP	Unkn	Probe 8 50ng	31,18	78,00	959,38	2605,93
68	G10	SYBR	HPRT1 RT	Unkn	Probe 8 50ng	28,57	77,20	1190,85	3394,93
69	H12	SYBR	YAP1	NRT	Probe 8 50ng		None	None	1,82

	A	B	C	D	E	F	G	H	I
1	File Name	Sample 9 and 10 B1 Thymoma.pcrd							
2	Created By	admin							
3	Notes								
4	ID								
5	Run Started	07/10/2025 15:32:26 UTC							
6	Run Ended	07/10/2025 17:29:09 UTC							
7	Sample Vol	20							
8	Lid Temp	105							
9	Protocol Fil	Originalprotokoll mit Schmelzkurve ab 60 Grad.prcl							
10	Plate Setup	Plattenvorlage PhD Projekt Ansatz.pltd							
11	Base Serial	BR207086							
12	Optical Hea	787BR15299							
13	CFX Manag	3.1.3086.0516.							
14									
15	Well group	All Wells							
16	Amplificatio	4							
17	Melt step	6							
18									
19									
20	Well	Fluor	Target	Content	Sample	Cq	Melt Temperature	Peak Height	End RFU
21	A03	SYBR	MST1	NRT	9 50ng		None	None	0,51
22	A04	SYBR	MOB1A	NRT	9 50ng		None	None	-0,87
23	A06	SYBR	TEAD4	NRT	9 50ng		None	None	0,18
24	A09	SYBR	TBP	NRT	9 50ng		None	None	0,12
25	A10	SYBR	HPRT1 RT	NRT	9 50ng		None	None	-1,61
26	A11	SYBR	YAP1	NTC	H20		None	None	-1,91
27	A12	SYBR	TBP	NTC	H20		None	None	-0,82
28	B01	SYBR	YAP1	Unkn	9 50ng	30,67	80,20	1412,35	3935,97
29	B03	SYBR	MST1	Unkn	9 50ng	27,21	75,60	2859,71	6770,29
30	B04	SYBR	MOB1A	Unkn	9 50ng	26,14	76,60	2888,39	7063,82
31	B06	SYBR	TEAD4	Unkn	9 50ng	32,17	79,00	1863,35	4658,14
32	B09	SYBR	TBP	Unkn	9 50ng	29,44	78,20	1963,73	5483,84
33	B10	SYBR	HPRT1 RT	Unkn	9 50ng	27,09	77,60	2219,18	6469,94
34	B12	SYBR	HPRT1 RT	NTC	H20		None	None	-2,22
35	C01	SYBR	YAP1	Unkn	9 50ng	30,82	80,20	1461,89	4070,79
36	C03	SYBR	MST1	Unkn	9 50ng	27,09	75,60	2942,32	6976,60
37	C04	SYBR	MOB1A	Unkn	9 50ng	26,24	76,60	2841,19	6917,31
38	C06	SYBR	TEAD4	Unkn	9 50ng	32,36	79,00	1872,48	4562,74
39	C09	SYBR	TBP	Unkn	9 50ng	29,17	78,20	2029,18	5857,33
40	C10	SYBR	HPRT1 RT	Unkn	9 50ng	27,17	77,60	2136,08	6363,55
41	C11	SYBR	MST1	NTC	H20		None	None	0,98
42	C12	SYBR	IC	Unkn	H20	25,12	80,80	3836,10	8884,92
43	D01	SYBR	YAP1	Unkn	9 50ng	30,52	80,20	1485,45	4382,85
44	D03	SYBR	MST1	Unkn	9 50ng	27,17	75,60	2733,27	6544,19
45	D04	SYBR	MOB1A	Unkn	9 50ng	26,39	76,40	2842,52	7017,40
46	D06	SYBR	TEAD4	Unkn	9 50ng	32,12	78,80	1827,04	4700,71
47	D09	SYBR	TBP	Unkn	9 50ng	29,05	78,20	2128,07	6087,44
48	D10	SYBR	HPRT1 RT	Unkn	9 50ng	27,21	77,60	2155,44	6327,92
49	D11	SYBR	MOB1A	NTC	H20		None	None	-0,50
50	D12	SYBR	IC	Unkn	H20	24,95	81,00	3918,57	9232,24
51	E01	SYBR	YAP1	Unkn	10 50ng	34,94	79,80	805,62	2322,05
52	E03	SYBR	MST1	Unkn	10 50ng	33,07	75,40	2047,33	4183,26
53	E04	SYBR	MOB1A	Unkn	10 50ng	30,51	76,40	2334,06	5404,76
54	E06	SYBR	TEAD4	Unkn	10 50ng	35,79	78,80	1201,14	2252,75
55	E09	SYBR	TBP	Unkn	10 50ng	35,05	78,00	1465,35	2724,68

	A	B	C	D	E	F	G	H	I
56	E10	SYBR	HPRT1 RT	Unkn	10 50ng	31,10	77,40	1914,36	5187,65
57	E12	SYBR	IC	Unkn	Probe 9	25,83	80,80	3601,18	8176,60
58	F01	SYBR	YAP1	Unkn	10 50ng	36,13	None	None	1762,06
59	F03	SYBR	MST1	Unkn	10 50ng	33,05	75,60	2114,46	4269,61
60	F04	SYBR	MOB1A	Unkn	10 50ng	30,57	76,40	2351,07	5304,74
61	F06	SYBR	TEAD4	Unkn	10 50ng	35,71	78,80	789,51	2219,80
62	F09	SYBR	TBP	Unkn	10 50ng	35,26	78,20	1451,59	2612,41
63	F10	SYBR	HPRT1 RT	Unkn	10 50ng	30,87	77,40	1875,85	5367,62
64	F11	SYBR	TEAD4	NTC	H20		None	None	6,43
65	F12	SYBR	IC	Unkn	Probe 10	25,00	81,00	3988,65	9396,73
66	G01	SYBR	YAP1	Unkn	10 50ng	35,77	None	None	1939,11
67	G03	SYBR	MST1	Unkn	10 50ng		None	None	-2,66
68	G04	SYBR	MOB1A	Unkn	10 50ng	30,64	76,40	2423,15	5564,06
69	G07	SYBR	TEAD4	Unkn	10 50ng	32,30	73,00	2414,13	4187,60
70	G09	SYBR	TBP	Unkn	10 50ng	35,14	78,20	1430,94	2651,85
71	G10	SYBR	HPRT1 RT	Unkn	10 50ng	30,93	77,40	1850,86	5188,18
72	A07	SYBR	HPRT1 IDT	NRT	9 50ng		None	None	-0,82
73	A08	SYBR	PPIA	NRT	9 50ng		None	None	86,73
74	B07	SYBR	HPRT1 IDT	Unkn	9 50ng	28,02	73,20	2908,77	5890,16
75	B08	SYBR	PPIA	Unkn	9 50ng	23,87	80,00	2410,24	6805,63
76	C07	SYBR	HPRT1 IDT	Unkn	9 50ng	28,21	73,20	3012,39	5840,89
77	C08	SYBR	PPIA	Unkn	9 50ng	23,98	80,00	2158,86	6196,04
78	D07	SYBR	HPRT1 IDT	Unkn	9 50ng	27,88	73,20	2934,90	5866,63
79	D08	SYBR	PPIA	Unkn	9 50ng	23,77	79,80	2233,89	6412,76
80	E07	SYBR	HPRT1 IDT	Unkn	10 50ng	32,88	73,00	2359,25	3678,36
81	E08	SYBR	PPIA	Unkn	10 50ng	29,74	79,60	1503,33	4646,53
82	F07	SYBR	HPRT1 IDT	Unkn	10 50ng	32,14	73,00	2538,47	4299,81
83	F08	SYBR	PPIA	Unkn	10 50ng	29,65	79,60	1680,68	5176,84
84	G06	SYBR	HPRT1 IDT	Unkn	10 50ng	35,28	78,60	1197,92	2643,82
85	G08	SYBR	PPIA	Unkn	10 50ng	29,91	79,60	1627,20	4885,59
86	G11	SYBR	HPRT1 IDT	NTC	H20		None	None	3,86
87	G12	SYBR	PPIA	NRT	Probe 10		None	None	-0,16
88	H11	SYBR	PPIA	NTC	H20		None	None	-1,19

	A	B	C	D	E	F	G	H	I
1	File Name	Sample 11 B1 and 12 B2 Thymoma.pcrd							
2	Created By	admin							
3	Notes								
4	ID								
5	Run Started	07/11/2025 10:28:04 UTC							
6	Run Ended	07/11/2025 12:24:55 UTC							
7	Sample Vol	20							
8	Lid Temp	105							
9	Protocol Fil	Originalprotokoll mit Schmelzkurve ab 60 Grad.prcl							
10	Plate Setup	Plattenvorlage PhD Projekt Ansatz.pltd							
11	Base Serial	BR207086							
12	Optical Hea	787BR15299							
13	CFX Manag	3.1.3086.0516.							
14									
15	Well group	All Wells							
16	Amplificatio	4							
17	Melt step	6							
18									
19									
20	Well	Fluor	Target	Content	Sample	Cq	Melt Temperature	Peak Height	End RFU
21	A11	SYBR	YAP1	NTC	H20		None	None	5,95
22	A12	SYBR	TBP	NTC	H20		None	None	-1,39
23	B01	SYBR	YAP1	Unkn	11 50ng		None	None	0,33
24	B03	SYBR	MST1	Unkn	11 50ng	29,64	75,60	2143,96	5165,44
25	B04	SYBR	MOB1A	Unkn	11 50ng	27,63	76,60	2337,60	5808,70
26	B06	SYBR	TEAD4	Unkn	11 50ng	34,11	34,11	1186,17	2951,64
27	B09	SYBR	TBP	Unkn	11 50ng	31,47	78,20	1563,06	4262,80
28	B10	SYBR	HPRT1 RT	Unkn	11 50ng	28,37	77,60	1904,58	5580,78
29	B12	SYBR	HPRT1 RT	NTC	H20		None	None	-0,32
30	C01	SYBR	YAP1	Unkn	11 50ng	33,16	80,00	869,45	2617,97
31	C03	SYBR	MST1	Unkn	11 50ng	29,33	75,60	2264,07	5478,28
32	C04	SYBR	MOB1A	Unkn	11 50ng	27,53	76,60	2323,51	5891,26
33	C06	SYBR	TEAD4	Unkn	11 50ng	33,14	33,14	1618,08	3661,74
34	C09	SYBR	TBP	Unkn	11 50ng	31,16	78,20	1566,43	4444,54
35	C10	SYBR	HPRT1 RT	Unkn	11 50ng	28,49	77,40	1892,05	5585,02
36	C11	SYBR	MST1	NTC	H20		None	None	-0,95
37	C12	SYBR	IC	Unkn	H20	24,66	80,80	3841,13	8970,44
38	D01	SYBR	YAP1	Unkn	11 50ng	33,44	80,00	844,83	2629,30
39	D03	SYBR	MST1	Unkn	11 50ng	29,27	75,60	2158,37	5335,83
40	D04	SYBR	MOB1A	Unkn	11 50ng	27,40	76,60	2378,24	6099,70
41	D06	SYBR	TEAD4	Unkn	11 50ng	34,08	34,08	1246,85	3116,12
42	D09	SYBR	TBP	Unkn	11 50ng	30,86	78,20	1710,22	4905,19
43	D10	SYBR	HPRT1 RT	Unkn	11 50ng	28,41	77,60	1997,33	5947,83
44	D11	SYBR	MOB1A	NTC	H20		None	None	-1,49
45	D12	SYBR	IC	Unkn	H20	24,94	81,00	3897,39	9218,46
46	E03	SYBR	MST1	Unkn	12 50ng	26,34	75,60	2921,90	7439,59
47	E09	SYBR	TBP	Unkn	12 50ng	28,46	78,20	1799,83	5714,61
48	E10	SYBR	HPRT1 RT	Unkn	12 50ng	26,57	77,60	2175,04	6723,71
49	E12	SYBR	IC	Unkn	Probe 11	26,29	81,00	3577,68	8123,82
50	F03	SYBR	MST1	Unkn	12 50ng	26,23	75,60	2724,81	7116,66
51	F09	SYBR	TBP	Unkn	12 50ng	28,43	78,20	1828,26	5905,81
52	F10	SYBR	HPRT1 RT	Unkn	12 50ng	26,60	77,60	2181,19	6842,23
53	F11	SYBR	TEAD4	NTC	H20		None	None	163,29
54	F12	SYBR	IC	Unkn	Probe 12	25,40	81,00	3825,82	9013,95
55	G03	SYBR	MST1	Unkn	12 50ng	26,26	75,60	2673,48	7136,20

	A	B	C	D	E	F	G	H	I
56	G09	SYBR	TBP	Unkn	12 50ng	29,87	78,20	1759,37	5276,41
57	G10	SYBR	HPRT1 RT	Unkn	12 50ng	26,57	77,60	2193,98	6927,25
58	H01	SYBR	YAP1	NRT	Probe 11 50ng		None	None	32,39
59	H03	SYBR	MST1	NRT	Probe 11 50ng		None	None	1,21
60	H04	SYBR	MOB1A	NRT	Probe 11 50ng		None	None	-0,96
61	H09	SYBR	TBP	NRT	Probe 12 50ng		None	None	-1,60
62	H10	SYBR	HPRT1 RT	NRT	Probe 11 50ng		None	None	-1,61
63	B07	SYBR	HPRT1 IDT	Unkn	11 50ng	28,34	73,40	2584,74	5399,26
64	B08	SYBR	PPIA	Unkn	11 50ng	26,41	80,00	1764,12	5672,71
65	C07	SYBR	HPRT1 IDT	Unkn	11 50ng	28,28	73,20	2537,01	5402,24
66	C08	SYBR	PPIA	Unkn	11 50ng	26,45	79,80	1664,79	5346,39
67	D07	SYBR	HPRT1 IDT	Unkn	11 50ng	28,44	73,20	2670,11	5506,24
68	D08	SYBR	PPIA	Unkn	11 50ng	26,62	79,80	1617,46	5168,45
69	E07	SYBR	HPRT1 IDT	Unkn	12 50ng	27,18	73,20	2791,63	6012,53
70	E08	SYBR	PPIA	Unkn	12 50ng	22,92	79,80	1942,10	6339,74
71	F07	SYBR	HPRT1 IDT	Unkn	12 50ng	26,73	73,20	2916,19	6422,59
72	F08	SYBR	PPIA	Unkn	12 50ng	28,40	79,80	1865,31	5203,01
73	G07	SYBR	HPRT1 IDT	Unkn	12 50ng	27,11	73,20	2827,79	6206,28
74	G08	SYBR	PPIA	Unkn	12 50ng	23,13	79,80	2103,44	6640,88
75	G11	SYBR	HPRT1 IDT	NTC	H20		None	None	-1,84
76	H07	SYBR	HPRT1 IDT	NRT	Probe 12 50ng		None	None	-1,14
77	H08	SYBR	PPIA	NRT	Probe 12 50ng	38,03	None	None	930,83
78	H11	SYBR	PPIA	NTC	H20		None	None	-1,38

	A	B	C	D	E	F	G	H	I
1	File Name	Sample 13 and Probe 16 B2 Thymoma.pcrd							
2	Created By	admin							
3	Notes								
4	ID								
5	Run Started	07/30/2025 14:06:27 UTC							
6	Run Ended	07/30/2025 16:02:39 UTC							
7	Sample Vol	20							
8	Lid Temp	105							
9	Protocol Fil	Originalprotokoll mit Schmelzkurve ab 60 Grad.prcl							
10	Plate Setup	Plattenvorlage PhD Projekt Ansatz.pltd							
11	Base Serial	BR203528							
12	Optical Hea	787BR12390							
13	CFX Manag	3.1.3086.0516.							
14									
15	Well group	All Wells							
16	Amplificatio	4							
17	Melt step	6							
18									
19									
20	Well	Fluor	Target	Content	Sample	Cq	Melt Temperature	Peak Height	End RFU
21	A11	SYBR	YAP1	NTC	H20		None	None	77,44
22	A12	SYBR	TBP	NTC	H20		None	None	1,13
23	B01	SYBR	YAP1	Unkn	Probe 13 50ng	34,41	79,60	424,83	1029,60
24	B03	SYBR	MST1	Unkn	Probe 13 50ng	30,43	75,40	1234,83	2688,83
25	B04	SYBR	MOB1A	Unkn	Probe 13 50ng	28,37	76,40	1469,15	3237,92
26	B06	SYBR	TEAD4	Unkn	Probe 13 50ng	35,48	78,60	603,02	1131,79
27	B09	SYBR	TBP	Unkn	Probe 13 50ng	32,32	78,00	998,87	2102,24
28	B10	SYBR	HPRT1 RT	Unkn	Probe 13 50ng	29,73	77,40	1204,14	3019,47
29	B11	SYBR	TAZ	NTC	H20		None	None	-1,84
30	B12	SYBR	HPRT1 RT	NTC	H20		None	None	165,96
31	C01	SYBR	YAP1	Unkn	Probe 13 50ng	33,14	79,80	537,08	1354,22
32	C03	SYBR	MST1	Unkn	Probe 13 50ng	30,21	75,40	1339,78	2857,72
33	C04	SYBR	MOB1A	Unkn	Probe 13 50ng	28,40	76,40	1493,92	3341,43
34	C06	SYBR	TEAD4	Unkn	Probe 13 50ng	36,26	78,60	562,57	907,33
35	C09	SYBR	TBP	Unkn	Probe 13 50ng	31,69	78,00	984,24	2389,71
36	C10	SYBR	HPRT1 RT	Unkn	Probe 13 50ng	29,14	77,40	1237,40	3309,14
37	C11	SYBR	MST1	NTC	H20		None	None	0,93
38	C12	SYBR	IC	Unkn	H20	23,57	81,00	2537,74	5815,73
39	D01	SYBR	YAP1	Unkn	Probe 13 50ng	32,87	79,80	540,06	1489,01
40	D03	SYBR	MST1	Unkn	Probe 13 50ng	30,21	75,40	1395,27	2921,62
41	D04	SYBR	MOB1A	Unkn	Probe 13 50ng	28,27	76,40	1483,74	3403,15
42	D06	SYBR	TEAD4	Unkn	Probe 13 50ng	36,17	78,60	568,24	953,98
43	D09	SYBR	TBP	Unkn	Probe 13 50ng	31,67	78,00	902,92	2376,78
44	D10	SYBR	HPRT1 RT	Unkn	Probe 13 50ng	29,68	77,40	1127,13	2971,55
45	D11	SYBR	MOB1A	NTC	H20		None	None	-0,41
46	D12	SYBR	IC	Unkn	H20	23,47	81,00	2411,26	5518,23
47	E01	SYBR	YAP1	Unkn	Probe 16 50ng	32,03	79,80	563,49	1671,22
48	E03	SYBR	MST1	Unkn	Probe 16 50ng	28,03	75,40	1421,37	3411,82
49	E04	SYBR	MOB1A	Unkn	Probe 16 50ng	26,49	76,40	1479,27	3615,24
50	E06	SYBR	TEAD4	Unkn	Probe 16 50ng	32,16	78,60	915,44	2404,32
51	E09	SYBR	TBP	Unkn	Probe 16 50ng	30,43	78,00	961,79	2716,03
52	E10	SYBR	HPRT1 RT	Unkn	Probe 16 50ng	27,80	77,40	1191,16	3443,85
53	E12	SYBR	IC	Unkn	Probe 13 50ng	24,20	80,80	2047,20	5037,01
54	F01	SYBR	YAP1	Unkn	Probe 16 50ng	31,65	79,80	538,42	1757,90
55	F03	SYBR	MST1	Unkn	Probe 16 50ng	27,98	75,40	1416,85	3381,14

	A	B	C	D	E	F	G	H	I
56	F04	SYBR	MOB1A	Unkn	Probe 16 50ng	26,36	76,40	1493,44	3775,83
57	F06	SYBR	TEAD4	Unkn	Probe 16 50ng	32,14	78,60	885,83	2452,18
58	F09	SYBR	TBP	Unkn	Probe 16 50ng	30,21	78,00	986,77	2833,37
59	F10	SYBR	HPRT1 RT	Unkn	Probe 16 50ng	27,73	77,40	1243,64	3629,18
60	F11	SYBR	TEAD4	NTC	H20		None	None	82,37
61	F12	SYBR	IC	Unkn	Probe 16 50ng	24,08	80,60	1977,74	4815,37
62	G01	SYBR	YAP1	Unkn	Probe 16 50ng	31,54	79,80	567,24	1790,65
63	G03	SYBR	MST1	Unkn	Probe 16 50ng	28,09	75,40	1408,87	3447,04
64	G04	SYBR	MOB1A	Unkn	Probe 16 50ng	26,50	76,40	1442,21	3585,58
65	G06	SYBR	TEAD4	Unkn	Probe 16 50ng	32,25	78,60	873,28	2494,33
66	G09	SYBR	TBP	Unkn	Probe 16 50ng	30,33	78,00	919,98	2810,10
67	G10	SYBR	HPRT1 RT	Unkn	Probe 16 50ng	27,71	77,40	1199,88	3522,32
68	G12	SYBR	TBP	NRT	Probe 13 50ng		None	None	0,15
69	H12	SYBR	HPRT1 RT	NRT	Probe 16 50ng		None	None	0,83
70	B07	SYBR	HPRT1 IDT	Unkn	Probe 13 50ng	31,02	73,00	1574,59	2192,89
71	B08	SYBR	PPIA	Unkn	Probe 13 50ng	27,31	79,60	1102,20	2973,36
72	C07	SYBR	HPRT1 IDT	Unkn	Probe 13 50ng	31,09	73,00	1631,75	2357,35
73	C08	SYBR	PPIA	Unkn	Probe 13 50ng	27,19	79,60	1144,05	3185,38
74	D07	SYBR	HPRT1 IDT	Unkn	Probe 13 50ng	31,12	73,00	1592,86	2391,16
75	D08	SYBR	PPIA	Unkn	Probe 13 50ng	27,14	79,60	1097,04	3096,92
76	E07	SYBR	HPRT1 IDT	Unkn	Probe 16 50ng	29,37	73,00	1637,40	2835,90
77	E08	SYBR	PPIA	Unkn	Probe 16 50ng	25,35	79,60	1044,90	3281,60
78	F07	SYBR	HPRT1 IDT	Unkn	Probe 16 50ng	29,71	73,00	1575,00	2724,28
79	F08	SYBR	PPIA	Unkn	Probe 16 50ng	25,33	79,60	1003,51	3226,14
80	G07	SYBR	HPRT1 IDT	Unkn	Probe 16 50ng	29,09	73,00	1646,22	2994,00
81	G08	SYBR	PPIA	Unkn	Probe 16 50ng	25,28	79,60	1038,53	3356,13
82	G11	SYBR	HPRT1 IDT	NTC	H20		None	None	-0,28
83	H11	SYBR	PPIA	NTC	H20		None	None	0,82

	A	B	C	D	E	F	G	H	I
1	File Name	Sample 14 B2 Thymoma.pcrd							
2	Created By	admin							
3	Notes								
4	ID								
5	Run Started	07/11/2025 15:05:02 UTC							
6	Run Ended	07/11/2025 17:01:53 UTC							
7	Sample Vol	20							
8	Lid Temp	105							
9	Protocol Fil	Originalprotokoll mit Schmelzkurve ab 60 Grad.prcl							
10	Plate Setup	Plattenvorlage PhD Projekt Ansatz.pltd							
11	Base Serial	BR207086							
12	Optical Hea	787BR15299							
13	CFX Manag	3.1.3086.0516.							
14									
15	Well group	All Wells							
16	Amplificatio	4							
17	Melt step	6							
18									
19									
20	Well	Fluor	Target	Content	Sample	Cq	Melt Temperature	Peak Height	End RFU
21	A11	SYBR	YAP1	NTC	H20		None	None	-1,10
22	A12	SYBR	TBP	NTC	H20		None	None	-1,15
23	B12	SYBR	HPRT1 RT	NTC	H20		None	None	0,13
24	C11	SYBR	MST1	NTC	H20		None	None	0,04
25	C12	SYBR	IC	Unkn	H20	27,90	80,80	4749,51	10623,28
26	D11	SYBR	MOB1A	NTC	H20		None	None	-0,97
27	D12	SYBR	IC	Unkn	H20	25,31	81,00	5138,12	12358,29
28	E03	SYBR	MST1	Unkn	Probe 14 50ng	29,47	75,60	3285,43	7893,89
29	E04	SYBR	MOB1A	Unkn	Probe 14 50ng	27,58	76,40	3292,10	8179,99
30	E06	SYBR	TEAD4	Unkn	Probe 14 50ng	33,78	33,78	2123,07	4597,26
31	E09	SYBR	TBP	Unkn	Probe 14 50ng	32,11	78,20	2360,55	5947,26
32	E10	SYBR	HPRT1 RT	Unkn	Probe 14 50ng	28,04	77,40	2892,53	8834,30
33	F03	SYBR	MST1	Unkn	Probe 14 50ng	30,73	75,60	3191,79	7214,80
34	F04	SYBR	MOB1A	Unkn	Probe 14 50ng	27,57	76,40	3425,77	8572,48
35	F06	SYBR	TEAD4	Unkn	Probe 14 50ng	33,52	33,52	2174,94	4828,41
36	F09	SYBR	TBP	Unkn	Probe 14 50ng	32,14	78,20	2394,98	5759,19
37	F10	SYBR	HPRT1 RT	Unkn	Probe 14 50ng	28,02	77,40	2875,81	8871,23
38	F11	SYBR	TEAD4	NTC	H20	38,48	None	None	848,34
39	F12	SYBR	IC	Unkn	Probe 14	26,13	80,80	4917,63	11799,34
40	G03	SYBR	MST1	Unkn	Probe 14 50ng	29,26	75,60	3296,25	8054,92
41	G04	SYBR	MOB1A	Unkn	Probe 14 50ng	27,61	76,40	3492,30	8889,15
42	G09	SYBR	TBP	Unkn	Probe 14 50ng	31,83	78,00	2089,40	5875,26
43	G06	SYBR	TEAD4	Unkn	Probe 14 50ng	34,43	34,43	1774,73	3949,39
44	G10	SYBR	HPRT1 RT	Unkn	Probe 14 50ng	28,05	77,40	2625,71	8333,24
45	H01	SYBR	YAP1	NRT	Probe 14 50ng		None	None	-2,13
46	H03	SYBR	MST1	NRT	Probe 14 50ng		None	None	-1,24
47	H04	SYBR	MOB1A	NRT	Probe 14 50ng		None	None	-0,40
48	H06	SYBR	TEAD4	NRT	Probe 14 50ng	38,30	None	None	933,97
49	H09	SYBR	TBP	NRT	Probe 14 50ng		None	None	-2,48
50	H10	SYBR	HPRT1 RT	NRT	Probe 14 50ng		None	None	-1,69
51	E07	SYBR	HPRT1 IDT	Unkn	14 50ng	29,01	73,00	3806,61	7452,06
52	E08	SYBR	PPIA	Unkn	14 50ng	25,91	79,80	2831,09	8383,92
53	F07	SYBR	HPRT1 IDT	Unkn	14 50ng	29,29	73,00	3768,26	7264,30
54	F08	SYBR	PPIA	Unkn	14 50ng	25,83	79,80	2815,52	8204,63
55	G07	SYBR	HPRT1 IDT	Unkn	14 50ng	29,21	73,00	3748,74	7446,65

	A	B	C	D	E	F	G	H	I
56	G08	SYBR	PPIA	Unkn	14 50ng	25,68	79,80	2847,21	8631,44
57	G11	SYBR	HPRT1 IDT	NTC	H20		None	None	-0,21
58	H07	SYBR	HPRT1 IDT	NRT	Probe 14 50ng		None	None	-2,14
59	H08	SYBR	PPIA	NRT	Probe 14 50ng		None	None	-0,74
60	H11	SYBR	PPIA	NTC	H20		None	None	0,04

	A	B	C	D	E	F	G	H	I
1	File Name	2025-07-14 Sample 15 B2 Thymoma.pcrd							
2	Created By	admin							
3	Notes								
4	ID								
5	Run Started	07/14/2025 13:44:32 UTC							
6	Run Ended	07/14/2025 15:40:57 UTC							
7	Sample Vol	20							
8	Lid Temp	105							
9	Protocol Fil	Originalprotokoll mit Schmelzkurve ab 60 Grad.prcl							
10	Plate Setup	Plattenvorlage PhD Projekt Ansatz.pltd							
11	Base Serial	BR207086							
12	Optical Hea	787BR15299							
13	CFX Manag	3.1.3086.0516.							
14									
15	Well group	All Wells							
16	Amplificatio	4							
17	Melt step	6							
18									
19									
20	Well	Fluor	Target	Content	Sample	Cq	Melt Temperature	Peak Height	End RFU
21	A11	SYBR	YAP1	NTC	H20		None	None	5,24
22	A12	SYBR	TBP	NTC	H20		None	None	0,24
23	B01	SYBR	YAP1	Unkn	Probe 15 50ng	33,78	80,00	1327,71	3197,36
24	B03	SYBR	MST1	Unkn	Probe 15 50ng	32,09	75,60	2396,86	4935,99
25	B04	SYBR	MOB1A	Unkn	Probe 15 50ng	29,98	76,60	2662,55	6295,99
26	B06	SYBR	TEAD4	Unkn	Probe 15 50ng	34,14	78,80	1584,39	3890,74
27	B09	SYBR	TBP	Unkn	Probe 15 50ng	33,44	78,20	1699,88	4114,58
28	B10	SYBR	HPRT1 RT	Unkn	Probe 15 50ng	31,53	77,40	2170,72	5632,94
29	B12	SYBR	HPRT1 RT	NTC	H20		None	None	-0,52
30	C01	SYBR	YAP1	Unkn	Probe 15 50ng	33,62	80,00	1179,01	3324,19
31	C03	SYBR	MST1	Unkn	Probe 15 50ng	31,63	75,60	2636,02	5683,14
32	C04	SYBR	MOB1A	Unkn	Probe 15 50ng	30,27	76,60	2675,98	6249,50
33	C06	SYBR	TEAD4	Unkn	Probe 15 50ng	33,87	78,80	1717,48	4176,87
34	C09	SYBR	TBP	Unkn	Probe 15 50ng	34,00	78,20	1779,68	3971,48
35	C10	SYBR	HPRT1 RT	Unkn	Probe 15 50ng	31,43	77,40	2182,75	5767,08
36	C11	SYBR	MST1	NTC	H20		None	None	-0,57
37	C12	SYBR	IC	Unkn	H20	25,75	80,80	4615,05	11055,05
38	D01	SYBR	YAP1	Unkn	Probe 15 50ng	33,85	80,00	1226,26	3313,01
39	D03	SYBR	MST1	Unkn	Probe 15 50ng	31,79	75,60	2510,89	5472,58
40	D04	SYBR	MOB1A	Unkn	Probe 15 50ng	30,48	76,40	2623,94	6045,80
41	D06	SYBR	TEAD4	Unkn	Probe 15 50ng	34,36	78,80	1760,66	3815,99
42	D09	SYBR	TBP	Unkn	Probe 15 50ng	34,41	78,00	1653,24	3597,13
43	D10	SYBR	HPRT1 RT	Unkn	Probe 15 50ng	31,26	77,40	2276,52	6038,26
44	D11	SYBR	MOB1A	NTC	H20		None	None	-0,48
45	D12	SYBR	IC	Unkn	H20	25,80	81,00	4399,18	10524,44
46	E12	SYBR	IC	Unkn	Probe 15	26,45	81,00	4356,52	10388,46
47	F11	SYBR	TEAD4	NTC	H20		None	None	68,51
48	B07	SYBR	PPIA	Unkn	Probe 15 50ng	29,54	80,00	1868,66	5478,98
49	B08	SYBR	HPRT1 IDT	Unkn	Probe 15 50ng	39,06	73,40	947,69	743,94
50	C07	SYBR	PPIA	Unkn	Probe 15 50ng	29,33	79,80	1947,20	5778,91
51	C08	SYBR	HPRT1 IDT	Unkn	Probe 15 50ng	33,33	73,20	2651,67	4094,93
52	D07	SYBR	PPIA	Unkn	Probe 15 50ng	29,53	79,80	1935,07	5616,16
53	D08	SYBR	HPRT1 IDT	Unkn	Probe 15 50ng	33,74	73,20	2729,09	3942,69
54	G11	SYBR	HPRT1 IDT	NTC	H20		None	None	0,27
55	G12	SYBR	HPRT1 IDT	NRT	Probe 15		None	None	0,94

	A	B	C	D	E	F	G	H	I
56	H11	SYBR	PPIA	NTC	H20		None	None	-1,26

	A	B	C	D	E	F	G	H	I
1	File Name	Sample 17 und 18 B3 Thymoma.pcrd							
2	Created By	admin							
3	Notes								
4	ID								
5	Run Started	07/15/2025 12:58:12 UTC							
6	Run Ended	07/15/2025 14:54:21 UTC							
7	Sample Vol	20							
8	Lid Temp	105							
9	Protocol Fil	Originalprotokoll mit Schmelzkurve ab 60 Grad.prcl							
10	Plate Setup	Plattenvorlage PhD Projekt Ansatz.pltd							
11	Base Serial	BR207086							
12	Optical Hea	787BR15299							
13	CFX Manag	3.1.3086.0516.							
14									
15	Well group	All Wells							
16	Amplificatio	4							
17	Melt step	6							
18									
19									
20	Well	Fluor	Target	Content	Sample	Cq	Melt Temperature	Peak Height	End RFU
21	A11	SYBR	YAP1	NTC	H20		None	None	-0,39
22	A12	SYBR	TBP	NTC	H20		None	None	-0,00
23	B01	SYBR	YAP1	Unkn	Probe 17 50ng	31,19	80,00	1469,16	4594,50
24	B03	SYBR	MST1	Unkn	Probe 17 50ng	29,63	75,60	2799,96	6776,46
25	B04	SYBR	MOB1A	Unkn	Probe 17 50ng	27,51	76,60	2974,61	7693,40
26	B06	SYBR	TEAD4	Unkn	Probe 17 50ng	33,41	79,00	1836,32	4632,63
27	B09	SYBR	TBP	Unkn	Probe 17 50ng	31,90	78,20	2021,29	5531,51
28	B10	SYBR	HPRT1 RT	Unkn	Probe 17 50ng	28,54	77,60	2505,39	7607,41
29	B12	SYBR	HPRT1 RT	NTC	H20		None	None	37,24
30	C01	SYBR	YAP1	Unkn	Probe 17 50ng	31,22	80,20	1388,98	4456,01
31	C03	SYBR	MST1	Unkn	Probe 17 50ng	29,68	75,60	2857,89	7025,98
32	C04	SYBR	MOB1A	Unkn	Probe 17 50ng	27,56	76,60	3114,15	8086,34
33	C06	SYBR	TEAD4	Unkn	Probe 17 50ng	33,11	78,80	1982,90	5110,91
34	C09	SYBR	TBP	Unkn	Probe 17 50ng	31,81	78,20	2086,29	5740,31
35	C10	SYBR	HPRT1 RT	Unkn	Probe 17 50ng	28,46	77,40	2480,10	7584,19
36	C11	SYBR	MST1	NTC	H20		None	None	-1,51
37	C12	SYBR	IC	Unkn	H20	25,35	80,80	4922,91	11816,38
38	D01	SYBR	YAP1	Unkn	Probe 17 50ng	30,96	80,20	1479,19	4889,72
39	D03	SYBR	MST1	Unkn	Probe 17 50ng	29,44	75,60	3037,20	7419,37
40	D04	SYBR	MOB1A	Unkn	Probe 17 50ng	27,47	76,60	2999,86	7859,96
41	D06	SYBR	TEAD4	Unkn	Probe 17 50ng	33,30	78,80	1955,76	4906,70
42	D09	SYBR	TBP	Unkn	Probe 17 50ng	31,64	78,20	2123,80	5844,86
43	D10	SYBR	HPRT1 RT	Unkn	Probe 17 50ng	28,36	77,40	2462,84	7650,13
44	D11	SYBR	MOB1A	NTC	H20		None	None	0,31
45	D12	SYBR	IC	Unkn	H20	25,52	81,00	4734,53	11532,94
46	E01	SYBR	YAP1	Unkn	Probe 18 50ng	28,54	80,20	1773,91	5859,76
47	E03	SYBR	MST1	Unkn	Probe 18 50ng	28,07	75,20	2661,92	6492,43
48	E04	SYBR	MOB1A	Unkn	Probe 18 50ng	25,33	76,40	3344,47	8964,83
49	E06	SYBR	TEAD4	Unkn	Probe 18 50ng	31,98	78,80	2282,78	6247,47
50	E09	SYBR	TBP	Unkn	Probe 18 50ng	29,17	78,20	2388,44	7277,80
51	E10	SYBR	HPRT1 RT	Unkn	Probe 18 50ng	27,30	77,40	2596,96	8182,33
52	E12	SYBR	IC	Unkn	Probe 17	25,98	81,00	4539,61	11159,18
53	F01	SYBR	YAP1	Unkn	Probe 18 50ng	28,62	80,20	1854,78	6296,03
54	F03	SYBR	MST1	Unkn	Probe 18 50ng	25,96	75,60	3535,56	9233,72
55	F04	SYBR	MOB1A	Unkn	Probe 18 50ng	25,50	76,40	3201,86	8728,64

	A	B	C	D	E	F	G	H	I
56	F06	SYBR	TEAD4	Unkn	Probe 18 50ng	31,71	78,80	2247,22	6359,83
57	F09	SYBR	TBP	Unkn	Probe 18 50ng	29,18	78,20	2386,98	7299,57
58	F10	SYBR	HPRT1 RT	Unkn	Probe 18 50ng	27,43	77,60	2710,27	8721,60
59	F11	SYBR	TEAD4	NTC	H20		None	None	-0,60
60	F12	SYBR	IC	Unkn	Probe 18	25,19	80,80	4824,88	11804,58
61	G01	SYBR	YAP1	Unkn	Probe 18 50ng	28,43	80,20	1767,11	6043,45
62	G03	SYBR	MST1	Unkn	Probe 18 50ng	26,28	75,60	3405,26	8897,42
63	G04	SYBR	MOB1A	Unkn	Probe 18 50ng	25,38	76,40	3324,07	9211,54
64	G06	SYBR	TEAD4	Unkn	Probe 18 50ng	32,09	78,80	2052,96	5944,15
65	G09	SYBR	TBP	Unkn	Probe 18 50ng	29,16	78,20	2363,25	7287,74
66	G10	SYBR	HPRT1 RT	Unkn	Probe 18 50ng	27,26	77,40	2591,07	8280,29
67	G12	SYBR	HPRT1 RT	NRT	Probe 17		None	None	1,30
68	H12	SYBR	YAP1	NRT	Probe 18		None	None	0,82
69	B07	SYBR	HPRT1 IDT	Unkn	Probe 17 50ng	30,08	73,20	3200,86	6123,10
70	B08	SYBR	PPIA	Unkn	Probe 17 50ng	25,98	80,00	2326,62	7312,87
71	C07	SYBR	HPRT1 IDT	Unkn	Probe 17 50ng	29,70	73,20	3347,51	6526,97
72	C08	SYBR	PPIA	Unkn	Probe 17 50ng	25,96	79,80	2340,93	7470,12
73	D07	SYBR	HPRT1 IDT	Unkn	Probe 17 50ng	30,11	73,20	3265,50	6226,16
74	D08	SYBR	PPIA	Unkn	Probe 17 50ng	25,76	79,80	2347,79	7465,55
75	E07	SYBR	HPRT1 IDT	Unkn	Probe 18 50ng	28,85	73,00	3560,01	7180,53
76	E08	SYBR	PPIA	Unkn	Probe 18 50ng	23,90	79,80	2814,62	8565,50
77	F07	SYBR	HPRT1 IDT	Unkn	Probe 18 50ng	28,63	73,00	3489,58	7190,55
78	F08	SYBR	PPIA	Unkn	Probe 18 50ng	23,74	79,80	2709,30	8417,17
79	G07	SYBR	HPRT1 IDT	Unkn	Probe 18 50ng	28,52	73,00	3512,69	7446,97
80	G08	SYBR	PPIA	Unkn	Probe 18 50ng	23,70	79,80	2730,22	8493,05
81	G11	SYBR	HPRT1 IDT	NTC	H20		None	None	-0,88
82	H08	SYBR	PPIA	NRT	Probe 18 50ng		None	None	-1,23
83	H11	SYBR	PPIA	NTC	H20		None	None	-1,57

	A	B	C	D	E	F	G	H	I
1	File Name	Sample 19 B3 Thymoma and NRT and IC Sample 20.pcrd							
2	Created By	admin							
3	Notes								
4	ID								
5	Run Started	07/16/2025 13:07:37 UTC							
6	Run Ended	07/16/2025 15:03:55 UTC							
7	Sample Vol	20							
8	Lid Temp	105							
9	Protocol Fil	Originalprotokoll mit Schmelzkurve ab 60 Grad.prcl							
10	Plate Setup	Plattenvorlage PhD Projekt Ansatz.pltd							
11	Base Serial	BR207086							
12	Optical Hea	787BR15299							
13	CFX Manag	3.1.3086.0516.							
14									
15	Well group	All Wells							
16	Amplificatio	4							
17	Melt step	6							
18									
19									
20	Well	Fluor	Target	Content	Sample	Cq	Melt Temperature	Peak Height	End RFU
21	A06	SYBR	TEAD4	Unkn	Probe 19 50ng	32,43	79,00	2207,43	5577,10
22	A11	SYBR	YAP1	NTC	H20		None	None	5,65
23	A12	SYBR	TBP	NTC	H20		None	None	1,21
24	B01	SYBR	YAP1	Unkn	Probe 19 50ng	30,89	80,00	1616,37	4849,23
25	B03	SYBR	MST1	Unkn	Probe 19 50ng	29,44	75,60	3123,17	7309,47
26	B04	SYBR	MOB1A	Unkn	Probe 19 50ng	27,87	76,60	3119,06	7729,86
27	B09	SYBR	TBP	Unkn	Probe 19 50ng	31,74	78,20	2264,43	5768,04
28	B10	SYBR	HPRT1 RT	Unkn	Probe 19 50ng	28,64	77,60	2554,00	7489,90
29	B12	SYBR	HPRT1 RT	NTC	H20		None	None	-0,77
30	C01	SYBR	YAP1	Unkn	Probe 19 50ng	31,41	80,00	1587,11	4679,96
31	C03	SYBR	MST1	Unkn	Probe 19 50ng	29,46	75,60	3154,45	7395,24
32	C04	SYBR	MOB1A	Unkn	Probe 19 50ng	27,91	76,60	3147,70	7752,73
33	C06	SYBR	TEAD4	Unkn	Probe 19 50ng	31,73	79,00	2347,86	6396,12
34	C09	SYBR	TBP	Unkn	Probe 19 50ng	31,59	78,20	2352,20	6102,77
35	C10	SYBR	HPRT1 RT	Unkn	Probe 19 50ng	28,54	77,40	2624,31	7742,83
36	C11	SYBR	MST1	NTC	H20		None	None	-0,04
37	C12	SYBR	IC	Unkn	H20	25,32	80,80	4968,74	11983,93
38	D01	SYBR	YAP1	Unkn	Probe 19 50ng	30,56	80,20	1595,97	5236,56
39	D03	SYBR	MST1	Unkn	Probe 19 50ng	29,59	75,60	3078,36	7279,45
40	D04	SYBR	MOB1A	Unkn	Probe 19 50ng	28,02	76,40	3155,69	7865,43
41	D06	SYBR	TEAD4	Unkn	Probe 19 50ng	31,94	78,80	2334,72	6349,56
42	D09	SYBR	TBP	Unkn	Probe 19 50ng	32,05	78,20	2327,58	5838,93
43	D10	SYBR	HPRT1 RT	Unkn	Probe 19 50ng	28,40	77,40	2540,81	7540,86
44	D11	SYBR	MOB1A	NTC	H20		None	None	-1,50
45	D12	SYBR	IC	Unkn	H20	25,42	80,80	4779,61	11628,70
46	E12	SYBR	IC	Unkn	Probe 19	25,54	81,00	4714,85	11459,70
47	F11	SYBR	TEAD4	NTC	H20		None	None	-3,27
48	F12	SYBR	IC	Unkn	Probe 20	25,75	81,00	4755,06	11680,75
49	G12	SYBR	HPRT1 RT	NRT	Probe 19		None	None	-0,37
50	H12	SYBR	YAP1	NRT	Probe 20		None	None	3,51
51	B07	SYBR	HPRT1 IDT	Unkn	Probe 19 50ng	29,52	73,20	3481,95	6624,57
52	B08	SYBR	PPIA	Unkn	Probe 19 50ng	25,43	80,00	2721,42	7815,62
53	C07	SYBR	HPRT1 IDT	Unkn	Probe 19 50ng	29,79	73,20	3567,85	6654,19
54	C08	SYBR	PPIA	Unkn	Probe 19 50ng	25,46	80,00	2637,13	7740,70
55	D07	SYBR	HPRT1 IDT	Unkn	Probe 19 50ng	30,08	73,20	3520,59	6512,52

	A	B	C	D	E	F	G	H	I
56	D08	SYBR	PPIA	Unkn	Probe 19 50ng	25,57	79,80	2623,81	7709,62
57	G11	SYBR	HPRT1 IDT	NTC	H20		None	None	86,27
58	H11	SYBR	PPIA	NTC	H20		None	None	-1,54

	A	B	C	D	E	F	G	H	I
1	File Name	Sample 20 B3 Thymoma.pcrd							
2	Created By	admin							
3	Notes								
4	ID								
5	Run Started	10/08/2025 15:36:47 UTC							
6	Run Ended	10/08/2025 17:33:12 UTC							
7	Sample Vol	20							
8	Lid Temp	105							
9	Protocol Fil	Originalprotokoll mit Schmelzkurve ab 60 Grad.prcl							
10	Plate Setup	Plattenvorlage Wiederholungsplatte IDT Primer.pltd							
11	Base Serial	BR203528							
12	Optical Hea	787BR12390							
13	CFX Manag	3.1.3086.0516.							
14									
15	Well group	All Wells							
16	Amplificatio	4							
17	Melt step	6							
18									
19									
20	Well	Fluor	Target	Content	Sample	Cq	Melt Temperature	Peak Height	End RFU
21	A10	SYBR	HPRT1 RT	NTC	H20		None	None	-0,44
22	A11	SYBR	TBP	NTC	H20		None	None	0,64
23	B04	SYBR	YAP1	Unkn	Pr. 20 50ng	30,99	79,80	511,95	1815,07
24	B05	SYBR	TEAD4	Unkn	Pr. 20 50ng	32,55	78,60	741,75	1989,19
25	B06	SYBR	MST1	Unkn	Pr. 20 50ng	30,08	75,40	1152,35	2681,61
26	B07	SYBR	MOB1A	Unkn	Pr. 20 50ng	28,20	76,40	1235,13	2973,10
27	B08	SYBR	HPRT1 RT	Unkn	Pr. 20 50ng	31,65	77,20	825,50	1941,75
28	B09	SYBR	TBP	Unkn	Pr. 20 50ng	31,02	78,00	831,44	2295,21
29	B12	SYBR	LATS1	NTC	H20		None	None	-1,32
30	C04	SYBR	YAP1	Unkn	Pr. 20 50ng	30,44	79,80	552,26	1904,59
31	C05	SYBR	TEAD4	Unkn	Pr. 20 50ng	32,46	78,60	752,02	2033,64
32	C06	SYBR	MST1	Unkn	Pr. 20 50ng	29,04	75,20	1214,09	2960,47
33	C07	SYBR	MOB1A	Unkn	Pr. 20 50ng	27,79	76,40	1260,18	3098,41
34	C08	SYBR	HPRT1 RT	Unkn	Pr. 20 50ng	31,47	77,20	800,77	1993,51
35	C09	SYBR	TBP	Unkn	Pr. 20 50ng	31,20	78,00	847,59	2345,79
36	D01	SYBR	TAZ	Unkn	Pr. 20 50ng	30,54	80,20	226,06	1505,83
37	D04	SYBR	YAP1	Unkn	Pr. 20 50ng	30,71	79,80	507,88	1847,75
38	D05	SYBR	TEAD4	Unkn	Pr. 20 50ng	32,71	78,60	693,50	1919,53
39	D06	SYBR	MST1	Unkn	Pr. 20 50ng	30,05	75,40	1195,49	2638,66
40	D07	SYBR	MOB1A	Unkn	Pr. 20 50ng	30,14	76,20	1085,32	2279,72
41	D08	SYBR	HPRT1 RT	Unkn	Pr. 20 50ng	31,49	77,20	802,74	1991,02
42	D09	SYBR	TBP	Unkn	Pr. 20 50ng	31,15	77,80	771,32	2251,63
43	D12	SYBR	YAP1	NTC	H20		None	None	27,42
44	E12	SYBR	TEAD4	NTC	H20	38,32	None	None	334,78
45	F12	SYBR	MST1	NTC	H20		None	None	0,80
46	G12	SYBR	MOB1A	NTC	H20		None	None	0,10
47	H12	SYBR	SAV1	NRT	Pr. 26 50ng		None	None	4,98
48	E07	SYBR	HPRT1 IDT	Unkn	Probe 20 50ng	31,94	73,00	3211,61	5606,82
49	E08	SYBR	PPIA	Unkn	Probe 20 50ng	27,53	79,80	2165,44	7123,42
50	F07	SYBR	HPRT1 IDT	Unkn	Probe 20 50ng	31,88	73,00	3045,97	5561,16
51	F08	SYBR	PPIA	Unkn	Probe 20 50ng	27,70	79,80	2081,31	6890,37
52	G07	SYBR	HPRT1 IDT	Unkn	Probe 20 50ng	31,76	73,00	3178,11	5819,93
53	G08	SYBR	PPIA	Unkn	Probe 20 50ng	27,49	79,80	2057,86	7036,53
54	G11	SYBR	HPRT1 IDT	NTC	H20		None	None	86,27
55	H08	SYBR	PPIA	NRT	Probe 20 50ng		None	None	1,25

	A	B	C	D	E	F	G	H	I
56	H11	SYBR	PPIA	NTC	H20		None	None	-1,54

	A	B	C	D	E	F	G	H	I
1	File Name	Sample 21 B3 Thymoma and 22 TC.pcrd							
2	Created By	admin							
3	Notes								
4	ID								
5	Run Started	07/17/2025 12:11:06 UTC							
6	Run Ended	07/17/2025 14:07:18 UTC							
7	Sample Vol	20							
8	Lid Temp	105							
9	Protocol Fil	Originalprotokoll mit Schmelzkurve ab 60 Grad.prcl							
10	Plate Setup	Plattenvorlage PhD Projekt Ansatz.pltd							
11	Base Serial	BR207086							
12	Optical Hea	787BR15299							
13	CFX Manag	3.1.3086.0516.							
14									
15	Well group	All Wells							
16	Amplificatio	4							
17	Melt step	6							
18									
19									
20	Well	Fluor	Target	Content	Sample	Cq	Melt Temperature	Peak Height	End RFU
21	A06	SYBR	TEAD4	Unkn	Probe 21 50ng	34,49	78,80	1365,38	3205,00
22	A11	SYBR	YAP1	NTC	H20		None	None	22,78
23	A12	SYBR	TBP	NTC	H20		None	None	0,90
24	B01	SYBR	YAP1	Unkn	Probe 21 50ng	32,38	80,00	1158,02	3486,28
25	B03	SYBR	MST1	Unkn	Probe 21 50ng	32,23	75,60	2273,39	4719,83
26	B04	SYBR	MOB1A	Unkn	Probe 21 50ng	29,35	76,60	2431,01	5829,36
27	B09	SYBR	TBP	Unkn	Probe 21 50ng	33,94	78,20	1567,46	3472,90
28	B10	SYBR	HPRT1 RT	Unkn	Probe 21 50ng	30,44	77,40	2054,13	5819,78
29	B12	SYBR	HPRT1 RT	NTC	H20		None	None	18,99
30	C01	SYBR	YAP1	Unkn	Probe 21 50ng	33,11	80,00	1088,06	3234,63
31	C03	SYBR	MST1	Unkn	Probe 21 50ng	31,60	75,60	2297,32	5122,79
32	C04	SYBR	MOB1A	Unkn	Probe 21 50ng	29,70	76,60	2530,33	5970,18
33	C06	SYBR	TEAD4	Unkn	Probe 21 50ng	34,17	78,80	1569,83	3731,20
34	C09	SYBR	TBP	Unkn	Probe 21 50ng	33,71	78,00	1535,18	3758,67
35	C10	SYBR	HPRT1 RT	Unkn	Probe 21 50ng	30,44	77,40	2111,56	5899,34
36	C11	SYBR	MST1	NTC	H20		None	None	0,92
37	C12	SYBR	IC	Unkn	H20	26,23	80,80	3995,74	9541,10
38	D01	SYBR	YAP1	Unkn	Probe 21 50ng	32,32	80,00	1109,69	3587,72
39	D03	SYBR	MST1	Unkn	Probe 21 50ng	31,05	75,60	2273,03	5393,70
40	D04	SYBR	MOB1A	Unkn	Probe 21 50ng	29,47	76,40	2447,48	5944,33
41	D06	SYBR	TEAD4	Unkn	Probe 21 50ng	33,93	78,60	1557,49	3947,80
42	D09	SYBR	TBP	Unkn	Probe 21 50ng	33,66	78,00	1616,85	3854,50
43	D10	SYBR	HPRT1 RT	Unkn	Probe 21 50ng	30,59	77,40	1924,87	5499,20
44	D11	SYBR	MOB1A	NTC	H20		None	None	-0,95
45	D12	SYBR	IC	Unkn	H20	26,25	81,00	3839,13	9232,93
46	E01	SYBR	YAP1	Unkn	Probe 22 50ng	32,30	80,20	1270,53	3732,31
47	E03	SYBR	MST1	Unkn	Probe 22 50ng	31,07	75,60	2532,34	5752,38
48	E04	SYBR	MOB1A	Unkn	Probe 22 50ng	29,35	76,40	2591,47	6182,58
49	E06	SYBR	TEAD4	Unkn	Probe 22 50ng	32,45	78,80	1722,22	4863,52
50	E09	SYBR	TBP	Unkn	Probe 22 50ng	32,52	78,20	1876,52	4665,94
51	E10	SYBR	HPRT1 RT	Unkn	Probe 22 50ng	29,47	77,60	2123,32	6260,72
52	E12	SYBR	IC	Unkn	Probe 21	27,65	80,80	2848,09	6399,93
53	F01	SYBR	YAP1	Unkn	Probe 22 50ng	32,39	80,20	1276,87	3883,52
54	F03	SYBR	MST1	Unkn	Probe 22 50ng	31,21	75,60	2352,57	5287,66
55	F04	SYBR	MOB1A	Unkn	Probe 22 50ng	28,89	76,40	2516,31	6353,42

	A	B	C	D	E	F	G	H	I
56	F06	SYBR	TEAD4	Unkn	Probe 22 50ng	32,34	78,80	1901,24	5077,54
57	F09	SYBR	TBP	Unkn	Probe 22 50ng	32,18	78,20	1867,08	4879,74
58	F10	SYBR	HPRT1 RT	Unkn	Probe 22 50ng	29,39	77,40	2120,86	6237,43
59	F11	SYBR	TEAD4	NTC	H20		None	None	2,60
60	F12	SYBR	IC	Unkn	Probe 22	26,75	81,00	3869,98	9246,98
61	G01	SYBR	YAP1	Unkn	Probe 22 50ng	32,44	80,20	1201,61	3768,41
62	G03	SYBR	MST1	Unkn	Probe 22 50ng	31,05	75,60	2357,18	5543,58
63	G04	SYBR	MOB1A	Unkn	Probe 22 50ng		None	None	-0,35
64	G09	SYBR	TBP	Unkn	Probe 22 50ng	32,48	78,20	1791,46	4647,98
65	G10	SYBR	HPRT1 RT	Unkn	Probe 22 50ng	29,72	77,60	2035,32	6075,32
66	G12	SYBR	MOB1A	NRT	Probe 21		None	None	0,44
67	H06	SYBR	TEAD4	Unkn	Probe 22 50ng	32,13	78,80	1817,87	5364,42
68	H12	SYBR	TEAD4	NRT	Probe 22		None	None	1,35
69	B07	SYBR	HPRT1 IDT	Unkn	Probe 21 50ng	32,50	73,20	2627,53	4283,77
70	B08	SYBR	PPIA	Unkn	Probe 21 50ng	27,89	80,00	1851,77	5602,84
71	C07	SYBR	HPRT1 IDT	Unkn	Probe 21 50ng	32,45	73,20	2715,47	4409,41
72	C08	SYBR	PPIA	Unkn	Probe 21 50ng	27,92	79,80	1884,22	5792,73
73	D07	SYBR	HPRT1 IDT	Unkn	Probe 21 50ng	33,52	73,20	2528,90	3676,96
74	D08	SYBR	PPIA	Unkn	Probe 21 50ng	27,86	79,80	1897,50	5832,01
75	E07	SYBR	HPRT1 IDT	Unkn	Probe 22 50ng	31,29	73,00	2810,39	5016,03
76	E08	SYBR	PPIA	Unkn	Probe 22 50ng	28,00	79,80	2123,53	6074,50
77	F07	SYBR	HPRT1 IDT	Unkn	Probe 22 50ng	31,43	73,00	2732,51	4889,28
78	F08	SYBR	PPIA	Unkn	Probe 22 50ng	27,70	79,80	2094,80	6030,67
79	G07	SYBR	HPRT1 IDT	Unkn	Probe 22 50ng	31,46	73,00	2662,30	4929,08
80	G08	SYBR	PPIA	Unkn	Probe 22 50ng	27,16	79,80	2055,57	6181,20
81	G11	SYBR	HPRT1 IDT	NTC	H20		None	None	-0,90
82	H08	SYBR	PPIA	NRT	Probe 22 50ng		None	None	0,23
83	H11	SYBR	PPIA	NTC	H20		None	None	0,82

	A	B	C	D	E	F	G	H	I
1	File Name	Sample 23 and 26 TC.pcrd							
2	Created By	admin							
3	Notes								
4	ID								
5	Run Started	07/28/2025 12:40:48 UTC							
6	Run Ended	07/28/2025 14:36:50 UTC							
7	Sample Vol	20							
8	Lid Temp	105							
9	Protocol Fil	Originalprotokoll mit Schmelzkurve ab 60 Grad.prcl							
10	Plate Setup	Plattenvorlage PhD Projekt Ansatz.pltd							
11	Base Serial	BR203528							
12	Optical Hea	787BR12390							
13	CFX Manag	3.1.3086.0516.							
14									
15	Well group	All Wells							
16	Amplificatio	4							
17	Melt step	6							
18									
19									
20	Well	Fluor	Target	Content	Sample	Cq	Melt Temperature	Peak Height	End RFU
21	A06	SYBR	TEAD4	Unkn	Probe 23 50ng	32,96	78,60	762,12	1830,64
22	A11	SYBR	YAP1	NTC	H20		None	None	42,97
23	A12	SYBR	TBP	NTC	H20		None	None	1,05
24	B01	SYBR	YAP1	Unkn	Probe 23 50ng	33,71	79,60	427,75	1121,02
25	B04	SYBR	MOB1A	Unkn	Probe 23 50ng	28,87	76,40	1204,55	2703,94
26	B06	SYBR	TEAD4	Unkn	Probe 23 50ng	32,03	78,60	846,26	2151,93
27	B09	SYBR	TBP	Unkn	Probe 23 50ng	35,00	77,80	641,12	1124,59
28	B10	SYBR	HPRT1 RT	Unkn	Probe 23 50ng	30,71	77,20	1004,54	2437,97
29	B12	SYBR	HPRT1 RT	NTC	H20		None	None	-0,13
30	C01	SYBR	YAP1	Unkn	Probe 23 50ng	33,84	79,80	438,07	1099,72
31	C04	SYBR	MOB1A	Unkn	Probe 23 50ng	29,00	76,40	1170,20	2626,50
32	C06	SYBR	TEAD4	Unkn	Probe 23 50ng	31,60	78,60	839,93	2386,64
33	C09	SYBR	TBP	Unkn	Probe 23 50ng	33,60	78,00	745,34	1527,42
34	C10	SYBR	HPRT1 RT	Unkn	Probe 23 50ng	30,69	77,20	981,75	2478,29
35	C11	SYBR	MST1	NTC	H20		None	None	-0,84
36	C12	SYBR	IC	Unkn	H20	24,53	80,80	2089,60	4824,77
37	D01	SYBR	YAP1	Unkn	Probe 23 50ng	33,52	79,80	489,55	1228,88
38	D04	SYBR	MOB1A	Unkn	Probe 23 50ng	28,63	76,40	1208,70	2826,37
39	D09	SYBR	TBP	Unkn	Probe 23 50ng	34,27	78,00	717,95	1348,80
40	D10	SYBR	HPRT1 RT	Unkn	Probe 23 50ng	30,73	77,20	980,89	2438,30
41	D11	SYBR	MOB1A	NTC	H20		None	None	0,05
42	D12	SYBR	IC	Unkn	H20	24,69	81,00	2007,70	4659,03
43	E01	SYBR	YAP1	Unkn	Probe 26 50ng	33,76	79,80	467,12	960,50
44	E03	SYBR	MST1	Unkn	Probe 26 50ng	32,27	75,40	1124,80	1748,35
45	E04	SYBR	MOB1A	Unkn	Probe 26 50ng	30,18	76,40	1125,04	1992,31
46	E06	SYBR	TEAD4	Unkn	Probe 26 50ng	32,55	78,60	788,01	1714,00
47	E09	SYBR	TBP	Unkn	Probe 26 50ng	34,29	78,00	767,48	1168,70
48	E10	SYBR	HPRT1 RT	Unkn	Probe 26 50ng	30,61	77,20	1017,98	2126,74
49	E12	SYBR	IC	Unkn	Probe 23	25,11	81,00	1998,89	4673,49
50	F01	SYBR	YAP1	Unkn	Probe 26 50ng	33,64	80,00	536,38	994,11
51	F03	SYBR	MST1	Unkn	Probe 26 50ng	32,35	75,40	999,70	1644,92
52	F04	SYBR	MOB1A	Unkn	Probe 26 50ng	30,07	76,40	1197,44	2170,45
53	F06	SYBR	TEAD4	Unkn	Probe 26 50ng	32,28	78,60	833,18	1874,72
54	F09	SYBR	TBP	Unkn	Probe 26 50ng	33,65	77,80	720,55	1346,68
55	F10	SYBR	HPRT1 RT	Unkn	Probe 26 50ng	30,58	77,20	994,95	2048,01

	A	B	C	D	E	F	G	H	I
56	F11	SYBR	TEAD4	NTC	H20		None	None	34,07
57	F12	SYBR	IC	Unkn	Probe 26	27,07	80,80	1842,38	3476,73
58	G01	SYBR	YAP1	Unkn	Probe 26 50ng	33,98	79,80	481,54	946,48
59	G03	SYBR	MST1	Unkn	Probe 26 50ng	32,23	75,40	1034,12	1667,01
60	G04	SYBR	MOB1A	Unkn	Probe 26 50ng	30,25	76,40	1206,34	2135,13
61	G09	SYBR	TBP	Unkn	Probe 26 50ng	34,12	77,80	717,41	1238,88
62	G10	SYBR	HPRT1 RT	Unkn	Probe 26 50ng	31,34	77,20	902,04	1811,89
63	G12	SYBR	TBP	NRT	Probe 23		None	None	1,40
64	H06	SYBR	TEAD4	Unkn	Probe 26 50ng	32,44	78,60	726,87	1777,81
65	H12	SYBR	HPRT1 RT	NRT	Probe 26		None	None	4,09
66	B07	SYBR	HPRT1 IDT	Unkn	Probe 23 50ng	34,21	73,00	1171,66	1269,63
67	B08	SYBR	PPIA	Unkn	Probe 23 50ng	27,30	79,60	873,43	2468,26
68	C07	SYBR	HPRT1 IDT	Unkn	Probe 23 50ng	34,24	72,80	1144,99	1283,65
69	C08	SYBR	PPIA	Unkn	Probe 23 50ng	27,48	79,60	877,00	2485,46
70	D07	SYBR	HPRT1 IDT	Unkn	Probe 23 50ng	34,61	72,80	1086,35	1197,24
71	D08	SYBR	PPIA	Unkn	Probe 23 50ng	27,14	79,60	882,20	2596,63
72	E07	SYBR	HPRT1 IDT	Unkn	Probe 26 50ng	33,09	73,00	1242,35	1333,96
73	E08	SYBR	PPIA	Unkn	Probe 26 50ng	27,94	79,60	915,50	2082,68
74	F07	SYBR	HPRT1 IDT	Unkn	Probe 26 50ng	33,18	72,80	1179,48	1299,97
75	F08	SYBR	PPIA	Unkn	Probe 26 50ng	28,00	79,60	845,45	1969,48
76	G07	SYBR	HPRT1 IDT	Unkn	Probe 26 50ng	33,37	73,00	1128,98	1279,76
77	G08	SYBR	PPIA	Unkn	Probe 26 50ng	27,94	79,60	849,94	1985,63
78	G11	SYBR	HPRT1 IDT	NTC	H20		None	None	1,25
79	H08	SYBR	PPIA	NRT	Probe 26 50ng	31,58	79,80	884,37	1517,89
80	H11	SYBR	PPIA	NTC	H20		None	None	1,92

	A	B	C	D	E	F	G	H	I
1	File Name	Sample 24 and 25 TC.pcrd							
2	Created By	admin							
3	Notes								
4	ID								
5	Run Started	07/21/2025 13:47:20 UTC							
6	Run Ended	07/21/2025 15:44:04 UTC							
7	Sample Vol	20							
8	Lid Temp	105							
9	Protocol Fil	Originalprotokoll mit Schmelzkurve ab 60 Grad.prcl							
10	Plate Setup	Plattenvorlage PhD Projekt Ansatz.pltd							
11	Base Serial	BR203528							
12	Optical Hea	787BR12390							
13	CFX Manag	3.1.3086.0516.							
14									
15	Well group	All Wells							
16	Amplificatio	4							
17	Melt step	6							
18									
19									
20	Well	Fluor	Target	Content	Sample	Cq	Melt Temperature	Peak Height	End RFU
21	A06	SYBR	TEAD4	Unkn	Probe 24 50ng	33,17	78,80	1938,09	4746,81
22	A11	SYBR	YAP1	NTC	H20		None	None	1,45
23	A12	SYBR	TBP	NTC	H20		None	None	0,87
24	B01	SYBR	YAP1	Unkn	Probe 24 50ng	33,71	79,60	1065,93	3224,63
25	B03	SYBR	MST1	Unkn	Probe 24 50ng	31,62	75,60	2659,76	5695,56
26	B04	SYBR	MOB1A	Unkn	Probe 24 50ng	29,00	76,40	2971,34	7313,61
27	B06	SYBR	TEAD4	Unkn	Probe 24 50ng	33,83	78,40	1473,44	3879,74
28	B09	SYBR	TBP	Unkn	Probe 24 50ng	33,30	77,80	1474,13	3998,14
29	B10	SYBR	HPRT1 RT	Unkn	Probe 24 50ng	29,74	77,20	2112,56	6266,97
30	B12	SYBR	HPRT1 RT	NTC	H20		None	None	0,35
31	C01	SYBR	YAP1	Unkn	Probe 24 50ng	34,02	80,00	1293,65	3293,16
32	C03	SYBR	MST1	Unkn	Probe 24 50ng	31,52	75,60	2741,47	6045,85
33	C04	SYBR	MOB1A	Unkn	Probe 24 50ng	29,17	76,40	2888,44	7145,74
34	C09	SYBR	TBP	Unkn	Probe 24 50ng	32,72	77,60	1495,44	4204,94
35	C10	SYBR	HPRT1 RT	Unkn	Probe 24 50ng	29,76	77,20	2055,59	6281,79
36	C11	SYBR	MST1	NTC	H20		None	None	1,00
37	C12	SYBR	IC	Unkn	H20	26,39	81,00	4793,21	11298,28
38	D01	SYBR	YAP1	Unkn	Probe 24 50ng	33,85	80,00	1235,26	3384,13
39	D03	SYBR	MST1	Unkn	Probe 24 50ng	31,67	75,60	2646,69	5947,24
40	D04	SYBR	MOB1A	Unkn	Probe 24 50ng	29,23	76,40	2862,43	7106,35
41	D06	SYBR	TEAD4	Unkn	Probe 24 50ng	33,42	78,40	1528,04	4257,39
42	D09	SYBR	TBP	Unkn	Probe 24 50ng	32,80	77,60	1443,36	4184,55
43	D10	SYBR	HPRT1 RT	Unkn	Probe 24 50ng	29,93	77,20	1956,64	5917,66
44	D11	SYBR	MOB1A	NTC	H20		None	None	-1,02
45	D12	SYBR	IC	Unkn	H20	26,47	81,00	4643,20	10898,87
46	E01	SYBR	YAP1	Unkn	Probe 25 50ng	34,57	79,80	1133,72	2958,67
47	E03	SYBR	MST1	Unkn	Probe 25 50ng	32,11	75,60	2469,41	5703,13
48	E04	SYBR	MOB1A	Unkn	Probe 25 50ng	29,75	76,20	2302,39	5724,56
49	E06	SYBR	TEAD4	Unkn	Probe 25 50ng	31,70	78,80	2177,06	6475,38
50	E09	SYBR	TBP	Unkn	Probe 25 50ng	34,48	78,20	1837,76	3825,53
51	E10	SYBR	HPRT1 RT	Unkn	Probe 25 50ng	30,45	77,40	2410,21	6916,16
52	E12	SYBR	IC	Unkn	Probe 24	27,17	81,00	4557,44	10647,09
53	F01	SYBR	YAP1	Unkn	Probe 25 50ng	35,70	80,00	986,10	2414,27
54	F03	SYBR	MST1	Unkn	Probe 25 50ng	32,53	75,60	2350,97	5370,89
55	F04	SYBR	MOB1A	Unkn	Probe 25 50ng	29,58	76,20	2315,43	5779,90

	A	B	C	D	E	F	G	H	I
56	F06	SYBR	TEAD4	Unkn	Probe 25 50ng	31,73	78,80	2051,36	6193,95
57	F09	SYBR	TBP	Unkn	Probe 25 50ng	34,14	78,00	1838,37	4279,62
58	F10	SYBR	HPRT1 RT	Unkn	Probe 25 50ng	30,50	77,40	2504,23	7157,01
59	F11	SYBR	TEAD4	NTC	H20	39,71	None	None	500,85
60	F12	SYBR	IC	Unkn	Probe 25	26,73	81,00	4481,44	10575,16
61	G01	SYBR	YAP1	Unkn	Probe 25 50ng	34,72	79,60	1085,63	3000,30
62	G03	SYBR	MST1	Unkn	Probe 25 50ng	32,57	75,60	2615,40	5504,62
63	G04	SYBR	MOB1A	Unkn	Probe 25 50ng	29,86	76,20	2139,17	5454,36
64	G06	SYBR	TEAD4	Unkn	Probe 25 50ng	31,59	78,80	2061,12	6416,86
65	G09	SYBR	TBP	Unkn	Probe 25 50ng	34,08	78,00	1830,53	4410,06
66	G10	SYBR	HPRT1 RT	Unkn	Probe 25 50ng	30,40	77,40	2401,52	6950,86
67	G12	SYBR	TEAD4	NRT	Probe 24		None	None	-0,71
68	B07	SYBR	HPRT1 IDT	Unkn	Probe 24 50ng	38,02	72,80	1632,48	1109,33
69	B08	SYBR	PPIA	Unkn	Probe 24 50ng	28,61	79,20	1604,81	5655,21
70	C07	SYBR	HPRT1 IDT	Unkn	Probe 24 50ng	37,46	72,80	1711,10	1342,87
71	C08	SYBR	PPIA	Unkn	Probe 24 50ng	28,36	79,20	1714,69	6053,95
72	D07	SYBR	HPRT1 IDT	Unkn	Probe 24 50ng	37,74	72,60	1561,80	1219,28
73	D08	SYBR	PPIA	Unkn	Probe 24 50ng	28,23	79,40	1652,29	5963,07
74	E07	SYBR	HPRT1 IDT	Unkn	Probe 25 50ng	32,98	73,20	3063,56	4889,79
75	E08	SYBR	PPIA	Unkn	Probe 25 50ng	28,57	79,80	2197,74	6776,33
76	F07	SYBR	HPRT1 IDT	Unkn	Probe 25 50ng	32,99	73,20	3015,07	4842,40
77	F08	SYBR	PPIA	Unkn	Probe 25 50ng	28,34	79,80	2055,43	6583,42
78	G07	SYBR	HPRT1 IDT	Unkn	Probe 25 50ng	32,60	73,20	2948,98	5140,17
79	G08	SYBR	PPIA	Unkn	Probe 25 50ng	28,31	79,80	2131,44	6808,53
80	G11	SYBR	HPRT1 IDT	NTC	H20		None	None	-1,96
81	H08	SYBR	PPIA	NRT	Probe 25 50ng		None	None	0,32
82	H11	SYBR	PPIA	NTC	H20		None	None	-0,38
83	H12	SYBR	HPRT1 IDT	NRT	Probe 25		None	None	0,52

	A	B	C	D	E	F	G	H	I
1	File Name	Repitition Sample 1 NG and 12 B2.pcrd							
2	Created By	admin							
3	Notes								
4	ID								
5	Run Started	08/05/2025 13:40:29 UTC							
6	Run Ended	08/05/2025 15:36:32 UTC							
7	Sample Vol	20							
8	Lid Temp	105							
9	Protocol Fil	Originalprotokoll mit Schmelzkurve ab 60 Grad.prcl							
10	Plate Setup	Plattenvorlage Wiederholungsplatte.pltd							
11	Base Serial	BR203528							
12	Optical Hea	787BR12390							
13	CFX Manag	3.1.3086.0516.							
14									
15	Well group	All Wells							
16	Amplificatio	4							
17	Melt step	6							
18									
19									
20	Well	Fluor	Target	Content	Sample	Cq	Melt Temperature	Peak Height	End RFU
21	B02	SYBR	MOB1A	Unkn	Pr. 1 50ng	25,96	76,20	1146,95	2728,44
22	B03	SYBR	TBP	Unkn	Pr. 1 50ng	29,15	78,00	848,42	2464,32
23	B04	SYBR	HPRT1 RT	Unkn	Pr. 1 50ng	27,09	77,40	1023,95	2959,98
24	B12	SYBR	MOB1A	NTC	H20		None	None	-0,13
25	C02	SYBR	MOB1A	Unkn	Pr. 1 50ng	26,09	76,40	1080,73	2582,83
26	C03	SYBR	TBP	Unkn	Pr. 1 50ng	29,34	78,00	761,76	2285,41
27	C04	SYBR	HPRT1 RT	Unkn	Pr. 1 50ng	27,17	77,40	1003,72	2849,12
28	C12	SYBR	TEAD4	NTC	H20		None	None	99,51
29	D02	SYBR	MOB1A	Unkn	Pr. 1 50ng	25,93	76,40	1091,33	2663,65
30	D03	SYBR	TBP	Unkn	Pr. 1 50ng	29,30	78,00	790,54	2384,62
31	D04	SYBR	HPRT1 RT	Unkn	Pr. 1 50ng	27,22	77,40	976,32	2854,10
32	D12	SYBR	YAP1	NTC	H20	39,32	None	None	227,64
33	E05	SYBR	TEAD4	Unkn	Pr. 12 50ng	31,13	78,80	761,21	2229,67
34	E06	SYBR	YAP1	Unkn	Pr. 12 50ng	29,56	80,00	589,27	1887,61
35	E07	SYBR	MOB1A	Unkn	Pr. 12 50ng	25,09	76,40	1278,93	3185,23
36	E09	SYBR	TBP	Unkn	Pr. 12 50ng	28,17	78,00	834,70	2575,02
37	E10	SYBR	HPRT1 RT	Unkn	Pr. 12 50ng	26,28	77,40	984,53	2917,76
38	F05	SYBR	TEAD4	Unkn	Pr. 12 50ng	31,00	78,60	729,76	2283,46
39	F06	SYBR	YAP1	Unkn	Pr. 12 50ng	29,45	80,00	575,80	1828,04
40	F07	SYBR	MOB1A	Unkn	Pr. 12 50ng	25,12	76,40	1171,28	2982,35
41	F09	SYBR	TBP	Unkn	Pr. 12 50ng	28,16	78,00	764,68	2433,51
42	F10	SYBR	HPRT1 RT	Unkn	Pr. 12 50ng	26,31	77,40	897,12	2697,74
43	F12	SYBR	TBP	NTC	H20		None	None	1,39
44	G06	SYBR	YAP1	Unkn	Pr. 12 50ng	29,95	80,00	543,76	1766,49
45	G07	SYBR	MOB1A	Unkn	Pr. 12 50ng	25,12	76,40	1165,54	2988,86
46	G09	SYBR	TBP	Unkn	Pr. 12 50ng	28,06	78,00	759,02	2513,76
47	G10	SYBR	HPRT1 RT	Unkn	Pr. 12 50ng	26,45	77,40	856,32	2591,42
48	G12	SYBR	HPRT1 RT	NTC	H20		None	None	-0,14
49	H05	SYBR	TEAD4	Unkn	Pr. 12 50ng	31,02	78,80	648,46	2096,92

	A	B	C	D	E	F	G	H	I
1	File Name	Repetition Sample 14.pcrd							
2	Created By	admin							
3	Notes								
4	ID								
5	Run Started	08/08/2025 12:38:49 UTC							
6	Run Ended	08/08/2025 14:34:57 UTC							
7	Sample Vol	20							
8	Lid Temp	105							
9	Protocol Fil	Originalprotokoll mit Schmelzkurve ab 60 Grad.prcl							
10	Plate Setup	Plattenvorlage Wiederholungsplatte.pltd							
11	Base Serial	BR203528							
12	Optical Hea	787BR12390							
13	CFX Manag	3.1.3086.0516.							
14									
15	Well group	All Wells							
16	Amplificatio	4							
17	Melt step	6							
18									
19									
20	Well	Fluor	Target	Content	Sample	Cq	Melt Temperature	Peak Height	End RFU
21	A08	SYBR	YAP1	NTC	H20		None	None	243,54
22	A09	SYBR	TBP	NTC	H20		None	None	-2,36
23	A10	SYBR	HPRT1 RT	NTC	H20		None	None	-1,5797059
24	B05	SYBR	YAP1	Unkn	Pr. 14 50ng	30,79	80,20	1147,67	3279,60
25	B07	SYBR	TBP	Unkn	Pr. 14 50ng	31,07	78,40	1508,92	3864,73
26	B08	SYBR	HPRT1 RT	Unkn	Pr. 14 50ng	27,32	77,80	1599,52	4770,45
27	C05	SYBR	YAP1	Unkn	Pr. 14 50ng	30,69	80,40	1176,55	3523,40
28	C07	SYBR	TBP	Unkn	Pr. 14 50ng	30,91	78,40	1535,12	4002,85
29	C08	SYBR	HPRT1 RT	Unkn	Pr. 14 50ng	27,31	77,80	1543,95	4741,67
30	D05	SYBR	YAP1	Unkn	Pr. 14 50ng	30,80	80,40	1137,41	3462,44
31	D07	SYBR	TBP	Unkn	Pr. 14 50ng	30,91	78,40	1483,11	3892,74
32	D08	SYBR	HPRT1 RT	Unkn	Pr. 14 50ng	27,24	77,80	1570,11	4782,94
33	F12	SYBR	IC	Unkn	H20	23,88	80,80	1717,93	4040,22
34	G12	SYBR	IC	Unkn	H20	23,81	80,80	1760,49	4089,42
35	H12	SYBR	IC	Unkn	Pr. 14 50ng	24,67	80,80	1554,57	3594,26

	A	B	C	D	E	F	G	H	I
1	File Name	LATS1, SAV1 Sample 1, 2, 3, 5..pcrd							
2	Created By	admin							
3	Notes								
4	ID								
5	Run Started	09/30/2025 14:33:15 UTC							
6	Run Ended	09/30/2025 16:29:22 UTC							
7	Sample Vol	20							
8	Lid Temp	105							
9	Protocol Fil	Originalprotokoll mit Schmelzkurve ab 60 Grad.prcl							
10	Plate Setup	Plattenvorlage Wiederholungsplatte IDT Primer neu.pltd							
11	Base Serial	BR203528							
12	Optical Hea	787BR12390							
13	CFX Manag	3.1.3086.0516.							
14									
15	Well group	All Wells							
16	Amplificatio	4							
17	Melt step	6							
18									
19									
20	Well	Fluor	Target	Content	Sample	Cq	Melt Temperature	Peak Height	End RFU
21	A02	SYBR	LATS1	Unkn	Pr. 5 50ng	30,18	75,60	2382,14	4885,26
22	A03	SYBR	SAV1	Unkn	Pr. 5 50ng	27,59	76,20	1776,86	4856,56
23	A04	SYBR	HPRT1 RT	Unkn	Pr. 5 50ng	33,52	77,80	1541,00	3262,74
24	A05	SYBR	TBP	Unkn	Pr. 5 50ng	31,83	78,40	1630,32	4058,76
25	B02	SYBR	LATS1	Unkn	Pr. 1 50ng	29,11	75,60	2183,54	4871,02
26	B03	SYBR	SAV1	Unkn	Pr. 1 50ng	29,77	76,00	1800,19	4771,79
27	B04	SYBR	HPRT1 RT	Unkn	Pr. 1 50ng	30,50	77,80	1587,65	4309,95
28	B05	SYBR	TBP	Unkn	Pr. 1 50ng	29,92	78,40	1678,18	4754,21
29	B07	SYBR	LATS1	Unkn	Pr. 2 50ng	29,28	75,60	2416,63	5340,57
30	B08	SYBR	SAV1	Unkn	Pr. 2 50ng	29,44	76,20	1904,65	5041,68
31	B09	SYBR	HPRT1 RT	Unkn	Pr. 2 50ng	31,95	77,80	1655,31	4094,18
32	B10	SYBR	TBP	Unkn	Pr. 2 50ng	30,67	78,60	1777,02	4776,76
33	B12	SYBR	LATS1	NTC	H20		None	None	-0,70
34	C02	SYBR	LATS1	Unkn	Pr. 1 50ng	37,47	75,60	1280,47	1391,86
35	C03	SYBR	SAV1	Unkn	Pr. 1 50ng	29,14	76,20	1698,83	4805,06
36	C04	SYBR	HPRT1 RT	Unkn	Pr. 1 50ng	30,33	77,80	1641,21	4639,40
37	C05	SYBR	TBP	Unkn	Pr. 1 50ng	30,39	78,40	1656,03	4640,71
38	C07	SYBR	LATS1	Unkn	Pr. 2 50ng	31,23	75,60	2172,10	4341,41
39	C08	SYBR	SAV1	Unkn	Pr. 2 50ng	29,40	76,20	1880,28	5150,12
40	C09	SYBR	HPRT1 RT	Unkn	Pr. 2 50ng	31,64	77,80	1613,27	4181,18
41	C10	SYBR	TBP	Unkn	Pr. 2 50ng	30,61	78,40	1744,73	4936,56
42	C12	SYBR	SAV1	NTC	H20		None	None	1,31
43	D02	SYBR	LATS1	Unkn	Pr. 1 50ng	28,80	75,60	2289,47	5311,82
44	D03	SYBR	SAV1	Unkn	Pr. 1 50ng	29,23	76,20	1819,28	5096,67
45	D04	SYBR	HPRT1 RT	Unkn	Pr. 1 50ng	30,24	77,80	1621,96	4601,97
46	D05	SYBR	TBP	Unkn	Pr. 1 50ng	30,00	78,40	1648,91	4838,34
47	D07	SYBR	LATS1	Unkn	Pr. 2 50ng	29,30	75,60	2232,18	5064,03
48	D08	SYBR	SAV1	Unkn	Pr. 2 50ng	29,44	76,20	1859,64	5138,03
49	D09	SYBR	HPRT1 RT	Unkn	Pr. 2 50ng	31,76	77,80	1548,41	4085,25
50	D10	SYBR	TBP	Unkn	Pr. 2 50ng	35,39	78,40	1371,73	2536,43
51	D12	SYBR	HPRT1 RT	NTC	H20		None	None	-0,15
52	E02	SYBR	LATS1	Unkn	Pr. 3 50ng	30,61	75,60	2208,08	4811,55
53	E03	SYBR	SAV1	Unkn	Pr. 3 50ng	30,32	76,20	1643,87	4537,61
54	E04	SYBR	HPRT1 RT	Unkn	Pr. 3 50ng	31,61	77,80	1628,22	4383,30
55	E05	SYBR	TBP	Unkn	Pr. 3 50ng	31,53	78,40	1632,79	4529,33

	A	B	C	D	E	F	G	H	I
56	E12	SYBR	TBP	NTC	H20		None	None	-0,46
57	F02	SYBR	LATS1	Unkn	Pr. 3 50ng	30,52	75,60	2226,90	4817,02
58	F03	SYBR	SAV1	Unkn	Pr. 3 50ng	30,12	76,20	1706,89	4824,65
59	F04	SYBR	HPRT1 RT	Unkn	Pr. 3 50ng	31,63	77,80	1533,90	4244,61
60	F05	SYBR	TBP	Unkn	Pr. 3 50ng	31,48	78,40	1526,49	4422,02
61	G02	SYBR	LATS1	Unkn	Pr. 3 50ng	30,54	75,60	2201,52	4926,57
62	G03	SYBR	SAV1	Unkn	Pr. 3 50ng	30,20	76,20	1690,92	4774,91
63	G04	SYBR	HPRT1 RT	Unkn	Pr. 3 50ng	31,54	77,80	1538,24	4267,58
64	G05	SYBR	TBP	Unkn	Pr. 3 50ng	31,23	78,40	1564,13	4542,39
65	G11	SYBR	HPRT1 RT	NRT	Probe 3 50ng		None	None	0,47
66	G12	SYBR	LATS1	NRT	Pr. 2 50ng		None	None	-0,01
67	H02	SYBR	LATS1	Unkn	Pr. 5 50ng	30,37	75,60	2118,78	4732,36
68	H03	SYBR	SAV1	Unkn	Pr. 5 50ng	27,11	76,20	1739,09	5222,12
69	H04	SYBR	HPRT1 RT	Unkn	Pr. 5 50ng	32,88	77,80	1494,64	3731,18
70	H05	SYBR	TBP	Unkn	Pr. 5 50ng	31,31	78,60	1677,66	4474,92
71	H07	SYBR	LATS1	Unkn	Pr. 5 50ng	30,02	75,60	2301,41	5246,00
72	H08	SYBR	SAV1	Unkn	Pr. 5 50ng	29,65	76,00	1792,60	5106,20
73	H09	SYBR	HPRT1 RT	Unkn	Pr. 5 50ng		None	None	2,57
74	H10	SYBR	TBP	Unkn	Pr. 5 50ng	31,23	78,40	1593,84	4523,03
75	H12	SYBR	SAV1	NRT	Pr. 5 50ng		None	None	0,95

	A	B	C	D	E	F	G	H	I
1	File Name	LATS1, SAV1 Sample 4, 6 to 9.pcrd							
2	Created By	admin							
3	Notes								
4	ID								
5	Run Started	10/01/2025 14:17:42 UTC							
6	Run Ended	10/01/2025 16:13:38 UTC							
7	Sample Vol	20							
8	Lid Temp	105							
9	Protocol Fil	Originalprotokoll mit Schmelzkurve ab 60 Grad.prcl							
10	Plate Setup	Plattenvorlage Wiederholungsplatte IDT Primer neu.pltd							
11	Base Serial	BR203528							
12	Optical Hea	787BR12390							
13	CFX Manag	3.1.3086.0516.							
14									
15	Well group	All Wells							
16	Amplificatio	4							
17	Melt step	6							
18									
19									
20	Well	Fluor	Target	Content	Sample	Cq	Melt Temperature	Peak Height	End RFU
21	A02	SYBR	LATS1	Unkn	Pr. 9 50ng	28,31	75,20	1972,39	3649,51
22	A03	SYBR	SAV1	Unkn	Pr. 9 50ng	28,23	75,60	1381,65	3378,04
23	A04	SYBR	HPRT1 RT	Unkn	Pr. 9 50ng	29,59	77,40	979,31	2713,15
24	A05	SYBR	TBP	Unkn	Pr. 9 50ng	29,40	78,00	1138,58	3235,99
25	B02	SYBR	LATS1	Unkn	Pr. 4 50ng	28,36	75,60	2355,74	5240,98
26	B03	SYBR	SAV1	Unkn	Pr. 4 50ng	27,18	76,20	1945,59	5464,65
27	B04	SYBR	HPRT1 RT	Unkn	Pr. 4 50ng	29,77	77,80	1639,86	4539,87
28	B05	SYBR	TBP	Unkn	Pr. 4 50ng	29,16	78,40	1750,45	5089,54
29	B07	SYBR	LATS1	Unkn	Pr. 6 50ng	28,38	75,60	2577,90	5668,68
30	B08	SYBR	SAV1	Unkn	Pr. 6 50ng	26,36	76,20	2118,15	5886,51
31	B09	SYBR	HPRT1 RT	Unkn	Pr. 6 50ng	30,09	77,80	1779,01	4685,31
32	B10	SYBR	TBP	Unkn	Pr. 6 50ng	29,37	78,60	1882,91	5143,01
33	B12	SYBR	LATS1	NTC	H20		None	None	1,03
34	C02	SYBR	LATS1	Unkn	Pr. 4 50ng	28,19	75,60	2472,08	5573,96
35	C03	SYBR	SAV1	Unkn	Pr. 4 50ng	32,48	76,00	1819,37	3967,40
36	C04	SYBR	HPRT1 RT	Unkn	Pr. 4 50ng	29,66	77,80	1673,59	4742,19
37	C05	SYBR	TBP	Unkn	Pr. 4 50ng	29,05	78,40	1737,40	5168,98
38	C07	SYBR	LATS1	Unkn	Pr. 6 50ng	32,22	75,60	2437,89	4301,73
39	C08	SYBR	SAV1	Unkn	Pr. 6 50ng	26,05	76,20	2154,74	6091,23
40	C09	SYBR	HPRT1 RT	Unkn	Pr. 6 50ng	30,23	77,80	1738,86	4677,83
41	C10	SYBR	TBP	Unkn	Pr. 6 50ng	29,20	78,60	1898,14	5344,55
42	C12	SYBR	SAV1	NTC	H20		None	None	0,01
43	D02	SYBR	LATS1	Unkn	Pr. 4 50ng	28,13	75,60	2499,73	5705,29
44	D03	SYBR	SAV1	Unkn	Pr. 4 50ng	26,73	76,20	2011,19	5798,31
45	D04	SYBR	HPRT1 RT	Unkn	Pr. 4 50ng	30,01	77,80	1611,87	4608,67
46	D05	SYBR	TBP	Unkn	Pr. 4 50ng	29,12	78,40	1714,31	5131,99
47	D07	SYBR	LATS1	Unkn	Pr. 6 50ng	28,40	75,60	2530,02	5702,67
48	D08	SYBR	SAV1	Unkn	Pr. 6 50ng	26,22	76,20	2064,40	6025,22
49	D09	SYBR	HPRT1 RT	Unkn	Pr. 6 50ng	29,83	77,80	1656,11	4640,11
50	D10	SYBR	TBP	Unkn	Pr. 6 50ng	29,03	78,60	1837,15	5264,96
51	D12	SYBR	HPRT1 RT	NTC	H20		None	None	0,82
52	E02	SYBR	LATS1	Unkn	Pr. 7 50ng	28,65	75,20	1956,42	3877,81
53	E03	SYBR	SAV1	Unkn	Pr. 7 50ng	28,04	75,60	1427,05	3823,41
54	E04	SYBR	HPRT1 RT	Unkn	Pr. 7 50ng	28,78	77,40	1042,16	3147,03
55	E05	SYBR	TBP	Unkn	Pr. 7 50ng	28,69	78,00	1090,10	3460,84

	A	B	C	D	E	F	G	H	I
56	E07	SYBR	LATS1	Unkn	Pr. 8 50ng	31,19	75,20	1813,31	3190,82
57	E08	SYBR	SAV1	Unkn	Pr. 8 50ng	29,41	75,60	1449,50	3679,40
58	E09	SYBR	HPRT1 RT	Unkn	Pr. 8 50ng	31,63	77,20	1081,88	2704,15
59	E10	SYBR	TBP	Unkn	Pr. 8 50ng	31,18	78,00	1086,05	3093,82
60	E12	SYBR	TBP	NTC	H20		None	None	-0,91
61	F02	SYBR	LATS1	Unkn	Pr. 7 50ng	27,67	75,20	1891,69	4023,96
62	F03	SYBR	SAV1	Unkn	Pr. 7 50ng	28,10	75,60	1386,67	3780,35
63	F04	SYBR	HPRT1 RT	Unkn	Pr. 7 50ng	28,78	77,40	984,35	3086,41
64	F05	SYBR	TBP	Unkn	Pr. 7 50ng	28,39	78,00	1142,89	3717,74
65	F07	SYBR	LATS1	Unkn	Pr. 8 50ng	30,84	75,20	1839,40	3464,41
66	F08	SYBR	SAV1	Unkn	Pr. 8 50ng	29,47	75,60	1408,57	3561,34
67	F09	SYBR	HPRT1 RT	Unkn	Pr. 8 50ng	31,52	77,20	986,05	2633,89
68	F10	SYBR	TBP	Unkn	Pr. 8 50ng		None	None	43,07
69	F11	SYBR	SAV1	NRT	Pr. 9 50ng		None	None	-1,55
70	G02	SYBR	LATS1	Unkn	Pr. 7 50ng	27,62	75,20	2001,98	4234,38
71	G03	SYBR	SAV1	Unkn	Pr. 7 50ng	27,80	75,80	1398,10	3827,51
72	G04	SYBR	HPRT1 RT	Unkn	Pr. 7 50ng	29,05	77,40	990,34	3067,39
73	G05	SYBR	TBP	Unkn	Pr. 7 50ng	28,30	78,00	1106,22	3693,70
74	G07	SYBR	LATS1	Unkn	Pr. 8 50ng	31,26	75,20	1704,83	3185,34
75	G08	SYBR	SAV1	Unkn	Pr. 8 50ng	29,88	75,60	1421,68	3559,92
76	G09	SYBR	HPRT1 RT	Unkn	Pr. 8 50ng	31,46	77,20	985,79	2651,21
77	G10	SYBR	TBP	Unkn	Pr. 8 50ng	31,25	78,00	933,38	2810,94
78	G11	SYBR	HPRT1 RT	NRT	Probe 9 50ng		None	None	1,17
79	G12	SYBR	LATS1	NRT	Pr. 6 50ng		None	None	1,82
80	H02	SYBR	LATS1	Unkn	Pr. 9 50ng	27,84	75,20	1906,11	4000,72
81	H03	SYBR	SAV1	Unkn	Pr. 9 50ng	27,76	75,60	1416,47	3993,80
82	H04	SYBR	HPRT1 RT	Unkn	Pr. 9 50ng	28,80	77,40	976,17	3122,20
83	H05	SYBR	TBP	Unkn	Pr. 9 50ng	28,84	78,00	1093,13	3484,25
84	H07	SYBR	LATS1	Unkn	Pr. 9 50ng	27,77	75,20	2051,38	4308,07
85	H08	SYBR	SAV1	Unkn	Pr. 9 50ng	27,74	75,60	1456,77	3977,96
86	H09	SYBR	HPRT1 RT	Unkn	Pr. 9 50ng	30,34	77,20	1060,11	2879,78
87	H10	SYBR	TBP	Unkn	Pr. 9 50ng	28,92	78,00	1096,83	3467,39
88	H11	SYBR	TBP	NRT	Probe 8 50ng		None	None	1,60
89	H12	SYBR	SAV1	NRT	Pr. 7 50ng		None	None	1,95

	A	B	C	D	E	F	G	H	I
1	File Name	LATS1, SAV1 Sample 11 to 15.pcrd							
2	Created By	admin							
3	Notes								
4	ID								
5	Run Started	10/06/2025 10:58:53 UTC							
6	Run Ended	10/06/2025 12:54:50 UTC							
7	Sample Vol	20							
8	Lid Temp	105							
9	Protocol Fil	Originalprotokoll mit Schmelzkurve ab 60 Grad.prcl							
10	Plate Setup	Plattenvorlage Wiederholungsplatte IDT Primer neu.pltd							
11	Base Serial	BR203528							
12	Optical Hea	787BR12390							
13	CFX Manag	3.1.3086.0516.							
14									
15	Well group	All Wells							
16	Amplificatio	4							
17	Melt step	6							
18									
19									
20	Well	Fluor	Target	Content	Sample	Cq	Melt Temperature	Peak Height	End RFU
21	A02	SYBR	LATS1	Unkn	Pr. 11 50ng	30,52	75,20	1322,94	2310,41
22	A03	SYBR	SAV1	Unkn	Pr. 11 50ng	30,35	75,60	923,63	2135,39
23	A04	SYBR	HPRT1 RT	Unkn	Pr. 11 50ng	30,47	77,40	717,12	1888,10
24	A05	SYBR	TBP	Unkn	Pr. 11 50ng	31,26	78,00	683,71	1969,59
25	A08	SYBR	SAV1	Unkn	Pr. 13 50ng	30,08	75,40	1053,28	2229,14
26	B02	SYBR	LATS1	Unkn	Pr. 12 50ng	27,40	75,20	1551,20	3094,48
27	B03	SYBR	SAV1	Unkn	Pr. 12 50ng	27,19	75,60	1149,93	2960,37
28	B04	SYBR	HPRT1 RT	Unkn	Pr. 12 50ng	28,78	77,40	798,22	2317,58
29	B05	SYBR	TBP	Unkn	Pr. 12 50ng	28,44	78,00	889,40	2753,05
30	B07	SYBR	LATS1	Unkn	Pr. 13 50ng	31,58	75,20	1443,76	2270,42
31	B08	SYBR	SAV1	Unkn	Pr. 13 50ng	29,82	75,40	1096,58	2440,55
32	B09	SYBR	HPRT1 RT	Unkn	Pr. 13 50ng	32,36	77,20	779,12	1696,39
33	B10	SYBR	TBP	Unkn	Pr. 13 50ng	31,95	78,00	816,28	1986,66
34	B12	SYBR	LATS1	NTC	H20		None	None	2,31
35	C02	SYBR	LATS1	Unkn	Pr. 12 50ng	27,34	75,20	1611,76	3243,76
36	C03	SYBR	SAV1	Unkn	Pr. 12 50ng	27,09	75,60	1186,70	3143,40
37	C04	SYBR	HPRT1 RT	Unkn	Pr. 12 50ng	28,79	77,40	816,82	2436,50
38	C05	SYBR	TBP	Unkn	Pr. 12 50ng	28,31	78,00	870,71	2783,82
39	C07	SYBR	LATS1	Unkn	Pr. 13 50ng	31,32	75,20	1494,98	2409,29
40	C08	SYBR	SAV1	Unkn	Pr. 13 50ng	30,30	75,60	1133,45	2630,19
41	C09	SYBR	HPRT1 RT	Unkn	Pr. 13 50ng	32,50	77,20	801,78	1727,14
42	C10	SYBR	TBP	Unkn	Pr. 13 50ng	32,19	78,00	822,78	1988,75
43	C12	SYBR	SAV1	NTC	H20		None	None	0,26
44	D02	SYBR	LATS1	Unkn	Pr. 12 50ng	27,35	75,20	1550,17	3230,68
45	D03	SYBR	SAV1	Unkn	Pr. 12 50ng	27,76	75,60	1159,54	2990,73
46	D04	SYBR	HPRT1 RT	Unkn	Pr. 12 50ng	28,64	77,40	790,37	2446,19
47	D05	SYBR	TBP	Unkn	Pr. 12 50ng	28,45	78,00	829,28	2662,70
48	D07	SYBR	LATS1	Unkn	Pr. 13 50ng	31,59	75,20	1408,29	2289,71
49	D08	SYBR	SAV1	Unkn	Pr. 13 50ng	30,68	75,60	1114,02	2473,72
50	D09	SYBR	HPRT1 RT	Unkn	Pr. 13 50ng	32,05	77,20	771,22	1790,26
51	D10	SYBR	TBP	Unkn	Pr. 13 50ng	32,10	78,00	837,53	2016,49
52	D12	SYBR	HPRT1 RT	NTC	H20		None	None	1,81
53	E02	SYBR	LATS1	Unkn	Pr. 14 50ng	30,19	75,20	1430,36	2578,86
54	E03	SYBR	SAV1	Unkn	Pr. 14 50ng	28,46	75,60	1158,10	2963,81
55	E04	SYBR	HPRT1 RT	Unkn	Pr. 14 50ng	29,84	77,40	828,65	2263,23

	A	B	C	D	E	F	G	H	I
56	E05	SYBR	TBP	Unkn	Pr. 14 50ng	31,18	78,00	860,60	2264,39
57	E07	SYBR	LATS1	Unkn	Pr. 15 50ng	31,21	75,20	1324,13	2416,04
58	E08	SYBR	SAV1	Unkn	Pr. 15 50ng	29,21	75,60	1137,50	2925,93
59	E09	SYBR	HPRT1 RT	Unkn	Pr. 15 50ng	33,26	77,20	794,67	1644,78
60	E10	SYBR	TBP	Unkn	Pr. 15 50ng	32,31	77,80	756,71	1979,85
61	E12	SYBR	TBP	NTC	H20		None	None	0,33
62	F02	SYBR	LATS1	Unkn	Pr. 14 50ng	30,14	75,20	1428,83	2673,05
63	F03	SYBR	SAV1	Unkn	Pr. 14 50ng	28,36	75,60	1124,72	2910,77
64	F04	SYBR	HPRT1 RT	Unkn	Pr. 14 50ng	29,65	77,40	790,01	2295,50
65	F05	SYBR	TBP	Unkn	Pr. 14 50ng	30,71	78,00	848,88	2381,86
66	F07	SYBR	LATS1	Unkn	Pr. 15 50ng	31,17	75,20	1422,88	2546,12
67	F08	SYBR	SAV1	Unkn	Pr. 15 50ng	29,40	75,60	1085,04	2784,78
68	F09	SYBR	HPRT1 RT	Unkn	Pr. 15 50ng	32,64	77,20	714,76	1707,55
69	F10	SYBR	TBP	Unkn	Pr. 15 50ng	32,15	78,00	748,78	2019,89
70	F11	SYBR	SAV1	NRT	Pr. 14 50ng		None	None	-0,57
71	G02	SYBR	LATS1	Unkn	Pr. 14 50ng	32,68	75,20	1353,22	2081,99
72	G03	SYBR	SAV1	Unkn	Pr. 14 50ng	28,38	75,60	1101,15	2867,74
73	G04	SYBR	HPRT1 RT	Unkn	Pr. 14 50ng	29,85	77,40	793,01	2235,66
74	G05	SYBR	TBP	Unkn	Pr. 14 50ng	34,08	78,00	717,89	1518,50
75	G07	SYBR	LATS1	Unkn	Pr. 15 50ng	31,11	75,00	1343,23	2557,33
76	G08	SYBR	SAV1	Unkn	Pr. 15 50ng	29,30	75,60	1079,08	2780,90
77	G09	SYBR	HPRT1 RT	Unkn	Pr. 15 50ng	32,90	77,20	681,70	1678,86
78	G10	SYBR	TBP	Unkn	Pr. 15 50ng	32,33	78,00	737,41	1976,50
79	G11	SYBR	HPRT1 RT	NRT	Probe 15 50ng		None	None	0,80
80	G12	SYBR	LATS1	NRT	Pr. 13 50ng		None	None	0,89
81	H02	SYBR	LATS1	Unkn	Pr. 11 50ng	30,35	75,20	1296,31	2553,24
82	H03	SYBR	SAV1	Unkn	Pr. 11 50ng	29,88	75,60	976,49	2637,37
83	H04	SYBR	HPRT1 RT	Unkn	Pr. 11 50ng	29,90	77,40	700,32	2159,90
84	H05	SYBR	TBP	Unkn	Pr. 11 50ng	30,59	78,00	701,80	2317,81
85	H07	SYBR	LATS1	Unkn	Pr. 11 50ng	29,88	75,20	1370,98	2792,40
86	H08	SYBR	SAV1	Unkn	Pr. 11 50ng	29,74	75,60	956,31	2537,28
87	H09	SYBR	HPRT1 RT	Unkn	Pr. 11 50ng	29,58	77,20	701,58	2184,77
88	H10	SYBR	TBP	Unkn	Pr. 11 50ng	30,53	78,00	715,84	2342,53
89	H11	SYBR	TBP	NRT	Probe 15 50ng		None	None	1,73
90	H12	SYBR	SAV1	NRT	Pr. 12 50ng		None	None	2,50

	A	B	C	D	E	F	G	H	I
1	File Name	LATS1, SAV1 Sample 16 to 20.pcrd							
2	Created By	admin							
3	Notes								
4	ID								
5	Run Started	10/06/2025 13:25:08 UTC							
6	Run Ended	10/06/2025 15:20:46 UTC							
7	Sample Vol	20							
8	Lid Temp	105							
9	Protocol Fil	Originalprotokoll mit Schmelzkurve ab 60 Grad.prcl							
10	Plate Setup	Plattenvorlage Wiederholungsplatte IDT Primer neu.pltd							
11	Base Serial	BR203528							
12	Optical Hea	787BR12390							
13	CFX Manag	3.1.3086.0516.							
14									
15	Well group	All Wells							
16	Amplificatio	4							
17	Melt step	6							
18									
19									
20	Well	Fluor	Target	Content	Sample	Cq	Melt Temperature	Peak Height	End RFU
21	A02	SYBR	LATS1	Unkn	Pr. 16 50ng	31,58	75,00	1744,95	2858,04
22	A03	SYBR	SAV1	Unkn	Pr. 16 50ng	30,93	75,60	1234,34	2786,19
23	A04	SYBR	HPRT1 RT	Unkn	Pr. 16 50ng	32,49	77,20	914,83	2186,27
24	A05	SYBR	TBP	Unkn	Pr. 16 50ng	35,01	77,80	782,60	1686,74
25	B02	SYBR	LATS1	Unkn	Pr. 17 50ng	29,43	75,60	2199,69	4845,99
26	B03	SYBR	SAV1	Unkn	Pr. 17 50ng	27,80	76,20	1841,47	5120,65
27	B04	SYBR	HPRT1 RT	Unkn	Pr. 17 50ng	30,24	77,80	1569,67	4324,99
28	B05	SYBR	TBP	Unkn	Pr. 17 50ng	30,48	78,40	1638,15	4479,67
29	B07	SYBR	LATS1	Unkn	Pr. 18 50ng	27,16	75,60	2638,13	6025,89
30	B08	SYBR	SAV1	Unkn	Pr. 18 50ng	25,44	76,20	2077,78	5959,38
31	B09	SYBR	HPRT1 RT	Unkn	Pr. 18 50ng	29,11	77,80	1654,38	4690,61
32	B10	SYBR	TBP	Unkn	Pr. 18 50ng	28,42	78,40	1856,55	5367,95
33	B12	SYBR	LATS1	NTC	H20		None	None	2,20
34	C02	SYBR	LATS1	Unkn	Pr. 17 50ng	29,36	75,60	2326,38	5080,52
35	C03	SYBR	SAV1	Unkn	Pr. 17 50ng	27,90	76,20	1772,02	5024,89
36	C04	SYBR	HPRT1 RT	Unkn	Pr. 17 50ng	30,19	77,80	1641,27	4564,75
37	C05	SYBR	TBP	Unkn	Pr. 17 50ng	31,01	78,40	1671,57	4508,60
38	C07	SYBR	LATS1	Unkn	Pr. 18 50ng	27,02	75,60	2426,70	5687,17
39	C08	SYBR	SAV1	Unkn	Pr. 18 50ng	25,63	76,20	1892,88	5609,38
40	C09	SYBR	HPRT1 RT	Unkn	Pr. 18 50ng	29,24	77,80	1675,73	4842,70
41	C10	SYBR	TBP	Unkn	Pr. 18 50ng	28,25	78,60	1846,66	5475,84
42	C12	SYBR	SAV1	NTC	H20		None	None	0,85
43	D02	SYBR	LATS1	Unkn	Pr. 17 50ng	29,25	75,60	2358,10	5299,58
44	D03	SYBR	SAV1	Unkn	Pr. 17 50ng	27,79	76,20	1978,17	5581,38
45	D04	SYBR	HPRT1 RT	Unkn	Pr. 17 50ng	30,06	77,80	1507,19	4311,88
46	D05	SYBR	TBP	Unkn	Pr. 17 50ng	30,63	78,40	1585,54	4526,72
47	D07	SYBR	LATS1	Unkn	Pr. 18 50ng	26,94	75,60	2346,64	5610,50
48	D08	SYBR	SAV1	Unkn	Pr. 18 50ng	25,67	76,20	1895,05	5431,59
49	D09	SYBR	HPRT1 RT	Unkn	Pr. 18 50ng	29,82	77,80	1647,68	4663,70
50	D10	SYBR	TBP	Unkn	Pr. 18 50ng	28,16	78,60	1718,75	5130,71
51	D12	SYBR	HPRT1 RT	NTC	H20		None	None	1,31
52	E02	SYBR	LATS1	Unkn	Pr. 19 50ng	30,30	75,60	2217,21	4686,56
53	E03	SYBR	SAV1	Unkn	Pr. 19 50ng	27,15	76,20	1888,68	5422,31
54	E04	SYBR	HPRT1 RT	Unkn	Pr. 19 50ng	29,92	77,80	1666,79	4657,02
55	E05	SYBR	TBP	Unkn	Pr. 19 50ng	31,02	78,40	1617,26	4352,36

	A	B	C	D	E	F	G	H	I
56	E07	SYBR	LATS1	Unkn	Pr. 20 50ng	29,77	75,60	2380,62	5240,26
57	E08	SYBR	SAV1	Unkn	Pr. 20 50ng	27,21	76,00	1937,69	5696,08
58	E09	SYBR	HPRT1 RT	Unkn	Pr. 20 50ng	32,11	77,80	1628,11	4150,19
59	E10	SYBR	TBP	Unkn	Pr. 20 50ng	30,68	78,40	1665,36	4688,21
60	E12	SYBR	TBP	NTC	H20		None	None	1,20
61	F02	SYBR	LATS1	Unkn	Pr. 19 50ng	29,97	75,60	2252,17	4897,88
62	F03	SYBR	SAV1	Unkn	Pr. 19 50ng	27,11	76,20	1887,71	5453,07
63	F04	SYBR	HPRT1 RT	Unkn	Pr. 19 50ng	30,01	77,80	1577,01	4501,92
64	F05	SYBR	TBP	Unkn	Pr. 19 50ng	30,49	78,40	1696,00	4623,13
65	F07	SYBR	LATS1	Unkn	Pr. 20 50ng	29,65	75,60	2365,43	5348,28
66	F08	SYBR	SAV1	Unkn	Pr. 20 50ng	27,31	76,00	1847,83	5496,15
67	F09	SYBR	HPRT1 RT	Unkn	Pr. 20 50ng	31,26	77,80	1602,36	4405,80
68	F10	SYBR	TBP	Unkn	Pr. 20 50ng	30,77	78,40	1571,42	4498,69
69	F11	SYBR	SAV1	NRT	Pr. 19 50ng		None	None	-0,65
70	G02	SYBR	LATS1	Unkn	Pr. 19 50ng	29,80	75,60	2289,43	5112,89
71	G03	SYBR	SAV1	Unkn	Pr. 19 50ng	27,24	76,20	1833,68	5355,61
72	G04	SYBR	HPRT1 RT	Unkn	Pr. 19 50ng	29,90	77,80	1565,68	4539,34
73	G05	SYBR	TBP	Unkn	Pr. 19 50ng	30,73	78,40	1699,28	4718,94
74	G07	SYBR	LATS1	Unkn	Pr. 20 50ng	31,11	75,00	1703,51	3346,98
75	G08	SYBR	SAV1	Unkn	Pr. 20 50ng	27,42	76,00	1843,73	5495,54
76	G09	SYBR	HPRT1 RT	Unkn	Pr. 20 50ng	31,52	77,80	1581,59	4349,07
77	G10	SYBR	TBP	Unkn	Pr. 20 50ng	32,22	78,00	1071,49	3079,46
78	G11	SYBR	HPRT1 RT	NRT	Probe 19 50ng		None	None	0,28
79	G12	SYBR	LATS1	NRT	Pr. 17 50ng		None	None	0,15
80	H02	SYBR	LATS1	Unkn	Pr. 16 50ng	31,05	75,20	1573,63	3038,45
81	H03	SYBR	SAV1	Unkn	Pr. 16 50ng	30,21	75,60	1197,14	3198,15
82	H04	SYBR	HPRT1 RT	Unkn	Pr. 16 50ng	32,21	77,40	826,98	2276,30
83	H05	SYBR	TBP	Unkn	Pr. 16 50ng	32,28	78,00	884,61	2568,18
84	H07	SYBR	LATS1	Unkn	Pr. 16 50ng	30,95	75,20	1719,65	3376,80
85	H08	SYBR	SAV1	Unkn	Pr. 16 50ng	30,36	75,60	1272,87	3212,86
86	H09	SYBR	HPRT1 RT	Unkn	Pr. 16 50ng	32,15	77,20	903,96	2463,77
87	H10	SYBR	TBP	Unkn	Pr. 16 50ng	32,09	78,00	851,47	2636,59
88	H11	SYBR	TBP	NRT	Probe 20 50ng		None	None	-0,54
89	H12	SYBR	SAV1	NRT	Pr. 18 50ng		None	None	-0,89

	A	B	C	D	E	F	G	H	I
1	File Name	LATS1, SAV1 Sample 21 to 25.pcrd							
2	Created By	admin							
3	Notes								
4	ID								
5	Run Started	10/07/2025 13:17:07 UTC							
6	Run Ended	10/07/2025 15:13:15 UTC							
7	Sample Vol	20							
8	Lid Temp	105							
9	Protocol Fil	Originalprotokoll mit Schmelzkurve ab 60 Grad.prcl							
10	Plate Setup	Plattenvorlage Wiederholungsplatte IDT Primer neu.pltd							
11	Base Serial	BR203528							
12	Optical Hea	787BR12390							
13	CFX Manag	3.1.3086.0516.							
14									
15	Well group	All Wells							
16	Amplificatio	4							
17	Melt step	6							
18									
19									
20	Well	Fluor	Target	Content	Sample	Cq	Melt Temperature	Peak Height	End RFU
21	A02	SYBR	LATS1	Unkn	Pr. 25 50ng	32,29	75,60	2223,56	3960,84
22	A03	SYBR	SAV1	Unkn	Pr. 25 50ng	30,72	76,00	1702,12	4279,75
23	A04	SYBR	HPRT1 RT	Unkn	Pr. 25 50ng	32,03	77,80	1428,89	3575,60
24	A05	SYBR	TBP	Unkn	Pr. 25 50ng	32,26	78,40	1532,79	3770,66
25	B02	SYBR	LATS1	Unkn	Pr. 21 50ng	30,97	75,60	2272,26	4554,56
26	B03	SYBR	SAV1	Unkn	Pr. 21 50ng	28,13	76,20	1828,49	5087,90
27	B04	SYBR	HPRT1 RT	Unkn	Pr. 21 50ng	31,83	77,80	1501,09	3855,39
28	B05	SYBR	TBP	Unkn	Pr. 21 50ng	32,02	78,40	1572,56	4085,78
29	B07	SYBR	LATS1	Unkn	Pr. 22 50ng	30,34	75,60	2540,60	5205,21
30	B08	SYBR	SAV1	Unkn	Pr. 22 50ng	27,79	76,20	1983,73	5412,43
31	B09	SYBR	HPRT1 RT	Unkn	Pr. 22 50ng	30,72	77,80	1550,92	4157,77
32	B10	SYBR	TBP	Unkn	Pr. 22 50ng	30,97	78,40	1651,22	4442,98
33	B12	SYBR	LATS1	NTC	H20		None	None	1,24
34	C02	SYBR	LATS1	Unkn	Pr. 21 50ng	31,30	75,60	2187,92	4456,00
35	C03	SYBR	SAV1	Unkn	Pr. 21 50ng	28,03	76,20	1791,61	5068,45
36	C04	SYBR	HPRT1 RT	Unkn	Pr. 21 50ng	32,64	77,80	1551,60	3776,08
37	C05	SYBR	TBP	Unkn	Pr. 21 50ng	32,15	78,40	1590,81	4017,21
38	C07	SYBR	LATS1	Unkn	Pr. 22 50ng	30,20	75,60	2459,38	5191,96
39	C08	SYBR	SAV1	Unkn	Pr. 22 50ng	27,93	76,20	1975,15	5482,56
40	C09	SYBR	HPRT1 RT	Unkn	Pr. 22 50ng	30,61	77,80	1597,50	4448,91
41	C10	SYBR	TBP	Unkn	Pr. 22 50ng	31,03	78,40	1728,92	4628,61
42	C12	SYBR	SAV1	NTC	H20		None	None	-0,39
43	D02	SYBR	LATS1	Unkn	Pr. 21 50ng	31,05	75,60	2174,89	4666,65
44	D03	SYBR	SAV1	Unkn	Pr. 21 50ng	27,88	76,20	1877,85	5407,48
45	D04	SYBR	HPRT1 RT	Unkn	Pr. 21 50ng	31,59	77,80	1521,06	4061,88
46	D05	SYBR	TBP	Unkn	Pr. 21 50ng	32,04	78,40	1563,59	4112,04
47	D07	SYBR	LATS1	Unkn	Pr. 22 50ng	30,02	75,60	2397,49	5202,64
48	D08	SYBR	SAV1	Unkn	Pr. 22 50ng	27,77	76,20	1884,42	5386,59
49	D09	SYBR	HPRT1 RT	Unkn	Pr. 22 50ng	30,98	77,80	1620,10	4365,90
50	D10	SYBR	TBP	Unkn	Pr. 22 50ng	30,91	78,40	1722,26	4704,06
51	D12	SYBR	HPRT1 RT	NTC	H20		None	None	1,61
52	E02	SYBR	LATS1	Unkn	Pr. 23 50ng		None	None	-0,24
53	E03	SYBR	SAV1	Unkn	Pr. 23 50ng	32,60	76,00	1748,01	4117,30
54	E04	SYBR	HPRT1 RT	Unkn	Pr. 23 50ng	32,62	77,80	1560,37	3821,43
55	E05	SYBR	TBP	Unkn	Pr. 23 50ng	33,40	78,40	1495,57	3585,50

	A	B	C	D	E	F	G	H	I
56	E07	SYBR	LATS1	Unkn	Pr. 24 50ng	29,64	75,60	2507,22	5345,52
57	E08	SYBR	SAV1	Unkn	Pr. 24 50ng	29,16	76,00	1786,13	5013,81
58	E09	SYBR	HPRT1 RT	Unkn	Pr. 24 50ng	29,90	77,80	1640,89	4853,49
59	E10	SYBR	TBP	Unkn	Pr. 24 50ng	30,16	78,40	1611,32	4856,80
60	E12	SYBR	TBP	NTC	H20		None	None	-1,92
61	F02	SYBR	LATS1	Unkn	Pr. 23 50ng	34,57	75,60	2004,66	3035,12
62	F03	SYBR	SAV1	Unkn	Pr. 23 50ng	32,76	76,00	1595,80	3840,06
63	F04	SYBR	HPRT1 RT	Unkn	Pr. 23 50ng	32,79	77,80	1472,00	3695,69
64	F05	SYBR	TBP	Unkn	Pr. 23 50ng	33,29	78,40	1575,64	3833,95
65	F07	SYBR	LATS1	Unkn	Pr. 24 50ng	29,41	75,60	2333,00	5408,42
66	F08	SYBR	SAV1	Unkn	Pr. 24 50ng	29,18	76,00	1697,55	4848,19
67	F09	SYBR	HPRT1 RT	Unkn	Pr. 24 50ng	29,53	77,80	1583,34	4764,79
68	F10	SYBR	TBP	Unkn	Pr. 24 50ng	30,41	78,40	1505,03	4619,31
69	F11	SYBR	SAV1	NRT	Pr. 23 50ng		None	None	-1,75
70	G02	SYBR	LATS1	Unkn	Pr. 23 50ng	34,96	75,60	2056,70	3025,54
71	G03	SYBR	SAV1	Unkn	Pr. 23 50ng	32,56	76,00	1713,72	4129,85
72	G04	SYBR	HPRT1 RT	Unkn	Pr. 23 50ng	32,80	77,80	1481,93	3747,97
73	G05	SYBR	TBP	Unkn	Pr. 23 50ng	33,07	78,40	1567,77	3961,88
74	G07	SYBR	LATS1	Unkn	Pr. 24 50ng	29,46	75,60	2368,48	5498,84
75	G08	SYBR	SAV1	Unkn	Pr. 24 50ng	28,82	76,00	1845,71	5326,43
76	G09	SYBR	HPRT1 RT	Unkn	Pr. 24 50ng	29,47	77,80	1507,71	4643,36
77	G10	SYBR	TBP	Unkn	Pr. 24 50ng	30,15	78,40	1502,54	4875,13
78	G11	SYBR	HPRT1 RT	NRT	Probe 24 50ng		None	None	-0,44
79	G12	SYBR	LATS1	NRT	Pr. 22 50ng		None	None	1,06
80	H02	SYBR	LATS1	Unkn	Pr. 25 50ng	32,56	75,60	2093,76	4159,81
81	H03	SYBR	SAV1	Unkn	Pr. 25 50ng	30,09	76,20	1821,90	5075,94
82	H04	SYBR	HPRT1 RT	Unkn	Pr. 25 50ng	31,45	77,80	1436,62	4055,95
83	H05	SYBR	TBP	Unkn	Pr. 25 50ng	32,31	78,40	1448,25	4048,85
84	H07	SYBR	LATS1	Unkn	Pr. 25 50ng	31,71	75,60	2239,17	4758,04
85	H08	SYBR	SAV1	Unkn	Pr. 25 50ng	30,06	76,00	1891,69	5258,41
86	H09	SYBR	HPRT1 RT	Unkn	Pr. 25 50ng	31,47	77,80	1522,22	4272,34
87	H10	SYBR	TBP	Unkn	Pr. 25 50ng	32,55	78,40	1470,10	4138,77
88	H11	SYBR	TBP	NRT	Probe 25 50ng		None	None	0,63
89	H12	SYBR	SAV1	NRT	Pr. 21 50ng		None	None	1,58

	A	B	C	D	E	F	G	H	I
1	File Name	LATS1, SAV1 Sample 10, 26, 20.pcrd							
2	Created By	admin							
3	Notes								
4	ID								
5	Run Started	10/08/2025 15:36:47 UTC							
6	Run Ended	10/08/2025 17:33:12 UTC							
7	Sample Vol	20							
8	Lid Temp	105							
9	Protocol Fil	Originalprotokoll mit Schmelzkurve ab 60 Grad.prcl							
10	Plate Setup	Plattenvorlage Wiederholungsplatte IDT Primer.pltd							
11	Base Serial	BR203528							
12	Optical Hea	787BR12390							
13	CFX Manag	3.1.3086.0516.							
14									
15	Well group	All Wells							
16	Amplificatio	4							
17	Melt step	6							
18									
19									
20	Well	Fluor	Target	Content	Sample	Cq	Melt Temperature	Peak Height	End RFU
21	A10	SYBR	HPRT1 RT	NTC	H20		None	None	-0,44
22	A11	SYBR	TBP	NTC	H20		None	None	0,64
23	B02	SYBR	LATS1	Unkn	Pr. 20 50ng	30,18	75,00	1384,24	2471,63
24	B03	SYBR	SAV1	Unkn	Pr. 20 50ng	27,20	75,60	1161,77	3109,71
25	B08	SYBR	HPRT1 RT	Unkn	Pr. 20 50ng	31,65	77,20	825,50	1941,75
26	B09	SYBR	TBP	Unkn	Pr. 20 50ng	31,02	78,00	831,44	2295,21
27	B12	SYBR	LATS1	NTC	H20		None	None	-1,32
28	C02	SYBR	LATS1	Unkn	Pr. 20 50ng	29,77	75,20	1390,54	2606,04
29	C03	SYBR	SAV1	Unkn	Pr. 20 50ng	27,19	75,60	1143,09	3132,54
30	C08	SYBR	HPRT1 RT	Unkn	Pr. 20 50ng	31,47	77,20	800,77	1993,51
31	C09	SYBR	TBP	Unkn	Pr. 20 50ng	31,20	78,00	847,59	2345,79
32	C12	SYBR	SAV1	NTC	H20		None	None	-0,45
33	D02	SYBR	LATS1	Unkn	Pr. 20 50ng	30,31	75,20	1261,93	2452,93
34	D03	SYBR	SAV1	Unkn	Pr. 20 50ng	27,36	75,60	1139,44	3166,56
35	D08	SYBR	HPRT1 RT	Unkn	Pr. 20 50ng	31,49	77,20	802,74	1991,02
36	D09	SYBR	TBP	Unkn	Pr. 20 50ng	31,15	77,80	771,32	2251,63
37	E02	SYBR	LATS1	Unkn	Pr. 10 50ng	32,79	75,00	1131,79	1832,61
38	E03	SYBR	SAV1	Unkn	Pr. 10 50ng	31,29	75,60	975,68	2285,09
39	E04	SYBR	HPRT1 RT	Unkn	Pr. 10 50ng	32,13	77,20	761,20	1777,12
40	E05	SYBR	TBP	Unkn	Pr. 10 50ng	32,49	78,00	757,64	1846,65
41	E07	SYBR	LATS1	Unkn	Pr. 26 50ng	35,74	75,40	1319,28	999,48
42	E08	SYBR	SAV1	Unkn	Pr. 26 50ng	32,76	75,80	1279,94	1795,02
43	E09	SYBR	HPRT1 RT	Unkn	Pr. 26 50ng	35,76	77,40	966,98	929,12
44	E10	SYBR	TBP	Unkn	Pr. 26 50ng	35,27	78,20	1088,47	1101,83
45	F02	SYBR	LATS1	Unkn	Pr. 10 50ng	31,72	75,20	1255,97	2181,88
46	F03	SYBR	SAV1	Unkn	Pr. 10 50ng	31,30	75,60	961,82	2272,01
47	F04	SYBR	HPRT1 RT	Unkn	Pr. 10 50ng	31,79	77,20	745,38	1879,51
48	F05	SYBR	TBP	Unkn	Pr. 10 50ng	32,39	77,80	719,68	1966,56
49	F07	SYBR	LATS1	Unkn	Pr. 26 50ng	35,27	75,40	1411,25	1163,35
50	F08	SYBR	SAV1	Unkn	Pr. 26 50ng	33,96	75,80	1209,14	1470,13
51	F09	SYBR	HPRT1 RT	Unkn	Pr. 26 50ng	35,25	77,60	979,62	1023,92
52	F10	SYBR	TBP	Unkn	Pr. 26 50ng	35,38	78,00	874,23	1039,60
53	G02	SYBR	LATS1	Unkn	Pr. 10 50ng	32,18	75,20	1342,21	2177,38
54	G03	SYBR	SAV1	Unkn	Pr. 10 50ng	31,26	75,60	987,71	2339,32
55	G04	SYBR	HPRT1 RT	Unkn	Pr. 10 50ng	31,86	77,20	757,76	1866,94

	A	B	C	D	E	F	G	H	I
56	G05	SYBR	TBP	Unkn	Pr. 10 50ng	31,95	78,00	739,24	2133,35
57	G07	SYBR	LATS1	Unkn	Pr. 26 50ng		None	None	10,06
58	G08	SYBR	SAV1	Unkn	Pr. 26 50ng	33,21	75,80	1200,86	1774,92
59	G09	SYBR	HPRT1 RT	Unkn	Pr. 26 50ng	35,16	77,40	994,07	1097,07
60	G10	SYBR	TBP	Unkn	Pr. 26 50ng	36,01	78,20	854,79	892,87
61	H12	SYBR	SAV1	NRT	Pr. 26 50ng		None	None	4,98

	A	B	C	D	E	F	G	H	I
1	File Name	Validation Cycler 2, reagent lot 2.pcrd							
2	Created By	admin							
3	Notes								
4	ID								
5	Run Started	07/24/2025 13:48:04 UTC							
6	Run Ended	07/24/2025 15:43:51 UTC							
7	Sample Vol	20							
8	Lid Temp	105							
9	Protocol Fil	Originalprotokoll mit Schmelzkurve ab 60 Grad.prcl							
10	Plate Setup	Plattenvorlage Vergleichstestung.pltd							
11	Base Serial	BR203528							
12	Optical Hea	787BR12390							
13	CFX Manag	3.1.3086.0516.							
14									
15	Well group	All Wells							
16	Amplificatio	4							
17	Melt step	6							
18									
19									
20	Well	Fluor	Target	Content	Sample	Cq	Melt Temperature	Peak Height	End RFU
21	A12	SYBR	YAP1	NTC	H20		None	None	0,19
22	B01	SYBR	YAP1	Unkn	Probe 3 50ng Cy	35,36	None	None	1686,46
23	B02	SYBR	MOB1A	Unkn	Probe 3 50ng Cy	28,74	76,40	2608,23	6023,85
24	B03	SYBR	TBP	Unkn	Probe 3 50ng Cy	32,46	78,20	1801,55	4218,18
25	B04	SYBR	HPRT1 RTP	Unkn	Probe 3 50ng Cy	29,60	77,40	2191,69	6061,72
26	B07	SYBR	YAP1	Unkn	Probe 3 50ng RL 2	34,32	None	None	1504,96
27	B08	SYBR	MOB1A	Unkn	Probe 3 50ng RL 2	28,99	76,20	1452,62	3285,29
28	B09	SYBR	TBP	Unkn	Probe 3 50ng RL 2	32,24	78,00	982,10	2616,05
29	B10	SYBR	HPRT1 RTP	Unkn	Probe 3 50ng RL 2	29,39	77,40	1261,24	3481,28
30	B12	SYBR	MOB1A	NTC	H20		None	None	-1,50
31	C01	SYBR	YAP1	Unkn	Probe 3 50ng Cy	33,53	80,00	1156,16	2746,71
32	C02	SYBR	MOB1A	Unkn	Probe 3 50ng Cy	28,42	76,40	2740,89	6497,08
33	C03	SYBR	TBP	Unkn	Probe 3 50ng Cy	32,12	78,20	1907,20	4581,34
34	C04	SYBR	HPRT1 RTP	Unkn	Probe 3 50ng Cy	29,42	77,40	2376,12	6634,91
35	C07	SYBR	YAP1	Unkn	Probe 3 50ng RL 2	33,50	None	None	1560,38
36	C08	SYBR	MOB1A	Unkn	Probe 3 50ng RL 2	28,68	76,20	1503,13	3494,00
37	C09	SYBR	TBP	Unkn	Probe 3 50ng RL 2	32,58	None	None	2462,25
38	C10	SYBR	HPRT1 RTP	Unkn	Probe 3 50ng RL 2	29,26	77,20	1273,29	3686,86
39	C12	SYBR	HPRT1 RTP	NTC	H20		None	None	-1,48
40	D01	SYBR	YAP1	Unkn	Probe 3 50ng Cy	34,65	79,80	1118,47	2333,24
41	D02	SYBR	MOB1A	Unkn	Probe 3 50ng Cy	28,24	76,40	2598,02	6350,57
42	D03	SYBR	TBP	Unkn	Probe 3 50ng Cy	31,97	78,20	1825,55	4848,54
43	D04	SYBR	HPRT1 RTP	Unkn	Probe 3 50ng Cy	29,47	77,40	2282,27	6576,31
44	D07	SYBR	YAP1	Unkn	Probe 3 50ng RL 2	33,67	None	None	1690,66
45	D08	SYBR	MOB1A	Unkn	Probe 3 50ng RL 2	29,03	76,20	1439,52	3284,79
46	D09	SYBR	TBP	Unkn	Probe 3 50ng RL 2	32,30	None	None	2587,59
47	D10	SYBR	HPRT1 RTP	Unkn	Probe 3 50ng RL 2	29,31	77,40	1190,43	3386,50
48	D12	SYBR	TBP	NTC	H20		None	None	0,60
49	E01	SYBR	YAP1	Unkn	Probe 9 50ng Cy	31,26	80,20	1415,75	3900,54
50	E02	SYBR	MOB1A	Unkn	Probe 9 50ng Cy	26,75	76,60	2917,25	7191,00
51	E03	SYBR	TBP	Unkn	Probe 9 50ng Cy	29,61	78,20	2332,67	6432,66
52	E04	SYBR	HPRT1 RTP	Unkn	Probe 9 50ng Cy	27,28	77,60	2521,86	7351,01
53	E07	SYBR	YAP1	Unkn	Probe 9 50ng RL2	31,12	None	None	2285,97
54	E08	SYBR	MOB1A	Unkn	Probe 9 50ng RL2	26,53	76,40	1677,08	4001,02
55	E09	SYBR	TBP	Unkn	Probe 9 50ng RL2	29,96	78,00	1182,77	3269,31

	A	B	C	D	E	F	G	H	I
56	E10	SYBR	HPRT1 RTP	Unkn	Probe 9 50ng RL2	27,17	77,40	1366,88	3885,32
57	F01	SYBR	YAP1	Unkn	Probe 9 50ng Cy	31,37	80,20	1465,63	4029,66
58	F02	SYBR	MOB1A	Unkn	Probe 9 50ng Cy	26,62	76,60	2878,45	7250,67
59	F03	SYBR	TBP	Unkn	Probe 9 50ng Cy	29,75	78,20	2219,88	6229,40
60	F04	SYBR	HPRT1 RTP	Unkn	Probe 9 50ng Cy	27,23	77,60	2572,71	7742,73
61	F07	SYBR	YAP1	Unkn	Probe 9 50ng RL2	30,88	None	None	2257,86
62	F08	SYBR	MOB1A	Unkn	Probe 9 50ng RL2	26,45	76,20	1672,29	4060,86
63	F09	SYBR	TBP	Unkn	Probe 9 50ng RL2	29,60	78,00	1253,49	3532,24
64	F10	SYBR	HPRT1 RTP	Unkn	Probe 9 50ng RL2	27,04	77,40	1429,99	4106,81
65	G01	SYBR	YAP1	Unkn	Probe 9 50ng Cy	31,33	80,20	1460,49	4007,78
66	G02	SYBR	MOB1A	Unkn	Probe 9 50ng Cy	26,65	76,60	2828,07	7162,54
67	G03	SYBR	TBP	Unkn	Probe 9 50ng Cy	29,43	78,20	2217,01	6279,22
68	G04	SYBR	HPRT1 RTP	Unkn	Probe 9 50ng Cy	26,84	77,60	2482,23	7549,01
69	G07	SYBR	YAP1	Unkn	Probe 9 50ng RL2	30,80	None	None	2396,96
70	G08	SYBR	MOB1A	Unkn	Probe 9 50ng RL2	26,68	76,20	1521,74	3697,76
71	G09	SYBR	TBP	Unkn	Probe 9 50ng RL2	29,68	78,00	1210,76	3452,59
72	G10	SYBR	HPRT1 RTP	Unkn	Probe 9 50ng RL2	27,02	77,40	1363,67	3987,85

	A	B	C	D	E	F	G	H	I
1	File Name	Validation reagent lot 3.pcrd							
2	Created By	admin							
3	Notes								
4	ID								
5	Run Started	08/04/2025 12:31:56 UTC							
6	Run Ended	08/04/2025 14:27:41 UTC							
7	Sample Vol	20							
8	Lid Temp	105							
9	Protocol Fil	Originalprotokoll mit Schmelzkurve ab 60 Grad.prcl							
10	Plate Setup	Plattenvorlage Vergleichstestung.pltd							
11	Base Serial	BR203528							
12	Optical Hea	787BR12390							
13	CFX Manag	3.1.3086.0516.							
14									
15	Well group	All Wells							
16	Amplificatio	4							
17	Melt step	6							
18									
19									
20	Well	Fluor	Target	Content	Sample	Cq	Melt Temperature	Peak Height	End RFU
21	A12	SYBR	YAP1	NTC	H20		None	None	-1,84
22	B01	SYBR	YAP1	Unkn	Probe 3 50ng	33,78	79,60	428,56	1181,81
23	B02	SYBR	MOB1A	Unkn	Probe 3 50ng	28,82	76,20	1133,40	2538,18
24	B03	SYBR	TBP	Unkn	Probe 3 50ng	32,05	77,80	699,23	1965,64
25	B04	SYBR	HPRT1 RTP	Unkn	Probe 3 50ng	29,44	77,20	888,40	2459,54
26	B05	SYBR	YAP1	Unkn	Probe 3 50ng	31,54	80,20	1071,86	2871,42
27	B12	SYBR	MOB1A	NTC	H20		None	None	-0,94
28	C01	SYBR	YAP1	Unkn	Probe 3 50ng	33,96	79,80	425,97	1182,58
29	C02	SYBR	MOB1A	Unkn	Probe 3 50ng	29,15	76,20	970,85	2265,31
30	C03	SYBR	TBP	Unkn	Probe 3 50ng	32,33	78,00	654,63	1748,34
31	C04	SYBR	HPRT1 RTP	Unkn	Probe 3 50ng	29,42	77,20	858,27	2419,99
32	C05	SYBR	YAP1	Unkn	Probe 3 50ng	31,19	80,20	1011,97	2873,76
33	C12	SYBR	TBP	NTC	H20		None	None	-2,03
34	D01	SYBR	YAP1	Unkn	Probe 3 50ng	33,62	79,60	393,51	1252,29
35	D02	SYBR	MOB1A	Unkn	Probe 3 50ng	28,85	76,40	1053,56	2546,72
36	D03	SYBR	TBP	Unkn	Probe 3 50ng	32,22	78,00	692,16	1915,18
37	D04	SYBR	HPRT1 RTP	Unkn	Probe 3 50ng	29,44	77,20	848,46	2433,89
38	D05	SYBR	YAP1	Unkn	Probe 3 50ng	31,65	80,20	872,27	2498,99
39	D12	SYBR	HPRT1 RTP	NTC	H20		None	None	-0,24
40	E01	SYBR	YAP1	Unkn	Probe 9 50ng	31,10	80,00	560,67	1698,65
41	E02	SYBR	MOB1A	Unkn	Probe 9 50ng	26,61	76,40	1206,76	2817,33
42	E03	SYBR	TBP	Unkn	Probe 9 50ng	29,75	78,00	803,80	2248,50
43	E04	SYBR	HPRT1 RTP	Unkn	Probe 9 50ng	27,13	77,40	1016,97	2851,21
44	E05	SYBR	MOB1A	Unkn	Probe 9 50ng	24,70	76,80	1536,96	4178,35
45	F01	SYBR	YAP1	Unkn	Probe 9 50ng	31,03	80,00	632,99	1810,88
46	F02	SYBR	MOB1A	Unkn	Probe 9 50ng	26,58	76,40	1199,96	2840,73
47	F03	SYBR	TBP	Unkn	Probe 9 50ng	29,69	78,00	820,17	2325,61
48	F04	SYBR	HPRT1 RTP	Unkn	Probe 9 50ng	27,21	77,40	973,30	2788,72
49	F05	SYBR	MOB1A	Unkn	Probe 9 50ng	24,65	76,80	1393,68	3930,42
50	G01	SYBR	YAP1	Unkn	Probe 9 50ng	30,86	80,00	546,52	1673,03
51	G02	SYBR	MOB1A	Unkn	Probe 9 50ng	26,35	76,40	1190,97	2855,10
52	G03	SYBR	TBP	Unkn	Probe 9 50ng	29,59	78,00	779,03	2252,27
53	G04	SYBR	HPRT1 RTP	Unkn	Probe 9 50ng	27,19	77,40	911,60	2681,56
54	G05	SYBR	MOB1A	Unkn	Probe 9 50ng	24,56	76,80	1545,55	4261,87

	A	B	C	D	E	F	G	H	I
1	File Name	Validation reagent lot 4.pcrd							
2	Created By	admin							
3	Notes								
4	ID								
5	Run Started	07/28/2025 08:02:02 UTC							
6	Run Ended	07/28/2025 09:57:46 UTC							
7	Sample Vol	20							
8	Lid Temp	105							
9	Protocol Fil	Originalprotokoll mit Schmelzkurve ab 60 Grad.prcl							
10	Plate Setup	Plattenvorlage Vergleichstestung.pltd							
11	Base Serial	BR203528							
12	Optical Hea	787BR12390							
13	CFX Manag	3.1.3086.0516.							
14									
15	Well group	All Wells							
16	Amplificatio	4							
17	Melt step	6							
18									
19									
20	Well	Fluor	Target	Content	Sample	Cq	Melt Temperature	Peak Height	End RFU
21	A12	SYBR	YAP1	NTC	H20		None	None	42,48
22	B01	SYBR	YAP1	Unkn	Probe 3 50ng	34,26	79,60	448,39	948,92
23	B02	SYBR	MOB1A	Unkn	Probe 3 50ng	28,96	76,20	1130,80	2339,31
24	B03	SYBR	TBP	Unkn	Probe 3 50ng	32,33	77,80	704,43	1686,09
25	B04	SYBR	HPRT1 RTP	Unkn	Probe 3 50ng	29,10	77,20	976,50	2557,05
26	B12	SYBR	MOB1A	NTC	H20		None	None	2,74
27	C01	SYBR	YAP1	Unkn	Probe 3 50ng	33,78	79,60	449,17	1067,16
28	C02	SYBR	MOB1A	Unkn	Probe 3 50ng	29,01	76,20	1114,55	2395,56
29	C03	SYBR	TBP	Unkn	Probe 3 50ng	32,12	77,80	746,06	1772,28
30	C04	SYBR	HPRT1 RTP	Unkn	Probe 3 50ng	28,94	77,20	986,33	2663,68
31	C12	SYBR	TBP	NTC	H20		None	None	2,17
32	D01	SYBR	YAP1	Unkn	Probe 3 50ng	34,26	79,80	458,95	1036,32
33	D02	SYBR	MOB1A	Unkn	Probe 3 50ng	28,81	76,20	1151,19	2567,44
34	D03	SYBR	TBP	Unkn	Probe 3 50ng	32,30	77,80	668,64	1687,84
35	D04	SYBR	HPRT1 RTP	Unkn	Probe 3 50ng	28,99	77,20	968,47	2645,06
36	D12	SYBR	HPRT1 RTP	NTC	H20	38,98	None	None	213,22
37	E01	SYBR	YAP1	Unkn	Probe 9 50ng	30,65	80,00	640,34	1675,51
38	E02	SYBR	MOB1A	Unkn	Probe 9 50ng	26,30	76,40	1336,80	3063,12
39	E03	SYBR	TBP	Unkn	Probe 9 50ng	29,41	78,00	985,55	2535,99
40	E04	SYBR	HPRT1 RTP	Unkn	Probe 9 50ng	26,39	77,40	1136,45	3178,05
41	F01	SYBR	YAP1	Unkn	Probe 9 50ng	30,73	80,00	641,18	1732,25
42	F02	SYBR	MOB1A	Unkn	Probe 9 50ng	26,21	76,40	1269,99	2985,49
43	F03	SYBR	TBP	Unkn	Probe 9 50ng	29,23	78,00	924,92	2547,80
44	F04	SYBR	HPRT1 RTP	Unkn	Probe 9 50ng	26,22	77,40	1109,69	3193,71
45	G01	SYBR	YAP1	Unkn	Probe 9 50ng	30,62	80,00	608,78	1722,29
46	G02	SYBR	MOB1A	Unkn	Probe 9 50ng	26,29	76,40	1274,64	3011,78
47	G03	SYBR	TBP	Unkn	Probe 9 50ng	29,17	78,00	936,52	2528,41
48	G04	SYBR	HPRT1 RTP	Unkn	Probe 9 50ng	26,29	77,20	1122,31	3151,34
