# Supplementary material for: Subtype-Dependent Expression Patterns of Core Hippo Pathway Components in Thymic Epithelial Tumors (TETs): An RT-qPCR Study
Source: Biomedicines. 2026 Jan 29;14(2):305. doi: 10.3390/biomedicines14020305 (PMC12937678; doi:10.3390/biomedicines14020305)
Supplement: Supplementary file 1 [file biomedicines-14-00305-s001.zip › Table S14 Replicate-level QC and exclusion criteria of samples included in analysis.pdf]

Table S14 Replicate-level QC and exclusion criteria of samples included in analysis. Triplicates were assessed based on intra-assay variability. All three Cq values within ≤ 0.5 Cq were considered technically consistent; Any replicate deviating by > 0.5 Cq from the mean of the two most consistent replicates was defined as a technical outlier and excluded ("gold standard“). For FFPE-derived RNA, a total spread of up to 0.8 Cq was accepted ("FFPE accepted“). Reactions without two consistent replicates (ΔCq > 0.8) were classified as invalid and repeated.

Target	Cq values	Total-ΔCq	Δ best 2	Used Replicates	Mean (Cq)	SD	Evaluation	Comment
**YAP1**	34.80 / 34.03 / 33.73	1.07	0.30	34.03 / 33.73	**33.88**	**0.21**	gold standard	Two consistent replicates (ΔCq = 0.30); third replicate excluded as technical outlier (> 1 Cq)
**MST1**	29.26 / 29.14 / 28.95	0.31	0.19	29.26 / 29.14 / 28.95	**29.12**	**0.16**	gold standard	
**TEAD4**	36.49 / 34.62 / 34.52	1.97	0.10	34.62 / 34.52	**34.57**	**0.07**	gold standard	Two consistent replicates (ΔCq = 0.10); third replicate excluded as technical outlier (> 1 Cq).
**TBP**	31.83 / 31.60 / 31.99	0.39	0.23	31.83 / 31.60 / 31.99	**31.81**	**0.20**	gold standard	
**HPRT1-RTP**	29.46 / 29.67 / 29.03	0.64	0.21	29.46 / 29.67	**29.57**	**0.15**	gold standard	Δ_best2 < 0.5, outlier >0,5 away from the mean of the Δ_best2 excluded.

Sample 1

Repetition

Target	Cq values	Total-ΔCq	Δ best 2	Used Replicates	Mean (Cq)	SD	Evaluation
**MOB1A**	25.96 / 26.09 / 25.93	0.16	0.13	25.96 / 26.09 / 25.93	**25.99**	**0.08**	gold standard
**TBP**	29.15 / 29.34 / 29.30	0.19	0.15	29.15 / 29.34 / 29.30	**29.26**	**0.10**	gold standard
**HPRT1-RTP**	27.09 / 27.17 / 27.22	0.13	0.08	27.09 / 27.17 / 27.22	**27.16**	**0.07**	gold standard

Block 2

Target	Cq values	Total-ΔCq	Δ best 2	Used Replicates	Mean (Cq)	SD	Evaluation	Comment
**LATS1**	29.11 / 37.47 / 28.80	8.67	0.31	29.11 / 28.80	**28.96**	**0.22**	gold standard	Two consistent replicates (ΔCq = 0.31); third replicate excluded as technical outlier (> 1 Cq).
**SAV1**	29.77 / 29.14 / 29.23	0.63	0.09	29.14 / 29.23	**29.19**	**0.06**	gold standard	Δ_best2 < 0.5, outlier >0,5 away from the mean of the Δ_best2 excluded.
**HPRT1-RTP**	30.50 / 30.33 / 30.24	0.26	0.09	30.50 / 30.33 / 30.24	**30.36**	**0.13**	gold standard	
**TBP-IDT**	29.92 / 30.39 / 30.00	0.47	0.08	29.92 / 30.39 / 30.00	**30.10**	**0.25**	gold standard	

Sample 2

Target	Cq values	Total-ΔCq	Δ best 2	Used Replicates	Mean (Cq)	SD	Evaluation	Comment
**YAP1**	33.65 / 33.60 / 33.75	0.15	0.05	all 3	**33.67**	**0.08**	gold standard	
**MST1**	30.23 / 30.46 / 30.21	0.25	0.02	all 3	**30.30**	**0.14**	gold standard	
**MOB1A**	29.41 / 29.35 / 29.16	0.25	0.19	all 3	**29.31**	**0.13**	gold standard	
**TEAD4**	35.39 / 35.58 / 36.11	0.72	0.19	35.39 / 35.58	**35.49**	**0.13**	gold standard	Two consistent replicates (ΔCq = 0.19); third replicate excluded as technical outlier (> 1 Cq).
**TBP**	32.62 / 32.65 / 33.07	0.45	0.03	all 3	**32.78**	**0.25**	gold standard	
**HPRT1-RTP**	30.72 / 30.85 / 31.69	0.97	0.13	30.72 / 30.85	**30.79**	**0.09**	gold standard	Two consistent replicates (ΔCq = 0.13); third replicate excluded as technical outlier (> 1 Cq).

Block 2

Target	Cq values	Total-ΔCq	Δ best 2	Used Replicates	Mean (Cq)	SD	Evaluation	Comment
**LATS1**	29.28 / 31.23 / 29.30	1.95	0.02	29.28 / 29.30	**29.29**	**0.01**	gold standard	Two consistent replicates (ΔCq = 0.02); third replicate excluded as technical outlier (> 1 Cq).
**SAV1**	29.44 / 29.40 / 29.44	0.04	0.04	all 3	**29.43**	**0.02**	gold standard	
**HPRT1-RTP**	31.95 / 31.64 / 31.76	0.31	0.12	all 3	**31.78**	**0.13**	gold standard	
**TBP-IDT**	30.67 / 30.61 / 35.39	4.78	0.06	30.67 / 30.61	**30.64**	**0.04**	gold standard	Two consistent replicates (ΔCq = 0.06); third replicate excluded as technical outlier (> 1 Cq).

Sample 3

Target	Cq values	Total-ΔCq	Δ best 2	Used Replicates	Mean (Cq)	SD	Evaluation	Comment
**YAP1**	33.91 / 33.93 / 34.54	0.63	0.02	33.91 / 33.93	**33.92**	**0.01**	gold standard	Two consistent replicates (ΔCq = 0.02); third replicate excluded as technical outlier (> 0,5 Cq).
**MST1**	30.43 / 36.20 / 30.45	5.77	0.02	30.43 / 30.45	**30.44**	**0.01**	gold standard	Two consistent replicates (ΔCq = 0.02); third replicate excluded as technical outlier (> 1 Cq).
**MOB1A**	28.63 / 28.66 / 28.60	0.06	0.03	alle 3	**28.63**	**0.03**	gold standard	
**TEAD4**	34.62 / 35.43 / 34.09	1.34	0.53	34.62 / 34.09	**34.36**	**0.38**	FFPE accepted	Two replicates within FFPE range (ΔCq = 0.53); third replicate excluded as technical outlier (> 1 Cq).
**TBP**	32.05 / 32.26 / 32.30	0.25	0.21	all 3	**32.20**	**0.13**	gold standard	
**HPRT1-RTP**	29.87 / 29.82 / 29.67	0.20	0.05	all 3	**29.79**	**0.10**	gold standard	

Block 2

Target	Cq values	Total-ΔCq	Δ best 2	Used Replicates	Mean (Cq)	SD	Evaluation	Comment
**LATS1**	30.61 / 30.52 / 30.54	0.09	0.09	all 3	**30.56**	**0.05**	gold standard	
**SAV1**	30.32 / 30.12 / 30.20	0.20	0.08	all 3	**30.21**	**0.10**	gold standard	
**HPRT1-RTP**	31.61 / 31.63 / 31.54	0.09	0.09	all 3	**31.59**	**0.05**	gold standard	
**TBP-IDT**	31.53 / 31.48 / 31.23	0.30	0.25	all 3	**31.41**	**0.16**	gold standard	

Sample 4

Target	Cq values	Total-ΔCq	Δ best 2	Used Replicates	Mean (Cq)	SD	Evaluation	Comment
**YAP1**	32.85 / 32.26 / 32.35	0.59	0.09	all 3	**32.49**	**0.32**	FFPE accepted	
**MST1**	28.81 / 29.32 / 28.73	0.59	0.08	all 3	**28.95**	**0.31**	FFPE accepted	
**MOB1A**	28.26 / 28.54 / 28.34	0.28	0.08	all 3	**28.38**	**0.14**	gold standard	
**TEAD4**	33.62 / 35.35 / 34.23	1.73	0.61	33.62 / 34.23	**33.93**	**0.43**	FFPE accepted	Two replicates within FFPE range (ΔCq = 0.61); third replicate excluded as technical outlier (> 1 Cq).
**TBP**	32.44 / 32.99 / 32.32	0.67	0.12	all 3	**32.58**	**0.36**	FFPE accepted	
**HPRT1-RTP**	30.29 / 30.22 / 30.30	0.08	0.07	all 3	**30.27**	**0.04**	gold standard	

Block 2

Target	Cq values	Total-ΔCq	Δ best 2	Used Replicates	Mean (Cq)	SD	Evaluation	Comment
**LATS1**	28.36 / 28.19 / 28.13	0.23	0.06	all 3	**28.23**	**0.12**	gold standard	
**SAV1**	27.18 / 32.48 / 26.73	5.75	0.45	27.18 / 26.73	**26.96**	**0.32**	gold standard	Two consistent replicates (ΔCq = 0.45); third replicate excluded as technical outlier (> 1 Cq).
**HPRT1-RTP**	29.77 / 29.66 / 30.01	0.35	0.11	all 3	**29.81**	**0.18**	gold standard	
**TBP-IDT**	29.16 / 29.05 / 29.12	0.11	0.11	all 3	**29.11**	**0.06**	gold standard	

Sample 5

Target	Cq values	Total-ΔCq	Δ best	Used 2 Replicates	Mean (Cq)	SD	Evaluation	Comment
**YAP1**	32.85 / 32.35 / 32.13	0.72	0.22	32.35 / 32.13	**32.24**	**0.16**	gold standard	Two consistent replicates (ΔCq = 0.22); third replicate excluded as technical outlier (> 1 Cq).
**MST1**	31.83 / 31.52 / 31.76	0.31	0.24	all 3	**31.70**	**0.16**	gold standard	
**MOB1A**	30.14 / 30.29 / 30.29	0.15	0.00	all 3	**30.24**	**0.09**	gold standard	
**TEAD4**	33.44 / 34.33 / 34.45	1.01	0.12	33.44 / 34.33	**33.89**	**0.64**	gold standard	Two consistent replicates (ΔCq = 0.11); third replicate excluded as technical outlier (> 1 Cq).
**TBP**	33.18 / 33.48 / 33.35	0.30	0.17	all 3	**33.34**	**0.15**	gold standard	
**HPRT1-RTP**	31.83 / 31.92 / 37.48	5.65	0.09	31.83 / 31.92	**31.88**	**0.06**	gold standard	Two consistent replicates (ΔCq = 0.09); third replicate excluded as technical outlier (> 1 Cq).

Block 2

Target	Cq values	Total-ΔCq	Δ best	Used 2 Replicates	Mean (Cq)	SD	Evaluation	Comment
**LATS1**	30.18 / 30.37 / 30.02	0.35	0.16	all 3	**30.19**	**0.18**	gold standard	
**SAV1**	27.59 / 27.11 / 29.65	2.54	0.48	27.59 / 27.11	**27.35**	**0.34**	gold standard	Two consistent replicates (ΔCq = 0.48); third replicate excluded as technical outlier (> 1 Cq).
**HPRT1-RTP**	33.52 / 32.88 / –	0.64	–	33.52 / 32.88	**33.20**	**0.45**	FFPE accepted	Pipetting error, only two replicates avaible.
**TBP-IDT**	31.83 / 31.31 / 31.23	0.60	0.08	all 3	**31.46**	**0.33**	FFPE accepted	

Sample 6

Target	Cq values	Total-ΔCq	Δ best	Used 2 Replicates	Mean (Cq)	SD	Evaluation	Comment
**YAP1**	30.56 / 37.90 / 30.05	7.85	0.51	30.56 / 30.05	**30.31**	**0.36**	FFPE accepted	Two replicates within FFPE range (ΔCq = 0.51); third replicate excluded as technical outlier (> 1 Cq).
**MST1**	30.61 / 31.23 / 30.89	0.62	0.28	all 3	**30.91**	**0.31**	FFPE accepted	
**MOB1A**	28.37 / 28.54 / 28.43	0.17	0.06	all 3	**28.45**	**0.09**	gold standard	
**TEAD4**	32.53 / 37.91 / 33.17	5.38	0.64	32.53 / 33.17	**32.85**	**0.45**	FFPE accepted	Two replicates within FFPE range (ΔCq = 0.64); third replicate excluded as technical outlier (> 1 Cq).
**TBP**	31.82 / 32.17 / 31.93	0.35	0.11	all 3	**31.97**	**0.18**	gold standard	
**HPRT1-RTP**	30.04 / — / 29.88	0.16	0.16	30.04 / 29.88	**29.96**	**0.11**	gold standard	Pipetting error, only two replicates avaible.

Block 2

Target	Cq values	Total-ΔCq	Δ best	Used 2 Replicates	Mean (Cq)	SD	Evaluation	Comment
**LATS1**	28.38 / 32.22 / 28.40	3.84	0.02	28.38 / 28.40	**28.39**	**0.01**	gold standard	Two consistent replicates (ΔCq = 0.02); third replicate excluded as technical outlier (> 1 Cq).
**SAV1**	26.36 / 26.05 / 26.22	0.31	0.17	all 3	**26.21**	**0.16**	gold standard	
**HPRT1-RTP**	30.09 / 30.23 / 29.83	0.40	0.26	all 3	**30.05**	**0.20**	gold standard	
**TBP-IDT**	29.37 / 29.20 / 29.03	0.34	0.17	all 3	**29.20**	**0.17**	gold standard	

Sample 7

Target	Cq values	Total-ΔCq	Δ best 2	Used Replicates	Mean (Cq)	SD	Evaluation	Comment
**YAP1**	32.79 / 33.50 / 34.18	1.39	0.68	32.79 / 33.50	**33.15**	**0.50**	FFPE accepted	Two replicates within FFPE range (ΔCq = 0.68); third replicate excluded as technical outlier (> 1 Cq).
**MOB1A**	28.02 / 28.13 / 27.97	0.16	0.05	all 3	**28.04**	**0.08**	gold standard	
**TBP**	30.93 / 31.48 / 31.33	0.55	0.15	all 3	**31.25**	**0.29**	FFPE accepted	
**HPRT1-RTP**	29.17 / 29.21 / 29.06	0.15	0.04	all 3	**29.15**	**0.08**	gold standard	

Repetition

Target	Cq values	Total-ΔCq	Δ best 2	Used Replicates	Mean (Cq)	SD	Evaluation	Comment
**MST1**	29.62 / 29.57 / 28.86	0.76	0.05	29.62 / 29.57	**29.60**	**0.04**	gold standard	Δ_best2 < 0.5, outlier >0,5 away from the mean of the Δ_best2 excluded.
**TEAD4**	33.14 / 33.27 / 33.19	0.13	0.05	all 3	**33.20**	**0.06**	gold standard	
**TBP**	31.33 / 31.37 / 31.77	0.44	0.04	all 3	**31.49**	**0.25**	gold standard	
**HPRT1-RTP**	28.37 / 28.29 / 28.18	0.19	0.09	all 3	**28.28**	**0.10**	gold standard	

Block 2

Target	Cq values	Total-ΔCq	Δ best	Used 2 Replicates	Mean (Cq)	SD	Evaluation	Comment
**LATS1**	28.65 / 27.67 / 27.62	1.03	0.05	27.67 / 27.62	**27.65**	**0.04**	gold standard	Two consistent replicates (ΔCq = 0.05); third replicate excluded as technical outlier (> 1 Cq).
**SAV1**	28.04 / 28.10 / 27.80	0.30	0.06	all 3	**27.98**	**0.16**	gold standard	
**HPRT1-RTP**	28.78 / 28.78 / 29.05	0.27	0.00	all 3	**28.87**	**0.15**	gold standard	
**TBP-IDT**	28.69 / 28.39 / 28.30	0.39	0.09	all 3	**28.46**	**0.21**	gold standard	

Sample 8

Target	Cq values	Total-ΔCq	Δ best 2	Used Replicates	Mean (Cq)	SD	Evaluation	Comment
**YAP1**	32.01 / 31.97 / 32.03	0.06	0.04	all 3	**32.00**	**0.03**	gold standard	
**MST1**	29.38 / 29.40 / 29.46	0.08	0.02	all 3	**29.41**	**0.04**	gold standard	
**MOB1A**	27.97 / 27.98 / 27.75	0.23	0.01	all 3	**27.90**	**0.13**	gold standard	
**TEAD4**	32.94 / 33.17 / 33.17	0.23	0.00	all 3	**33.09**	**0.13**	gold standard	
**TBP**	31.03 / 31.09 / 31.18	0.15	0.06	all 3	**31.10**	**0.08**	gold standard	
**HPRT1-RTP**	30.06 / 28.52 / 28.57	1.54	0.05	28.52 / 28.57	**28.55**	**0.04**	gold standard	Two consistent replicates (ΔCq = 0.05); third replicate excluded as technical outlier (> 1 Cq).

Block 2

Target	Cq values	Total-ΔCq	Δ best 2	Used Replicates	Mean (Cq)	SD	Evaluation	Comment
**LATS1**	31.19 / 30.84 / 31.26	0.42	0.07	all 3	**31.10**	**0.22**	gold standard	
**SAV1**	29.41 / 29.47 / 29.88	0.47	0.06	all 3	**29.59**	**0.27**	gold standard	
**HPRT1-RTP**	31.63 / 31.52 / 31.46	0.17	0.06	all 3	**31.54**	**0.09**	gold standard	
**TBP-IDT**	31.18 / 31.25 / -	0.07	0.07	31.18 / 31.25	**31.22**	**0.05**	gold standard	Pipetting error, only two replicates avaible.

Sample 9

Target	Cq values	Total-ΔCq	Δ best 2	Used Replicates	Mean (Cq)	SD	Evaluation	Comment
**YAP1**	30.60 / 30.75 / 30.46	0.29	0.14	all 3	**30.60**	**0.15**	gold standard	
**MST1**	27.15 / 27.03 / 27.12	0.12	0.03	all 3	**27.10**	**0.06**	gold standard	
**MOB1A**	26.09 / 26.19 / 26.33	0.24	0.10	all 3	**26.20**	**0.12**	gold standard	
**TEAD4**	32.11 / 32.30 / 32.06	0.24	0.05	all 3	**32.16**	**0.13**	gold standard	
**TBP**	29.37 / 29.11 / 28.98	0.39	0.13	all 3	**29.15**	**0.20**	gold standard	
**HPRT1-RTP**	27.04 / 27.12 / 27.16	0.12	0.04	all 3	**27.11**	**0.06**	gold standard	

Block 2

Target	Cq values	Total-ΔCq	Δ best 2	Used Replicates	Mean (Cq)	SD	Evaluation	Comment
**LATS1**	28.31 / 27.84 / 27.77	0.54	0.07	all 3	**27.97**	**0.28**	FFPE accepted	
**SAV1**	28.23 / 27.76 / 27.74	0.49	0.02	all 3	**27.91**	**0.28**	gold standard	
**HPRT1-RTP**	29.59 / 28.80 / 30.34	1.54	0.75	30,34 / 29.59	**29.67**	**0.53**	FFPE accepted	Two replicates within FFPE range (ΔCq = 0.75); third replicate excluded as technical outlier (> 1 Cq).
**TBP-IDT**	29.40 / 28.84 / 28.92	0.56	0.08	28.84 / 28.92	**28.88**	**0.06**	gold standard	Δ_best2 < 0.5, outlier >0,5 away from the mean of the Δ_best2 excluded.

Sample 10

Target	Cq values	Total-ΔCq	Δ best 2	Used Replicates	Mean (Cq)	SD	Evaluation	Comment
**YAP1**	34.86 / 36.06 / 35.70	1.20	0.36	36.06 / 35.70	**35.88**	**0.25**	gold standard	Two consistent replicates (ΔCq = 0.36); third replicate excluded as technical outlier (> 1 Cq).
**MST1**	33.02 / 33.00 / –	0.02	0.02	33.02 / 33.00	**33.01**	**0.01**	gold standard	Pipetting error, only two replicates avaible.
**MOB1A**	30.45 / 30.50 / 30.58	0.13	0.05	all 3	**30.51**	**0.07**	gold standard	
**TEAD4**	35.72 / 35.64 / 35.22	0.50	0.08	all 3	**35.53**	**0.26**	gold standard	
**TBP**	35.00 / 35.21 / 35.09	0.21	0.09	all 3	**35.10**	**0.11**	gold standard	
**HPRT1-RTP**	31.05 / 30.79 / 30.85	0.26	0.06	all 3	**30.90**	**0.13**	gold standard	

Block 2

Target	Cq values	Total-ΔCq	Δ best 2	Used Replicates	Mean (Cq)	SD	Evaluation	Comment
**LATS1**	32.79 / 31.72 / 32.18	1.07	0.46	31.72 / 32.18	**31.95**	**0.33**	gold standard	Two consistent replicates (ΔCq = 0.46); third replicate excluded as technical outlier (> 1 Cq).
**SAV1**	31.29 / 31.30 / 31.26	0.04	0.01	all 3	**31.28**	**0.02**	gold standard	
**HPRT1-RTP**	32.13 / 31.79 / 31.86	0.34	0.07	all 3	**31.93**	**0.18**	gold standard	
**TBP**	32.49 / 32.39 / 31.95	0.54	0.10	all 3	**32.32**	**0.29**	FFPE accepted	

Sample 11

Target	Cq values	Total-ΔCq	Δ best 2	Used Replicates	Mean (Cq)	SD	Evaluation	Comment
**YAP1**	33.16 / 33.44	0.28	0.28	33.16 / 33.44	**33.30**	**0.20**	gold standard	Pipetting error, only two replicates avaible.
**MST1**	29.64 / 29.33 / 29.27	0.37	0.06	all 3	**29.41**	**0.20**	gold standard	
**MOB1A**	27.63 / 27.53 / 27.40	0.23	0.10	all 3	**27.52**	**0.12**	gold standard	
**TEAD4**	34.11 / 33.14 / 34.08	0.97	0.03	34.11 / 34.08	**34.10**	**0.02**	gold standard	Δ_best2 < 0.5, outlier >0,5 away from the mean of the Δ_best2 excluded.
**TBP**	31.47 / 31.16 / 30.86	0.61	0.30	all 3	**31.16**	**0.31**	FFPE accepted	
**HPRT1-RTP**	28.37 / 28.49 / 28.41	0.12	0.04	all 3	**28.42**	**0.06**	gold standard	

Block 2

Target	Cq values	Total-ΔCq	Δ best 2	Used Replicates	Mean (Cq)	SD	Evaluation	Comment
**LATS1**	30.52 / 30.35 / 29.88	0.64	0.17	30.52 / 30.35	**30.44**	**0.12**	gold standard	Δ_best2 < 0.5, outlier >0,5 away from the mean of the Δ_best2 excluded.
**SAV1**	30.35 / 29.88 / 29.74	0.61	0.14	29.88 / 29.74	**29.81**	**0.10**	gold standard	Δ_best2 < 0.5, outlier >0,5 away from the mean of the Δ_best2 excluded.
**HPRT1-RTP**	30.47 / 29.90 / 29.58	0.89	0.32	29.90 / 29.58	**29.74**	**0.20**	gold standard	Δ_best2 < 0.5, outlier >0,5 away from the mean of the Δ_best2 excluded.
**TBP-IDT**	31.26 / 30.59 / 30.53	0.73	0.06	30.59 / 30.53	**30.56**	**0.04**	gold standard	Δ_best2 < 0.5, outlier >0,5 away from the mean of the Δ_best2 excluded.

Sample 12

Target	Cq values	Total-ΔCq	Δ best 2	Used Replicates	Mean (Cq)	SD	Evaluation	Comment
**MST1**	26.34 / 26.23 / 26.26	0.11	0.03	all 3	**26.28**	**0.06**	gold standard	
**TBP**	28.46 / 28.43 / 29.87	1.44	0.03	28.46 / 28.43	**28.45**	**0.02**	gold standard	Two consistent replicates (ΔCq = 0.03); third replicate excluded as technical outlier (> 1 Cq).
**HPRT1-RTP**	26.57 / 26.60 / 26.57	0.03	0.00	all 3	**26.58**	**0.02**	gold standard	

Repetition

Target	Cq values	Total-ΔCq	Δ best 2	Used Replicates	Mean (Cq)	SD	Evaluation	Comment
**TEAD4**	31.13 / 31.00 / 31.02	0.13	0.02	all 3	**31.05**	**0.07**	gold standard	
**YAP1**	29.56 / 29.45 / 29.95	0.50	0.11	all 3	**29.65**	**0.27**	gold standard	
**MOB1A**	25.09 / 25.12 / 25.12	0.03	0.00	all 3	**25.11**	**0.02**	gold standard	
**TBP**	28.17 / 28.16 / 28.06	0.11	0.10	all 3	**28.13**	**0.06**	gold standard	
**HPRT1-RTP**	26.28 / 26.31 / 26.45	0.17	0.03	all 3	**26.35**	**0.09**	gold standard	

Block 2

Target	Cq values	Total-ΔCq	Δ best 2	Used Replicates	Mean (Cq)	SD	Evaluation	Comment
**LATS1**	27.40 / 27.34 / 27.35	0.06	0.01	all 3	**27.36**	**0.03**	gold standard	
**SAV1**	27.19 / 27.09 / 27.76	0.67	0.10	27.19 / 27.09	**27.14**	**0.07**	gold standard	Δ_best2 < 0.5, outlier >0,5 away from the mean of the Δ_best2 excluded.
**HPRT1-RTP**	28.78 / 28.79 / 28.64	0.15	0.01	all 3	**28.74**	**0.08**	gold standard	
**TBP-IDT**	28.44 / 28.31 / 28.45	0.14	0.13	all 3	**28.40**	**0.07**	gold standard	

Sample 13

Target	Cq values	Total-ΔCq	Δ best 2	Used Replicates	Mean (Cq)	SD	Evaluation	Comment
**YAP1**	34.41 / 33.14 / 32.87	1.54	0.27	33.14 / 32.87	**33.01**	**0.19**	gold standard	Two consistent replicates (ΔCq = 0.27); third replicate excluded as technical outlier (> 1 Cq).
**MST1**	30.43 / 30.21 / 30.21	0.22	0.00	all 3	**30.28**	**0.13**	gold standard	
**MOB1A**	28.37 / 28.40 / 28.27	0.13	0.10	all 3	**28.35**	**0.07**	gold standard	
**TEAD4**	35.48 / 36.26 / 36.17	0.78	0.09	36.26 / 36.17	**36.22**	**0.06**	gold standard	Δ_best2 < 0.5, outlier >0,5 away from the mean of the Δ_best2 excluded.
**TBP**	32.32 / 31.69 / 31.67	0.65	0.02	31.69 / 31.67	**31.68**	**0.01**	gold standard	Δ_best2 < 0.5, outlier >0,5 away from the mean of the Δ_best2 excluded.
**HPRT1-RTP**	29.73 / 29.14 / 29.68	0.59	0.05	29.73 / 29.68	**29.71**	**0.04**	gold standard	Δ_best2 < 0.5, outlier >0,5 away from the mean of the Δ_best2 excluded.

Block 2

Target	Cq values	Total-ΔCq	Δ best 2	Used Replicates	Mean (Cq)	SD	Evaluation	Comment
**LATS1**	31.58 / 31.32 / 31.59	0.27	0.01	all 3	**31.50**	**0.12**	gold standard	
**SAV1**	30.08 / 30.30 / 30.68	0.60	0.22	all 3	**30.35**	**0.31**	FFPE accepted	
**HPRT1**	32.36 / 32.50 / 32.05	0.45	0.31	all 3	**32.30**	**0.23**	gold standard	
**TBP**	31.95 / 32.19 / 32.10	0.24	0.14	all 3	**32.08**	**0.12**	gold standard	

Sample 14

Target	Cq values	Total-ΔCq	Δ best 2	Used Replicates	Mean (Cq)	SD	Evaluation	Comment
**MST1**	29.47 / 30.73 / 29.26	1.47	0.21	29.47 / 29.26	**29.36**	**0.15**	gold standard	Two consistent replicates (ΔCq = 0.21); third replicate excluded as technical outlier (> 1 Cq).
**MOB1A**	27.58 / 27.57 / 27.61	0.04	0.01	all 3	**27.59**	**0.02**	gold standard	
**TEAD4**	33.78 / 33.52 / 34.43	0.91	0.26	33.78 / 33.52	**33.65**	**0.18**	gold standard	Δ_best2 < 0.5, outlier >0,5 away from the mean of the Δ_best2 excluded.
**TBP**	32.11 / 32.14 / 31.83	0.31	0.03	all 3	**32.03**	**0.17**	gold standard	
**HPRT1-RTP**	28.04 / 28.02 / 28.05	0.03	0.01	all 3	**28.04**	**0.02**	gold standard	

Repetition

Target	Cq values	Total-ΔCq	Δ best 2	Used Replicates	Mean (Cq)	SD	Evaluation	Comment
**YAP1**	30.79 / 30.69 / 30.80	0.11	0.01	all 3	**30.76**	**0.06**	gold standard	
**TBP**	31.07 / 30.91 / 30.91	0.16	0.00	all 3	**30.96**	**0.09**	gold standard	
**HPRT1-RTP**	27.32 / 27.31 / 27.24	0.08	0.01	all 3	**27.29**	**0.04**	gold standard	

Block 2

Target	Cq values	Total-ΔCq	Δ best 2	Used Replicates	Mean (Cq)	SD	Evaluation	Comment
**LATS1**	30.19 / 30.14 / 32.68	2.54	0.05	30.19 / 30.14	**30.17**	**0.04**	gold standard	Two consistent replicates (ΔCq = 0.05); third replicate excluded as technical outlier (> 1 Cq)..
**SAV1**	28.46 / 28.36 / 28.38	0.10	0.02	all 3	**28.40**	**0.05**	gold standard	
**HPRT1**	29.84 / 29.65 / 29.85	0.20	0.19	all 3	**29.78**	**0.11**	gold standard	
**TBP**	31.18 / 30.71 / 34.08	3.37	0.47	31.18 / 30.71	**30.95**	**0.33**	gold standard	Two consistent replicates (ΔCq = 0.47); third replicate excluded as technical outlier (> 1 Cq)..

Sample 15

Target	Cq values	Total-ΔCq	Δ best 2	Used Replicates	Mean (Cq)	SD	Evaluation	Comment
**YAP1**	33.78 / 33.62 / 33.85	0.23	0.07	all 3	**33.75**	**0.12**	gold standard	
**MST1**	32.09 / 31.63 / 31.79	0.46	0.16	all 3	**31.84**	**0.23**	gold standard	
**MOB1A**	29.98 / 30.27 / 30.48	0.50	0.21	all 3	**30.24**	**0.25**	gold standard	
**TEAD4**	34.14 / 33.87 / 34.36	0.49	0.22	all 3	**34.12**	**0.25**	gold standard	
**TBP**	33.44 / 34.00 / 34.41	0.97	0.41	34.41 / 34.00	**34.21**	**0.29**	gold standard	Δ_best2 < 0.5, outlier >0,5 away from the mean of the Δ_best2 excluded.
**HPRT1-RTP**	31.53 / 31.43 / 31.26	0.27	0.10	all 3	**31.41**	**0.14**	gold standard	

Block 2

Target	Cq values	Total-ΔCq	Δ best 2	Used Replicates	Mean (Cq)	SD	Evaluation	Comment
**LATS1**	31.17 / 31.11 / 31.21	0.10	0.10	all 3	**31.16**	**0.05**	gold standard	
**SAV1**	29.40 / 29.30 / 29.21	0.19	0.09	all 3	**29.30**	**0.10**	gold standard	
**HPRT1**	32.64 / 32.90 / 33.26	0.62	0.26	all 3	**32.93**	**0.31**	FFPE accepted	
**TBP**	32.15 / 32.33 / 32.31	0.18	0.16	all 3	**32.26**	**0.09**	gold standard	

Sample 16

Target	Cq values	Total-ΔCq	Δ best 2	Used Replicates	Mean (Cq)	SD	Evaluation	Comment
**YAP1**	32.03 / 31.65 / 31.54	0.49	0.11	all 3	**31.74**	**0.26**	gold standard	
**MST1**	28.03 / 27.98 / 28.09	0.11	0.05	all 3	**28.03**	**0.06**	gold standard	
**MOB1A**	26.49 / 26.36 / 26.50	0.14	0.01	all 3	**26.45**	**0.08**	gold standard	
**TEAD4**	32.16 / 32.14 / 32.25	0.11	0.02	all 3	**32.18**	**0.06**	gold standard	
**TBP**	30.43 / 30.21 / 30.33	0.22	0.12	all 3	**30.32**	**0.11**	gold standard	
**HPRT1-RTP**	27.80 / 27.73 / 27.71	0.09	0.02	all 3	**27.75**	**0.05**	gold standard	

Block 2

Target	Cq values	Total-ΔCq	Δ best 2	Used Replicates	Mean (Cq)	SD	Evaluation	Comment
**LATS1**	31.58 / 31.05 / 30.95	0.63	0.10	31.05 / 30.95	**31.00**	**0,07**	gold standard	Δ_best2 < 0.5, outlier >0,5 away from the mean of the Δ_best2 excluded.
**SAV1**	30.93 / 30.21 / 30.36	0.72	0.15	30.21 / 30.36	**30.29**	**0.11**	gold standard	Δ_best2 < 0.5, outlier >0,5 away from the mean of the Δ_best2 excluded.
**HPRT1**	32.49 / 32.21 / 32.15	0.34	0.06	all 3	**32.28**	**0.18**	gold standard	
**TBP**	35.01 / 32.28 / 32.09	2.92	0.19	32.28 / 32.09	**32.19**	**0.13**	gold standard	Two consistent replicates (ΔCq = 0.19); third replicate excluded as technical outlier (> 1 Cq).

Sample 17

Target	Cq values	Total-ΔCq	Δ best 2	Used Replicates	Mean (Cq)	SD	Evaluation	Comment
**YAP1**	31.19 / 31.22 / 30.96	0.26	0.03	all 3	**31.12**	**0.14**	gold standard	
**MST1**	29.63 / 29.68 / 29.44	0.24	0.05	all 3	**29.58**	**0.13**	gold standard	
**MOB1A**	27.51 / 27.56 / 27.47	0.09	0.04	all 3	**27.51**	**0.05**	gold standard	
**TEAD4**	33.41 / 33.11 / 33.30	0.30	0.11	all 3	**33.27**	**0.15**	gold standard	
**TBP**	31.90 / 31.81 / 31.64	0.26	0.17	all 3	**31.78**	**0.13**	gold standard	
**HPRT1-RTP**	28.54 / 28.46 / 28.36	0.18	0.10	all 3	**28.45**	**0.09**	gold standard	

Block 2

Target	Cq values	Total-ΔCq	Δ best 2	Used Replicates	Mean (Cq)	SD	Evaluation	Comment
**LATS1**	29.43 / 29.36 / 29.25	0.18	0.11	all 3	**29.35**	**0.09**	gold standard	
**SAV1**	27.80 / 27.90 / 27.79	0.11	0.01	all 3	**27.83**	**0.06**	gold standard	
**HPRT1**	30.24 / 30.19 / 30.06	0.18	0.13	all 3	**30.16**	**0.09**	gold standard	
**TBP**	30.48 / 31.01 / 30.63	0.53	0.15	all 3	**30.70**	**0.27**	FFPE accepted	

Sample 18

Target	Cq values	Total-ΔCq	Δ best 2	Used Replicates	Mean (Cq)	SD	Evaluation	Comment
**YAP1**	28.54 / 28.62 / 28.43	0.19	0.09	all 3	**28.53**	**0.10**	gold standard	
**MST1**	28.07 / 25.96 / 26.28	2.11	0.32	25.96 / 26.28	**26.12**	**0.23**	gold standard	Two consistent replicates (ΔCq = 0.32); third replicate excluded as technical outlier (> 1 Cq).
**MOB1A**	25.33 / 25.50 / 25.38	0.17	0.05	all 3	**25.40**	**0.09**	gold standard	
**TEAD4**	31.98 / 31.71 / 32.09	0.38	0.27	all 3	**31.93**	**0.19**	gold standard	
**TBP**	29.17 / 29.18 / 29.16	0.02	0.01	all 3	**29.17**	**0.01**	gold standard	
**HPRT1-RTP**	27.30 / 27.43 / 27.26	0.17	0.04	all 3	**27.33**	**0.09**	gold standard	

Block 2

Target	Cq values	Total-ΔCq	Δ best 2	Used Replicates	Mean (Cq)	SD	Evaluation	Comment
**LATS1**	27.16 / 27.02 / 26.94	0.22	0.08	all 3	**27.04**	**0.11**	gold standard	
**SAV1**	25.44 / 25.63 / 25.67	0.23	0.23	all 3	**25.58**	**0.13**	gold standard	
**HPRT1**	29.11 / 29.24 / 29.82	0.71	0.13	29.11 / 29.24	**29.18**	**0.09**	gold standard	Δ_best2 < 0.5, outlier >0,5 away from the mean of the Δ_best2 excluded.
**TBP**	28.42 / 28.25 / 28.16	0.26	0.09	all 3	**28.28**	**0.13**	gold standard	.

Sample 19

Target	Cq values	Total-ΔCq	Δ best 2	Used Replicates	Mean (Cq)	SD	Evaluation	Comment
**YAP1**	30.89 / 31.41 / 30.56	0.85	0.33	30.89 / 30.56	**30.73**	**0.23**	gold standard	Δ_best2 < 0.5, outlier >0,5 away from the mean of the Δ_best2 excluded.
**MST1**	29.44 / 29.46 / 29.59	0.15	0.02	all 3	**29.50**	**0.08**	gold standard	
**MOB1A**	27.87 / 27.91 / 28.02	0.15	0.04	all 3	**27.93**	**0.08**	gold standard	
**TEAD4**	32.43 / 31.73 / 31.94	0.70	0.21	31.73 / 31.94	**31.84**	**0.15**	gold standard	Δ_best2 < 0.5, outlier >0,5 away from the mean of the Δ_best2 excluded.
**TBP**	31.74 / 31.59 / 32.05	0.46	0.15	all 3	**31.79**	**0.23**	gold standard	
**HPRT1-RTP**	28.64 / 28.54 / 28.40	0.24	0.14	all 3	**28.53**	**0.12**	gold standard	

Block 2

Target	Cq values	Total-ΔCq	Δ best 2	Used Replicates	Mean (Cq)	SD	Evaluation	Comment
**LATS1**	30.30 / 29.97 / 29.80	0.50	0.17	all 3	**30.02**	**0.25**	gold standard	
**SAV1**	27.15 / 27.11 / 27.24	0.13	0.13	all 3	**27.17**	**0.07**	gold standard	
**HPRT1**	29.92 / 30.01 / 29.90	0.11	0.11	all 3	**29.94**	**0.06**	gold standard	
**TBP**	31.02 / 30.49 / 30.73	0.53	0.24	all 3	**30.75**	**0.27**	FFPE accepted	

Sample 20

Target	Cq values	Total-ΔCq	Δ best 2	Used Replicates	Mean (Cq)	SD	Evaluation	Comment
**LATS1**	30.18 / 29.77 / 30.31	0.54	0.13	all 3	**30.09**	**0.28**	FFPE accepted	
**SAV1**	27.20 / 27.19 / 27.36	0.17	0.01	all 3	**27.25**	**0.09**	gold standard	
**YAP1**	30.99 / 30.44 / 30.71	0.55	0.28	all 3	**30.71**	**0.28**	FFPE accepted	
**TEAD4**	32.55 / 32.46 / 32.71	0.25	0.09	all 3	**32.57**	**0.13**	gold standard	
**MST1**	30.08 / 29.04 / 30.05	1.04	0.03	30.08 / 30.05	**30.07**	**0.02**	gold standard	Δ_best2 < 0.5, outlier >0,5 away from the mean of the Δ_best2 excluded.
**MOB1A**	28.20 / 27.79 / 30.14	2.35	0.41	27.79 / 28.20	**28.00**	**0.29**	gold standard	Δ_best2 < 0.5, outlier >0,5 away from the mean of the Δ_best2 excluded.
**HPRT1-RTP**	31.65 / 31.47 / 31.49	0.18	0.02	all 3	**31.54**	**0.10**	gold standard	
**TBP**	31.02 / 31.20 / 31.15	0.18	0.05	all 3	**31.12**	**0.09**	gold standard	

Sample 21

Target	Cq values	Total-ΔCq	Δ best 2	Used Replicates	Mean (Cq)	SD	Evaluation	Comment
**YAP1**	32.38 / 33.11 / 32.32	0.79	0.06	32.38 / 32.32	**32.35**	**0.04**	gold standard	Δ_best2 < 0.5, outlier >0,5 away from the mean of the Δ_best2 excluded.
**MST1**	32.23 / 31.60 / 31.05	1.18	0.55	31.60 / 31.05	**31.33**	**0.39**	FFPE accepted	Two replicates within FFPE range (ΔCq = 0.55); third replicate excluded as technical outlier (> 1 Cq).
**MOB1A**	29.35 / 29.70 / 29.47	0.35	0.12	all 3	**29.51**	**0.18**	gold standard	
**TEAD4**	34.49 / 34.17 / 33.93	0.56	0.24	all 3	**34.20**	**0.28**	gold standard	
**TBP**	33.94 / 33.71 / 33.66	0.28	0.05	all 3	**33.77**	**0.15**	gold standard	
**HPRT1-RTP**	30.44 / 30.44 / 30.59	0.15	0.00	all 3	**30.49**	**0.09**	gold standard	

Block 2

Target	Cq values	Total-ΔCq	Δ best 2	Used Replicates	Mean (Cq)	SD	Evaluation	Comment
**LATS1**	30.97 / 31.30 / 31.05	0.33	0.07	all 3	**31.11**	**0.17**	☑ Goldstandard?	
**SAV1**	28.13 / 28.03 / 27.88	0.25	0.10	all 3	**28.01**	**0.13**	☑ Goldstandard?	
**HPRT1-RTP**	31.83 / 32.64 / 31.59	1.05	0.24	31.83 / 31.59	**31.71**	0.17	☑ Goldstandard?	Δ_best2 < 0.5, outlier >0,5 away from the mean of the Δ_best2 excluded.
**TBP**	32.02 / 32.15 / 32.04	0.13	0.02	all 3	**32.07**	**0.07**	☑ Goldstandard?	

Sample 22

Target	Cq values	Total-ΔCq	Δ best 2	Used Replicates	Mean (Cq)	SD	Evaluation	Comment
**YAP1**	32.30 / 32.39 / 32.44	0.14	0.09	all 3	**32.38**	**0.07**	gold standard	
**MST1**	31.07 / 31.21 / 31.05	0.16	0.02	all 3	**31.11**	**0.09**	gold standard	
**MOB1A**	29.35 / 28.89 / –	–	0.46	29.35/28.89	**29.12**	**0.33**	gold standard	Pipetting error, only two replicates avaible.
**TEAD4**	32.45 / 32.34 / 32.13	0.32	0.21	all 3	**32.31**	**0.16**	gold standard	
**TBP**	32.52 / 32.18 / 32.48	0.34	0.30	all 3	**32.39**	**0.18**	gold standard	
**HPRT1-RTP**	29.47 / 29.39 / 29.72	0.33	0.08	all 3	**29.53**	**0.18**	gold standard	

Block 2

Target	Cq values	Total-ΔCq	Δ best 2	Used Replicates	Mean (Cq)	SD	Evaluation	Comment
**LATS1**	30.34 / 30.20 / 30.02	0.32	0.14	all 3	**30.19**	**0.16**	gold standard	
**SAV1**	27.79 / 27.93 / 27.77	0.16	0.02	all 3	**27.83**	**0.09**	gold standard	
**HPRT1-RTP**	30.72 / 30.61 / 30.98	0.37	0.11	all 3	**30.77**	**0.19**	gold standard	
**TBP**	30.97 / 31.03 / 30.91	0,06	0.06	all 3	**30.97**	**0.06**	gold standard	

Sample 23

Target	Cq values	Total-ΔCq	Δ best 2	Used Replicates	Mean (Cq)	SD	Evaluation	Comment
**YAP1**	33.71 / 33.84 / 33.52	0.32	0.19	all 3	**33.69**	**0.16**	gold standard	
**MOB1A**	28.87 / 29.00 / 28.63	0.37	0.24	all 3	**28.83**	**0.19**	gold standard	
**TEAD4**	32.96 / 32.03 / 31.60	1.36	0.43	32.03 / 31.60	**31.82**	**0.30**	gold standard	Two consistent replicates (ΔCq = 0.43); third replicate excluded as technical outlier (> 1 Cq).
**TBP**	35.00 / 33.60 / 34.27	1.40	0.67	33,60 / 34.27	**33.94**	**0.47**	FFPE accepted	Δ_best2 < 0.5, outlier >0,5 away from the mean of the Δ_best2 excluded.
**HPRT1-RTP**	30.71 / 30.69 / 30.73	0.04	0.02	all 3	**30.71**	**0.02**	gold standard	

Repetition

Target	Cq values	Total-ΔCq	Δ best 2	Used Replicates	Mean (Cq)	SD	Evaluation	Comment
**MST1**	34.54 / 34.80	0.26	0.26	both	**34.67**	**0.18**	gold standard	Pipetting error, only two replicates avaible.
**TBP**	33.76 / 33.37 / 33.89	0.52	0.13	all 3	**33.67**	**0.27**	FFPE accepted	
**HPRT1-RTP**	30.17 / 30.14 / 30.10	0.07	0.03	all 3	**30.14**	**0.04**	gold standard	

Block 2

Target	Cq values	Total-ΔCq	Δ best 2	Used Replicates	Mean (Cq)	SD	Evaluation	Comment
**LATS1**	34.57 / 34.96 / 34.76	0.39	0.19	all 3	**34.76**	**0.20**	gold standard	
**SAV1**	32.60 / 32.76 / 32.56	0.20	0.04	all 3	**32.64**	**0.10**	gold standard	
**HPRT1-RTP**	32.62 / 32.79 / 32.80	0.18	0.01	all 3	**32.74**	**0.10**	gold standard	
**TBP**	33.40 / 33.29 / 33.07	0.33	0.11	all 3	**33.25**	**0.17**	gold standard	

Sample 24

Target	Cq values	Total-ΔCq	Δ best 2	Used Replicates	Mean (Cq)	SD	Evaluation	Comment
**YAP1**	33.71 / 34.02 / 33.85	0.31	0.14	all 3	**33.86**	**0.16**	gold standard	
**MST1**	31.62 / 31.52 / 31.67	0.15	0.10	all 3	**31.60**	**0.08**	gold standard	
**MOB1A**	29.00 / 29.17 / 29.23	0.23	0.17	all 3	**29.13**	**0.12**	gold standard	
**TEAD4**	33.17 / 33.83 / 33.42	0.66	0.25	33.17 / 33.42	**33.30**	**0.18**	gold standard	Δ_best2 < 0.5, outlier >0,5 away from the mean of the Δ_best2 excluded.
**TBP**	33.30 / 32.72 / 32.80	0.58	0.08	32.72 / 32.80	**32.76**	**0.06**	gold standard	Δ_best2 < 0.5, outlier >0,5 away from the mean of the Δ_best2 excluded.
**HPRT1-RTP**	29.74 / 29.76 / 29.93	0.19	0.02	all 3	**29.81**	**0.11**	gold standard	

Block 2

Target	Cq values	Total-ΔCq	Δ best 2	Used Replicates	Mean (Cq)	SD	Evaluation	Comment
**LATS1**	29.64 / 29.41 / 29.46	0.23	0.05	all 3	**29.50**	**0.12**	gold standard	
**SAV1**	29.16 / 29.18 / 28.82	0.36	0.02	all 3	**29.05**	**0.20**	gold standard	
**HPRT1-RTP**	29.90 / 29.53 / 29.47	0.43	0.06	all 3	**29.63**	**0.23**	gold standard	
**TBP**	30.16 / 30.41 / 30.15	0.26	0.01	all 3	**30.24**	**0.15**	gold standard	

Sample 25

Target	Cq values	Total-ΔCq	Δ best 2	Used Replicates	Mean (Cq)	SD	Evaluation	Comment
**YAP1**	34.57 / 35.70 / 34.72	1.13	0.15	34.57 / 34.72	**34.65**	**0.63**	gold standard	Δ_best2 < 0.5, outlier >0,5 away from the mean of the Δ_best2 excluded.
**MST1**	32.11 / 32.53 / 32.57	0.46	0.04	all 3	**32.40**	**0.25**	gold standard	
**MOB1A**	29.75 / 29.58 / 29.86	0.28	0.17	all 3	**29.73**	**0.14**	gold standard	
**TEAD4**	31.70 / 31.73 / 31.59	0.14	0.11	all 3	**31.67**	**0.08**	gold standard	
**TBP**	34.48 / 34.14 / 34.08	0.40	0.06	all 3	**34.23**	**0.21**	gold standard	
**HPRT1-RTP**	28.73 / 28.67 / 29.11	0.44	0.06	all 3	**28.84**	**0.24**	gold standard	

Block 2

Target	Cq values	Total-ΔCq	Δ best 2	Used Replicates	Mean (Cq)	SD	Evaluation	Comment
**LATS1**	32.29 / 32.56 / 31.71	0.85	0.27	32.29 / 32.56	**32.43**	**0.19**	gold standard	Δ_best2 < 0.5, outlier >0,5 away from the mean of the Δ_best2 excluded.
**SAV1**	30.72 / 30.09 / 30.06	0.66	0.03	30.09 / 30.06	**30.08**	**0.02**	gold standard	Δ_best2 < 0.5, outlier >0,5 away from the mean of the Δ_best2 excluded.
**HPRT1-RTP**	32.03 / 31.45 / 31.47	0.58	0.02	31.45 / 31.47	**31.46**	**0.01**	gold standard	Δ_best2 < 0.5, outlier >0,5 away from the mean of the Δ_best2 excluded.
**TBP**	32.26 / 32.31 / 32.55	0.29	0.05	all 3	**32.37**	**0.16**	gold standard	

Sample 26

Target	Cq values	Total-ΔCq	Δ best 2	Used Replicates	Mean (Cq)	SD	Evaluation	Comment
**YAP1**	33.76 / 33.64 / 33.98	0.34	0.12	all 3	**33.79**	**0.17**	gold standard	
**MST1**	32.27 / 32.35 / 32.23	0.12	0.04	all 3	**32.28**	**0.06**	gold standard	
**MOB1A**	30.18 / 30.07 / 30.25	0.18	0.11	all 3	**30.17**	**0.09**	gold standard	
**TEAD4**	32.55 / 32.28 / 32.44	0.27	0.16	all 3	**32.42**	**0.14**	gold standard	
**TBP**	34.29 / 33.65 / 34.12	0.64	0.17	34.29 / 34.12	**34.21**	**0.12**	gold standard	Δ_best2 < 0.5, outlier >0,5 away from the mean of the Δ_best2 excluded.
**HPRT1-RTP**	30.61 / 30.58 / 31.34	0.76	0.03	30.61 / 30.58	**30.60**	**0,02**	gold standard	Δ_best2 < 0.5, outlier >0,5 away from the mean of the Δ_best2 excluded.

Block 2

Target	Cq values	Total-ΔCq	Δ best 2	Used Replicates	Mean (Cq)	SD	Evaluation	Comment
**LATS1**	35.74 / 35.27	0.47	0.47	both	**35.51**	**0.33**	gold standard	Pipetting error, only two replicates avaible.
**SAV1**	32.76 / 33.96 / 33.21	1.2	0.45	32.76 / 33.21	**32.99**	**0.32**	gold standard	Δ_best2 < 0.5, outlier >0,5 away from the mean of the Δ_best2 excluded.
**HPRT1-RTP**	35.76 / 35.25 / 35.16	0.6	0.09	35.25 / 35.16	**35.21**	**0.06**	gold standard	Δ_best2 < 0.5, outlier >0,5 away from the mean of the Δ_best2 excluded.
**TBP**	35.27 / 35.38 / 36.01	0.74	0.11	35.27 / 35.38	**35.33**	**0,08**	gold standard	Δ_best2 < 0.5, outlier >0,5 away from the mean of the Δ_best2 excluded.
