# Supplementary material for: Subtype-Dependent Expression Patterns of Core Hippo Pathway Components in Thymic Epithelial Tumors (TETs): An RT-qPCR Study
Source: Biomedicines. 2026 Jan 29;14(2):305. doi: 10.3390/biomedicines14020305 (PMC12937678; doi:10.3390/biomedicines14020305)
Supplement: Supplementary file 1 [file biomedicines-14-00305-s001.zip › Table S15 IHC antibodies, retrieval, controls, and scoring.pdf]

Table S15. IHC antibodies, retrieval, controls, and scoring. 2-µm FFPE sections; Autostainer Link48 (Dako/Agilent) with EnVision FLEX detection. Antibodies were titrated within manufacturer-recommended ranges and optimized using Dako Target Retrieval solutions (pH 9.0 and pH 6.1) as documented in the validation forms. Positive control tissues were included in each run. IHC scoring was performed by an experienced pathologist (L.G.) blinded to clinical information; ≥1000 tumor cells were evaluated per case, and nuclear and cytoplasmic staining were recorded separately as % positive tumor cells and intensity (0–3).

Target	Antibody (clone)	Host / clonality	Supplier	Catalog / RRID (if available)	Working dilution (study)	Retrieval tested/used (summary)	Primary incubation (summary)	Positive control tissue (each run)
**YAP1**	1A12	Mouse monoclonal	Invitrogen (Thermo Fisher)	MA5-17200	1:1000	TR pH 9.0 and pH 6.1 tested	30 min (validated)	Prostate
**Active YAP1 (AYAP)**	EPR19812	Rabbit monoclonal	Abcam	ab205270	1:2000	TR pH 9.0 and pH 6.1 tested	30 min (validated)	Human breast cancer
**TAZ (WWTR1)**	2A12A10	Mouse monoclonal	Proteintech	66500-1-lg	1:600	TR pH 9.0 and/or pH 6.1 tested (validated)	30 min (validated)	Human breast cancer
**TEAD4**	polyclonal	Rabbit polyclonal	Invitrogen (Thermo Fisher)	PA5-21977	1:500	TR pH 9.0 and pH 6.1 tested	30 min (validated)	Placenta
**MST1 (STK4)**	polyclonal	Rabbit polyclonal	Invitrogen (Thermo Fisher)	PA5-22015	1:250	TR pH 9.0 and pH 6.1 tested	30 min (validated)	Human adenocarcinoma
**SAV1**	OTI2B7	Mouse monoclonal	Invitrogen (Thermo Fisher)	MA5-26689	1:100	TR pH 9.0 and pH 6.1 tested	540 min (validated)	Kidney
**LATS1**	polyclonal	Rabbit polyclonal	Proteintech	17049-1-AP	1:200	TR pH 9.0 and pH 6.1 tested	30 min (validated)	Human breast cancer
**MOB1A**	polyclonal	Rabbit polyclonal	Invitrogen (Thermo Fisher)	PA5-98902	1:350	TR pH 9.0 and pH 6.1 tested	40 min (validated)	Human breast cancer
