# Supplementary material for: Subtype-Dependent Expression Patterns of Core Hippo Pathway Components in Thymic Epithelial Tumors (TETs): An RT-qPCR Study
Source: Biomedicines. 2026 Jan 29;14(2):305. doi: 10.3390/biomedicines14020305 (PMC12937678; doi:10.3390/biomedicines14020305)
Supplement: Supplementary file 1 [file biomedicines-14-00305-s001.zip › Table S16 Relative gene expression in relation to IHC results on a per-sample basis. .pdf]

Table S16. Relative gene expression in relation to IHC results on a per-sample basis. This table summarizes the immunohistochemical evaluation of nuclear and cytoplasmic staining patterns. For each tumor sample, the percentage of positively stained tumor cells was assessed separately for the nucleus and cytoplasm. Staining intensity was scored on a scale from 0 to 3, where 0 = negative, 1 = weak, 2 = moderate, and 3 = strong staining. The parameters "Nuclear (%)" and "Cytoplasmic (%)" represent the proportion of tumor cells showing nuclear or cytoplasmic positivity, while "Staining Intensity" reflects the corresponding qualitative assessment of signal strength for each compartment.

MST1						
**Sample**	**FC**	**Log2FC**	**IHC**			
**1 (NG)**	1,42	0,51				
**3 (NG)**	0,70	-0,51	Nuclear	Staining Intensity	Cytoplasmic	Staining Intensity
**4 (A)**	2,66	1,41	0	0	5	1
**5 (A)**	0,90	-0,15	0	0	60	2
**6 (A)**	0,50	-1,01	0	0	30	0,5
**7 (B1)**	0,58	-0,78	0	0	15	1
**8 (B1)**	0,64	-0,65	0	0	20	1
**9 (B1)**	0,98	-0,03	0	0	5	0,5
**10 (B1)**	0,48	-1,07	0	0	3	1
**11 (B1)**	0,62	-0,68	0	0	5	1
**12 (B2)**	1,13	0,17	0	0	30	1
**13 (B2)**	0,64	-0,65	0	0	20	1
**14 (B2)**	0,76	-0,39	0	0	40	1
**15 (B2)**	0,94	-0,09	0	0	5	1
**16 (B2)**	0,96	-0,06	0	0	10	1
**17 (B3)**	0,69	-0,53	0	0	95	2
**18 (B3)**	2,10	1,07	0	0	1	1
**19 (B3)**	0,76	-0,40	0	0	15	0,5
**20 (B3)**	1,15	0,20	0	0	1	1
**21 (B3)**	0,83	-0,26	0	0	90	1
**22 (TC)**	0,43	-1,21	0	0	90	0,5
**23 (TC)**	0,07	-3,83	0	0	80	1
**24 (TC)**	0,38	-1,38	0	0	90	2
**25 (TC)**	0,26	-1,93	0	0	1	1
**26 (TC)**	0,52	-0,94	0	0	80	1,5

SAV1						
**Sample**	**FC**	**Log2FC**	**IHC**			
			Nuclear	Staining Intensity	Cytoplasmic	Staining Intensity
**1 (NG)**	0,92	-0,13				
**3 (NG)**	1,09	0,13				
**4 (A)**	2,52	1,34	0	0	100	2
**5 (A)**	14,07	3,82	0	0	90	2
**6 (A)**	4,76	2,25	0	0	100	3
**7 (B1)**	0,72	-0,48	0	0	100	3
**8 (B1)**	1,54	0,63	0	0	80	2
**9 (B1)**	1,15	0,20	0	0	100	3
**10 (B1)**	0,80	-0,32	0	0	100	3
**11 (B1)**	0,56	-0,82	0	0	80	2
**12 (B2)**	1,20	0,26	0	0	100	3
**13 (B2)**	1,60	0,67	0	0	100	3
**14 (B2)**	1,74	0,80	0	0	100	3
**15 (B2)**	4,38	2,13	0	0	100	3
**16 (B2)**	1,72	0,78	0	0	100	3
**17 (B3)**	2,70	1,44	0	0	100	3
**18 (B3)**	3,96	1,99	0	0	100	3
**19 (B3)**	4,03	2,01	0	0	100	3
**20 (B3)**	7,54	2,92	0	0	100	3
**21 (B3)**	6,57	2,72	0	0	100	3
**22 (TC)**	3,67	1,88	0	0	100	3
**23 (TC)**	0,57	-0,81	0	0	100	3
**24 (TC)**	0,82	-0,28	0	0	100	3
**25 (TC)**	1,59	0,67	0	0	100	3
**26 (TC)**	2,17	1,12	0	0	100	3

LATS1						
**Sample**	**FC**	**Log2FC**	**IHC**			
			Nuclear	Staining Intensity	Cytoplasmic	Staining Intensity
**1 (NG)**	1,12	0,16				
**3 (NG)**	0,89	-0,16				
**4 (A)**	1,09	0,13	0	0	100	2
**5 (A)**	2,05	1,04	0	0	100	2
**6 (A)**	1,09	0,13	0	0	80	1
**7 (B1)**	0,94	-0,09	0	0	70	1
**8 (B1)**	0,56	-0,83	0	0	40	1
**9 (B1)**	1,15	0,20	0	0	80	1
**10 (B1)**	0,52	-0,93	0	0	90	2
**11 (B1)**	0,38	-1,40	0	0	70	2
**12 (B2)**	1,08	0,10	0	0	90	1
**13 (B2)**	0,75	-0,42	0	0	100	3
**14 (B2)**	0,53	-0,91	0	0	100	1
**15 (B2)**	1,26	0,33	0	0	60	1
**16 (B2)**	1,09	0,13	0	0	40	1
**17 (B3)**	0,98	-0,03	0	0	100	3
**18 (B3)**	1,50	0,59	0	0	100	3
**19 (B3)**	0,58	-0,78	0	0	100	3
**20 (B3)**	1,10	0,14	0	0	80	1
**21 (B3)**	0,80	-0,32	0	0	95	1
**22 (TC)**	0,74	-0,43	0	0	100	2
**23 (TC)**	0,14	-2,87	0	0	100	1,5
**24 (TC)**	0,63	-0,67	0	0	100	3
**25 (TC)**	0,33	-1,62	0	0	100	2
**26 (TC)**	0,39	-1,34	0	0	90	1

MOB1A						
**Sample**	**FC**	**Log2FC**	**IHC**			
**1 (NG)**	0,95	-0,07				
**3 (NG)**	1,05	0,07	Nuclear	Staining Intensity	Cytoplasmic	Staining Intensity
**4 (A)**	1,68	0,75	0	0	100	3
**5 (A)**	1,06	0,08	0	0	100	3
**6 (A)**	1,17	0,22	0	0	100	3
**7 (B1)**	0,91	-0,13	0	0	95	3
**8 (B1)**	0,78	-0,37	0	0	100	3
**9 (B1)**	0,78	-0,36	0	0	90	3
**10 (B1)**	1,15	0,20	0	0	100	3
**11 (B1)**	0,98	-0,02	0	0	100	3
**12 (B2)**	0,89	-0,16	0	0	90	3
**13 (B2)**	1,04	0,05	0	0	70	1
**14 (B2)**	1,11	0,15	0	0	100	2
**15 (B2)**	1,21	0,28	0	0	100	2
**16 (B2)**	1,22	0,29	0	0	100	3
**17 (B3)**	1,24	0,31	0	0	100	3
**18 (B3)**	1,47	0,56	0	0	100	2
**19 (B3)**	0,96	-0,06	0	0	100	3
**20 (B3)**	2,05	1,04	0	0	80	2
**21 (B3)**	1,25	0,33	0	0	100	3
**22 (TC)**	0,73	-0,45	0	0	100	3
**23 (TC)**	2,30	1,20	0	0	100	3
**24 (TC)**	0,91	-0,14	0	0	100	3
**25 (TC)**	0,71	-0,49	0	0	100	3
**26 (TC)**	0,96	-0,06	0	0	100	3

	YAP1							active YAP (AYAP)			
**Sample**	**FC**	**Log2FC**	**IHC**					**IHC**			
			Nuclear	Staining Intensity	Cytoplasmic	Staining Intensity		Nuclear	Staining Intensity	Cytoplasmic	Staining Intensity
**1 (NG)**	0,91	-0,13									
**3 (NG)**	1,10	0,13									
**4 (A)**	3,98	1,99	90	2	60	2		90	2	100	2
**5 (A)**	10,76	3,43	70	3	95	3		90	2	100	2
**6 (A)**	13,11	3,71	95	3	100	2		100	3	95	1
**7 (B1)**	1,08	0,11	40	2	0	0		50	2	5	1
**8 (B1)**	1,84	0,88	25	2	0	0		60	3	0	0
**9 (B1)**	1,50	0,59	25	1	0	0		40	2	0	0
**10 (B1)**	1,13	0,18	80	2	0	0		60	2	10	1
**11 (B1)**	0,73	-0,45	5	1	30	1		20	1	40	1
**12 (B2)**	1,57	0,65	55	2	0	0		90	2	0	0
**13 (B2)**	1,67	0,74	20	1	10	1		20	1	10	1
**14 (B2)**	2,68	1,42	30	2	60	1		30	1	40	1
**15 (B2)**	4,34	2,12	80	2	0	0		80	2	5	1
**16 (B2)**	1,28	0,35	80	1	5	1		70	2	5	1
**17 (B3)**	4,15	2,05	20	1	60	1		10	1	40	1 (0,5)
**18 (B3)**	6,86	2,78	95	2	70	1		95	3	100	1
**19 (B3)**	5,61	2,49	10	1	15	1		10	1	5	1
**20 (B3)**	12,79	3,68	90	2	0	0		90	2	60	1
**21 (B3)**	7,15	2,84	70	1	80	1		80	1	90	2
**22 (TC)**	3,11	1,64	5	1	0	0		30	1	40	1
**23 (TC)**	3,23	1,69	30	2	20	1		30	2	70	2
**24 (TC)**	1,40	0,48	5	1	0	0		1	1	0	0
**25 (TC)**	0,96	-0,06	10	1	5	1		5	1	30	1
**26 (TC)**	3,19	1,67	5	1	3	1		1	1	70	1

TEAD4						
**Sample**	**FC**	**Log2FC**	**IHC**			
**1 (NG)**	0,84	-0,26				
**3 (NG)**	1,20	0,26	Nuclear	Staining Intensity	Cytoplasmic	Staining Intensity
**4 (A)**	2,17	1,12	0	0	100	2
**5 (A)**	5,07	2,34	90	2	90	2
**6 (A)**	3,33	1,74	90	2	100	2
**7 (B1)**	1,24	0,31	0	0	25	1
**8 (B1)**	1,28	0,36	15	1	10	0,5
**9 (B1)**	0,75	-0,41	20	1	50	1
**10 (B1)**	2,13	1,09	0	0	40	1
**11 (B1)**	0,62	-0,69	0	0	50	1
**12 (B2)**	0,88	-0,19	0	0	70	2
**13 (B2)**	0,27	-1,90	0	0	30	2
**14 (B2)**	1,01	0,01	0	0	70	1
**15 (B2)**	4,97	2,31	0	0	30	1
**16 (B2)**	1,39	0,48	0	0	20	1
**17 (B3)**	1,38	0,47	90	2	100	2
**18 (B3)**	0,96	-0,06	80	1	100	3
**19 (B3)**	3,84	1,94	10	1	100	2
**20 (B3)**	5,21	2,38	0	0	90	1
**21 (B3)**	2,93	1,55	60	1	90	2
**22 (TC)**	4,83	2,27	50	1	100	3
**23 (TC)**	17,48	4,13	60	2	80	2
**24 (TC)**	3,05	1,61	0	0	100	2
**25 (TC)**	11,22	3,49	20	1	100	3
**26 (TC)**	12,19	3,61	5	1	100	2
