# Supplementary material for: Subtype-Dependent Expression Patterns of Core Hippo Pathway Components in Thymic Epithelial Tumors (TETs): An RT-qPCR Study
Source: Biomedicines. 2026 Jan 29;14(2):305. doi: 10.3390/biomedicines14020305 (PMC12937678; doi:10.3390/biomedicines14020305)
Supplement: Supplementary file 1 [file biomedicines-14-00305-s001.zip › Table S2 Reagent lot comparison.pdf]

Table S2. Reagent lot comparison. To assess lot-to-lot consistency of the qPCR reagents, two cDNA samples Samples 3 and 9, 50 ng input each) were analysed in technical triplicates using four independent reagent lots (Lot 1–4). The assays targeted *YAP1*, *MOB1A*, *TBP* and the reference gene *HPRT1* [RealTimePrimers.com (RTP)]. Intra-assay variation was low, with SDs of technical triplicates typically <0.30 Cq for all genes and both samples; only *YAP1* in sample 3 with Lot 1 showed a higher SD (0.92 Cq) at high Cq values. Between-lot variation of the mean Cq values was ≤0.73 Cq for sample 3 (*YAP1* 0.73; *MOB1*A 0.47; *TBP* 0.19; *HPRT1* 0.49) and ≤0.88 Cq for sample 9 (*YAP1* 0.65; *MOB1A* 0.41; *TBP* 0.48; *HPRT1* (RTP) 0.88). As all study samples were analysed by ΔΔCq normalisation to *HPRT1* (RTP) and *TBP*, this lot-to-lot variation was considered acceptable,and data generated with different reagent lots were pooled for downstream analyses.

Sample 3 (50ng)
Cq values are given as mean ± SD (n = 3 technical replicates).

Gene	Lot 1	Lot 2	Lot 3	Lot 4
YAP1	34.51 ± 0.92	33.83 ± 0.43	33.79 ± 0.17	34.10 ± 0.28
MOB1A	28.47 ± 0.25	28.90 ± 0.19	28.94 ± 0.18	28.93 ± 0.10
TBP	32.18 ± 0.25	32.37 ± 0.18	32.20 ± 0.14	32.25 ± 0.11
HPRT1 (RTP)	29.50 ± 0.09	29.32 ± 0.07	29.43 ± 0.01	29.01 ± 0.08

Sample 9 (50ng)
Cq values are given as mean ± SD (n = 3 technical replicates).

Gene	Lot 1	Lot 2	Lot 3	Lot 4
YAP1	31.32 ± 0.06	30.93 ± 0.17	31.00 ± 0.12	30.67 ± 0.06
MOB1A	26.67 ± 0.07	26.55 ± 0.12	26.51 ± 0.14	26.27 ± 0.05
TBP	29.60 ± 0.16	29.75 ± 0.19	29.68 ± 0.08	29.27 ± 0.12
HPRT1 (RTP)	27.12 ± 0.24	27.08 ± 0.08	27.18 ± 0.04	26.30 ± 0.09
