# Supplementary material for: Subtype-Dependent Expression Patterns of Core Hippo Pathway Components in Thymic Epithelial Tumors (TETs): An RT-qPCR Study
Source: Biomedicines. 2026 Jan 29;14(2):305. doi: 10.3390/biomedicines14020305 (PMC12937678; doi:10.3390/biomedicines14020305)
Supplement: Supplementary file 1 [file biomedicines-14-00305-s001.zip › Table S3 Inter-run variability.pdf]

Table S3. Inter-run variability through blocks. To assess the stability of the reference genes across qPCR plates, Cq values of *TBP* and *HPRT1* [RealTimePrimers.com (RTP)] were extracted for all samples and runs. Agreement between the two reference genes was evaluated by calculating the correlation between *TBP* and *HPRT1* (RTP) Cq values cross all measurements. In the absence of a pooled inter-run calibrator, potential plate effects were assessed by comparing reference-gene Cq distributions across plates. *TBP* and *HPRT1* (RTP) Cq values were highly correlated (Spearman's correlation coefficient rho=0.88, p <0,001 for the first block and rho=0.89, p <0.001 for the second block) and showed acceptable inter-run deviations (Kruskal–Wallis, p >0.10 in all cases) indicating that both reference genes responded concordantly to biological and technical variability between runs and plates.

**First Block**		
Plate (1)		
Sample	TBP	HPRT1 RTP
1 (NG)	31,81	29,57
2 (NG)	32,78	30,79
Plate (2)		
Sample	TBP	HPRT1 RTP
3 (NG)	32,2	29,79
4 (A)	32,58	30,27
Plate (3)		
Sample	TBP	HPRT1 RTP
5 (A)	33,34	31,88
6 (A)	31,97	29,96
Plate (4)		
Sample	TBP	HPRT1 RTP
9 (B1)	29,15	27,11
10 (B1)	35,1	30,9
Plate (5)		
Sample	TBP	HPRT1 RTP
11 (B1)	31,16	28,42
12 (B2)	28,45	26,58
Plate (6)		
Sample	TBP	HPRT1 RTP
14 (B2)	32,03	28,04
Plate (7)		
Sample	TBP	HPRT1 RTP
15 (B2)	34,21	31,41
Plate (8)		
Sample	TBP	HPRT1 RTP
17 (B3)	31,78	28,45
18 (B3)	29,17	27,33

**Second Block**		
Plate (1)		
Sample	TBP	HPRT1 RTP
1 (NG)	30,1	30,36
2 (NG)	30,64	31,78
3 (NG)	31,41	31,59
5 (A)	31,46	33,2
Plate (2)		
Sample	TBP	HPRT1 RTP
4 (A)	29,11	29,81
6 (A)	29,2	30,05
7 (B1)	28,46	28,87
8 (B1)	31,22	31,54
9 (B1)	28,88	29,67
Plate (3)		
Sample	TBP	HPRT1 RTP
11 (B1)	30,56	29,74
12 (B2)	28,4	28,74
13 (B2)	32,08	32,3
14 (B2)	30,95	29,78
15 (B2)	32,26	32,93
Plate (4)		
Sample	TBP	HPRT1 RTP
16 (B2)	32,19	32,28
17 (B3)	30,7	30,16
18 (B3)	28,28	29,18
19 (B3)	30,75	29,94
Plate (5)		
Sample	TBP	HPRT1 RTP
10 (B1)	32,32	31,93
20 (B3)	31,12	31,54
26 (TC)	35,33	35,21
11 (Repitition)	30,64	30,08

Plate (9)			
Sample	TBP		HPRT1 RTP
19 (B3)		31,79	28,53
Plate (10)			
Sample	TBP		HPRT1 RTP
21 (B3)		33,77	30,49
22 (TC)		32,39	29,53
Plate (11)			
Sample	TBP		HPRT1 RTP
23 (TC)		33,94	30,71
26 (TC)		34,21	30,6
Plate (12)			
Sample	TBP		HPRT1 RTP
24 (TC)		32,76	29,81
25 (TC)		34,23	28,84
Plate (13)	WDH		
Sample	TBP		HPRT1 RTP
13 (B2)		31,68	29,71
16 (B2)		30,32	27,75
Plate (14)	WDH		
Sample	TBP		HPRT1 RTP
7 (B1)		31,49	28,28
8 (B1)		31,1	28,55
23 (TC)		33,67	30,14
Plate (15)			
Sample	TBP		HPRT1 RTP
12 (B2)		28,13	26,35
1 (NG)		29,26	27,16
Plate (16)			
Sample	TBP		HPRT1 RTP
14 (B2)		30,96	27,29
