# Supplementary material for: Subtype-Dependent Expression Patterns of Core Hippo Pathway Components in Thymic Epithelial Tumors (TETs): An RT-qPCR Study
Source: Biomedicines. 2026 Jan 29;14(2):305. doi: 10.3390/biomedicines14020305 (PMC12937678; doi:10.3390/biomedicines14020305)
Supplement: Supplementary file 1 [file biomedicines-14-00305-s001.zip › Table S4 Cross-instrument comparison.pdf]

Table S4. Cross-instrument comparison of Cq values between CFX96 cycler 1 and cycler 2.
Sample 3 and 9 were tested in technical triplicates for inter-instrument comparison. For each gene and sample, mean Cq values and standard deviations were calculated from triplicate measurements using the sample standard deviation, and the difference in Cq between instruments was determined. Across all eight gene–sample combinations, the mean absolute difference in Cq values was 0.31 cycles (range, 0.01–0.72), with |ΔCq| < 0.8 for all comparisons and SD < 0.30 Cq in 7/8 gene–sample combinations (maximum SD, 0.92 Cq for YAP1 in sample 3 on cycler 2), indicating high inter-instrument comparability.

Sample	Gene	Cycler 1 (mean ± SD)	Cycler 2 (mean ± SD)	ΔCq (C2 – C1)	|ΔCq|
**3**	YAP1	34.13 ± 0.36	34.51 ± 0.92	+0.38	0.38
**3**	MOB1A	28.63 ± 0.03	28.47 ± 0.25	−0.16	0.16
**3**	TBP	32.20 ± 0.13	32.18 ± 0.25	−0.02	0.02
**3**	HPRT1	29.79 ± 0.10	29.50 ± 0.09	−0.29	0.29
**9**	YAP1	30.60 ± 0.15	31.32 ± 0.06	+0.72	0.72
**9**	MOB1A	26.20 ± 0.12	26.67 ± 0.07	+0.47	0.47
**9**	TBP	29.15 ± 0.20	29.60 ± 0.16	+0.44	0.44
**9**	HPRT1	27.11 ± 0.06	27.12 ± 0.24	+0.01	0.01

* Cq values are given as mean ± SD (n = 3 technical replicates). ΔCq = mean Cq(cycler 2) – mean Cq(cycler 1).
