# Supplementary material for: Subtype-Dependent Expression Patterns of Core Hippo Pathway Components in Thymic Epithelial Tumors (TETs): An RT-qPCR Study
Source: Biomedicines. 2026 Jan 29;14(2):305. doi: 10.3390/biomedicines14020305 (PMC12937678; doi:10.3390/biomedicines14020305)
Supplement: Supplementary file 1 [file biomedicines-14-00305-s001.zip › Table S5 Calculation of internal controls (ICs).pdf]

Table S5. Calculation of internal controls (ICs). An internal amplification control (QuantiNova Internal Control RNA, QN IC RNA) of the QuantiNova® LNA® PCR Reference Assay (QIAGEN, Hilden, Germany; Ref. No. 249920) was included once for every sample. The QN IC RNA monitors the efficiency of reverse transcription and amplification and is intended to detect instrument or reagent malfunction, assay setup errors, and the presence of PCR inhibitors. It is detected as a 200 bp amplicon. Assay performance was considered acceptable for ΔCq (sample − IC) < 2. Two FFPE-derived specimens showed ΔCq values of 2.11 and 2.46. n view of the known variability associated with FFPE material, these values were judged acceptable and the samples were retained for analysis.

IC H20		Diff IC	Mean IC H20	Sample Number	IC Sample	Diff Sample
				1	26,94	0,41
25,96	27,11	-1,15	26,535	2	28,16	1,63
				3	26,97	1,47
25,57	25,43	0,14	25,5	4	27,26	1,76
				5	27,22	2,11
25,06	25,16	-0,1	25,11	6	26,53	1,42
24,43	24,91	-0,48	24,67	7	26,3	1,63
24,38	24,55	0,17	24,465	8	25,21	0,75
				9	25,83	0,79
24,95	25,12	-0,17	25,035	10	25	0,04
				11	26,29	1,49
24,66	24,94	-0,28	24,8	12	25,4	0,60
23,57	23,47	0,1	23,52	13	24,2	0,68
27,9	25,31	2,59	26,605	14	26,13	2,61
25,75	25,8	-0,05	25,775	15	26,45	0,68
23,57	23,47	0,1	23,52	16	24,08	0,56
				17	25,98	0,54
25,35	25,52	-0,17	25,435	18	25,19	0,25
				19	25,54	0,17
25,32	25,42	-0,1	25,37	20	25,75	0,38
				21	27,65	1,37
26,32	26,25	0,07	26,285	22	26,75	0,47
24,53	24,69	-0,16	24,61	23	25,11	0,50
				24	27,71	1,28
26,39	26,47	-0,08	26,43	25	26,73	0,30
24,53	24,69	-0,16	24,61	26	27,07	2,46
**Repetition of sample 14**						
23,88	23,81	-0,07	23,845	14	24,67	0,83
