# Supplementary material for: Subtype-Dependent Expression Patterns of Core Hippo Pathway Components in Thymic Epithelial Tumors (TETs): An RT-qPCR Study
Source: Biomedicines. 2026 Jan 29;14(2):305. doi: 10.3390/biomedicines14020305 (PMC12937678; doi:10.3390/biomedicines14020305)
Supplement: Supplementary file 1 [file biomedicines-14-00305-s001.zip › Table S6 Primer assay oligonucleotide sequences.pdf]

Table S6. Primer assay oligonucleotide sequences.

Target	Company	Assay Identification Number	Amplicon size (bp)	Sequence Forward Primer	Sequence Reverse Primer	Exon Location	RefSeq Number
STE20-like kinase 1 *(MST1/ STK4)*	Integrated DNA Technologies (IDT™)	Hs.PT.58.20785666	141	5'-AGCCCCACAGTACTCCAT-3'	5'-GTTCCTGTGGAATCAGACCTC-3'	3-4	NM_0006282
Salvador family WW domain-containing protein 1 *(SAV1)*	(IDT™)	Hs.PT.58.45488696	105	5'-AGATCGTGCACATGAAGACTAC-3'	5'-GCAACTCTCCCAATACCTGAA-3'	2-3	NM_021818
Large tumor suppressor kinase 1 *(LATS1)*	(IDT™)	Hs.PT.58.40644872	137	5'-TTGCTGGGACGGACTCT-3'	5'-TTCAAGGAAGTCCCCAGGA-3'	3-5	NM_004690; NM_001270519
MOB kinase activator 1A *(MOB1A)*	(IDT™)	Hs.PT.58.40138473	112	5'-TTGATCTGGTTAAAGAAATCCACAG-3'	5'-CAGAAGCAACTCTAGGAAGTGG-3'	2-3	NM_018221
Yes-associated protein 1 *(YAP1)*	(IDT™)	Hs.PT.58.22607088	104	5'-TGACTGATTCTCTGGTTCATGG-3'	5'-AGGAAGGCCATGCTGTC-3'	4-6	NM_006106
TEA domain transcription factor 4 *(TEAD4)*	(IDT™)	Hs.PT.58.23238289	107	5'-AGCTGCCTTCATCCTCGAT-3'	5'-GAAAAAGGGTGGACTCAAGGA-3'	10-11	NM_201443
Hypoxanthine-guanine phosphoribosyltransferase 1 (*HPRT1*)	RealTimePrimers.com	VHPS-4263	94	5'-TGACACTGGCAAAACAATGCA-3'	5'-GGTCCTTTTCACCAGCAAGCT-3'	4-5	NM_000194.1
TATA-box binding protein *(TBP)*	(IDT™)	Hs.PT.58.20792004	113	5'-TCGTGGCTCTCTTATCCTCAT-3'	5'-CAGTGAATCTTGGTTGTAAACTTGA-3'	4-5	NM_003194

HPRT1[1]	(IDT™)	Hs.PT.58.20881146	111	5'-CCAATTACTTTTATGTCCCCTGTT-3'	5'-CATCAAAGCACTGAATAGAAATAGTGA-3'	3-4	NM_000194
Peptidyl-prolyl isomerase A *(PPIA)*[1]	(IDT™)	Hs.PT.39a.22214851	138	5'-TCTTTCACTTTGCCAAACACC-3'	5'-CATCCTAAAGCATACGGGTCC-3'	4-5	NM_21130
SAV1[2]	(IDT™)	Hs.PT.58.45727297	92	5'-GAATGAAGGCATAAGATTCCGAAG-3'	5'-CCAAAAACGAAGTGTCCAAGC-3'	1b-2	NM_021818
Transcriptional co-activator with PDZ-binding motif *(TAZ/WWTR1)*[2]	(IDT™)	Hs.PT.58.1944253 /Hs.PT.58.19363927	130/124	5'-CTAAGAAGGCGATGAATCAGC-3'/5'-TCGACAGAGGGCAGCTT-3'	5'-TGCTGCTGGTGTTGGTG-3'/5'-CAATGCGCTGACCACTCA-3'	5-6/6-7	NM_015472; NM_001168280; NM_001168278
LATS1[2]	(IDT™)	Hs.PT.58.39498320	134	TTCAAGGAAGTCCCCAGGA	TTGCTGGGACGGACTCT	1-3	NM_004690

[1] Reference Genes not used for normalization due to higher instability ranking from RefFinder and/or gDNA amplification.

[2] Primer assays not used for further analysis due to gDNA amplification, primer-dimer amplification or non-specific amplification.
