# Supplementary material for: Subtype-Dependent Expression Patterns of Core Hippo Pathway Components in Thymic Epithelial Tumors (TETs): An RT-qPCR Study
Source: Biomedicines. 2026 Jan 29;14(2):305. doi: 10.3390/biomedicines14020305 (PMC12937678; doi:10.3390/biomedicines14020305)
Supplement: Supplementary file 1 [file biomedicines-14-00305-s001.zip › Table S7 Primer efficiency assessment.pdf]

Table S7. Primer efficiency assessment. To verify commercial primer assay performance under FFPE conditions, PCR efficiency was assessed using a 1:5 serial dilution (50–0.4 ng input cDNA) of a representative FFPE thymic sample (sample 19). For the reference gene *HPRT1* (RealTimePrimers.com), five dilution points with Cq values within the dynamic range and low replicate variation ($\Delta Cq \leq 1$) yielded an efficiency of approximately 107% (E = 1.07, $R^2 = 1.0$). For several target genes, the most diluted points showed increased replicate variability or loss of amplification, consistent with the limited amount of amplifiable template in FFPE-derived RNA, and therefore did not allow for robust efficiency estimation for every single assay. As all primer pairs nevertheless showed log-linear amplification over at least three dilution steps within a comparable Cq range, relative expression levels were calculated using the comparative Cq ($2^{-\Delta\Delta Cq}$) method under the assumption of approximately similar amplification efficiencies across assays (E ≈2).

pure	1:5	1:25	(1:125)	(1:625)
**YAP1**	**YAP1**	**YAP1**	**YAP1**	**YAP1**
29,81	31,24	33,24	34,72	36,83
Diff Cq :	1,43	2,00	1,47	2,11
Slope: -2,46	R^2: 0,99	E: 1,55	E: 155%	

pure	1:5	1:25	1:125
**SAV1**	**SAV1**	**SAV1**	**SAV1**
27,03	28,95	30,82	33,06
Diff Cq :	1,92	1,86	2,25
Slope: -2,86	R^2: 1,0	E: 1,24	E: 124%

pure	1:5	1:25	
**MST1**	**MST1**	**MST1**	
28,67	30,29	32,95	
Diff Cq :	1,62	2,66	
Slope: -3,06	R^2: 0,98	E: 1,22	E: 122%

pure	1:5	1:25	1:125
**TBP**	**TBP**	**TBP**	**TBP**
30,51	31,94	34,03	35,65
Diff Cq :	1,43	2,08	1,62
Slope: -2,52	R^2: 1,0	E: 1,51	E: 151%

pure	1:5	1:25	
**TEAD4**	**TEAD4**	**TEAD4**	
31,31	32,85	34,34	
Diff Cq :	1,54	1,49	
Slope: -2,17	R^2: 1,00	E: 1,89	E: 189%

pure	1:5	1:25	
**LATS1**	**LATS1**	**LATS1**	
30,39	32,07	33,87	
Diff Cq :	1,68	1,81	
Slope: -2,50	R^2: 1,00	E: 1,51	E: 152%

pure	1:5	1:25	1:125
**HPRT1 RTP**	**HPRT1 RTP**	**HPRT1 RTP**	**HPRT1 RTP**
29,66	31,71	33,99	36,08
Diff Cq :	2,05	2,28	2,09
Slope: -3,16	R^2: 1,00	E: 1,07	E: 107%

pure	1:5	1:25	1:125
**MOB1A**	**MOB1A**	**MOB1A**	**MOB1A**
27,20	28,64	30,95	33,20
Diff Cq :	1,43	2,31	2,25
Slope: -2,90	R^2: 0,99	E: 1,21	E: 121%
