# Supplementary material for: Subtype-Dependent Expression Patterns of Core Hippo Pathway Components in Thymic Epithelial Tumors (TETs): An RT-qPCR Study
Source: Biomedicines. 2026 Jan 29;14(2):305. doi: 10.3390/biomedicines14020305 (PMC12937678; doi:10.3390/biomedicines14020305)
Supplement: Supplementary file 1 [file biomedicines-14-00305-s001.zip › Table S8 Replicate-level QC and exclusion criteria of excluded housekeeping gene (HKG) samples excluded in analysis.pdf]

Table S8. Replicate-level QC and exclusion criteria of excluded housekeeping gene (HKG) samples excluded in analysis. Triplicates were assessed based on intra-assay variability. All three Cq values within ≤ 0.5 Cq were considered technically consistent; Any replicate deviating by > 0.5 Cq from the mean of the two most consistent replicates was defined as a technical outlier and excluded ("gold standard"). For FFPE-derived RNA, a total spread of up to 0.8 Cq was accepted ("FFPE accepted"). Reactions without two consistent replicates (ΔCq > 0.8) were classified as invalid and repeated.

Sample	Target	Cq1	Cq2	Cq3	Δmax Cq (all 3)	ΔCq best 2	Used replicates	Mean value	Comment
1	HPRT1-IDT	30.74	30.51	31.2	0.69	0.23	R1+R2	30.62	gold standard (outlier excluded)
1	PPIA	26.88	26.61	26.59	0.29	0.02	R1+R2+R3	26.69	gold standard (all ≤0,5)
2	HPRT1-IDT	32.37	32.61	32.28	0.33	0.09	R1+R2+R3	32.42	gold standard (all ≤0,5)
2	PPIA	29.33	28.73	28.8	0.6	0.07	R2+R3	28.77	gold standard (outlier excluded)
3	HPRT1-IDT	30.86	31.38	31.08	0.52	0.22	R1+R2+R3	31.11	FFPE accepted
3	PPIA	27.51	27.29	27.49	0.22	0.02	R1+R2+R3	27.43	gold standard (all ≤0,5)
4	HPRT1-IDT	31.74	32.1	32.1	0.36	0.0	R1+R2+R3	31.98	gold standard (all ≤0,5)
4	PPIA	27.7	27.79	27.7	0.09	0.0	R1+R2+R3	27.73	gold standard (all ≤0,5)
5	HPRT1-IDT	33.16	33.27	33.84	0.68	0.11	R1+R2	33.22	gold standard (outlier excluded)
5	PPIA	28.48	28.33	28.9	0.57	0.15	R1+R2+R3	28.57	FFPE accepted
6	HPRT1-IDT	31.5	31.49	32.01	0.52	0.01	R1+R2	31.49	gold standard (outlier excluded)
6	PPIA	26.51	26.38	30.66	4.28	0.13	R1+R2	26.45	gold standard (outlier excluded)
7	HPRT1-IDT	29.42	31.82	30.01	2.4	0.59	R1+R3	29.72	FFPE accepted (outlier excluded)
7	PPIA	26.55	27.02	26.48	0.54	0.07	R1+R3	26.52	gold standard (outlier excluded)
8	HPRT1-IDT	30.54	30.54	31.64	1.1	0.0	R1+R2	30.54	gold standard (outlier excluded)
8	PPIA	26.44	26.29	26.26	0.18	0.03	R1+R2+R3	26.33	gold standard (all ≤0,5)
9	HPRT1-IDT	27.95	28.16	27.8	0.36	0.15	R1+R2+R3	27.97	gold standard (all ≤0,5)

**9**	PPIA	23.79	23.89	23.7	0.19	0.09	R1+R2+R3	23.79	gold standard (all ≤0,5)
**10**	HPRT1-IDT	32.8	32.08	32.24	0.72	0.16	R2+R3	32.16	gold standard (outlier excluded)
**10**	PPIA	29.67	29.58	29.83	0.25	0.09	R1+R2+R3	29.69	gold standard (all ≤0,5)
**11**	HPRT1-IDT	28.34	28.28	28.44	0.16	0.06	R1+R2+R3	28.35	gold standard (all ≤0,5)
**11**	PPIA	26.41	26.45	26.62	0.21	0.04	R1+R2+R3	26.49	gold standard (all ≤0,5)
**12**	HPRT1-IDT	27.18	26.73	27.11	0.45	0.07	R1+R2+R3	27.01	gold standard (all ≤0,5)
**12**	PPIA	22.92	28.4	23.13	5.48	0.21	R1+R3	23.02	gold standard (outlier excluded)
**13**	HPRT1-IDT	31.02	31.09	31.12	0.1	0.03	R1+R2+R3	31.08	gold standard (all ≤0,5)
**13**	PPIA	27.31	27.19	27.14	0.17	0.05	R1+R2+R3	27.21	gold standard (all ≤0,5)
**14**	HPRT1-IDT	29.01	29.29	29.21	0.28	0.08	R1+R2+R3	29.17	gold standard (all ≤0,5)
**14**	PPIA	25.91	25.83	25.68	0.23	0.08	R1+R2+R3	25.81	gold standard (all ≤0,5)
**15**	HPRT1-IDT	29.54	29.33	29.53	0.21	0.01	R1+R2+R3	29.47	gold standard (all ≤0,5)
**15**	PPIA	39.06	33.33	33.74	5.73	0.41	R2+R3	33.53	gold standard (outlier excluded)
**16**	HPRT1-IDT	29.37	29.71	29.09	0.62	0.28	R1+R2+R3	29.39	FFPE accepted
**16**	PPIA	25.35	25.33	25.28	0.07	0.02	R1+R2+R3	25.32	gold standard (all ≤0,5)
**17**	HPRT1-IDT	30.08	29.7	30.11	0.41	0.03	R1+R2+R3	29.96	gold standard (all ≤0,5)
**17**	PPIA	25.98	25.96	25.76	0.22	0.02	R1+R2+R3	25.9	gold standard (all ≤0,5)
**18**	HPRT1-IDT	28.85	28.63	28.52	0.33	0.11	R1+R2+R3	28.67	gold standard (all ≤0,5)
**18**	PPIA	23.9	23.74	23.7	0.2	0.04	R1+R2+R3	23.78	gold standard (all ≤0,5)
**19**	HPRT1-IDT	29.52	29.79	30.08	0.56	0.27	R1+R2+R3	29.8	FFPE accepted
**19**	PPIA	25.43	25.46	25.57	0.14	0.03	R1+R2+R3	25.49	gold standard (all ≤0,5)

**20**	HPRT1-IDT	31.94	31.88	31.76	0.18	0.06	R1+R2+R3	31.86	gold standard (all ≤0,5)
**20**	PPIA	27.53	27.7	27.49	0.21	0.04	R1+R2+R3	27.57	gold standard (all ≤0,5)
**21**	HPRT1-IDT	32.5	32.45	33.52	1.07	0.05	R1+R2	32.48	gold standard (outlier excluded)
**21**	PPIA	27.89	27.92	27.86	0.06	0.03	R1+R2+R3	27.89	gold standard (all ≤0,5)
**22**	HPRT1-IDT	31.29	31.43	31.46	0.17	0.03	R1+R2+R3	31.39	gold standard (all ≤0,5)
**22**	PPIA	28.0	27.7	27.16	0.84	0.3	R1+R2	27.85	gold standard (outlier excluded))
**23**	HPRT1-IDT	34.21	34.24	34.61	0.4	0.03	R1+R2+R3	34.35	gold standard (all ≤0,5)
**23**	PPIA	27.3	27.48	27.14	0.34	0.16	R1+R2+R3	27.31	gold standard (all ≤0,5)
**24**	HPRT1-IDT	38.02	37.46	37.74	0.56	0.28	R1+R2+R3	37.74	FFPE accepted
**24**	PPIA	28.61	28.36	28.23	0.38	0.13	R1+R2+R3	28.4	gold standard (all ≤0,5)
**25**	HPRT1-IDT	32.98	32.99	32.6	0.39	0.01	R1+R2+R3	32.86	gold standard (all ≤0,5)
**25**	PPIA	28.57	28.34	28.31	0.26	0.03	R1+R2+R3	28.41	gold standard (all ≤0,5)
**26**	HPRT1-IDT	33.09	33.18	33.37	0.28	0.09	R1+R2+R3	33.21	gold standard (all ≤0,5)
**26**	PPIA	27.94	28.0	27.94	0.06	0.0	R1+R2+R3	27.96	gold standard (all ≤0,5)
