# Supplementary material for: Subtype-Dependent Expression Patterns of Core Hippo Pathway Components in Thymic Epithelial Tumors (TETs): An RT-qPCR Study
Source: Biomedicines. 2026 Jan 29;14(2):305. doi: 10.3390/biomedicines14020305 (PMC12937678; doi:10.3390/biomedicines14020305)
Supplement: Supplementary file 1 [file biomedicines-14-00305-s001.zip › Table S9 Housekeeping gene (HKG) Cq datasets used for RefFinder stability analysis. .pdf]

Table S9. Housekeeping gene (HKG) Cq datasets used for RefFinder stability analysis. The table presents the mean Cq values calculated from valid technical replicates for each target gene. These values served as the input dataset for the RefFinder algorithm to determine reference gene stability.

Sample	TBP	HPRT1 (RTP)	HPRT1 (IDT)	PPIA
1 (NG)	31.81	29.57	30.62	26.69
2 (NG)	32.78	30.79	32.42	28.77
3 (NG)	32.20	29.79	31.11	27.43
4 (A)	32.58	30.27	31.98	27.73
5 (A)	33.34	31.88	33.22	28.57
6 (A)	31.97	29.96	31.49	26.45
7 (B1)	31.25	29.15	29.72	26.52
8 (B1)	31.10	28.55	30.54	26.33
9 (B1)	29.15	27.11	27.97	23.79
10 (B1)	35.10	30.90	32.16	29.69
11 (B1)	31.16	28.42	28.35	26.49
12 (B2)	28.45	26.58	27.01	23.02
13 (B2)	31.68	29.71	31.08	27.21
14 (B2)	32.03	28.04	29.17	25.81
15 (B2)	34.21	31.41	29.47	33.53
16 (B2)	30.32	27.75	29.39	25.32
17 (B3)	31.78	28.45	29.96	25.09
18 (B3)	29.17	27.33	28.67	23.78
19 (B3)	31.79	28.53	29.09	25.49
20 (B3)	31.12	31.54	31.86	27.57
21 (B3)	33.77	30.49	32.48	27.89
22 (TC)	32.39	29.53	31.39	27.85
23 (TC)	33.94	30.71	34.35	27.31
24 (TC)	32.76	29.81	37.74	28.04
25 (TC)	34.23	28.84	32.86	28.41
26 (TC)	34.21	30.60	33.21	27.96
